# Supplementary material for: Hypoxia truncates and constitutively activates the key cholesterol synthesis enzyme squalene monooxygenase
Source: eLife. 2023 Jan 19;12:e82843. doi: 10.7554/eLife.82843 (PMC9851614; doi:10.7554/eLife.82843)

Figure 1B – SM

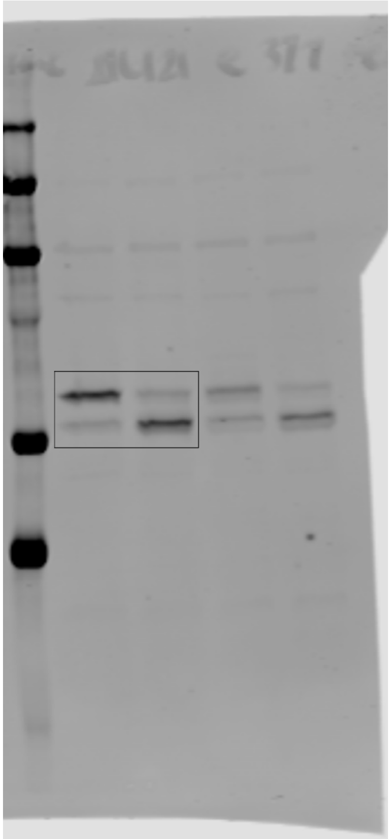

Figure 1B – HIF1α

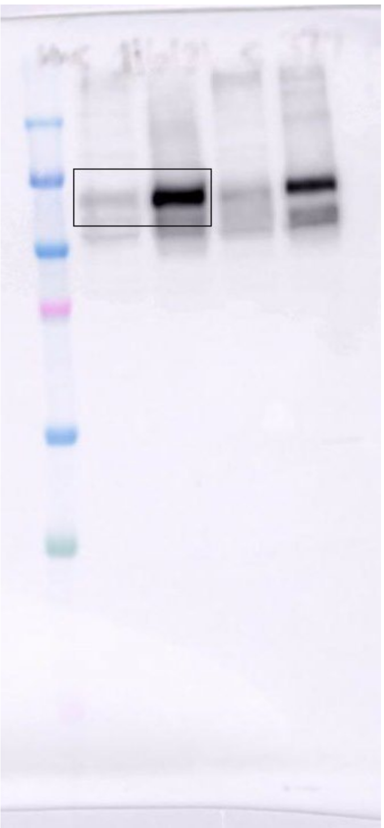

Figure 1B – GAPDH

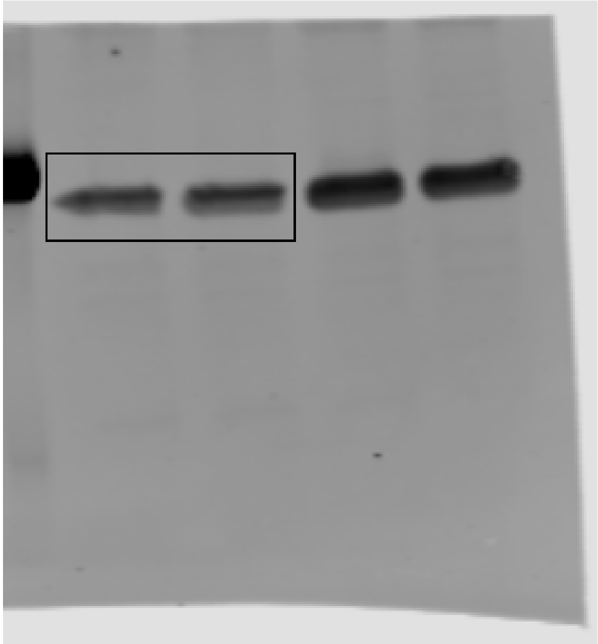

Figure 1C – SM

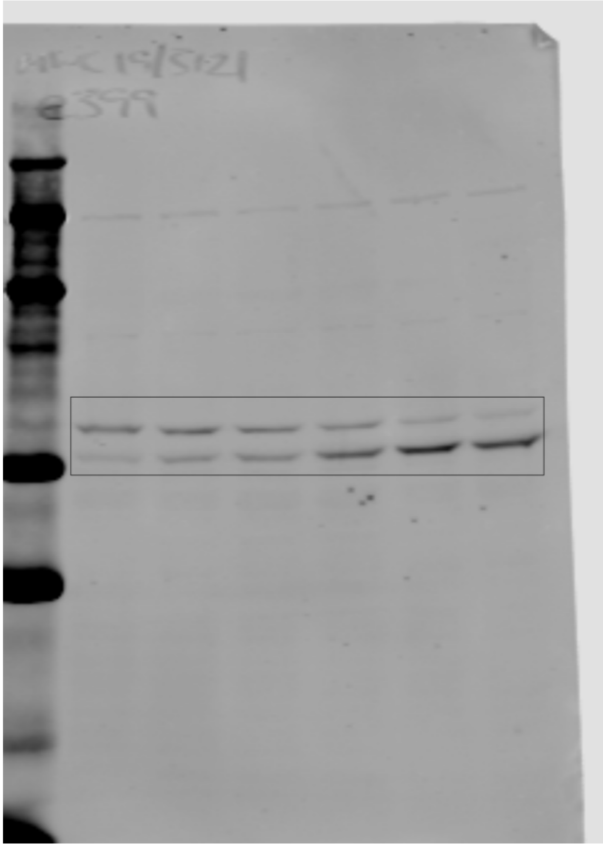

Figure 1C – HIF1α

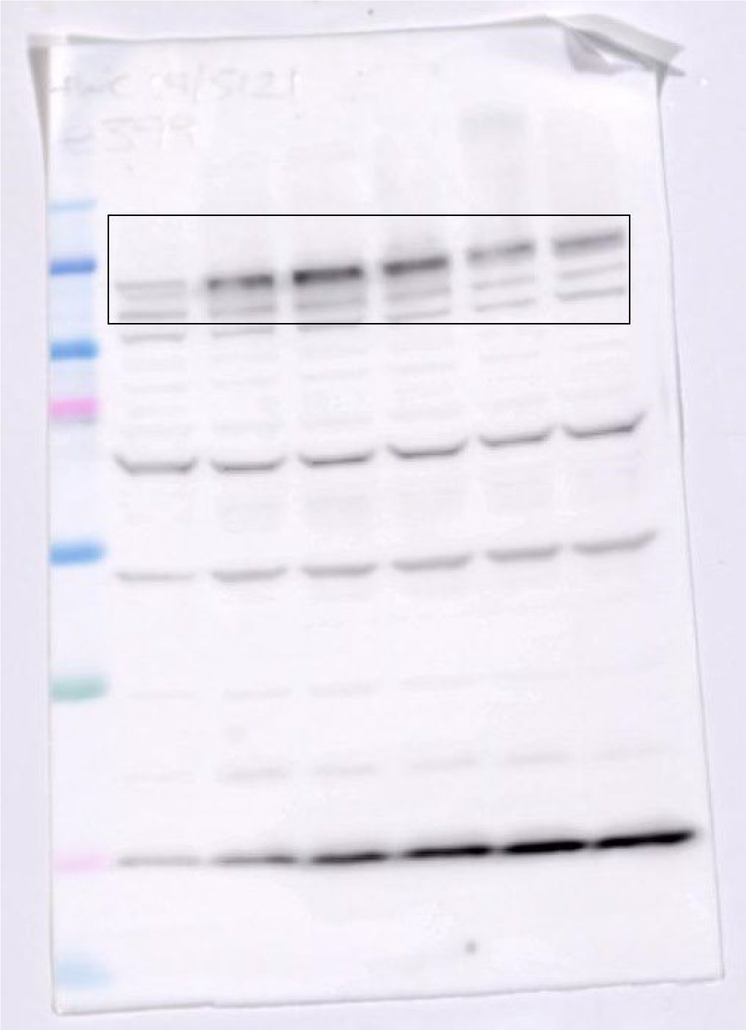

Figure 1C – GAPDH

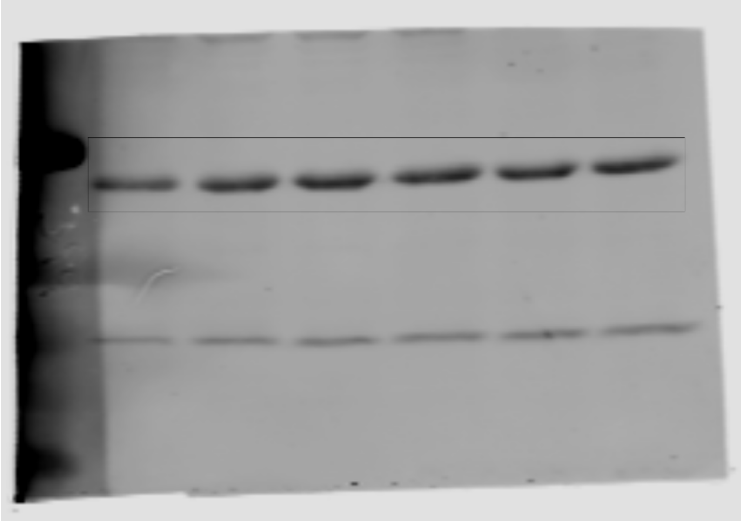

Figure 1D – 0.5% O_2 – SM

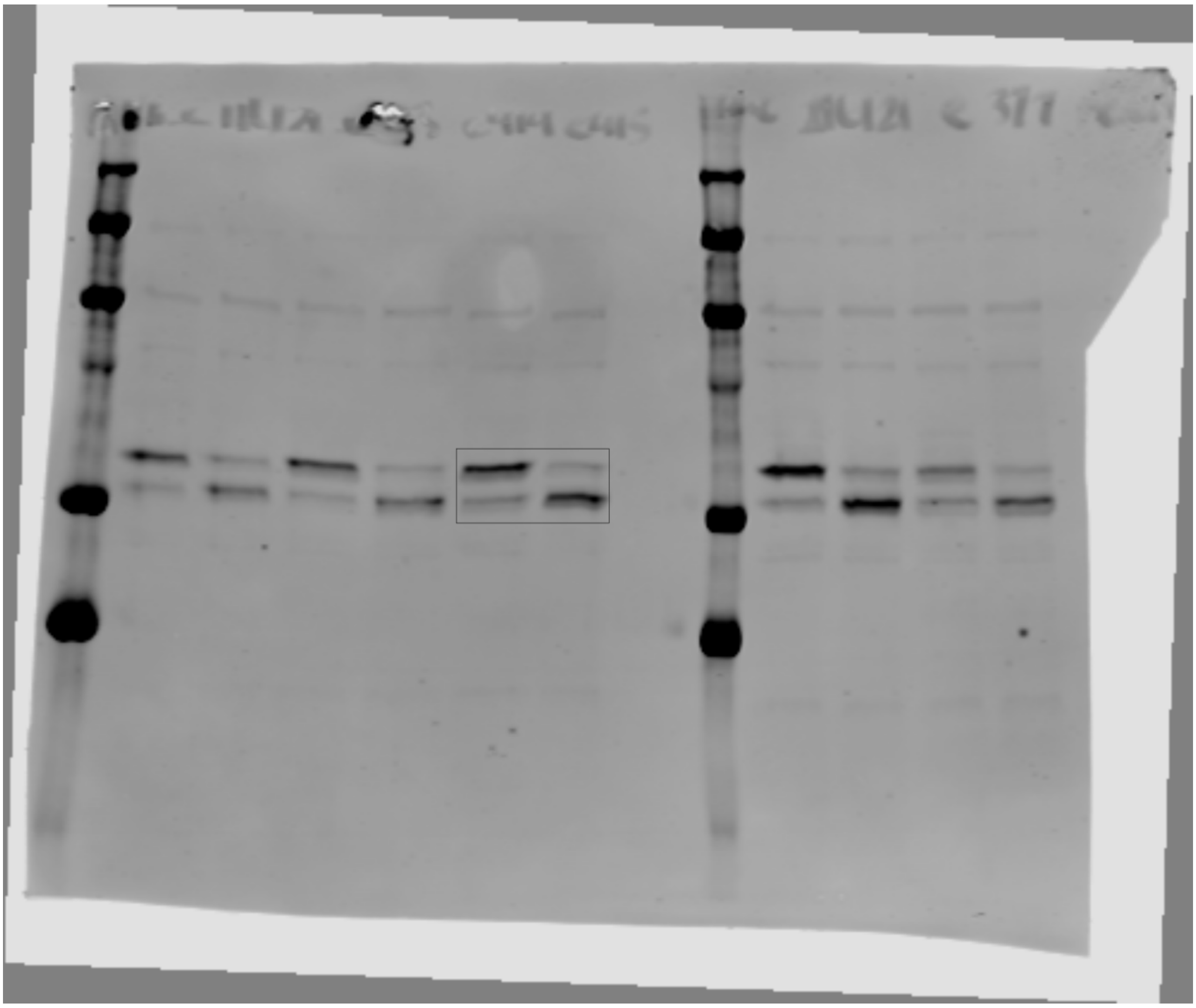

Figure 1D – 0.5% O_2 – HIF1α

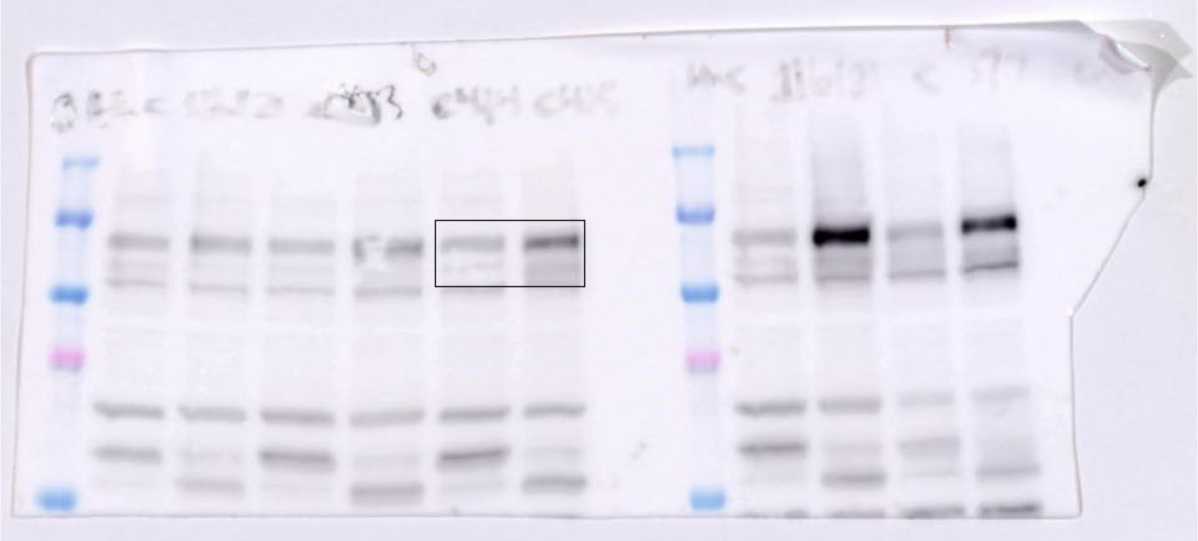

Figure 1D – 0.5% O_2 – GAPDH

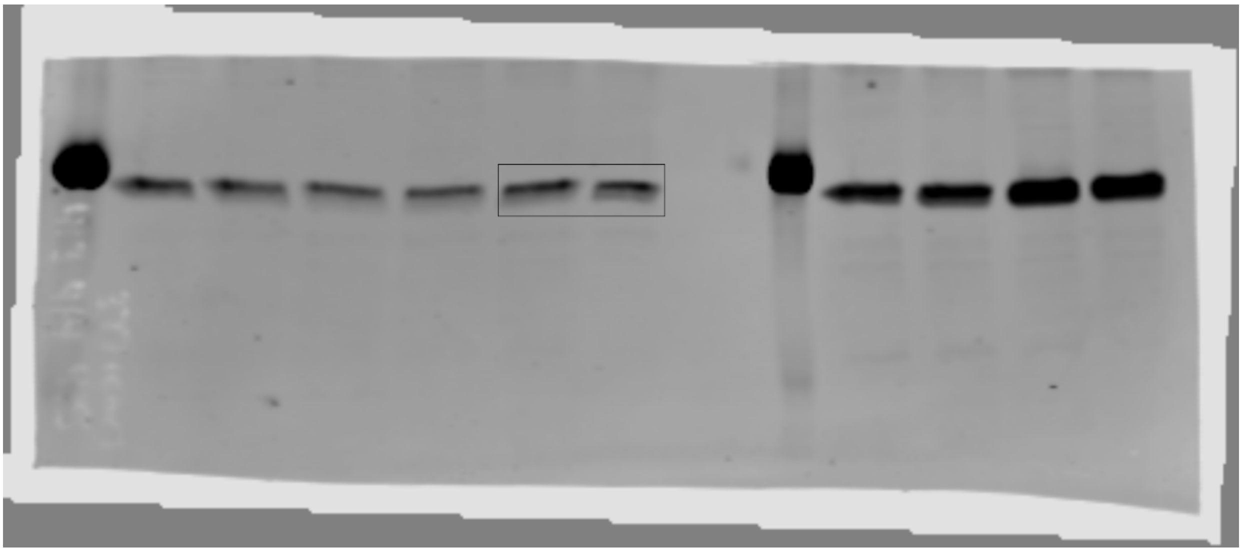

Figure 1D – 1% O_2 – SM

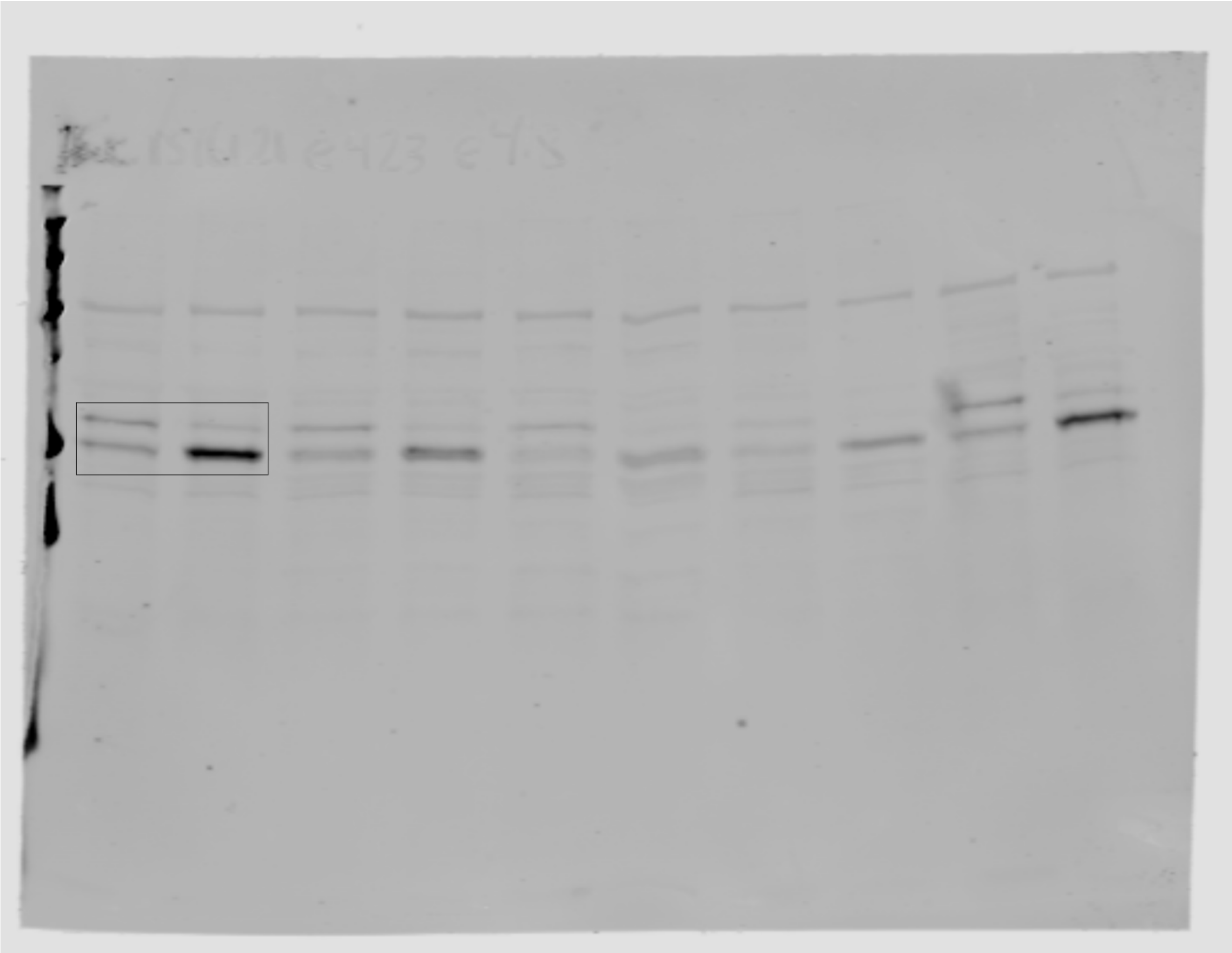

Figure 1D – 1% O_2 – HIF1α

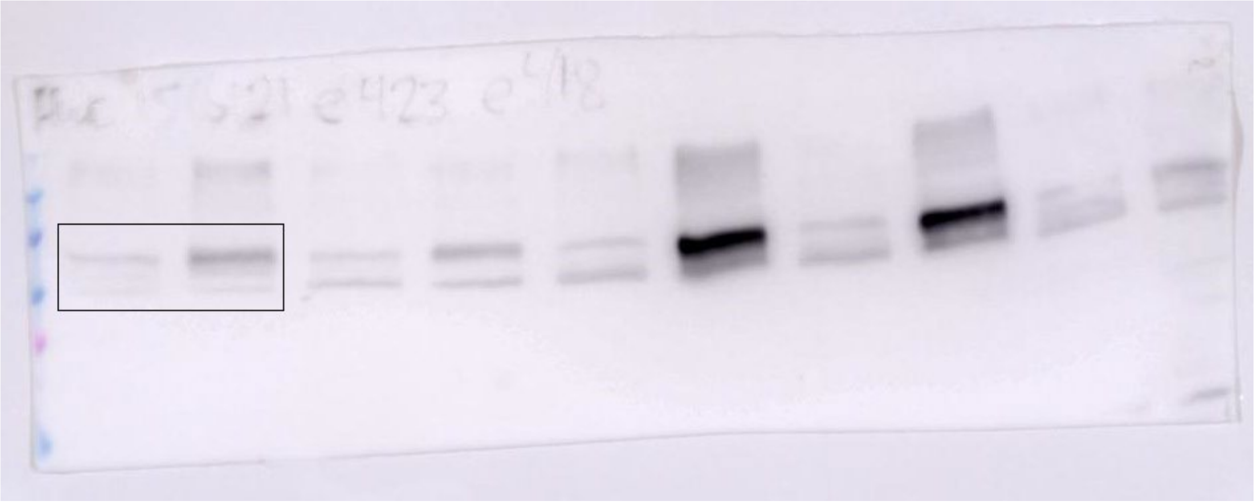

Figure 1D – 1% O_2 – GAPDH

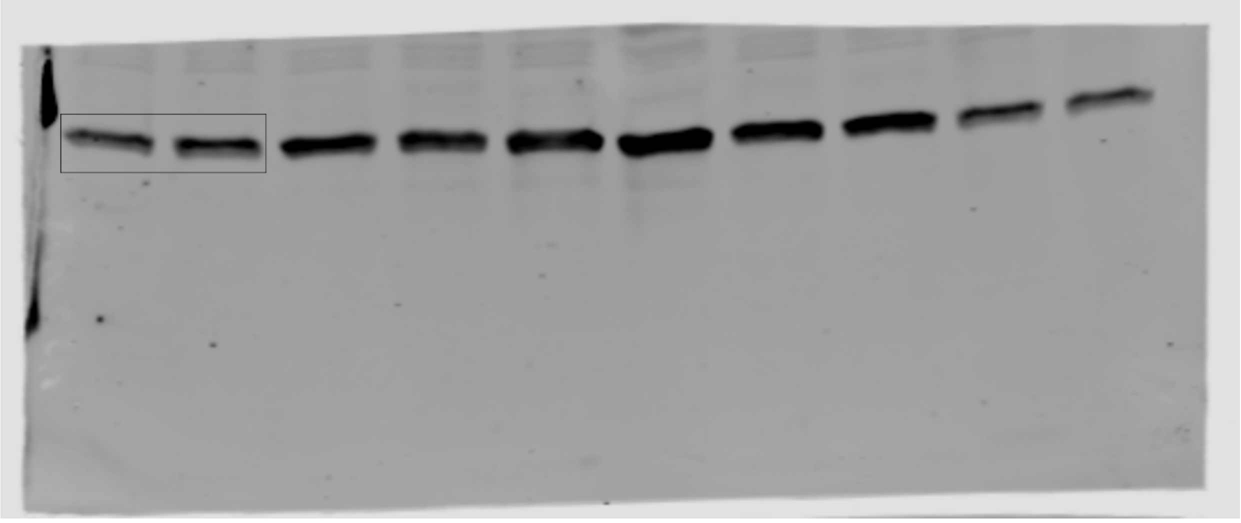

Figure 1D – 2% O_2 – SM

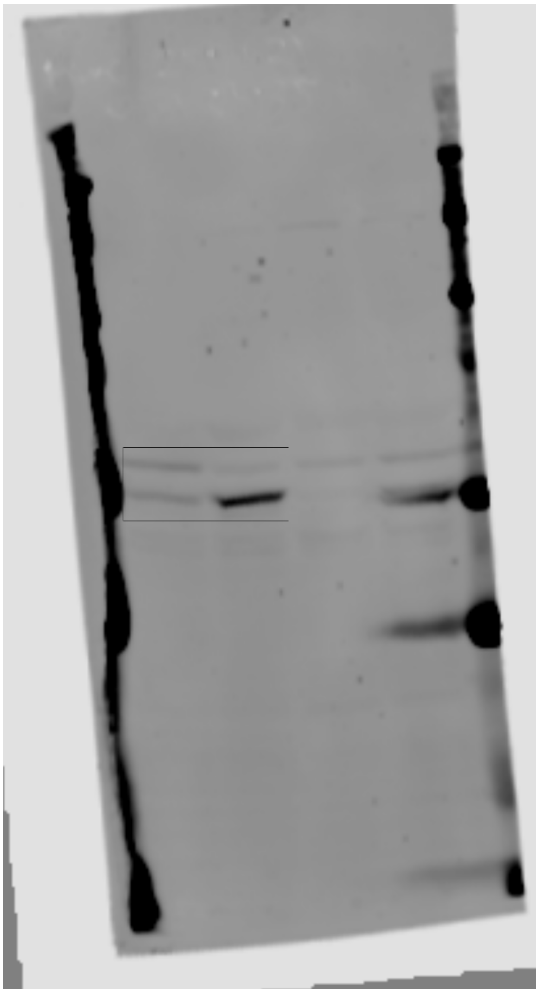

Figure 1D – 2% O_2 – HIF1α

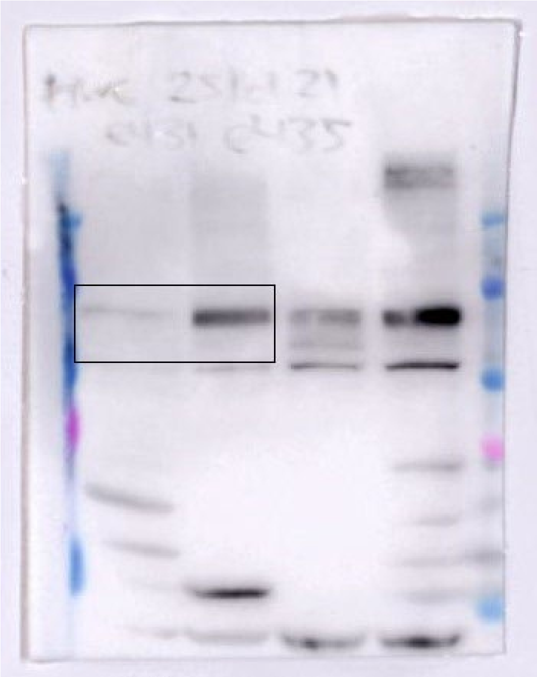

Figure 1D – 2% O_2 – GAPDH

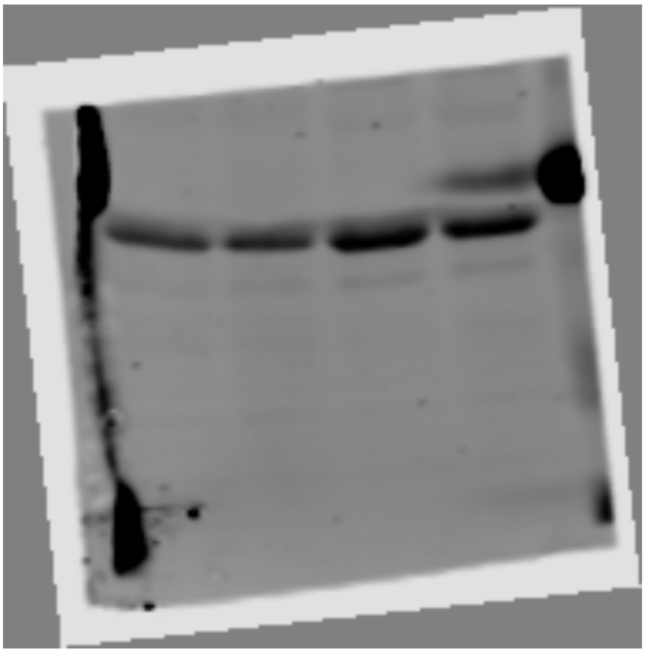

Figure 1D – 3% O_2 – SM

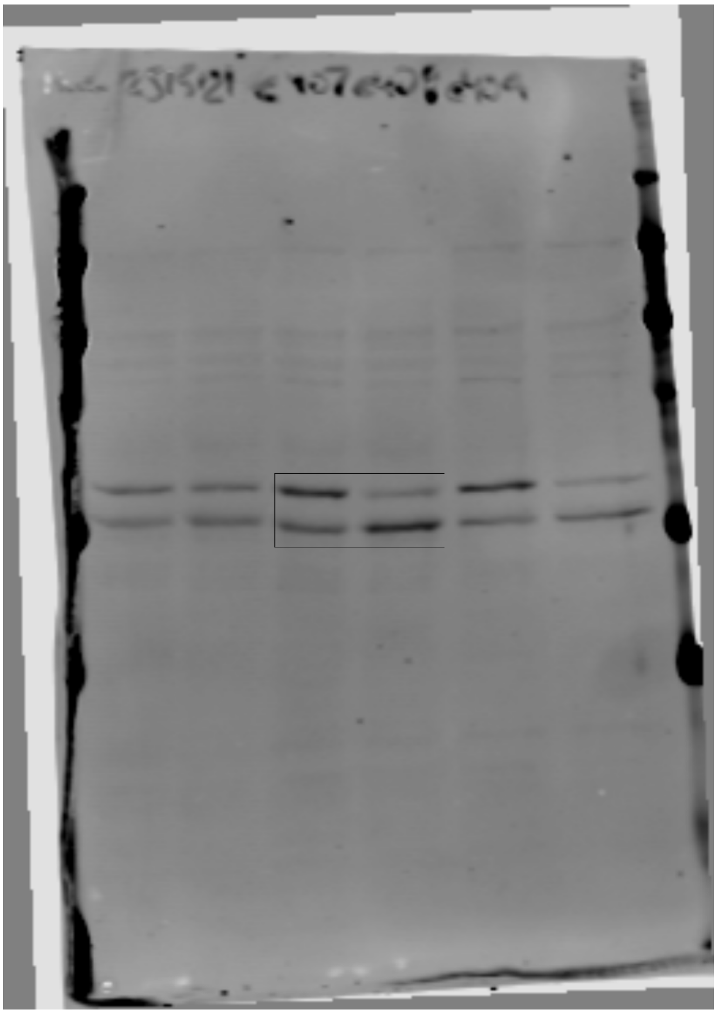

Figure 1D – 3% O_2 – HIF1α

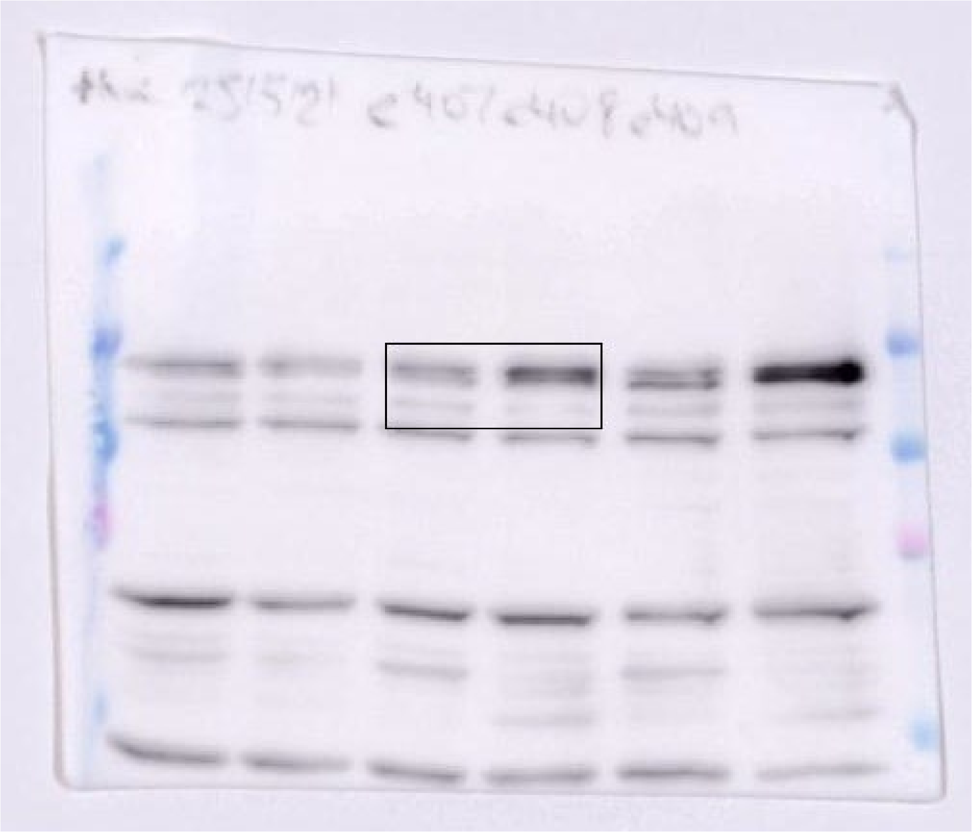

Figure 1D – 3% O_2 – GAPDH

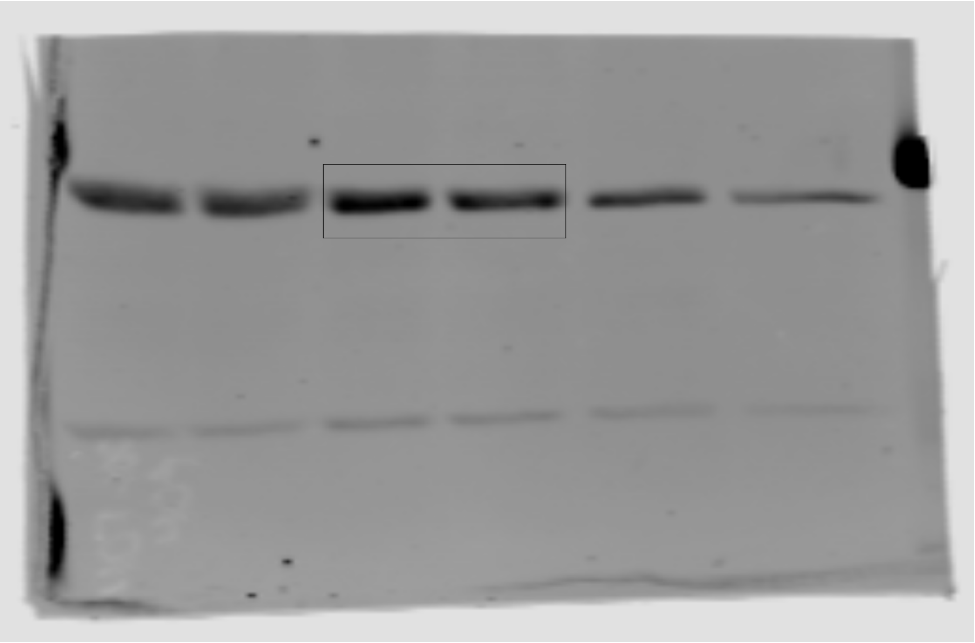

Figure 1D – 4% O_2 – SM

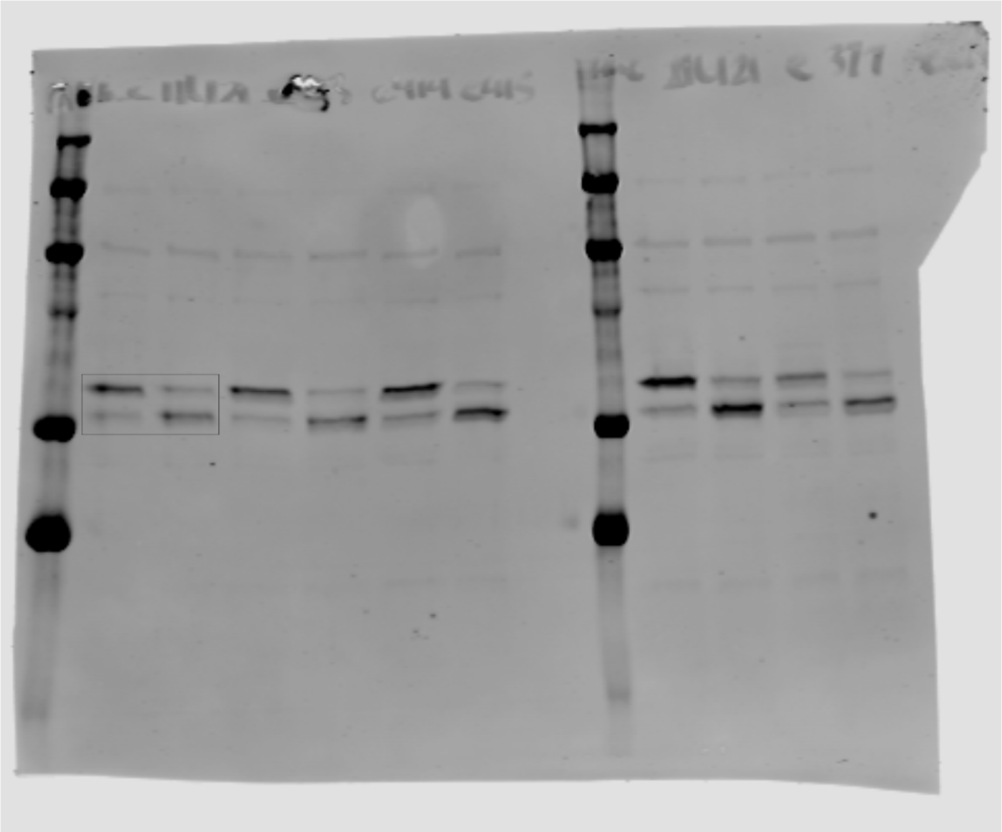

Figure 1D – 4% O_2 – HIF1α

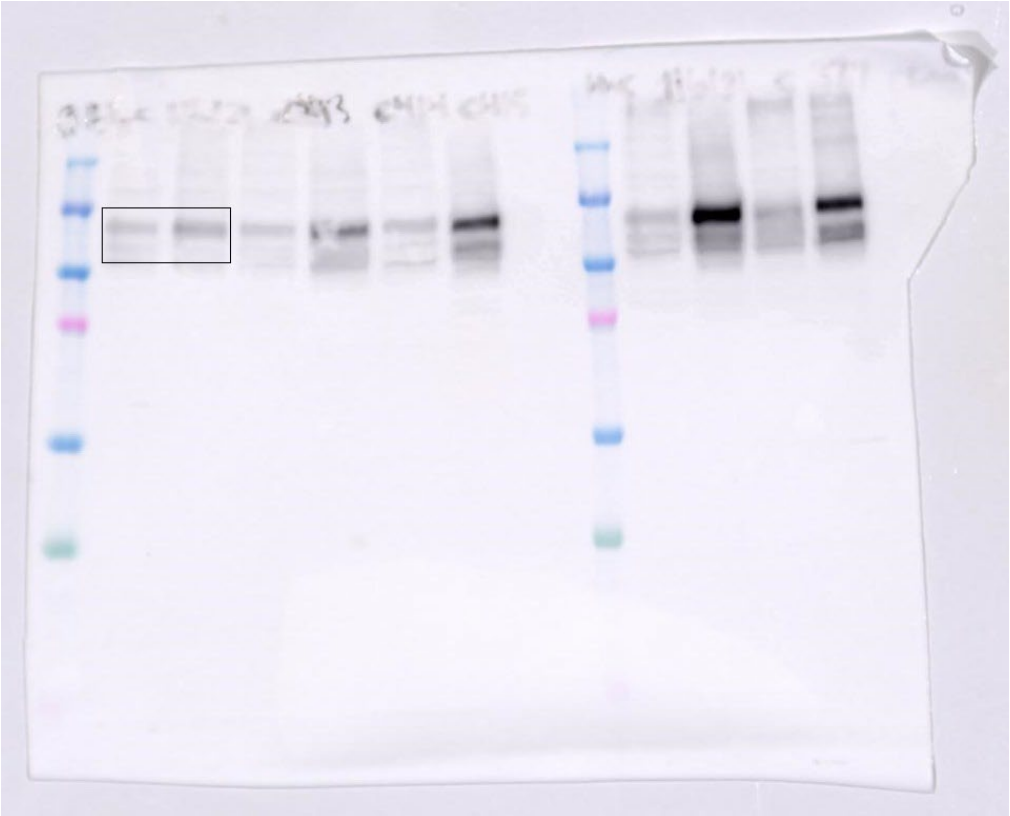

Figure 1D – 4% O_2 – GAPDH

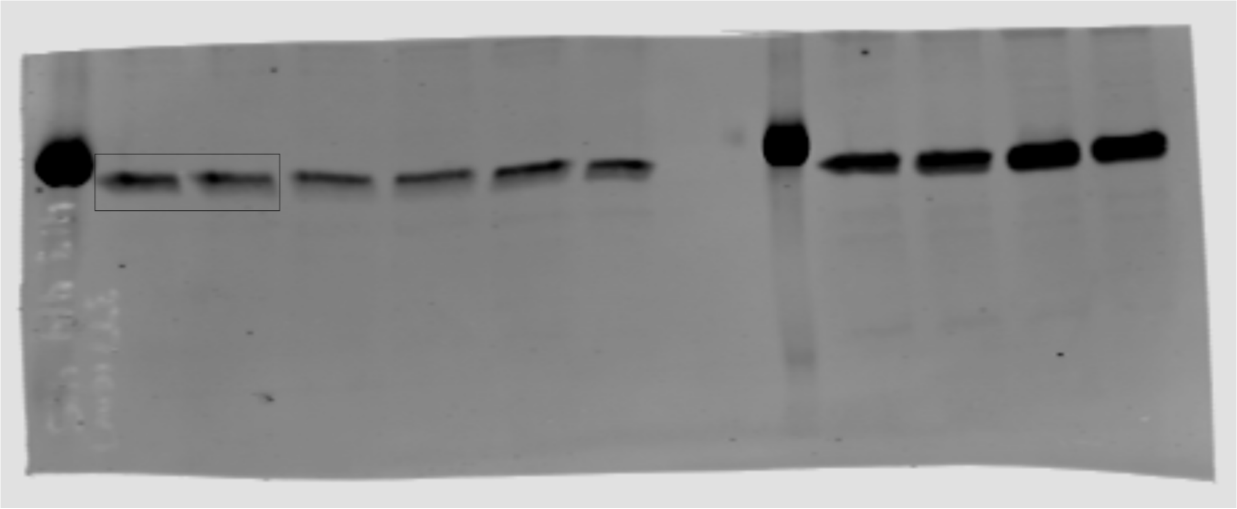

Figure 1D – 5% O_2 – SM

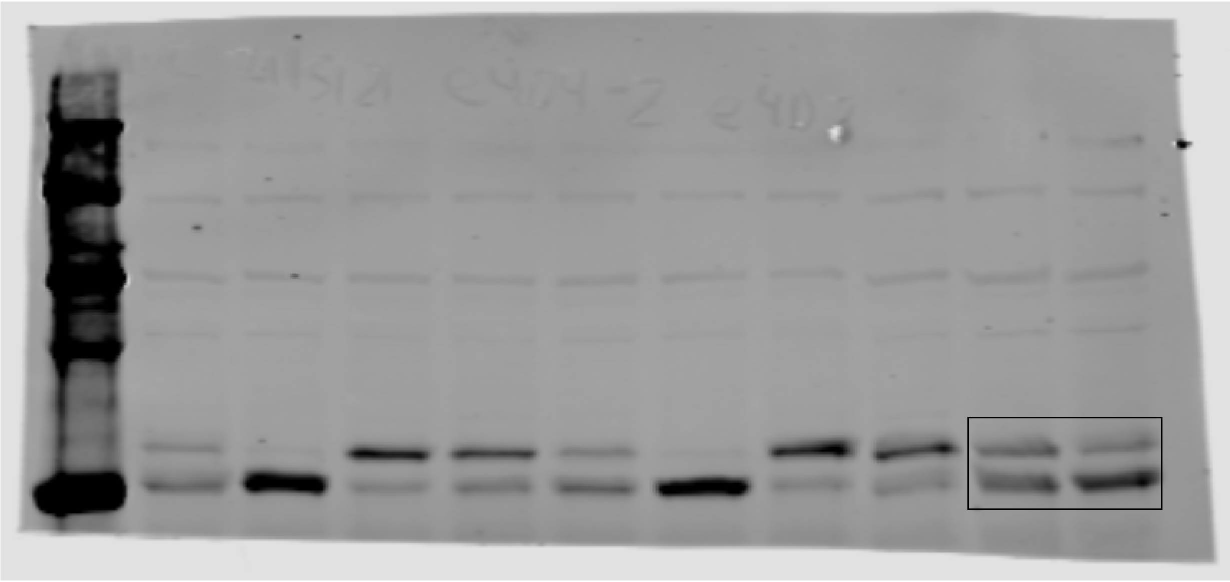

Figure 1D – 5% O_2 – HIF1α

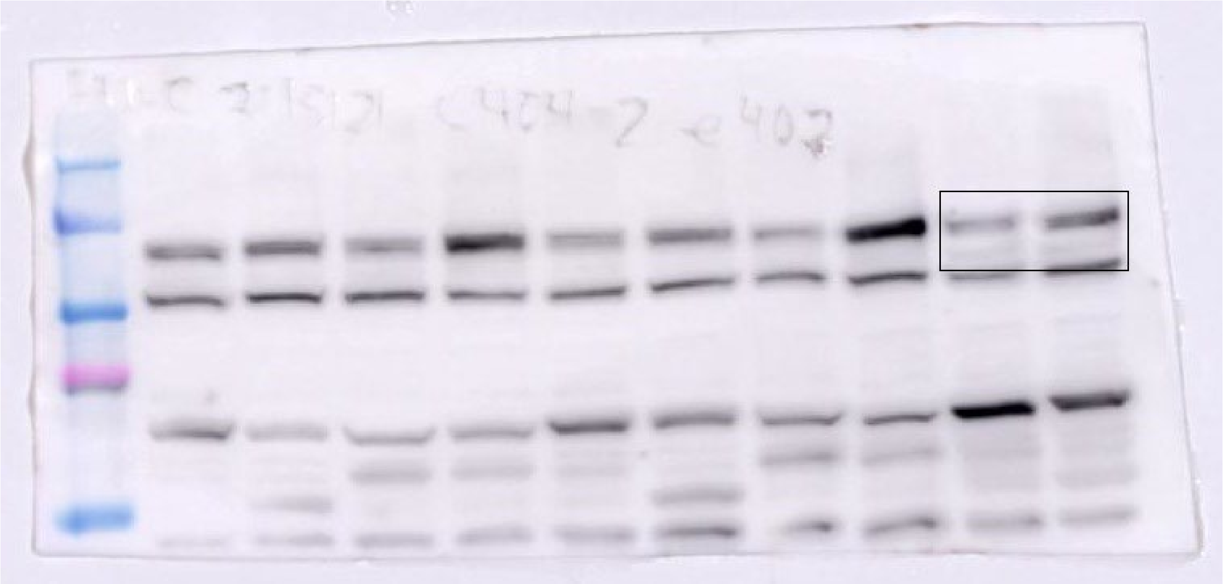

Figure 1D – 5% O_2 – GAPDH

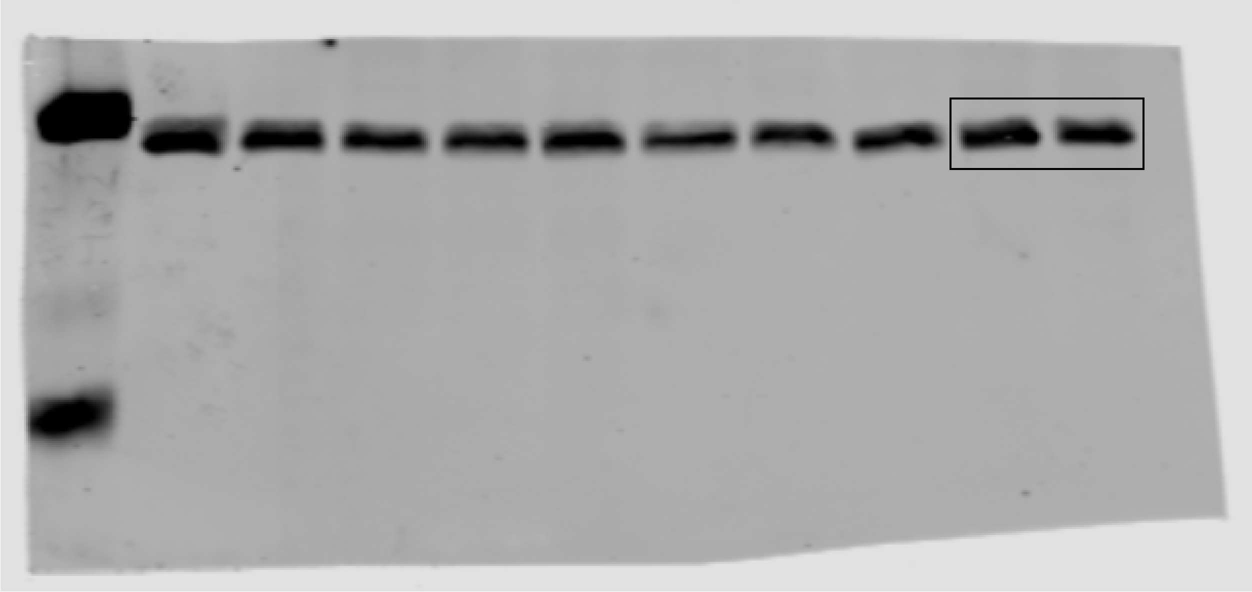

Figure 1D – 10% O_2 – SM

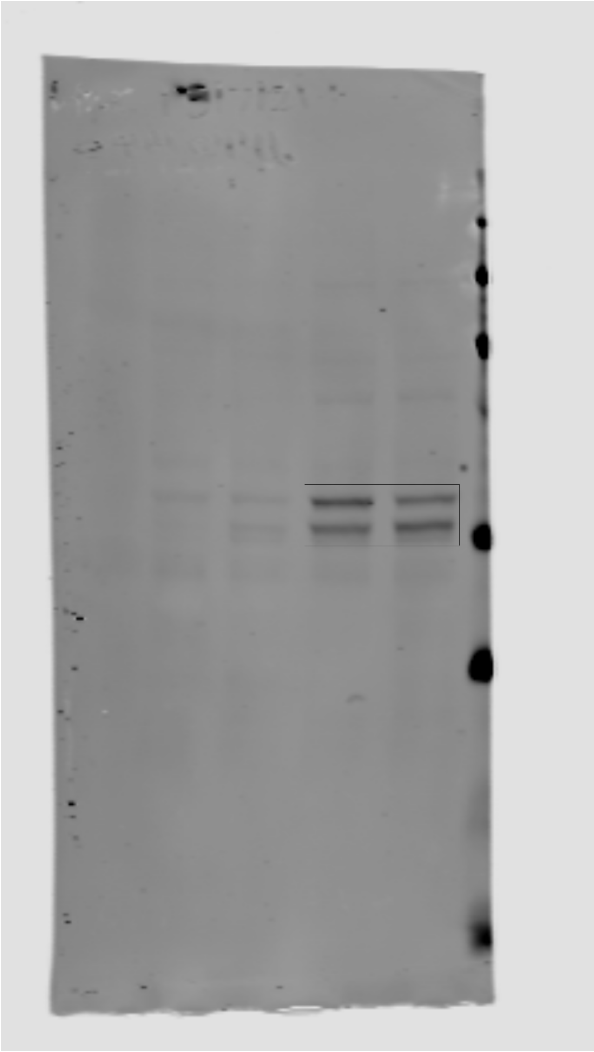

Figure 1D – 10% O_2 – HIF1α

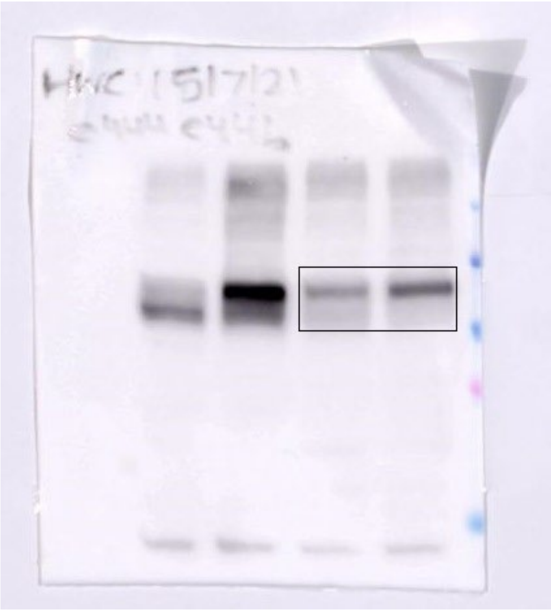

Figure 1D – 10% O_2 – GAPDH

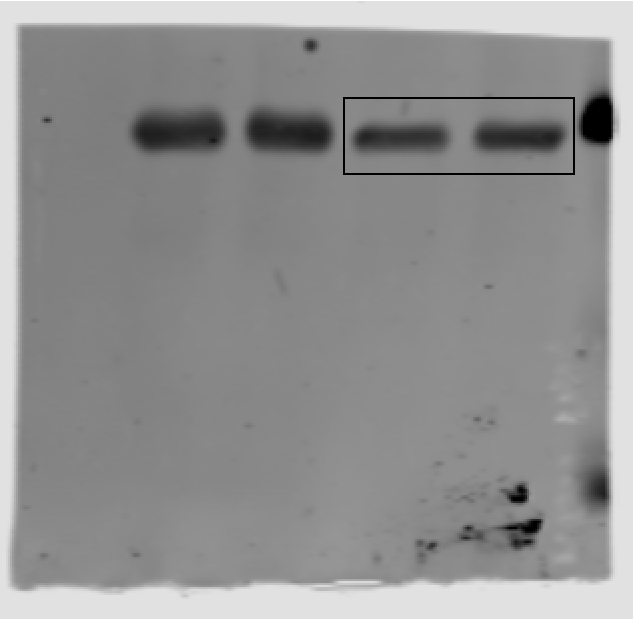

Supplement: Figure 1—source data 1. [file elife-82843-fig1-data1.zip › Figure 1-annotated source data.pdf]
