# Supplementary figures and images for: Hypoxia truncates and constitutively activates the key cholesterol synthesis enzyme squalene monooxygenase (part 1 of 2)
Source: eLife. 2023 Jan 19;12:e82843. doi: 10.7554/eLife.82843 (PMC9851614; doi:10.7554/eLife.82843)

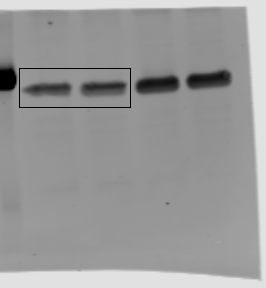

Supplement: Figure 1—source data 1. [file elife-82843-fig1-data1.zip › Annotated/Fig. 1B GAPDH.tif]

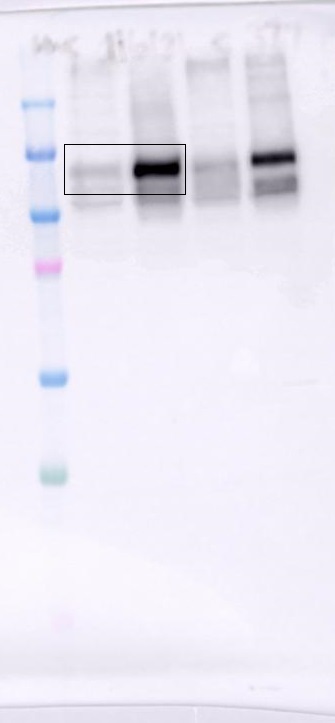

Supplement: Figure 1—source data 1. [file elife-82843-fig1-data1.zip › Annotated/Fig. 1B HIF1a.jpg]

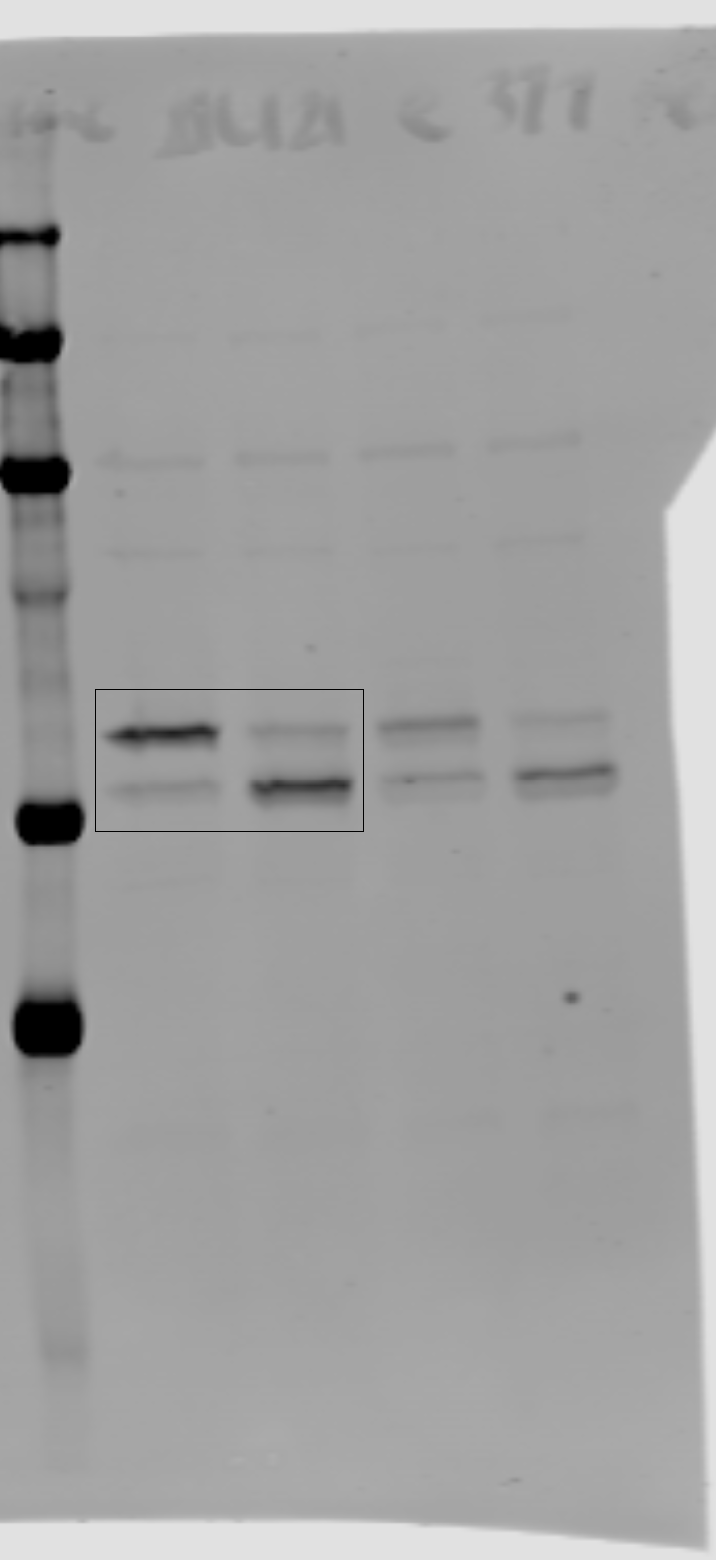

Supplement: Figure 1—source data 1. [file elife-82843-fig1-data1.zip › Annotated/Fig. 1B SM.tif]

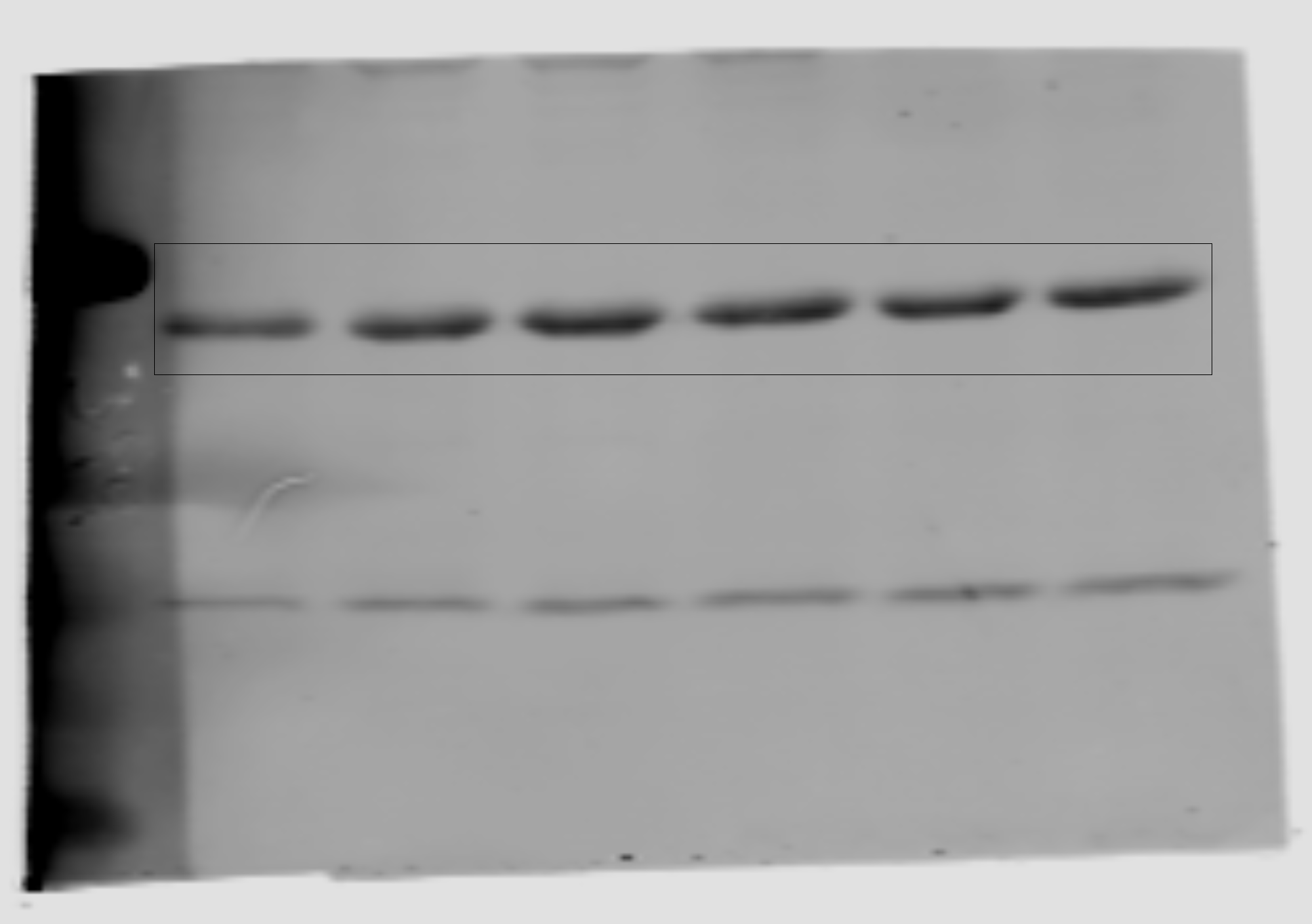

Supplement: Figure 1—source data 1. [file elife-82843-fig1-data1.zip › Annotated/Fig. 1C GAPDH.tif]

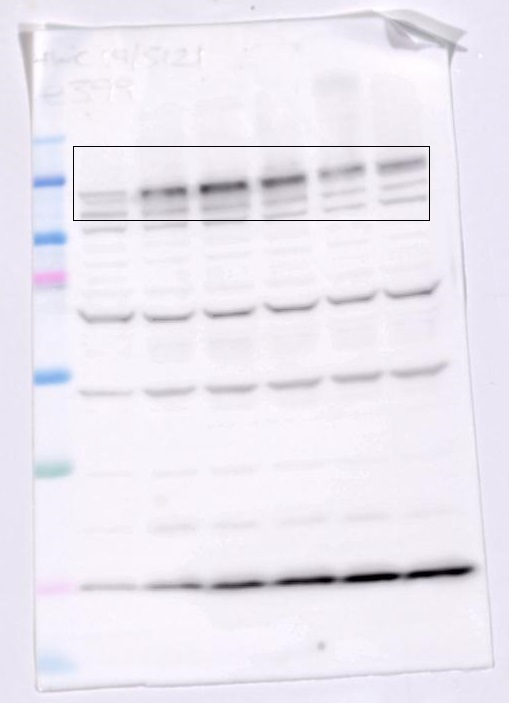

Supplement: Figure 1—source data 1. [file elife-82843-fig1-data1.zip › Annotated/Fig. 1C HIF1a.jpg]

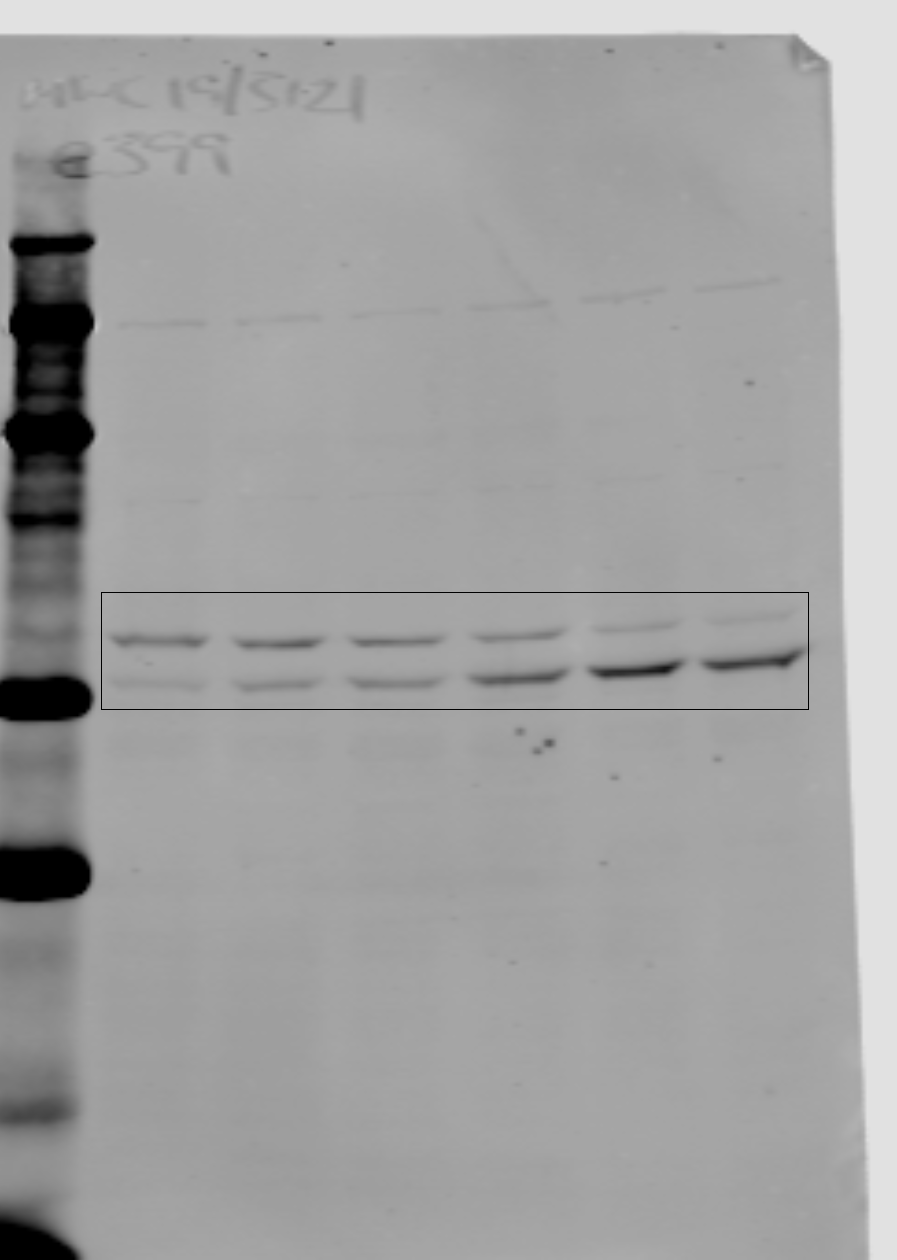

Supplement: Figure 1—source data 1. [file elife-82843-fig1-data1.zip › Annotated/Fig. 1C SM.tif]

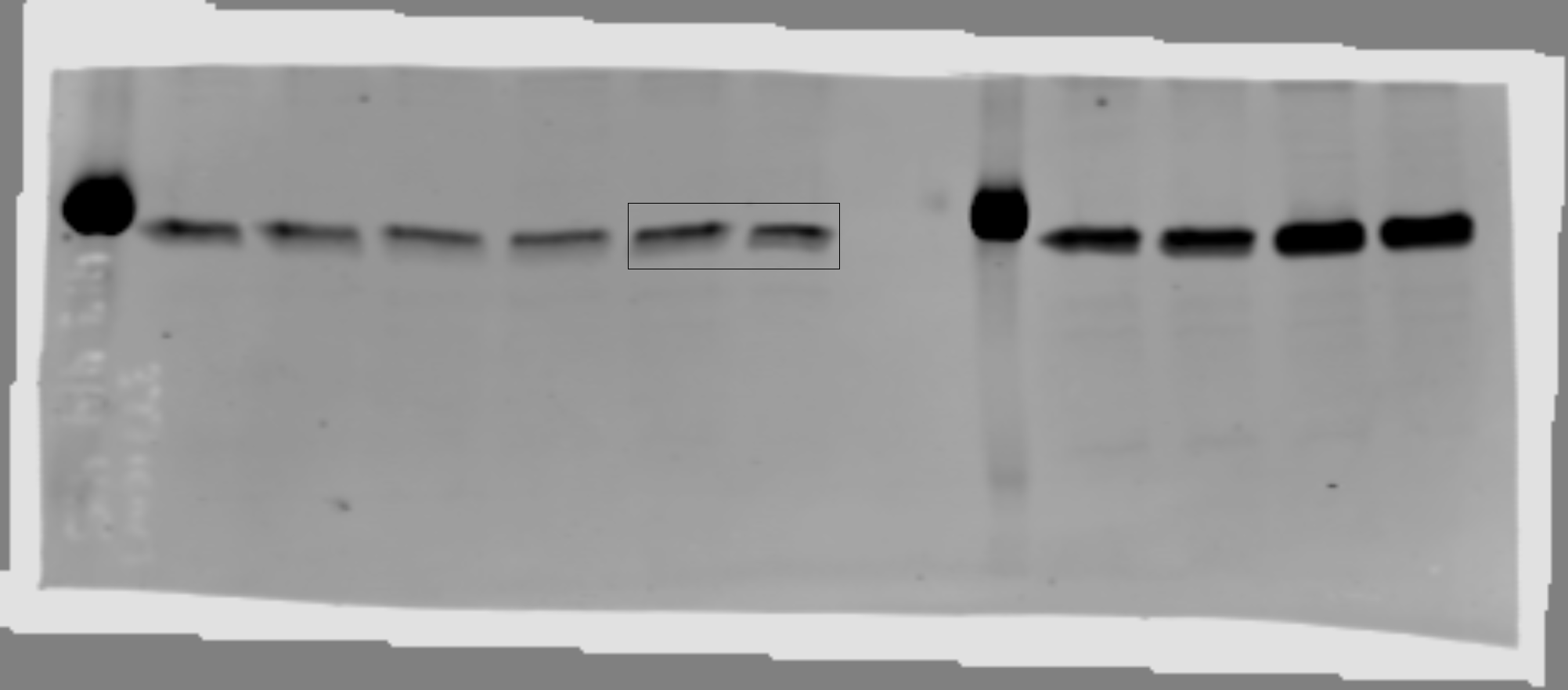

Supplement: Figure 1—source data 1. [file elife-82843-fig1-data1.zip › Annotated/Fig. 1D-0.5% GAPDH.tif]

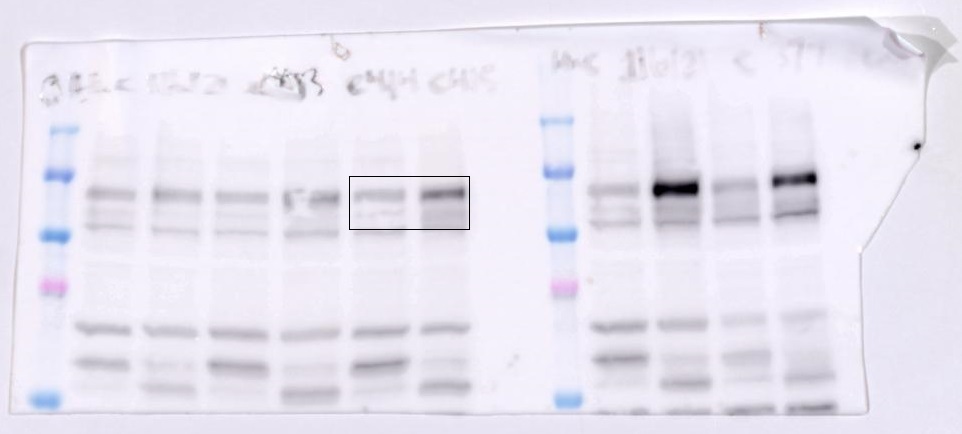

Supplement: Figure 1—source data 1. [file elife-82843-fig1-data1.zip › Annotated/Fig. 1D-0.5% HIF1a.jpg]

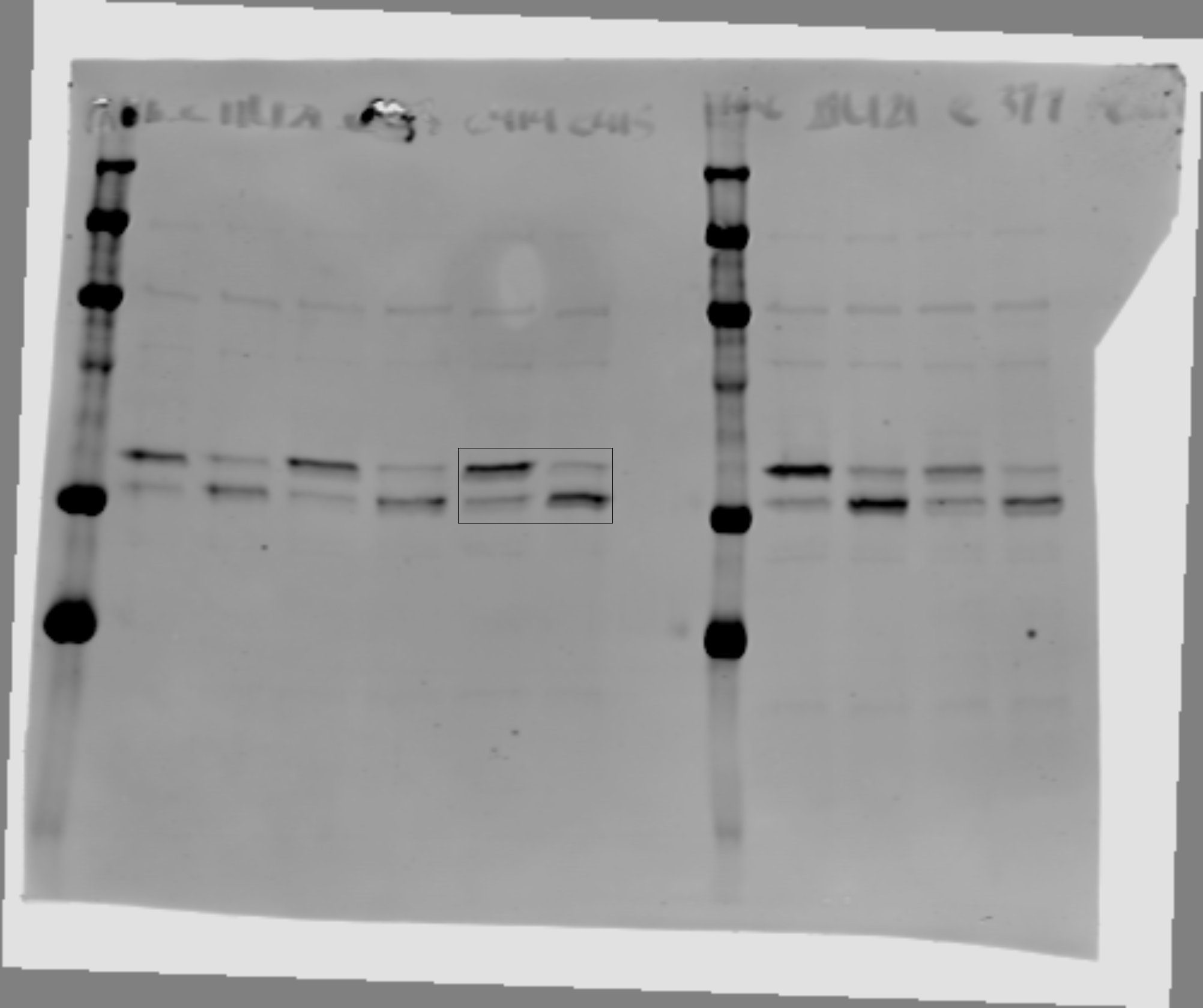

Supplement: Figure 1—source data 1. [file elife-82843-fig1-data1.zip › Annotated/Fig. 1D-0.5% SM.tif]

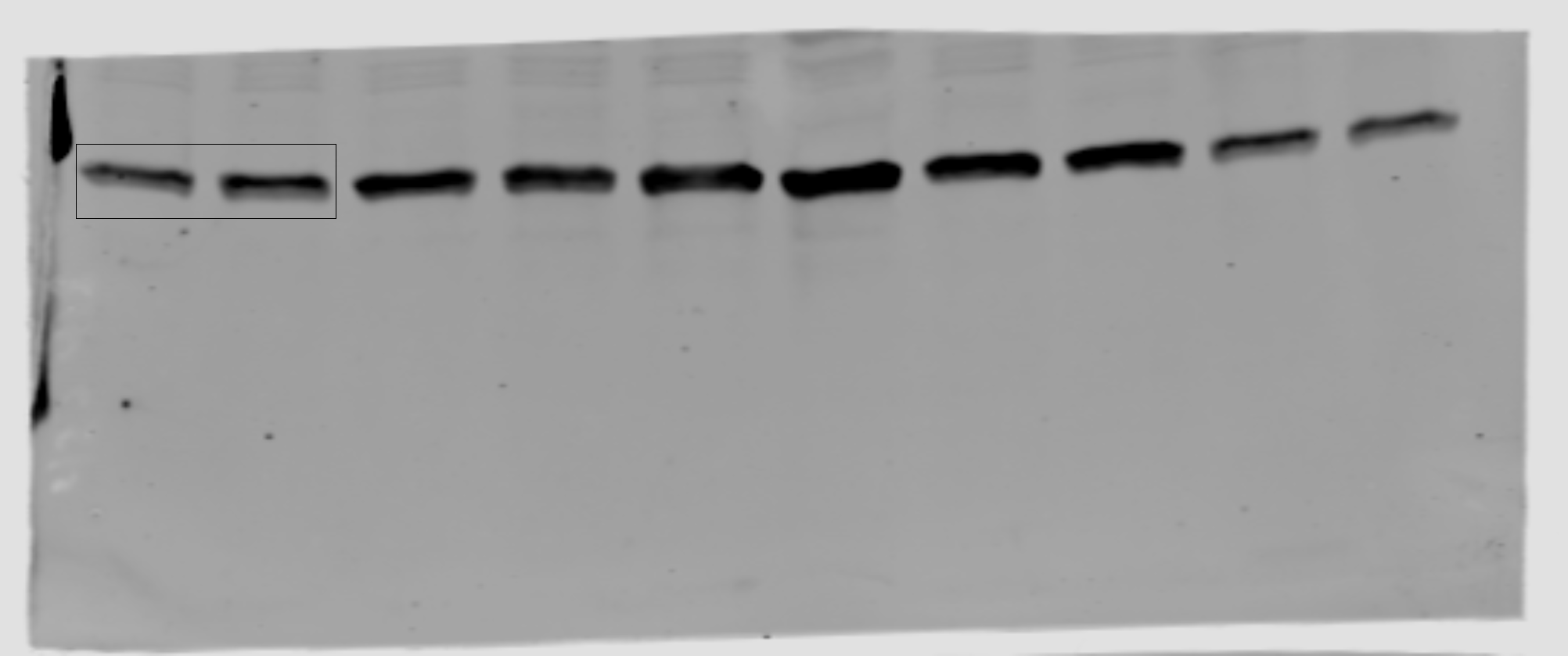

Supplement: Figure 1—source data 1. [file elife-82843-fig1-data1.zip › Annotated/Fig. 1D-1% GAPDH.tif]

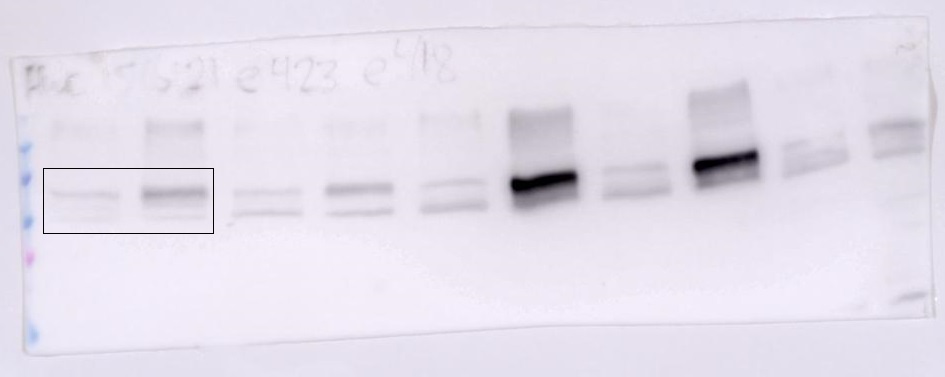

Supplement: Figure 1—source data 1. [file elife-82843-fig1-data1.zip › Annotated/Fig. 1D-1% HIF1a.jpg]

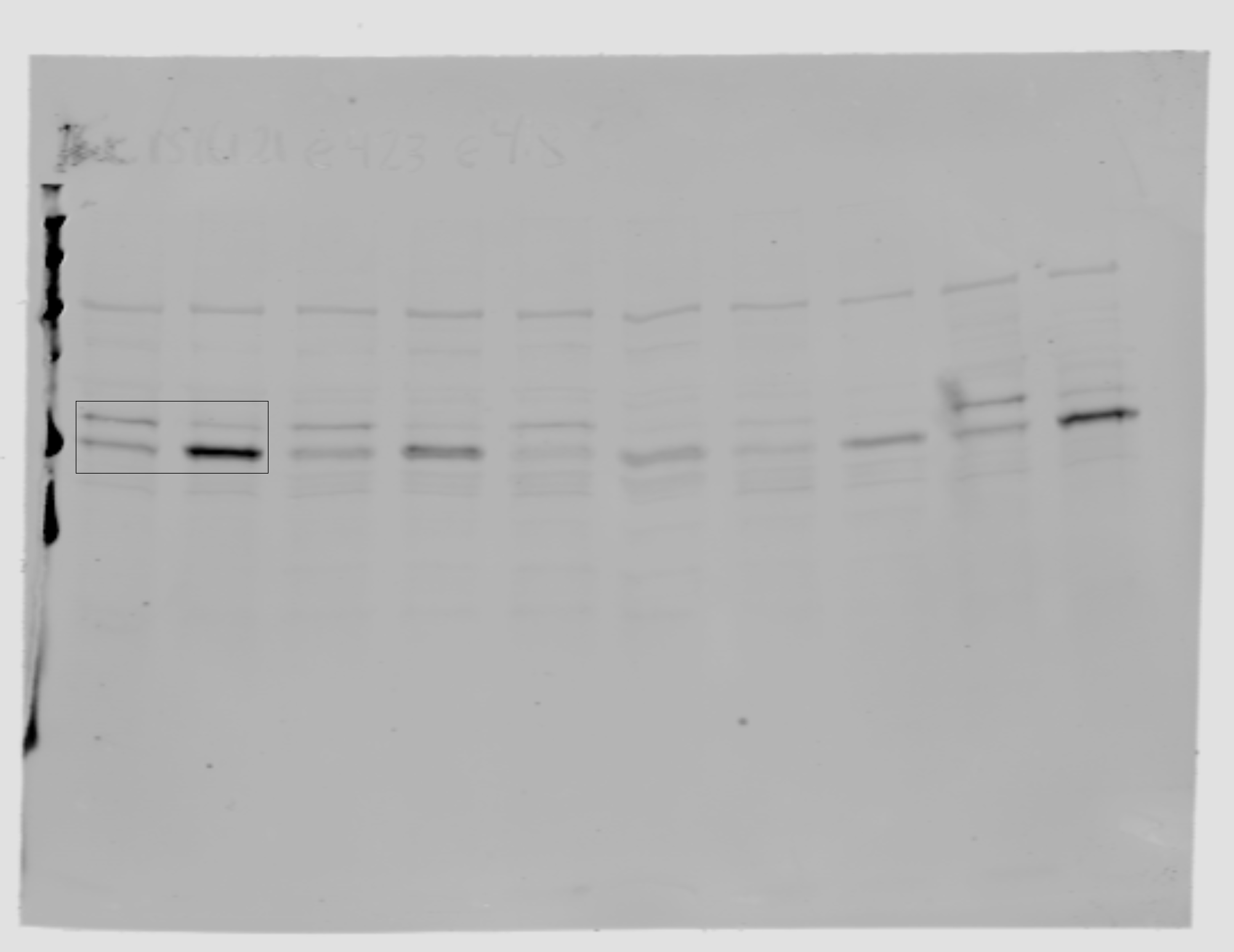

Supplement: Figure 1—source data 1. [file elife-82843-fig1-data1.zip › Annotated/Fig. 1D-1% SM.tif]

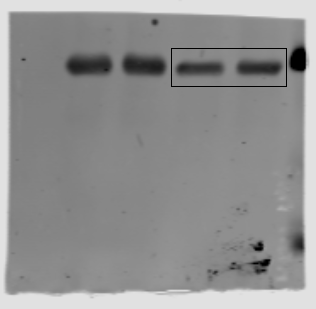

Supplement: Figure 1—source data 1. [file elife-82843-fig1-data1.zip › Annotated/Fig. 1D-10% GAPDH.tif]

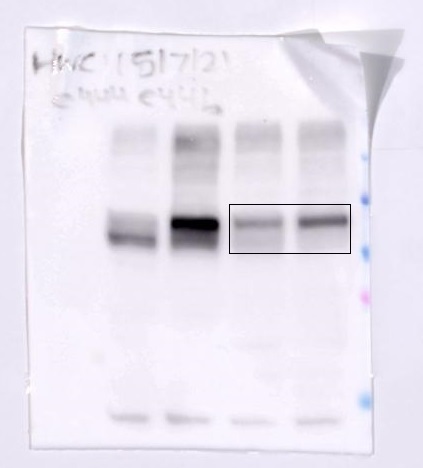

Supplement: Figure 1—source data 1. [file elife-82843-fig1-data1.zip › Annotated/Fig. 1D-10% HIF1a.jpg]

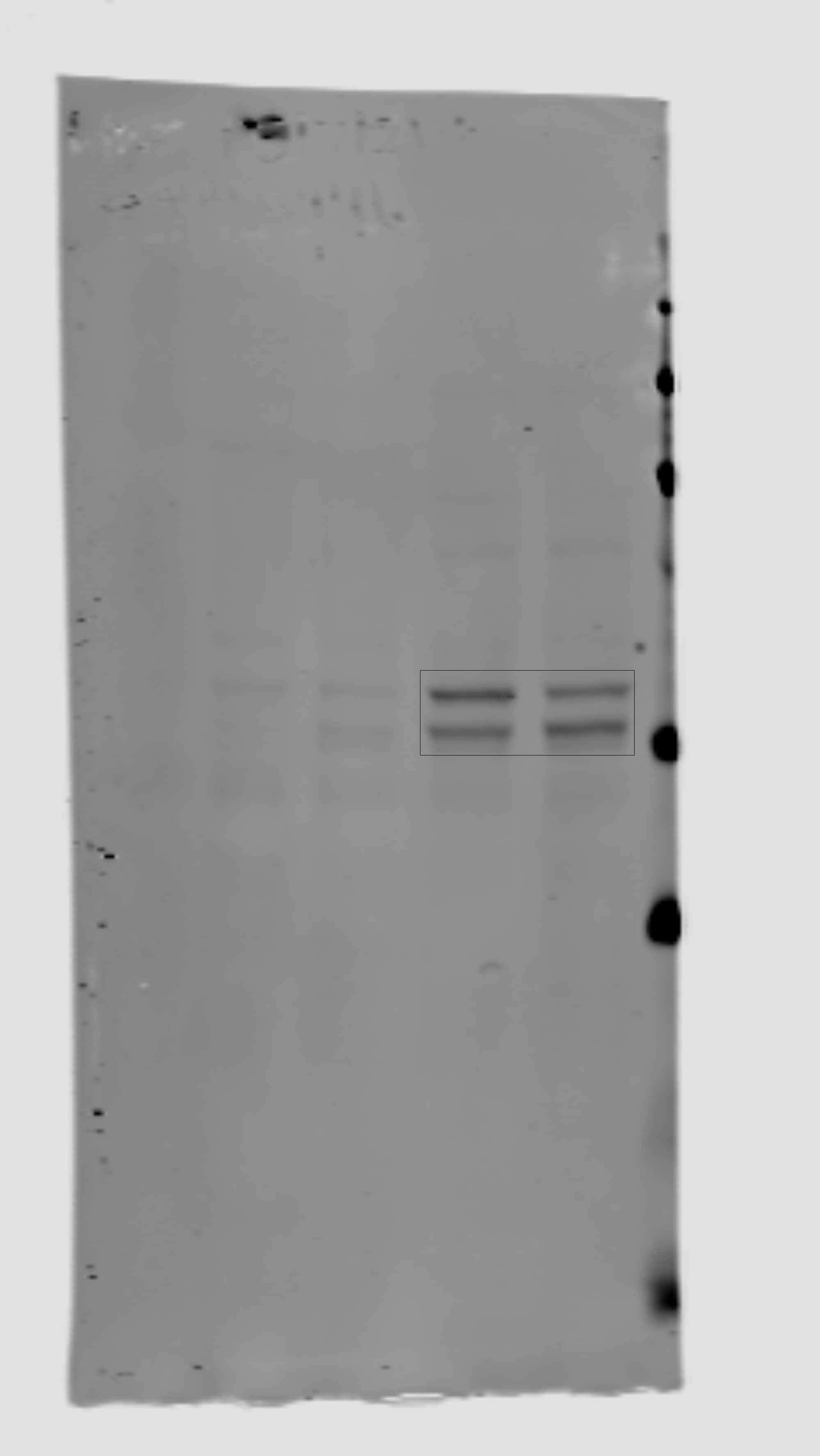

Supplement: Figure 1—source data 1. [file elife-82843-fig1-data1.zip › Annotated/Fig. 1D-10% SM.tif]

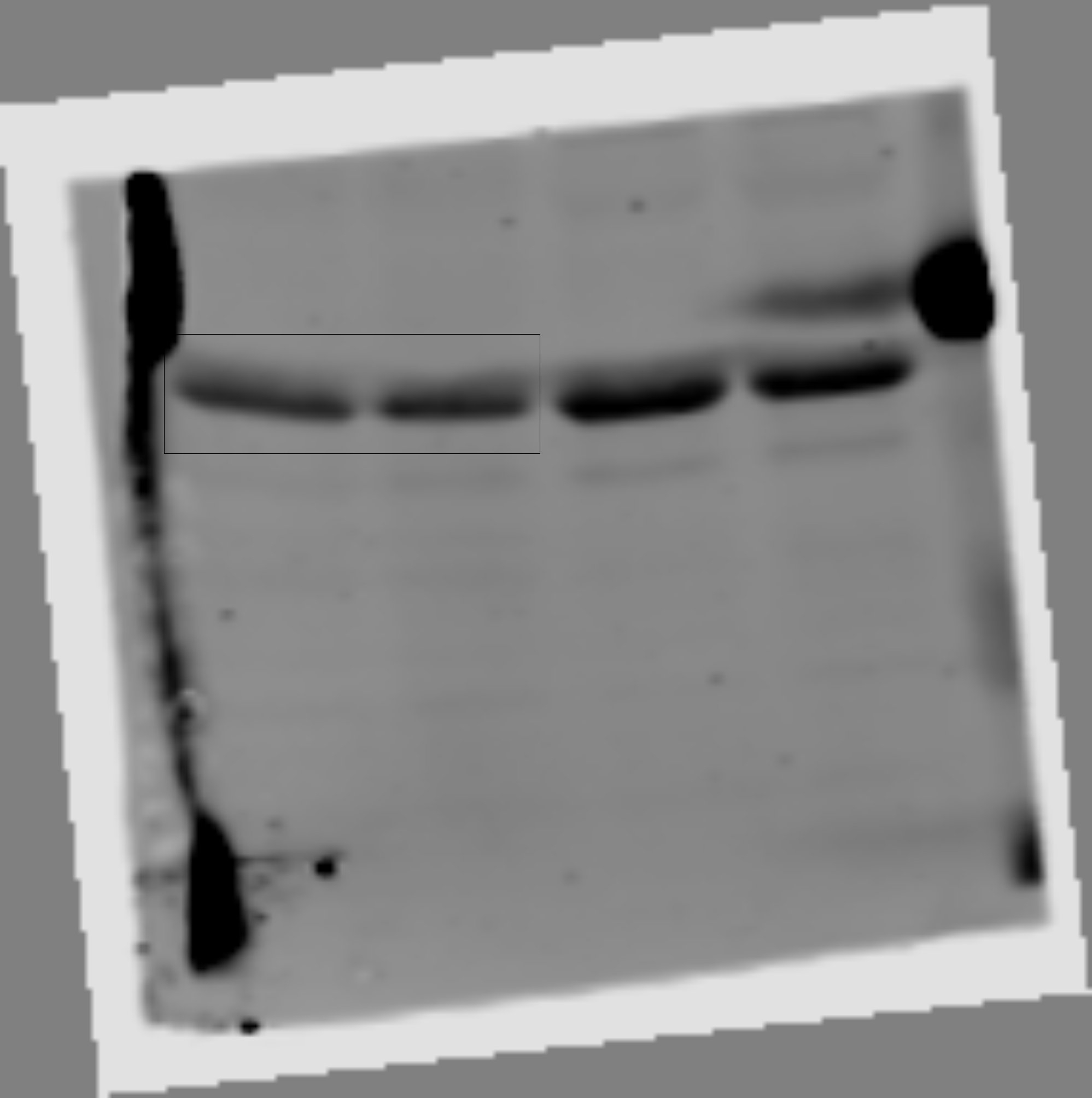

Supplement: Figure 1—source data 1. [file elife-82843-fig1-data1.zip › Annotated/Fig. 1D-2% GAPDH.tif]

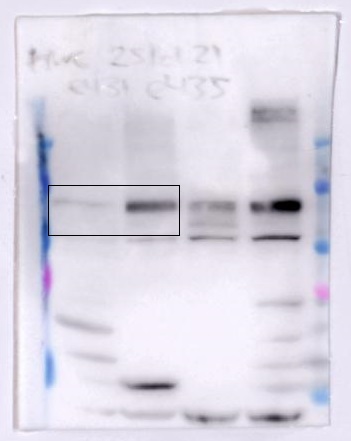

Supplement: Figure 1—source data 1. [file elife-82843-fig1-data1.zip › Annotated/Fig. 1D-2% HIF1a.jpg]

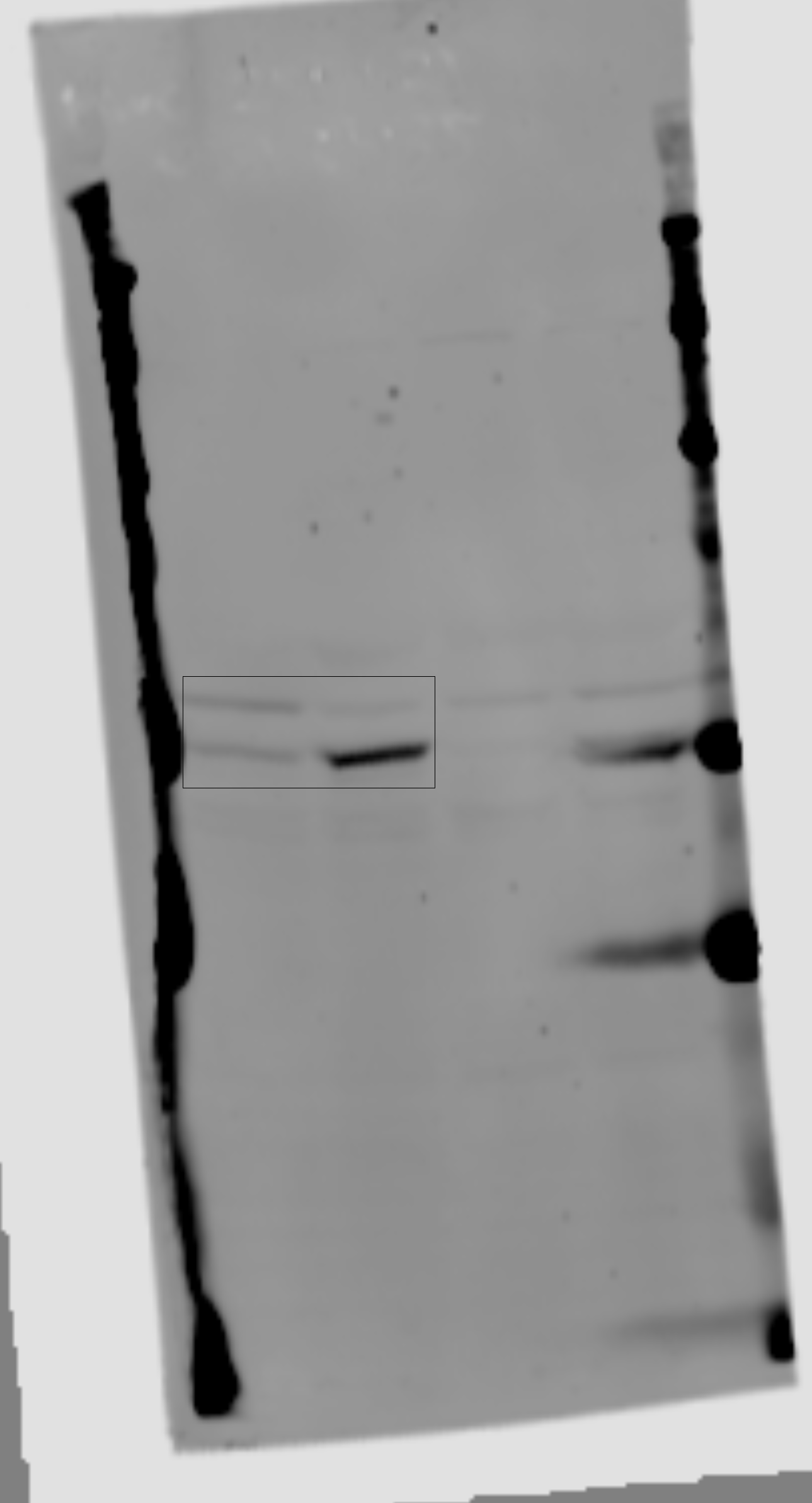

Supplement: Figure 1—source data 1. [file elife-82843-fig1-data1.zip › Annotated/Fig. 1D-2% SM.tif]

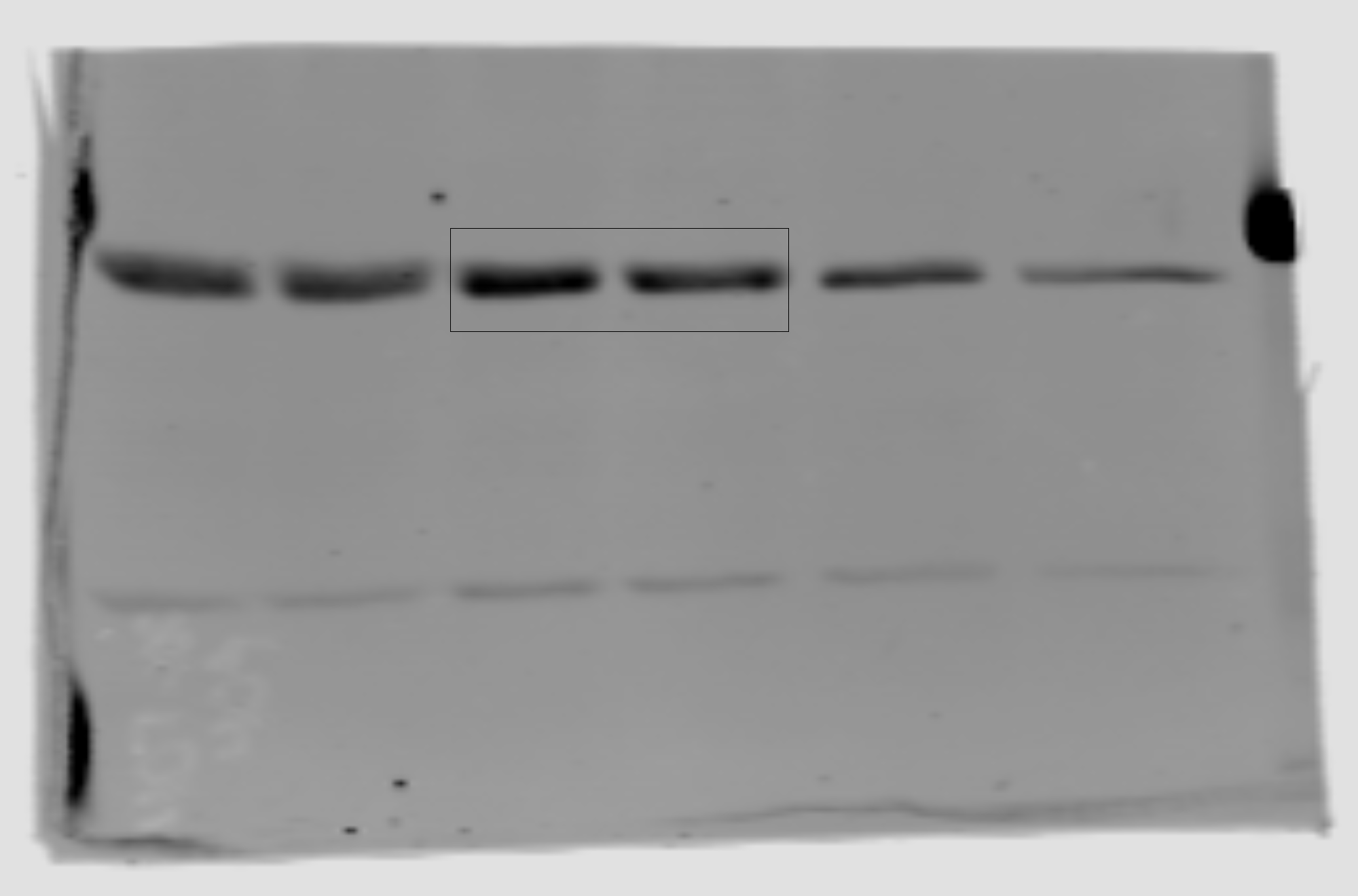

Supplement: Figure 1—source data 1. [file elife-82843-fig1-data1.zip › Annotated/Fig. 1D-3% GAPDH.tif]

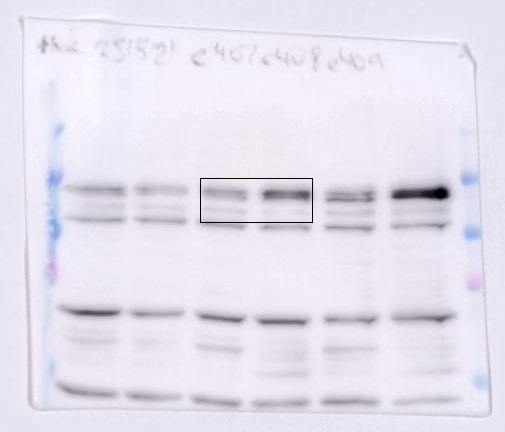

Supplement: Figure 1—source data 1. [file elife-82843-fig1-data1.zip › Annotated/Fig. 1D-3% HIF1a.jpg]

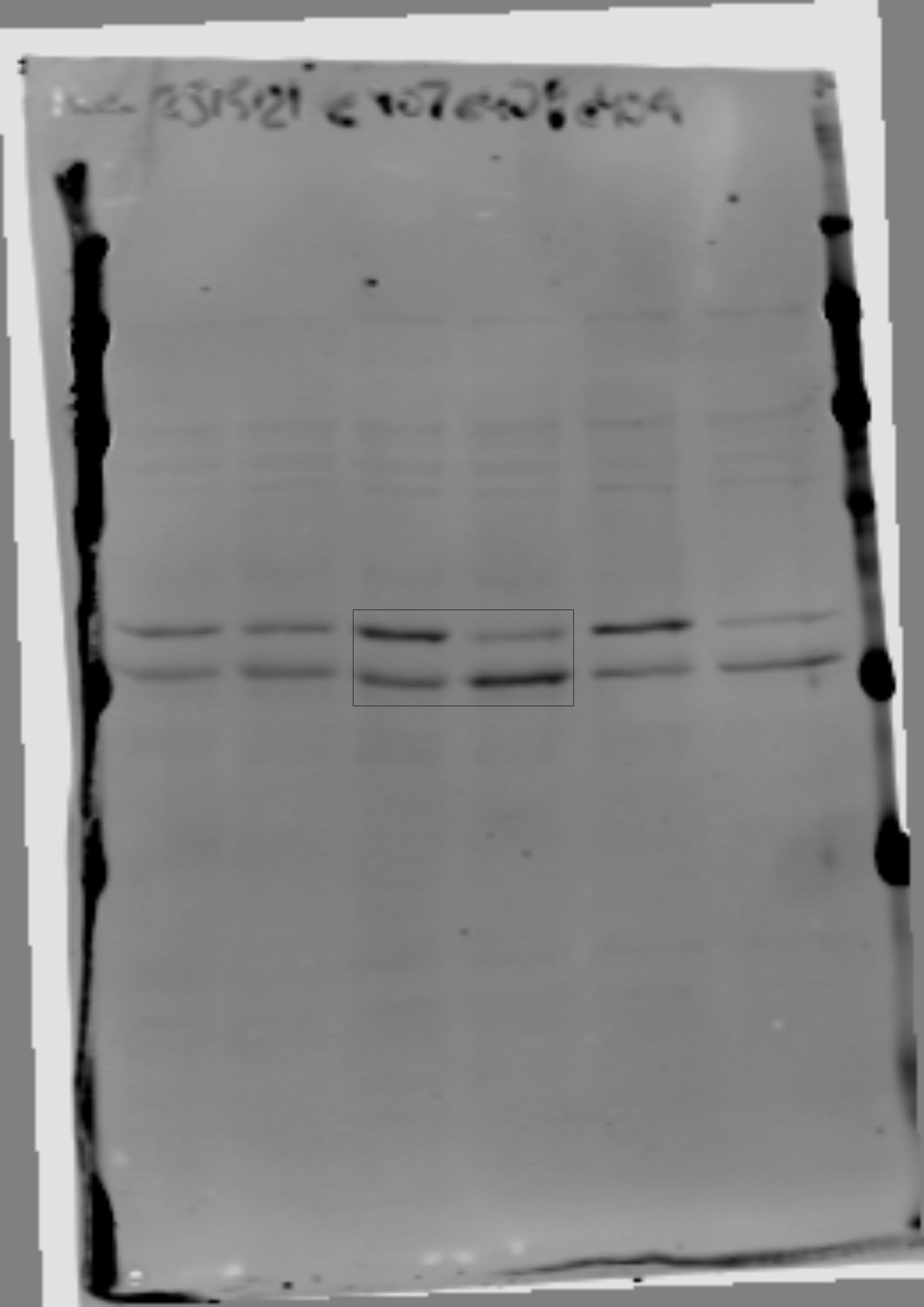

Supplement: Figure 1—source data 1. [file elife-82843-fig1-data1.zip › Annotated/Fig. 1D-3% SM.tif]

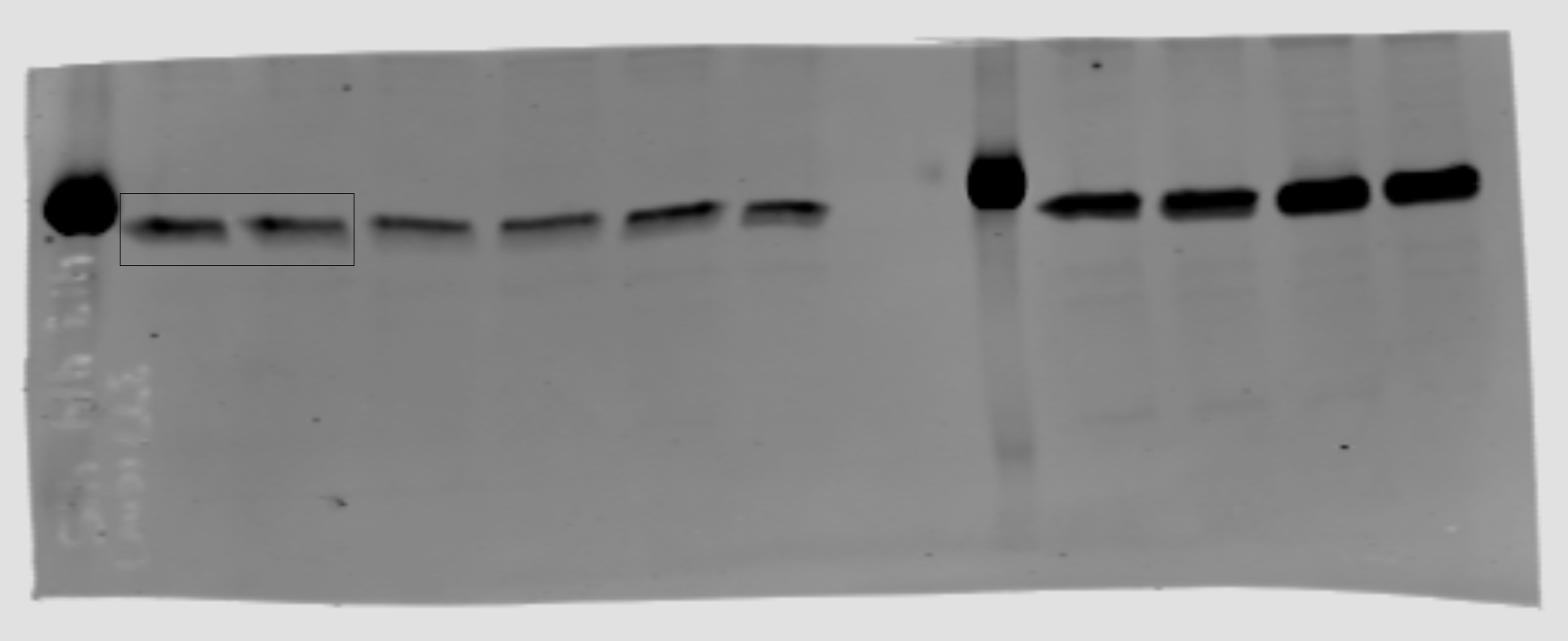

Supplement: Figure 1—source data 1. [file elife-82843-fig1-data1.zip › Annotated/Fig. 1D-4% GAPDH.tif]

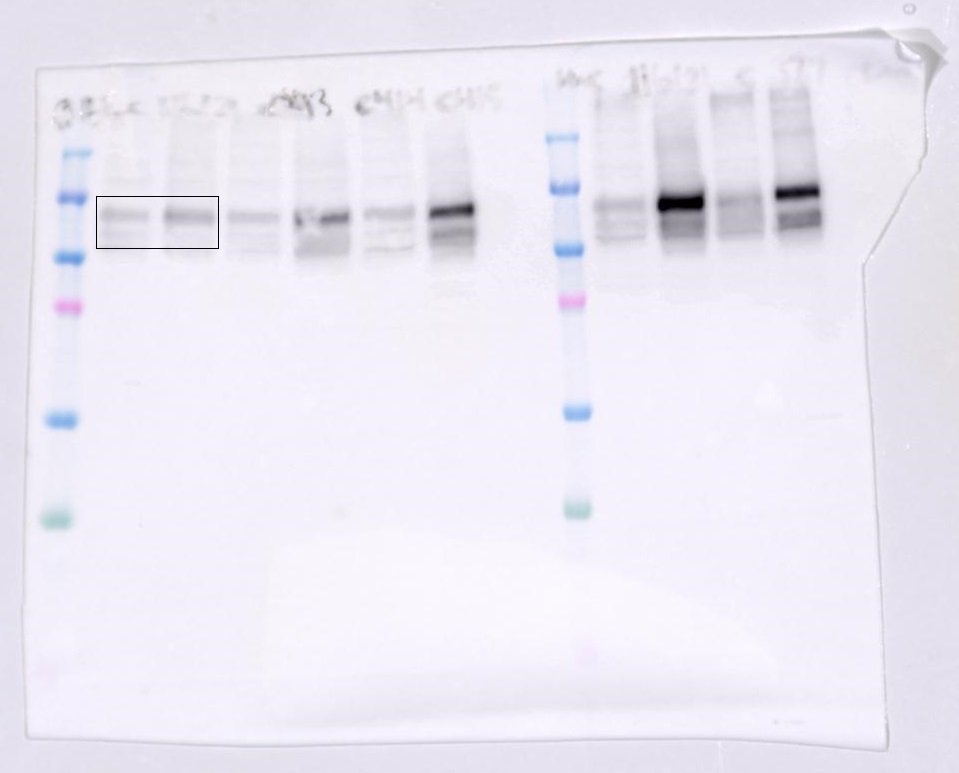

Supplement: Figure 1—source data 1. [file elife-82843-fig1-data1.zip › Annotated/Fig. 1D-4% HIF1a.jpg]

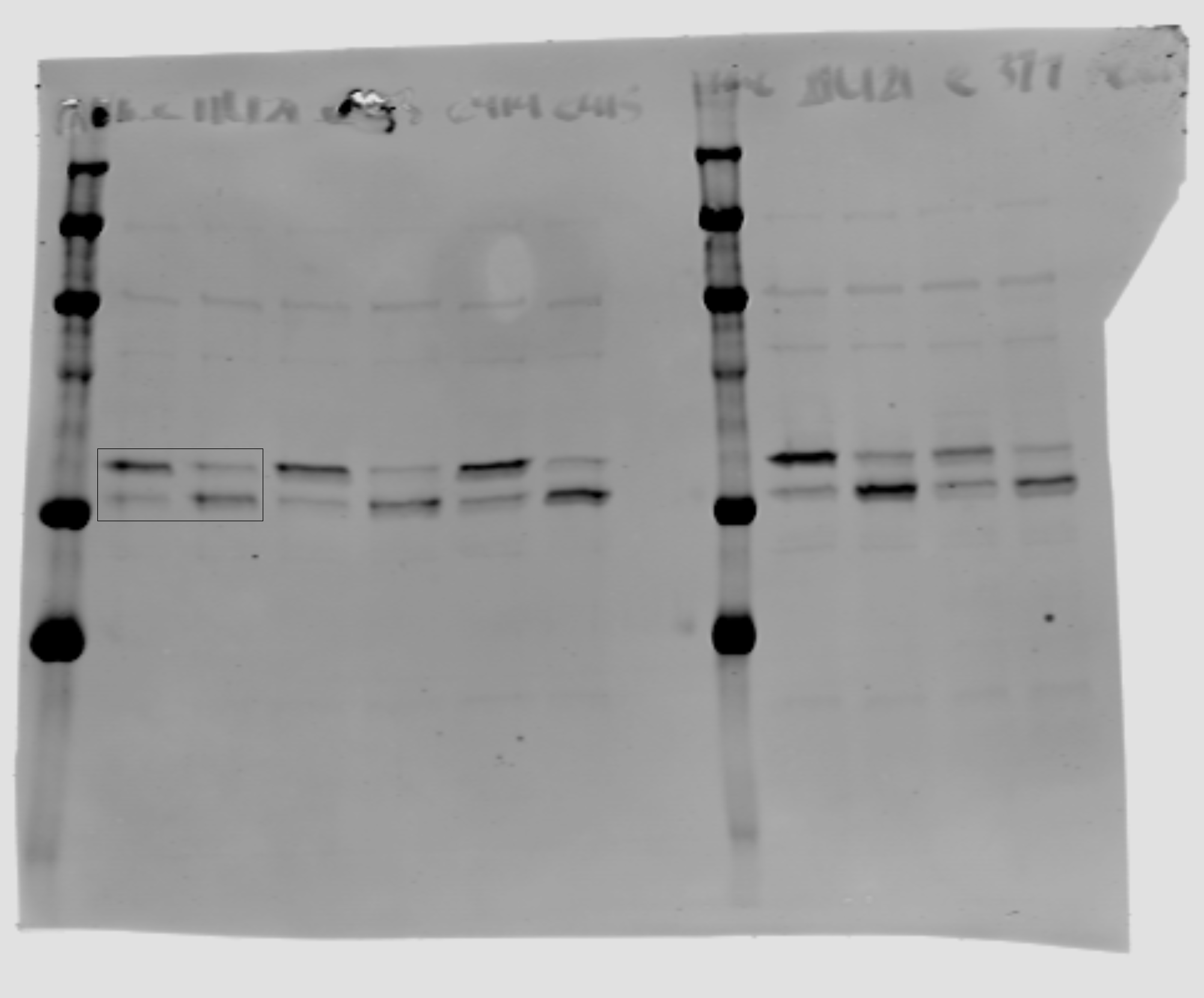

Supplement: Figure 1—source data 1. [file elife-82843-fig1-data1.zip › Annotated/Fig. 1D-4% SM.tif]

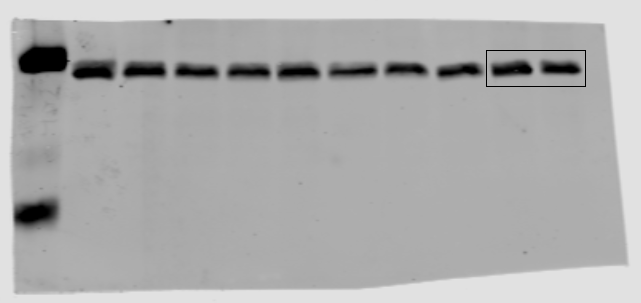

Supplement: Figure 1—source data 1. [file elife-82843-fig1-data1.zip › Annotated/Fig. 1D-5% GAPDH.tif]

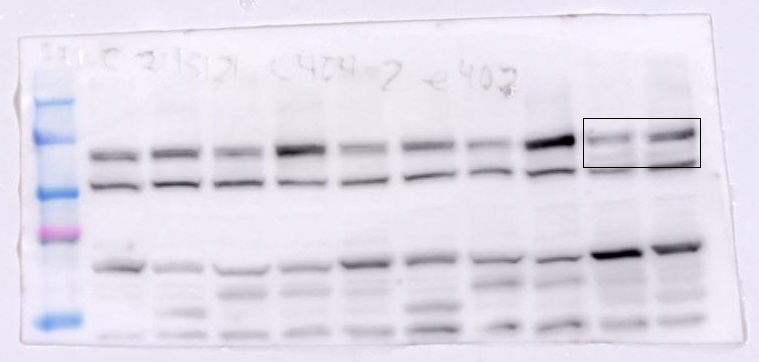

Supplement: Figure 1—source data 1. [file elife-82843-fig1-data1.zip › Annotated/Fig. 1D-5% HIF1a.jpg]

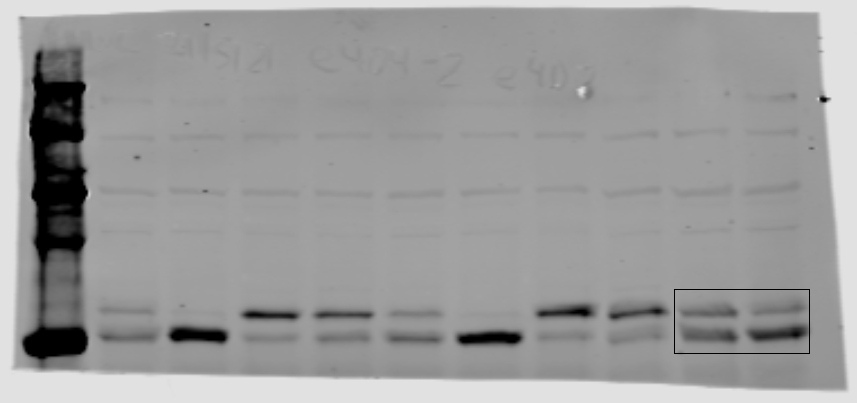

Supplement: Figure 1—source data 1. [file elife-82843-fig1-data1.zip › Annotated/Fig. 1D-5% SM.tif]

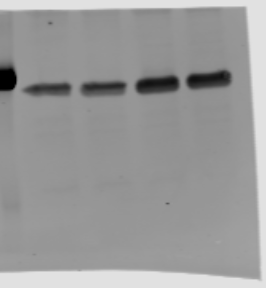

Supplement: Figure 1—source data 1. [file elife-82843-fig1-data1.zip › Fig. 1B GAPDH.tif]

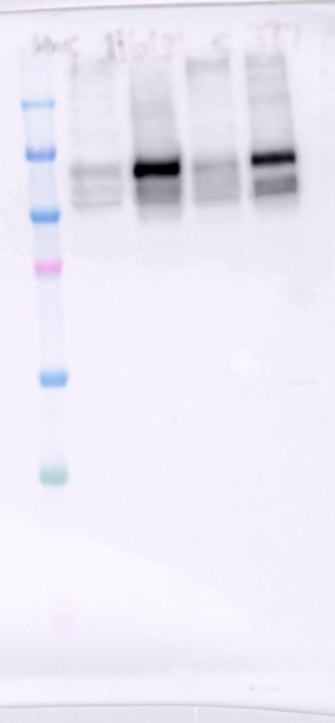

Supplement: Figure 1—source data 1. [file elife-82843-fig1-data1.zip › Fig. 1B HIF1a.jpg]

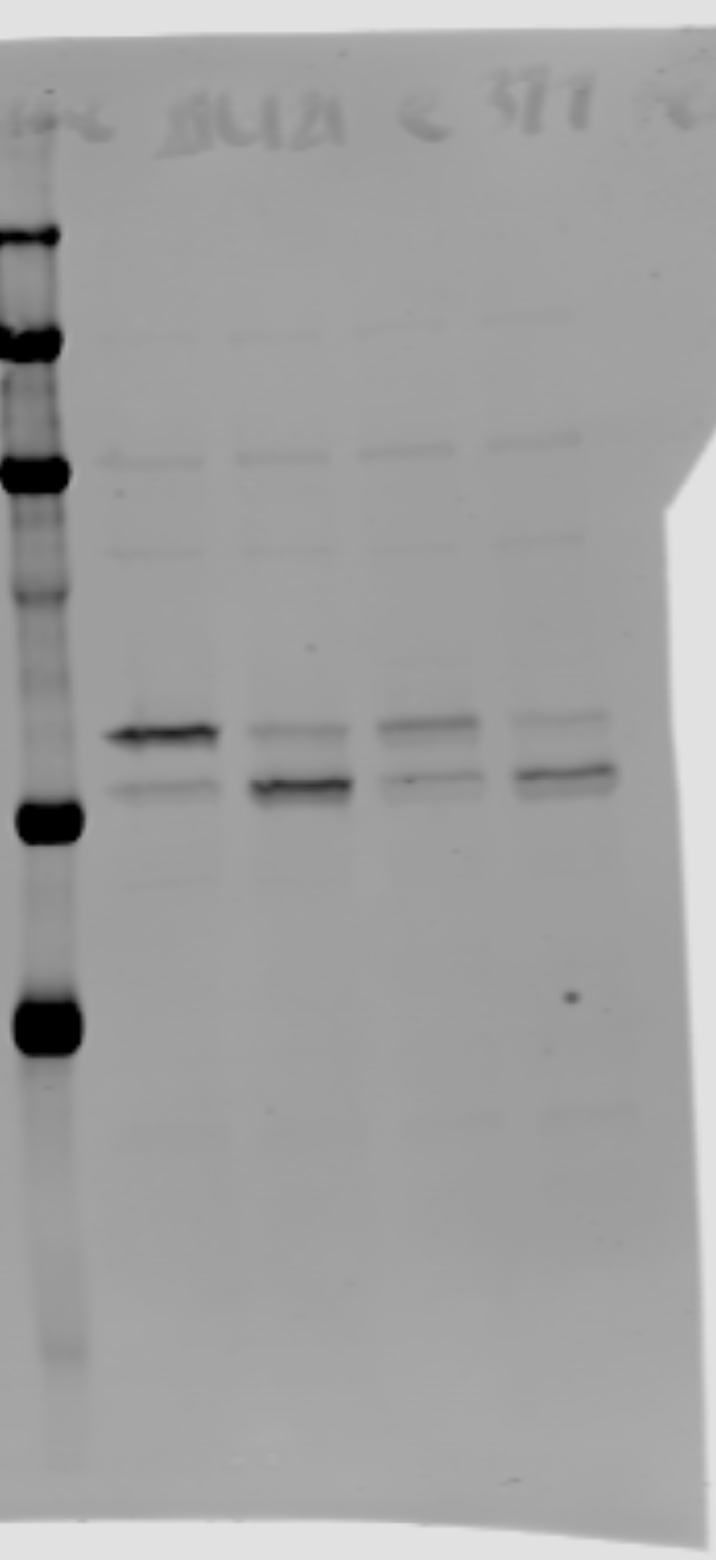

Supplement: Figure 1—source data 1. [file elife-82843-fig1-data1.zip › Fig. 1B SM.tif]

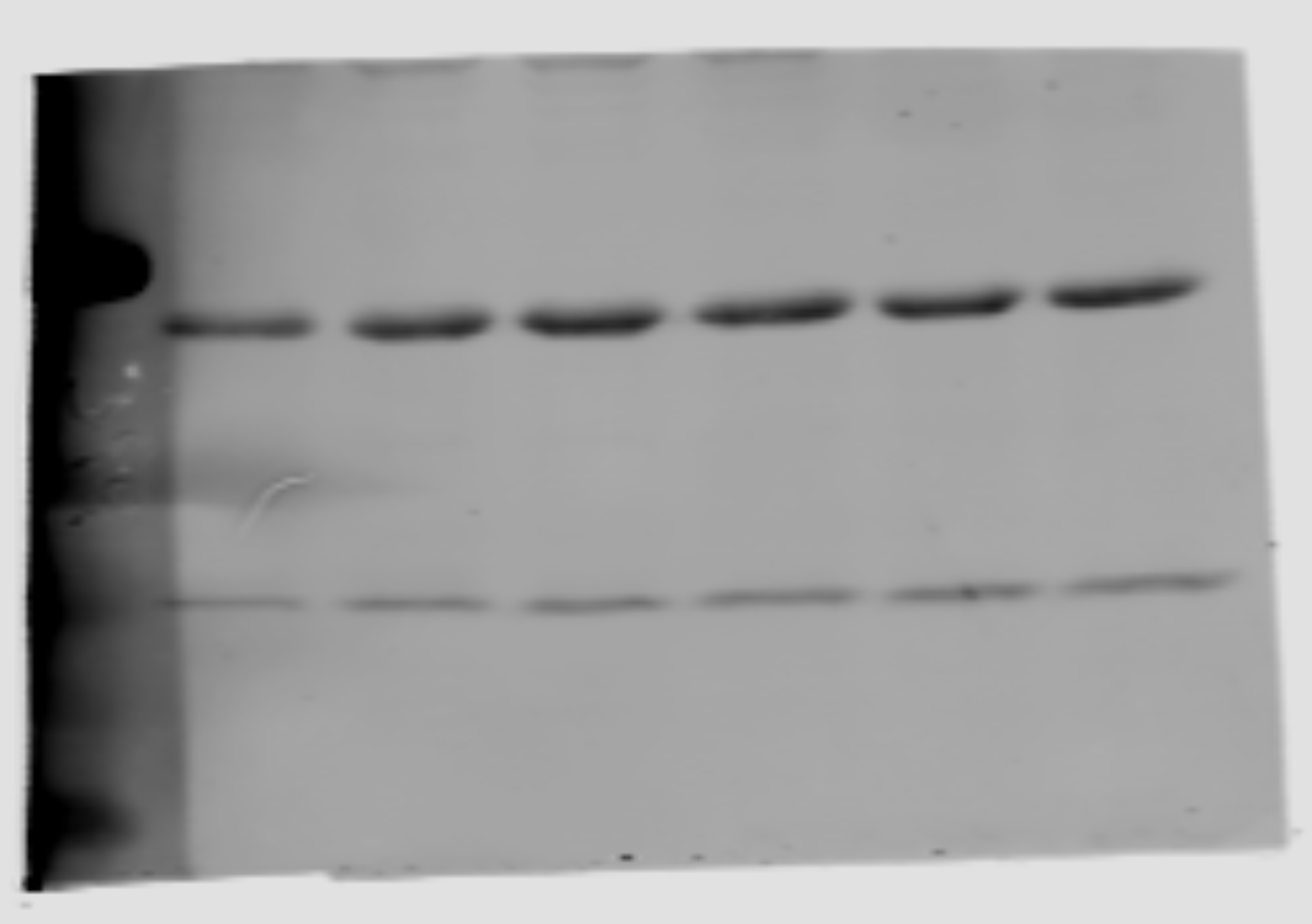

Supplement: Figure 1—source data 1. [file elife-82843-fig1-data1.zip › Fig. 1C GAPDH.tif]

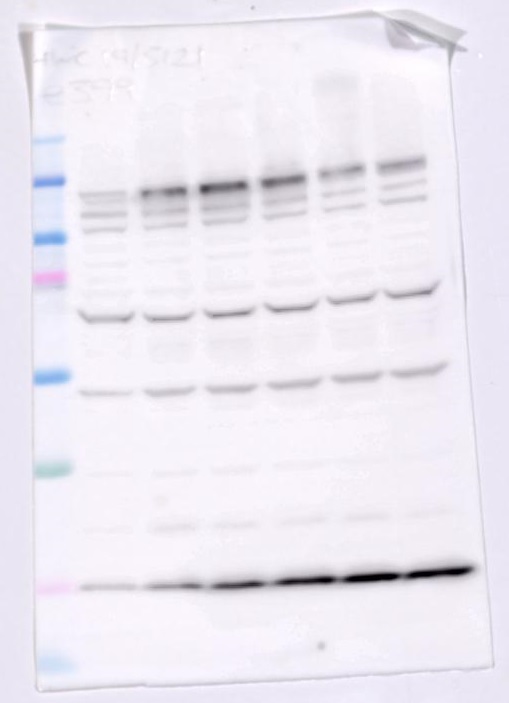

Supplement: Figure 1—source data 1. [file elife-82843-fig1-data1.zip › Fig. 1C HIF1a.jpg]

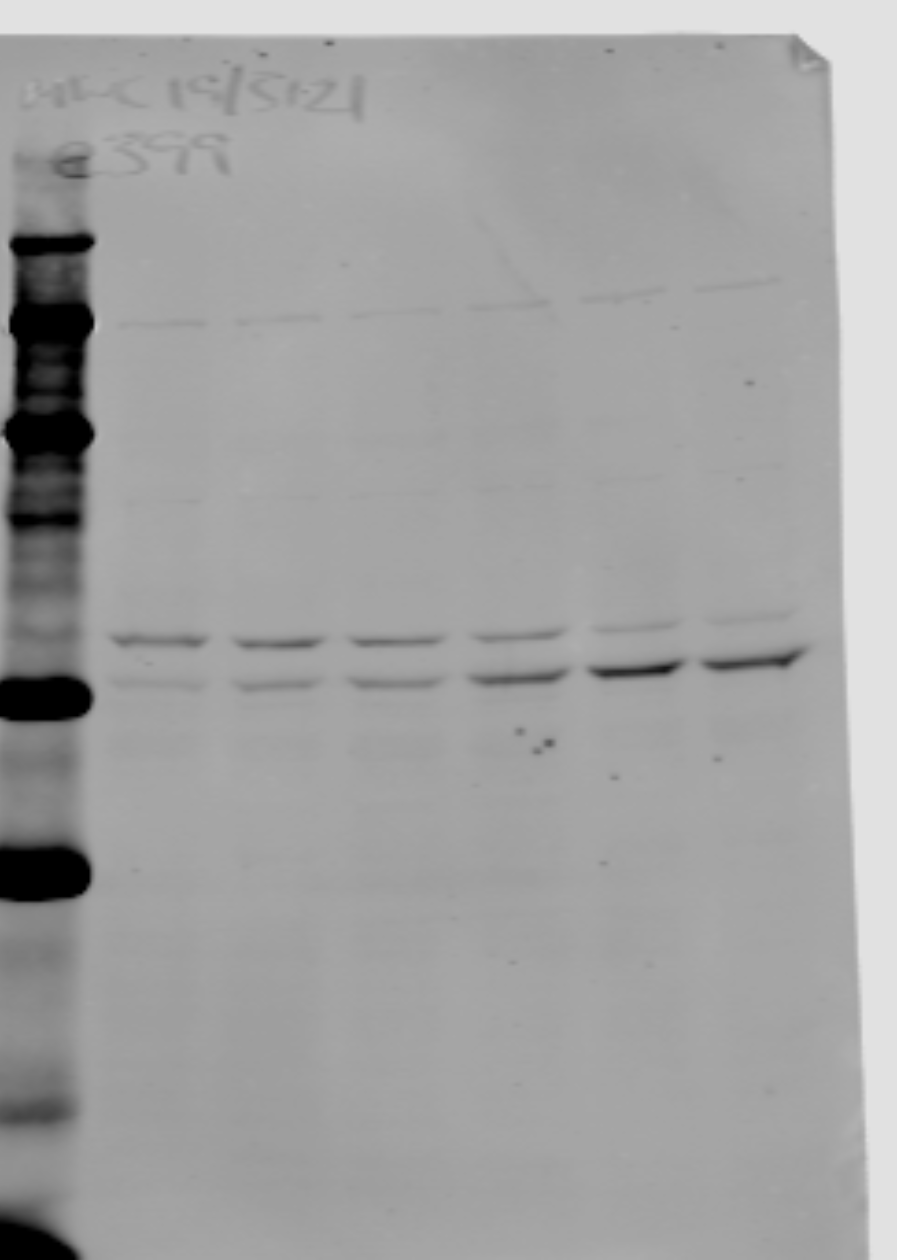

Supplement: Figure 1—source data 1. [file elife-82843-fig1-data1.zip › Fig. 1C SM.tif]

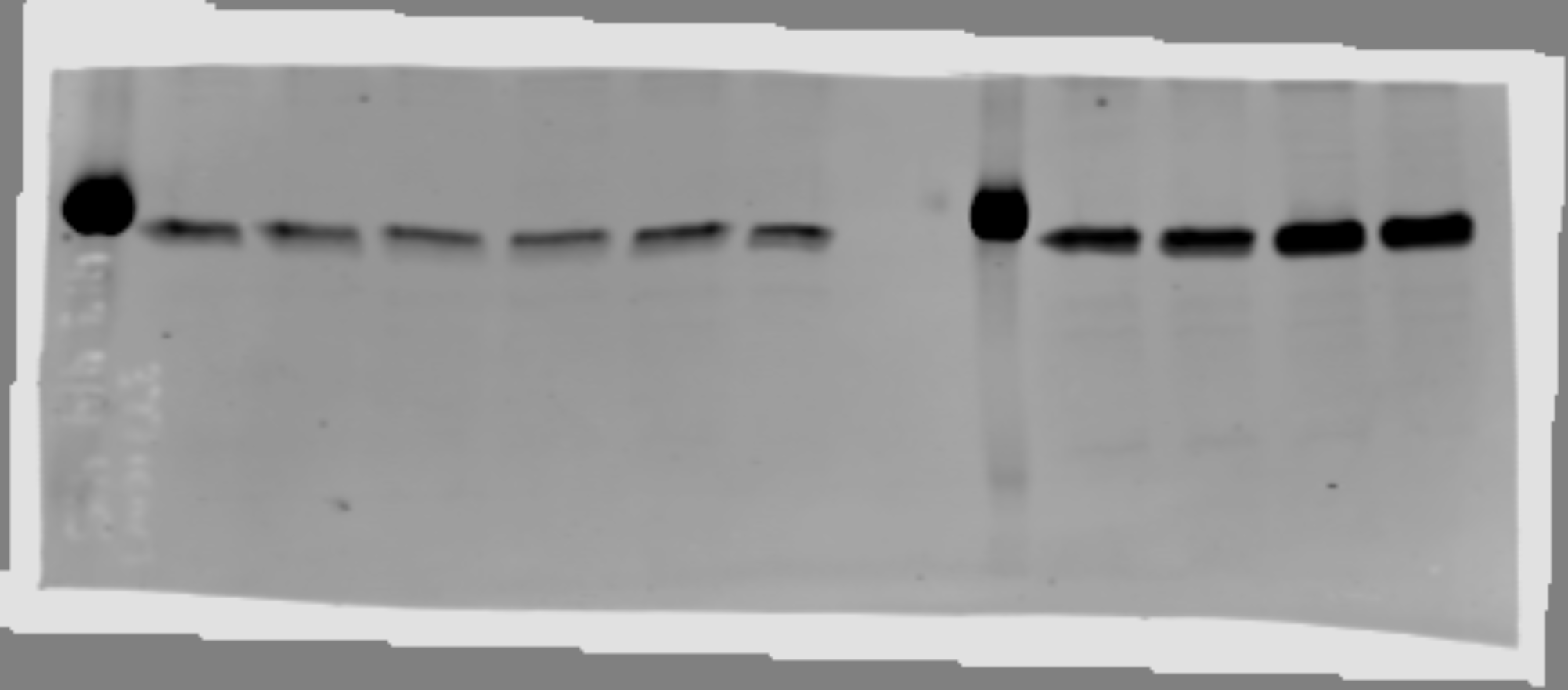

Supplement: Figure 1—source data 1. [file elife-82843-fig1-data1.zip › Fig. 1D-0.5% GAPDH.tif]

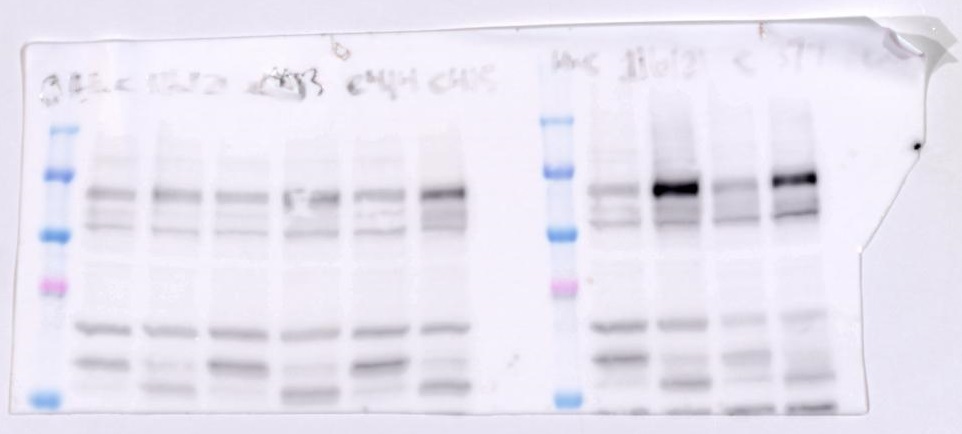

Supplement: Figure 1—source data 1. [file elife-82843-fig1-data1.zip › Fig. 1D-0.5% HIF1a.jpg]

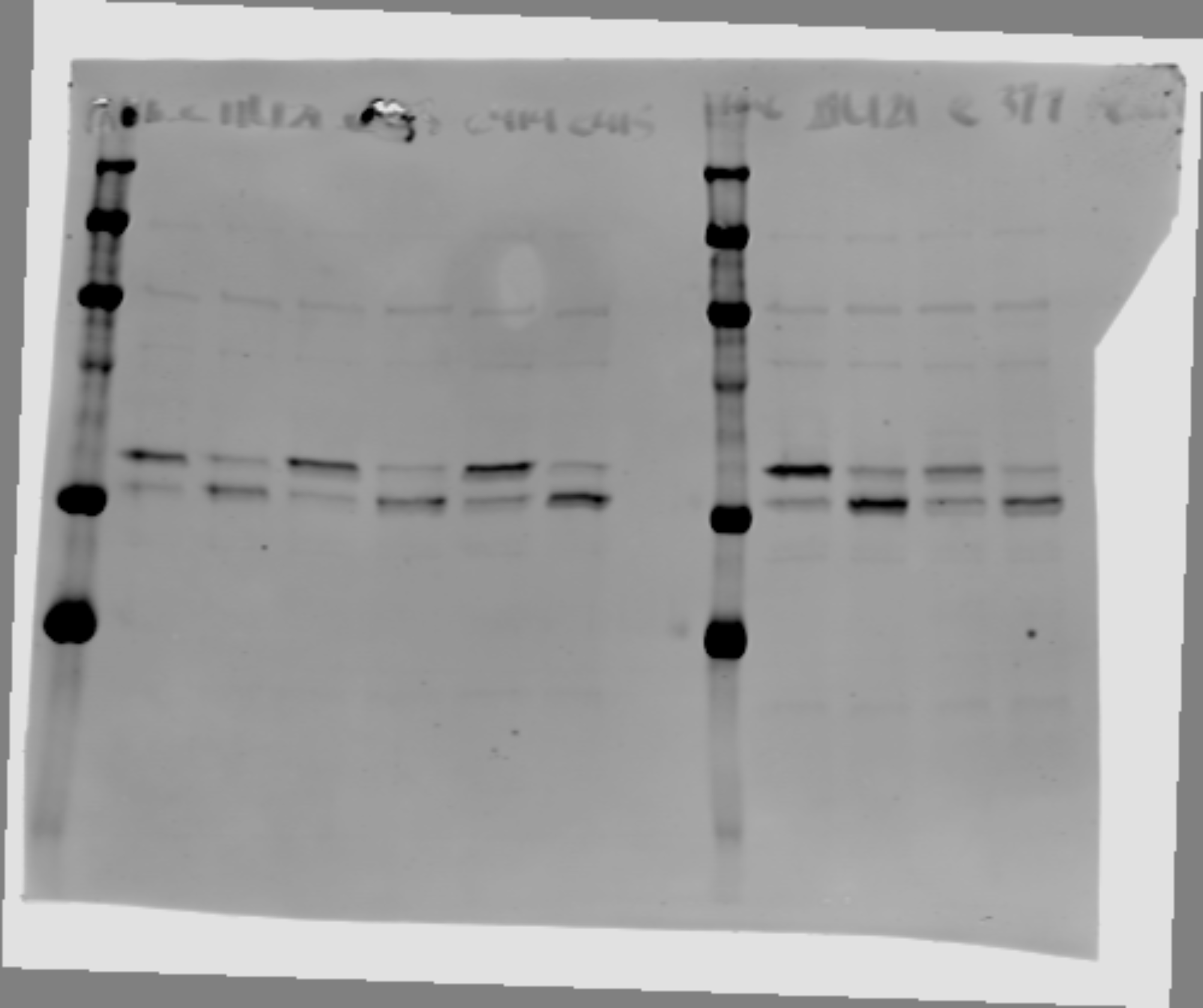

Supplement: Figure 1—source data 1. [file elife-82843-fig1-data1.zip › Fig. 1D-0.5% SM.tif]

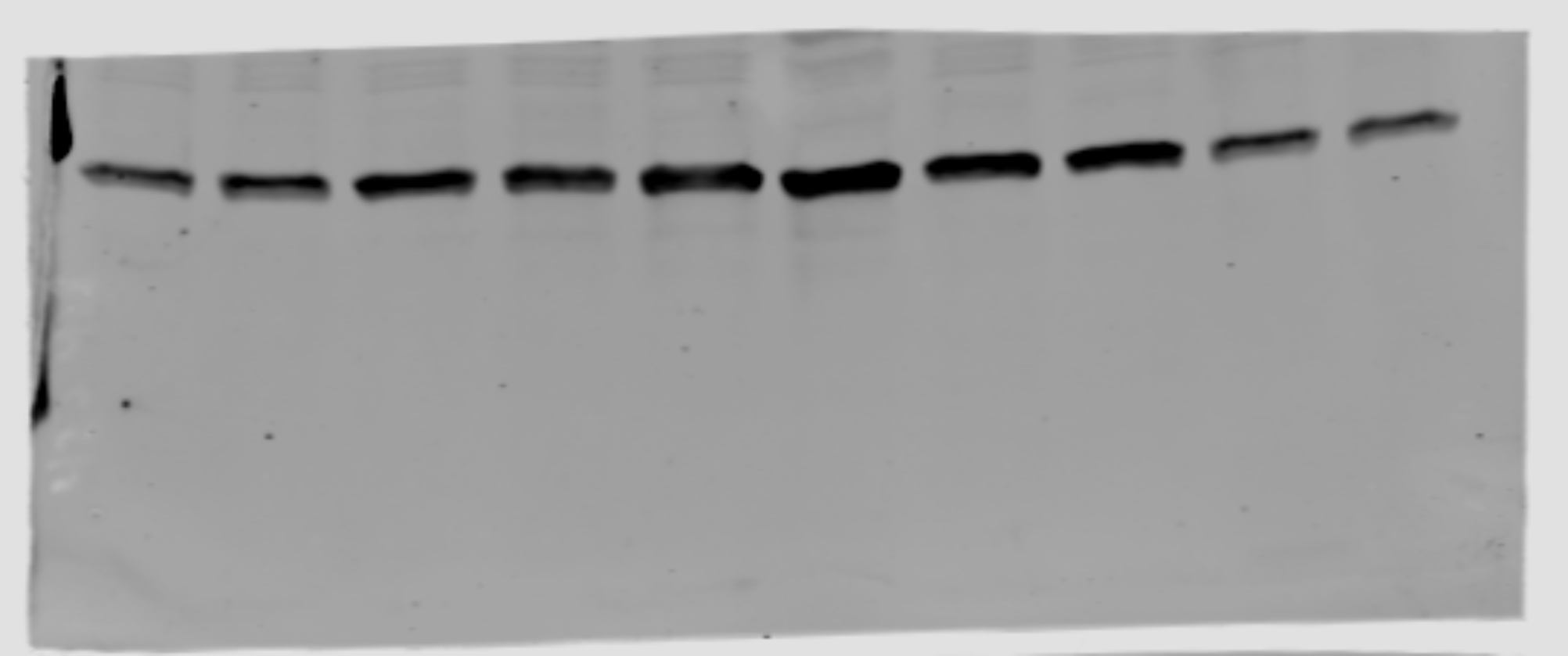

Supplement: Figure 1—source data 1. [file elife-82843-fig1-data1.zip › Fig. 1D-1% GAPDH.tif]

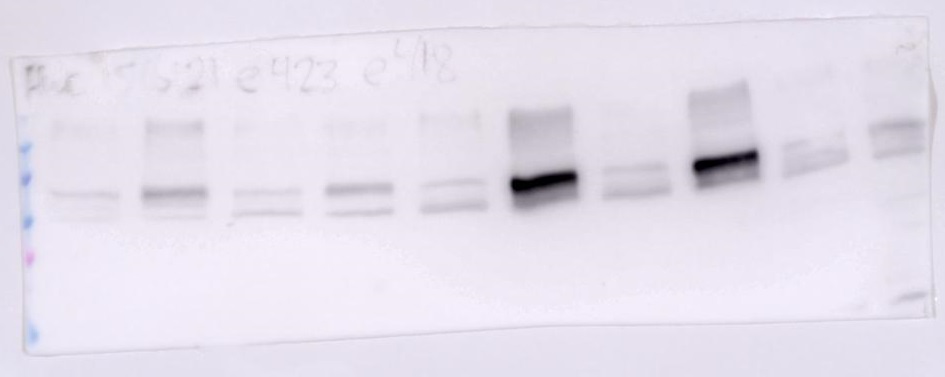

Supplement: Figure 1—source data 1. [file elife-82843-fig1-data1.zip › Fig. 1D-1% HIF1a.jpg]

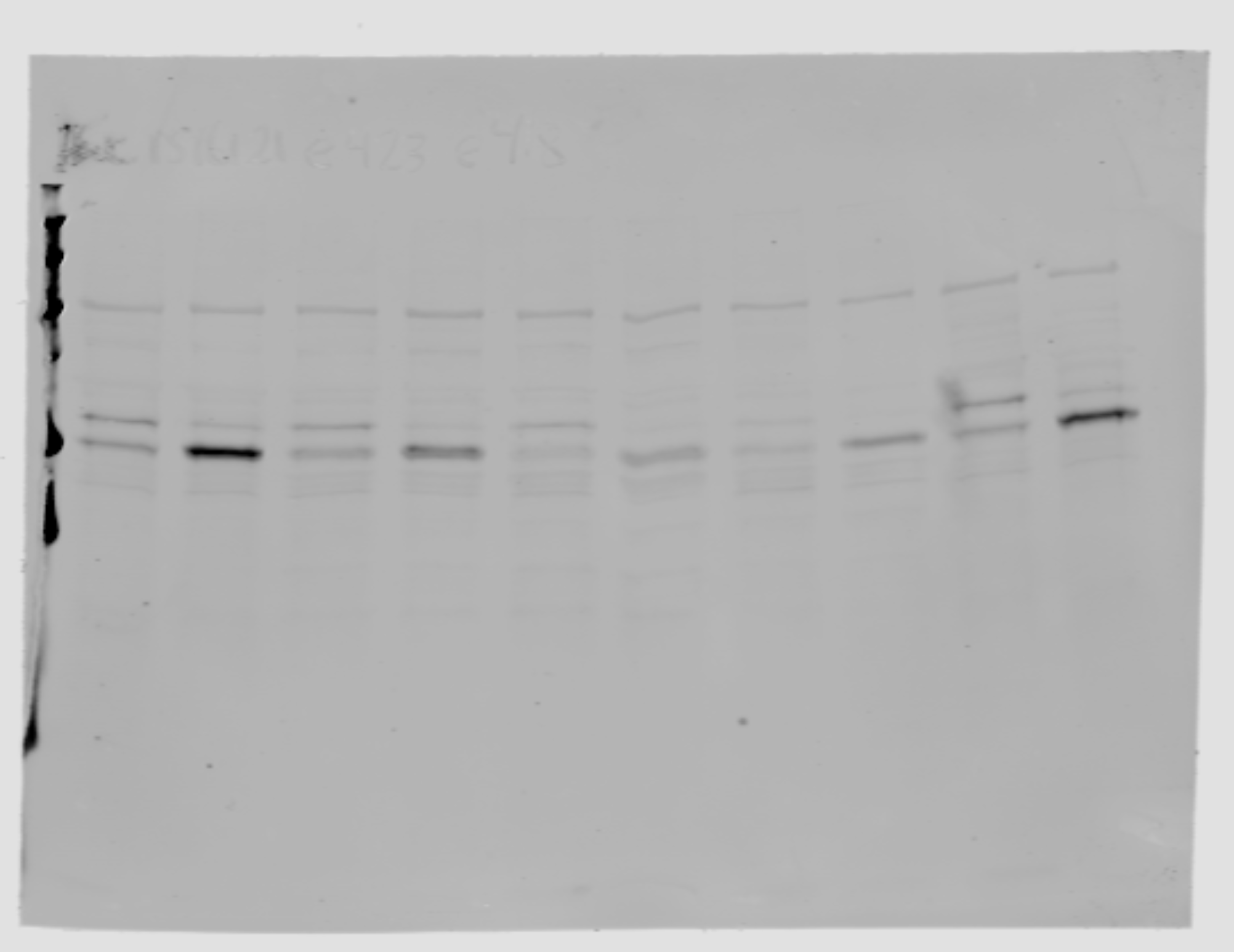

Supplement: Figure 1—source data 1. [file elife-82843-fig1-data1.zip › Fig. 1D-1% SM.tif]

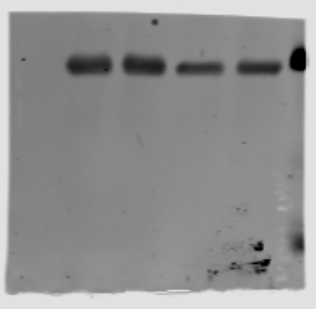

Supplement: Figure 1—source data 1. [file elife-82843-fig1-data1.zip › Fig. 1D-10% GAPDH.tif]

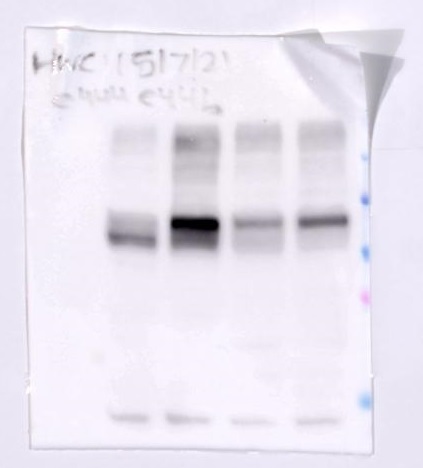

Supplement: Figure 1—source data 1. [file elife-82843-fig1-data1.zip › Fig. 1D-10% HIF1a.jpg]

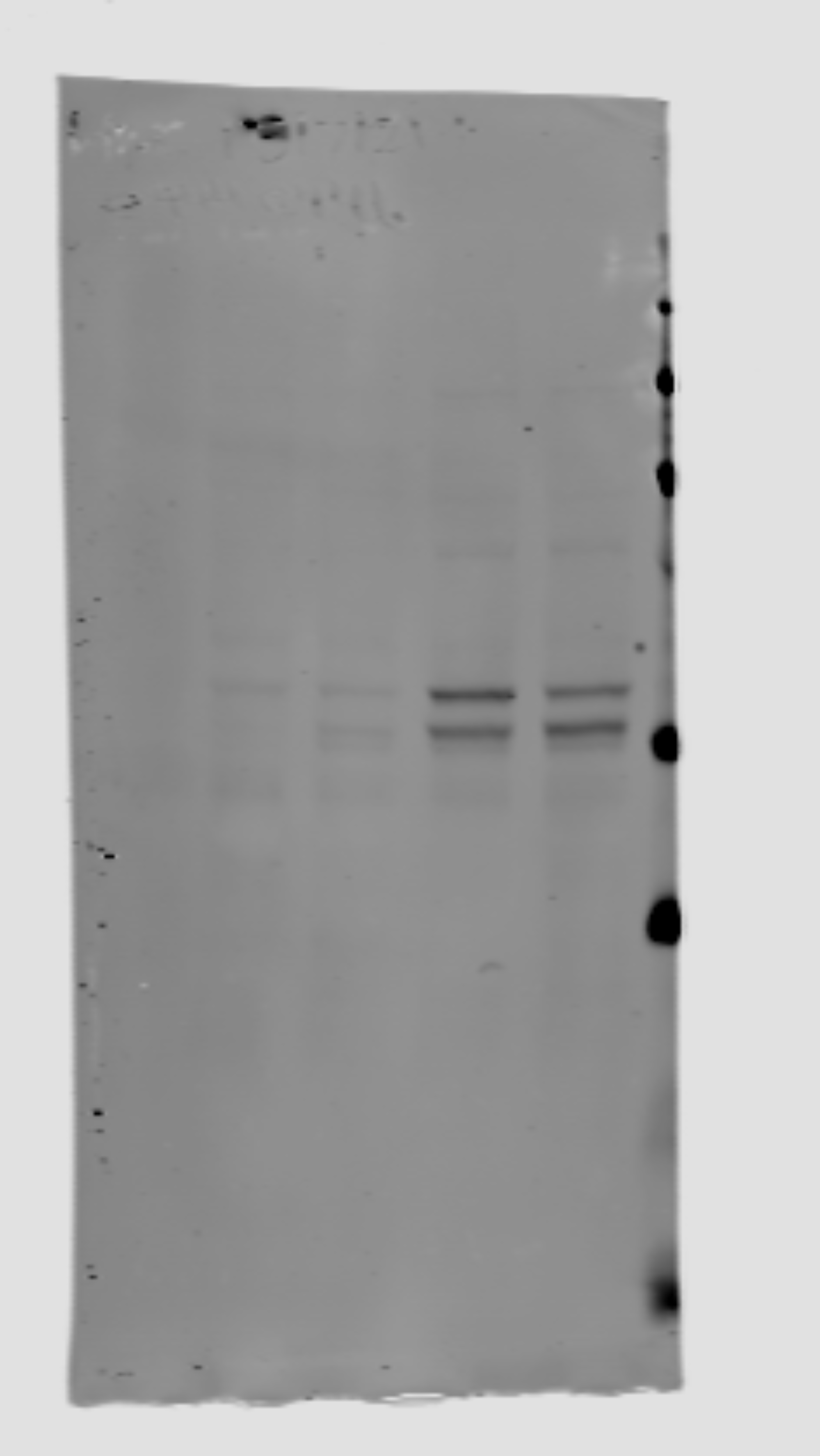

Supplement: Figure 1—source data 1. [file elife-82843-fig1-data1.zip › Fig. 1D-10% SM.tif]

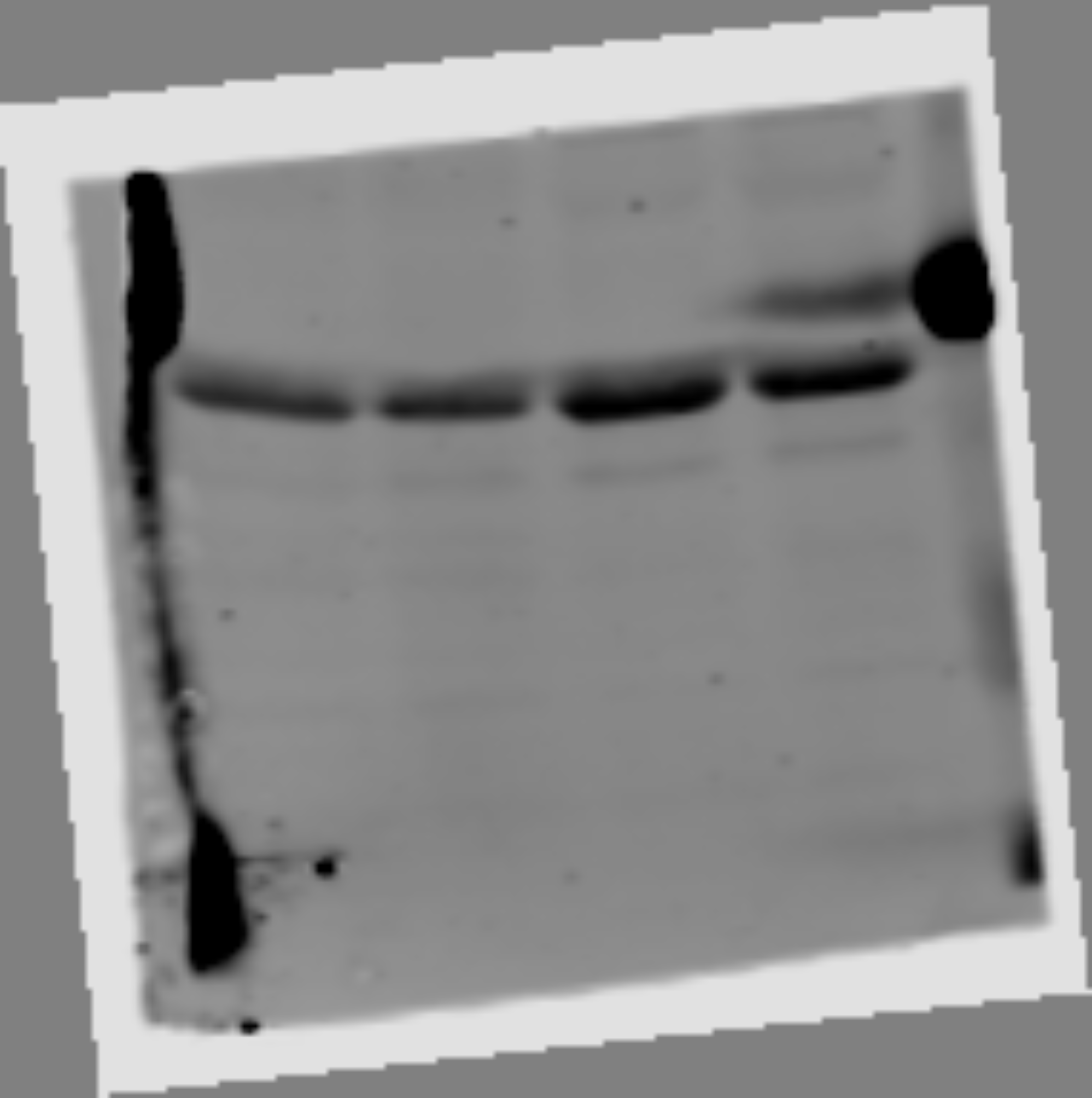

Supplement: Figure 1—source data 1. [file elife-82843-fig1-data1.zip › Fig. 1D-2% GAPDH.tif]

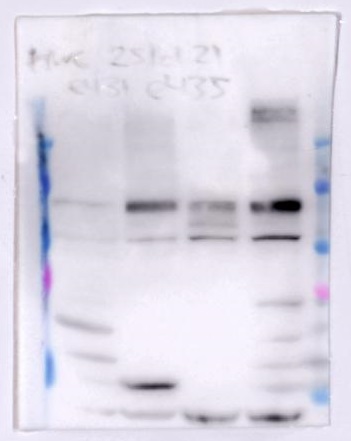

Supplement: Figure 1—source data 1. [file elife-82843-fig1-data1.zip › Fig. 1D-2% HIF1a.jpg]

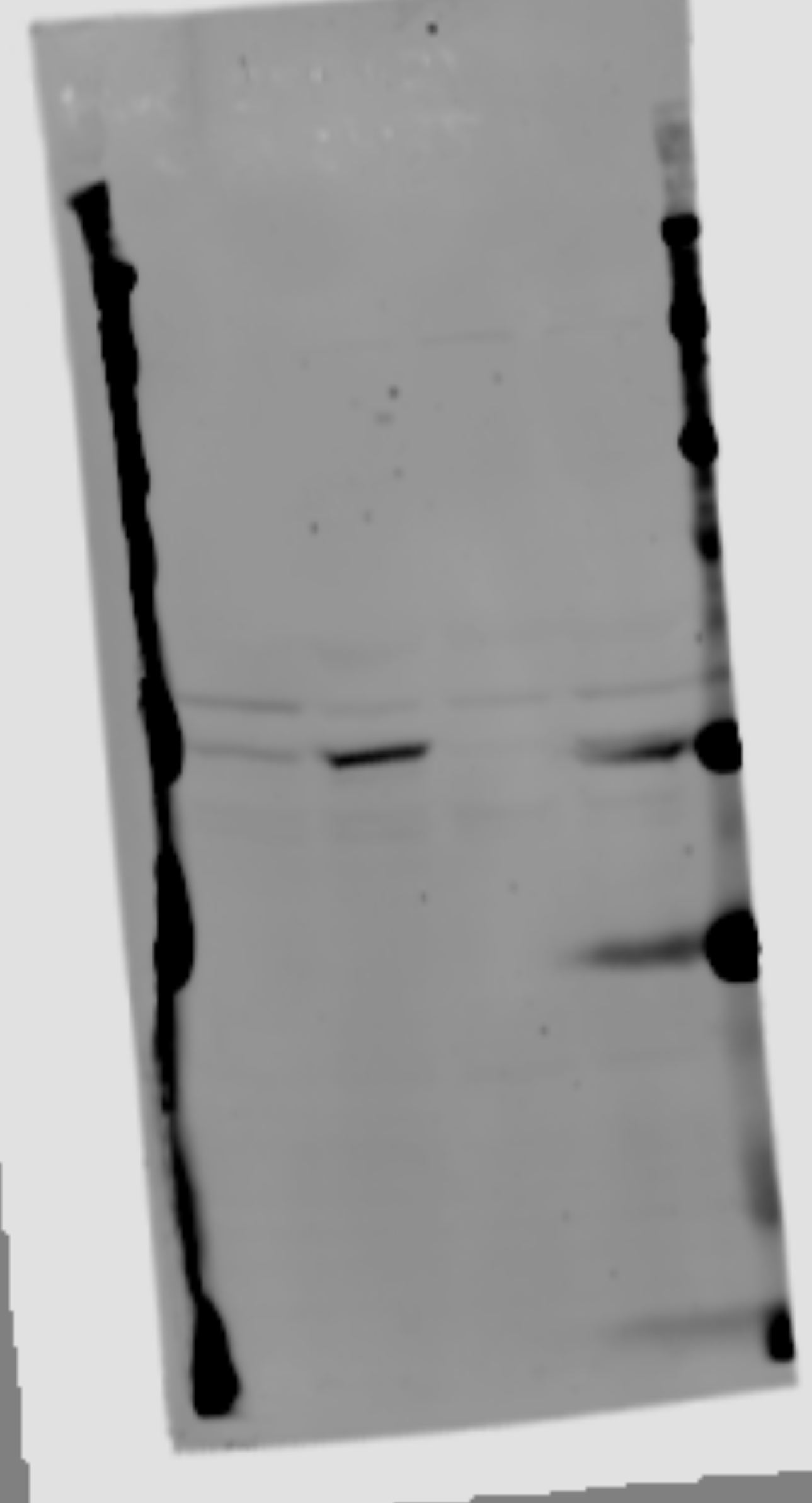

Supplement: Figure 1—source data 1. [file elife-82843-fig1-data1.zip › Fig. 1D-2% SM.tif]

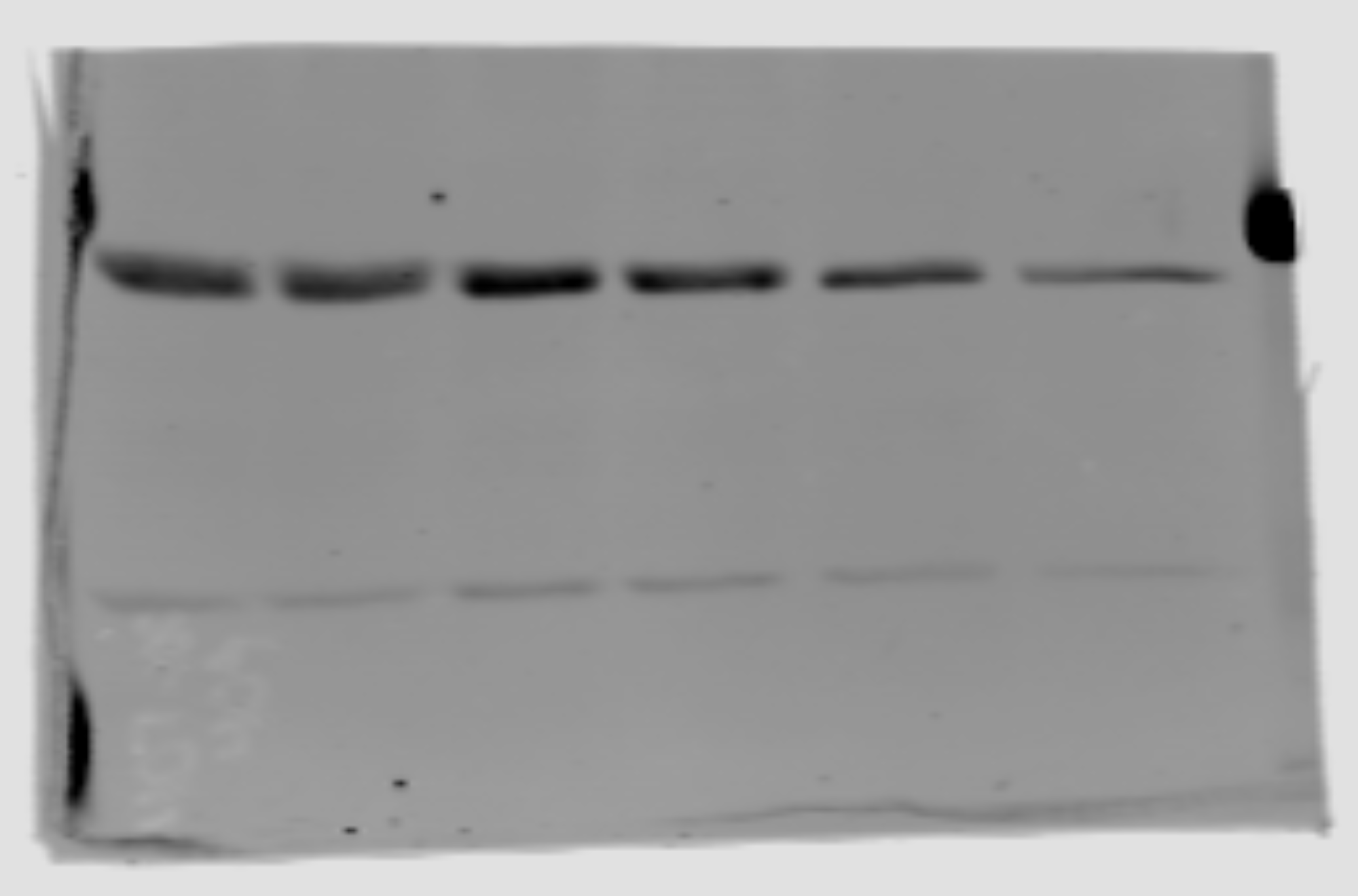

Supplement: Figure 1—source data 1. [file elife-82843-fig1-data1.zip › Fig. 1D-3% GAPDH.tif]

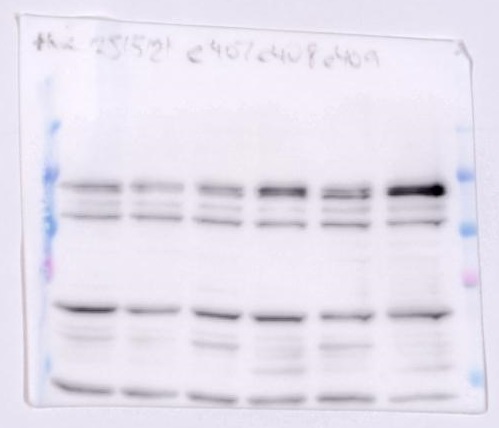

Supplement: Figure 1—source data 1. [file elife-82843-fig1-data1.zip › Fig. 1D-3% HIF1a.jpg]

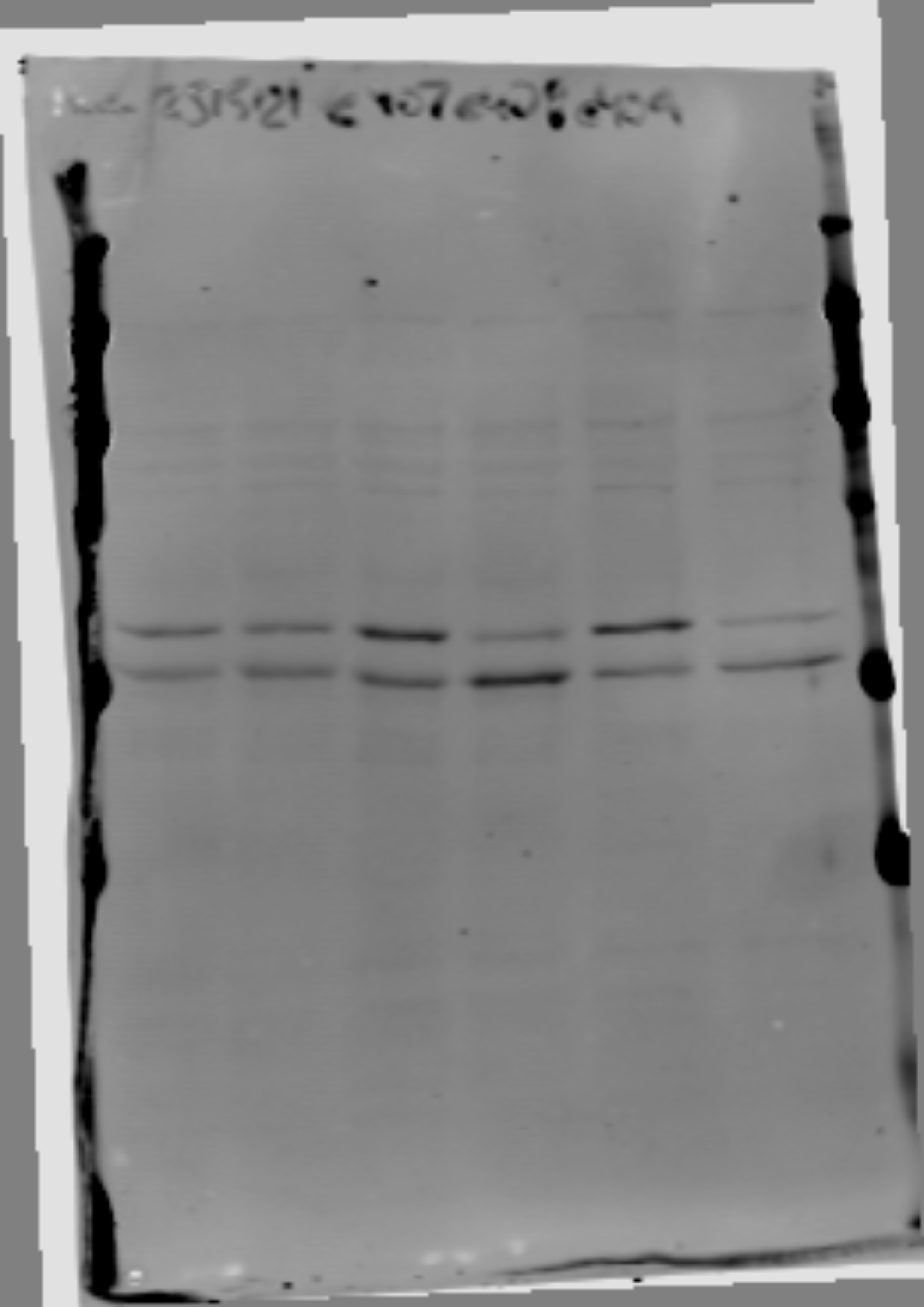

Supplement: Figure 1—source data 1. [file elife-82843-fig1-data1.zip › Fig. 1D-3% SM.tif]

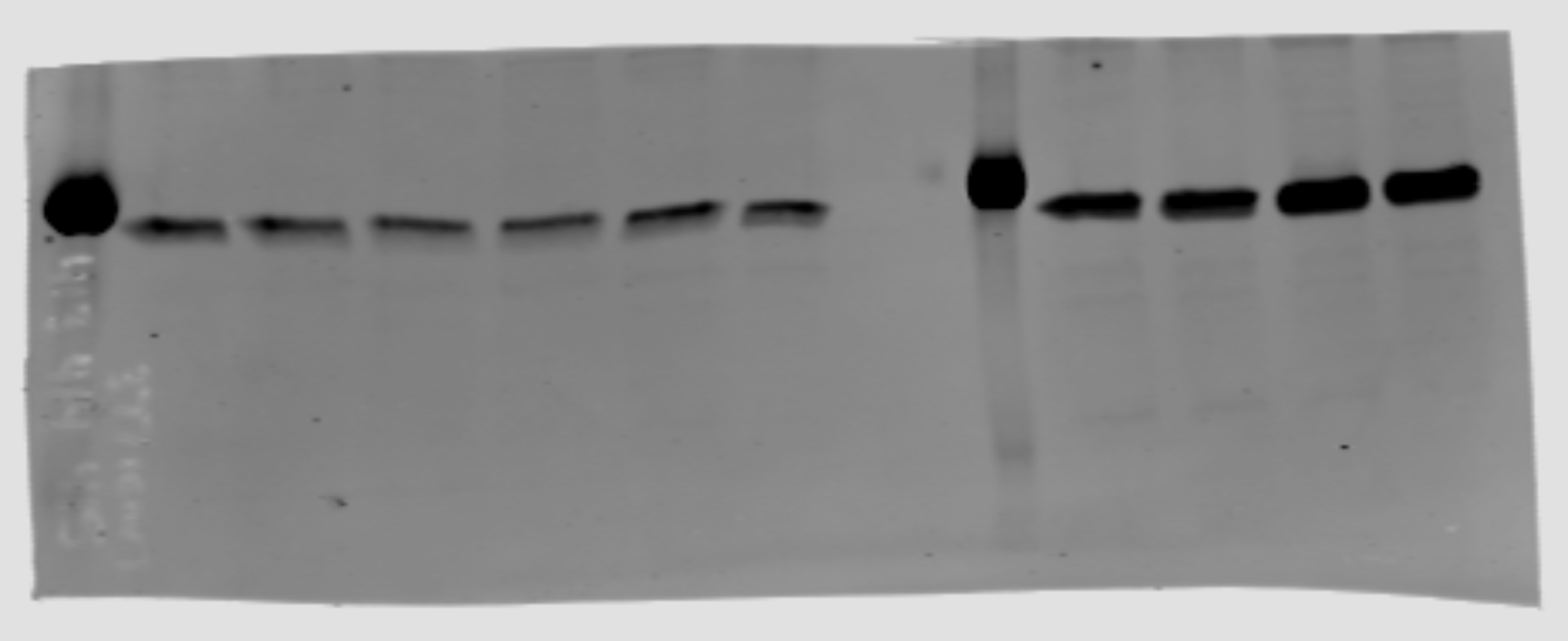

Supplement: Figure 1—source data 1. [file elife-82843-fig1-data1.zip › Fig. 1D-4% GAPDH.tif]

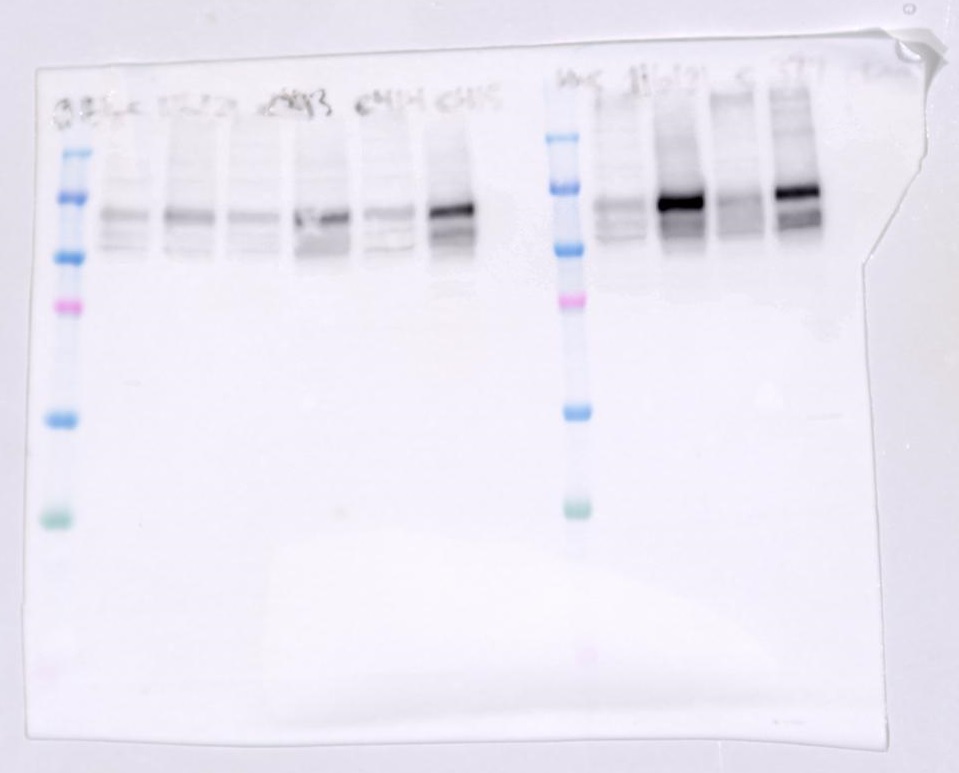

Supplement: Figure 1—source data 1. [file elife-82843-fig1-data1.zip › Fig. 1D-4% HIF1a.jpg]

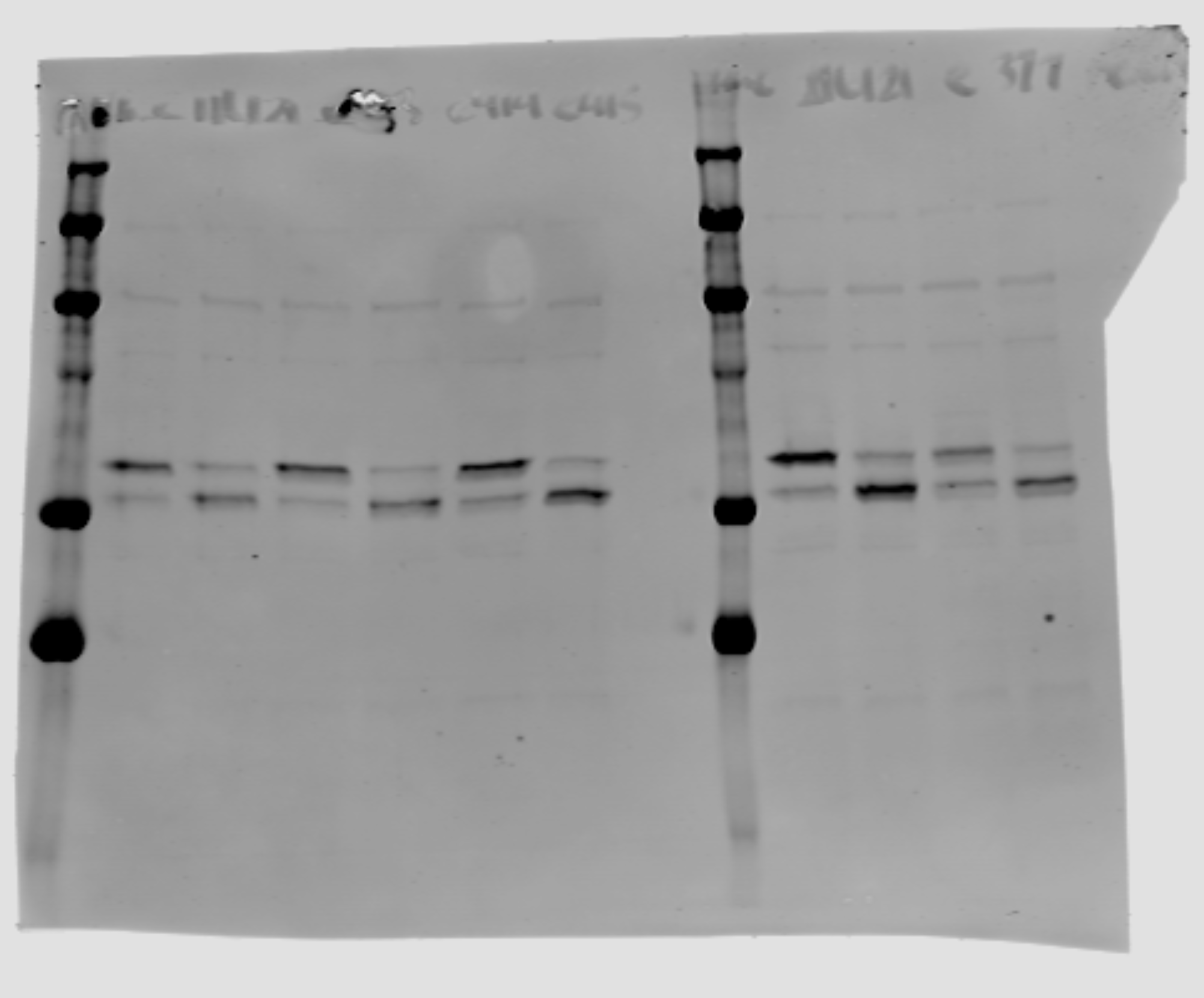

Supplement: Figure 1—source data 1. [file elife-82843-fig1-data1.zip › Fig. 1D-4% SM.tif]

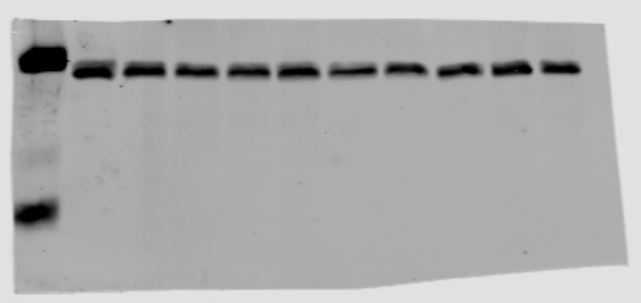

Supplement: Figure 1—source data 1. [file elife-82843-fig1-data1.zip › Fig. 1D-5% GAPDH.tif]

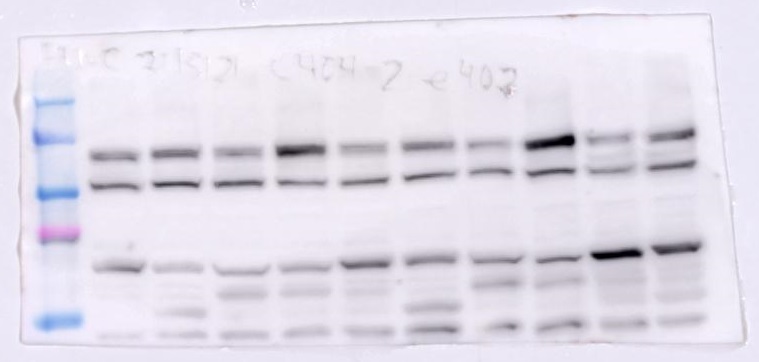

Supplement: Figure 1—source data 1. [file elife-82843-fig1-data1.zip › Fig. 1D-5% HIF1a.jpg]

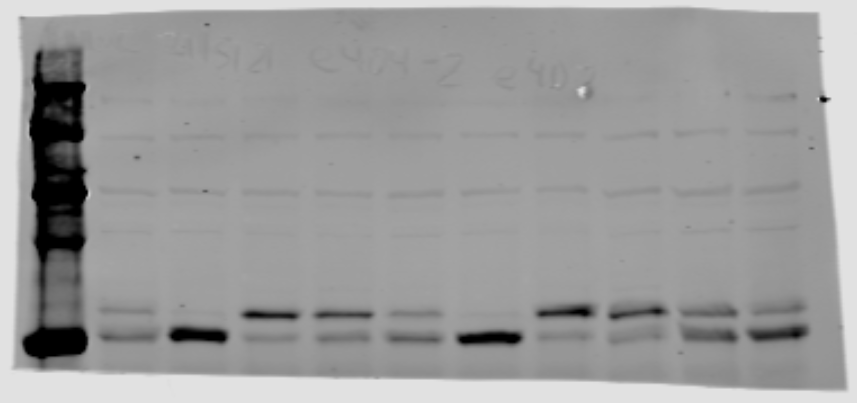

Supplement: Figure 1—source data 1. [file elife-82843-fig1-data1.zip › Fig. 1D-5% SM.tif]

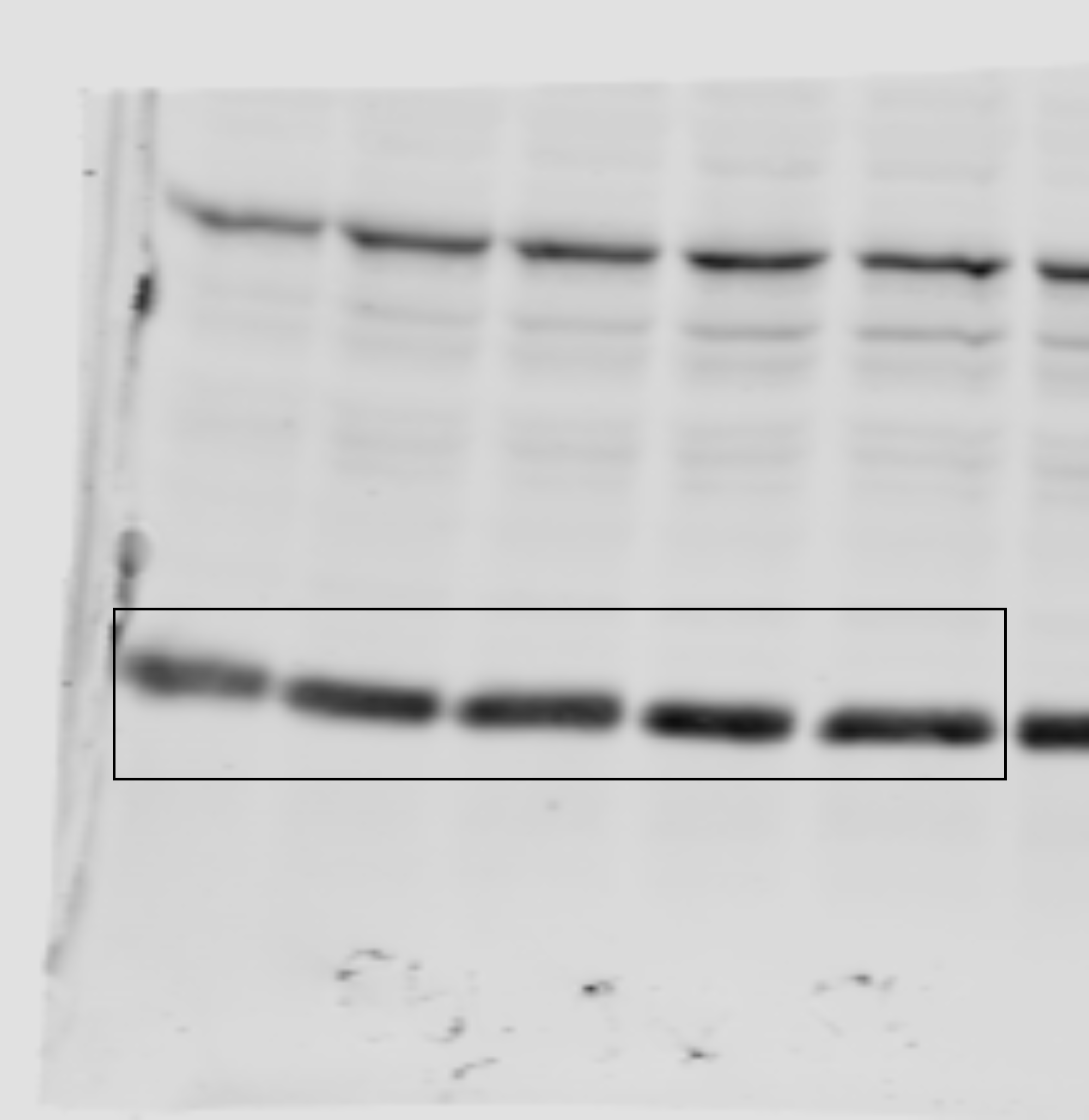

Supplement: Figure 1—figure supplement 2—source data 1. [file elife-82843-fig1-figsupp2-data1.zip › Annotated/Fig. 1-2E GAPDH.tif]

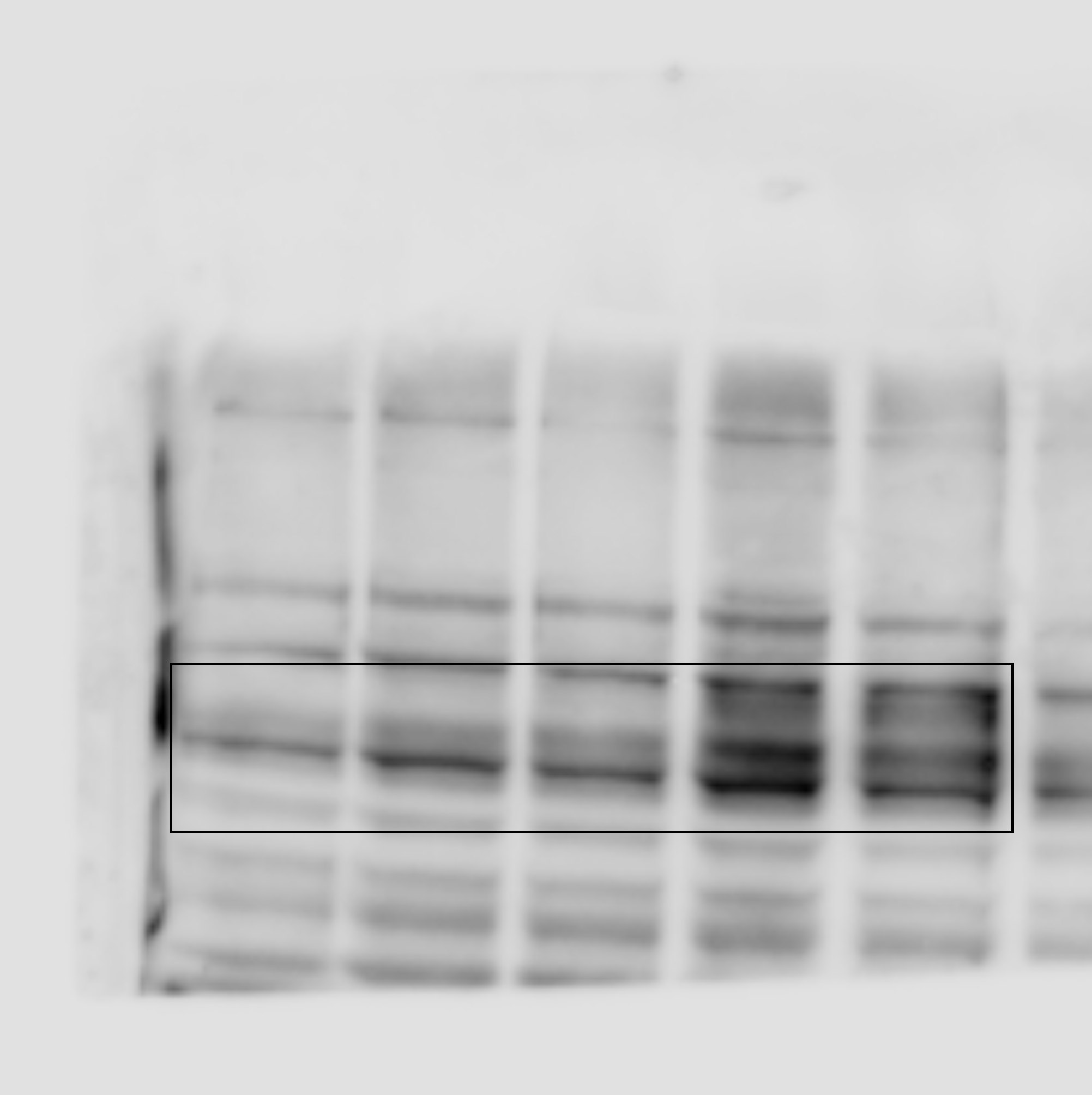

Supplement: Figure 1—figure supplement 2—source data 1. [file elife-82843-fig1-figsupp2-data1.zip › Annotated/Fig. 1-2E HIF1a.tif]

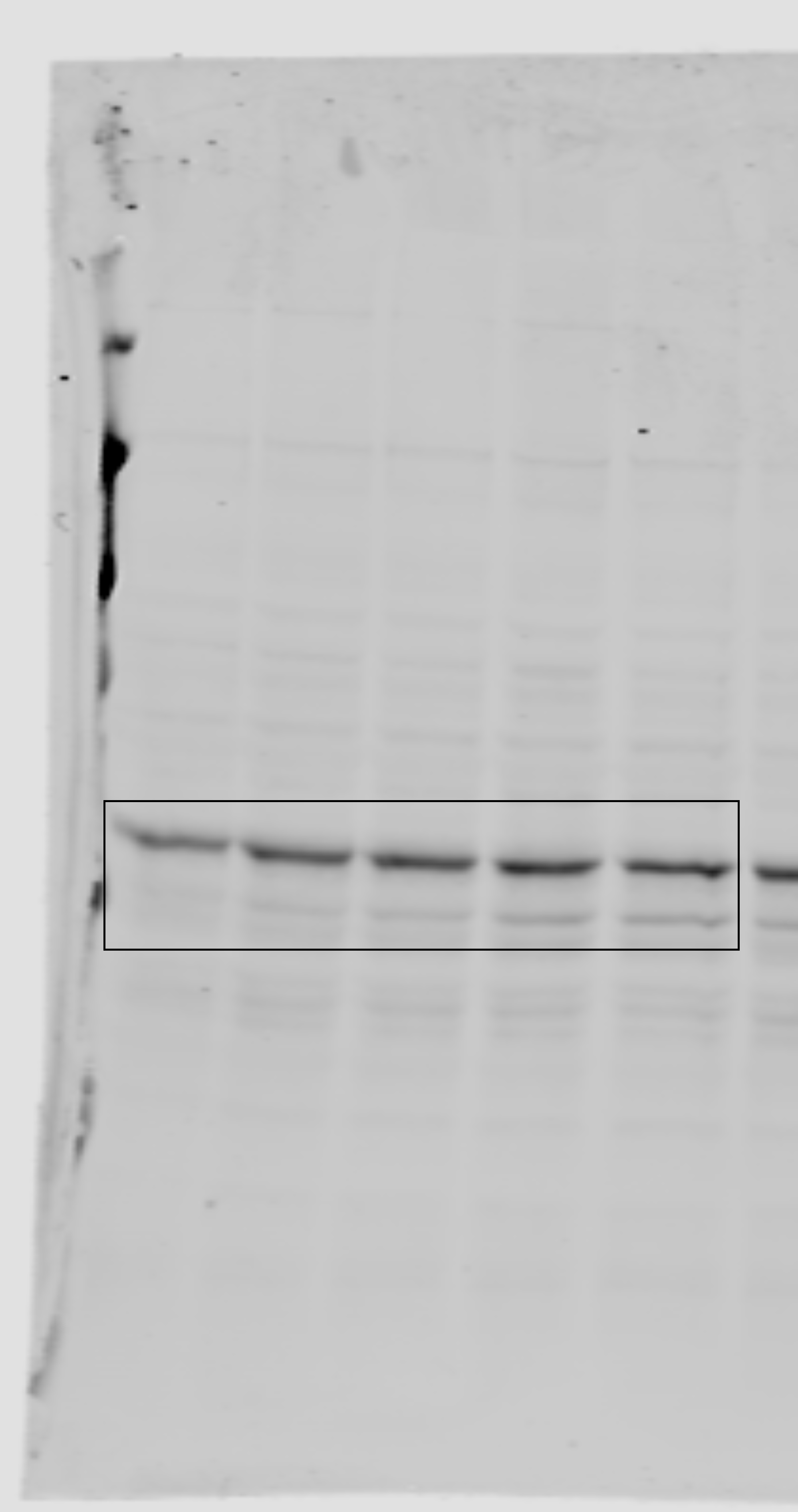

Supplement: Figure 1—figure supplement 2—source data 1. [file elife-82843-fig1-figsupp2-data1.zip › Annotated/Fig. 1-2E SM.tif]

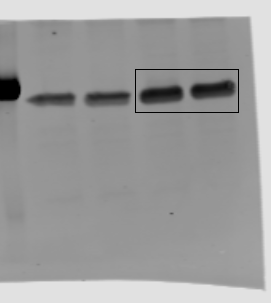

Supplement: Figure 1—figure supplement 2—source data 1. [file elife-82843-fig1-figsupp2-data1.zip › Annotated/Fig. 1-2F-HCT116 GAPDH.tif]

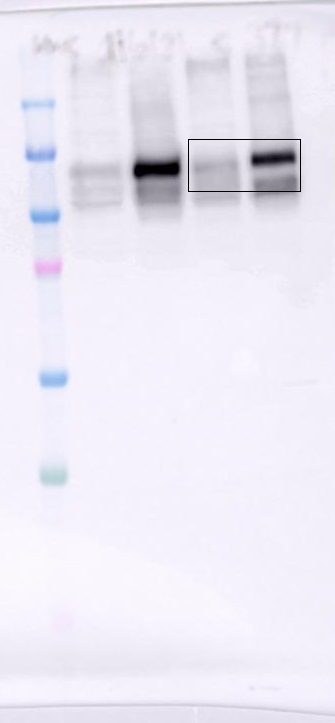

Supplement: Figure 1—figure supplement 2—source data 1. [file elife-82843-fig1-figsupp2-data1.zip › Annotated/Fig. 1-2F-HCT116 HIF1a.jpg]

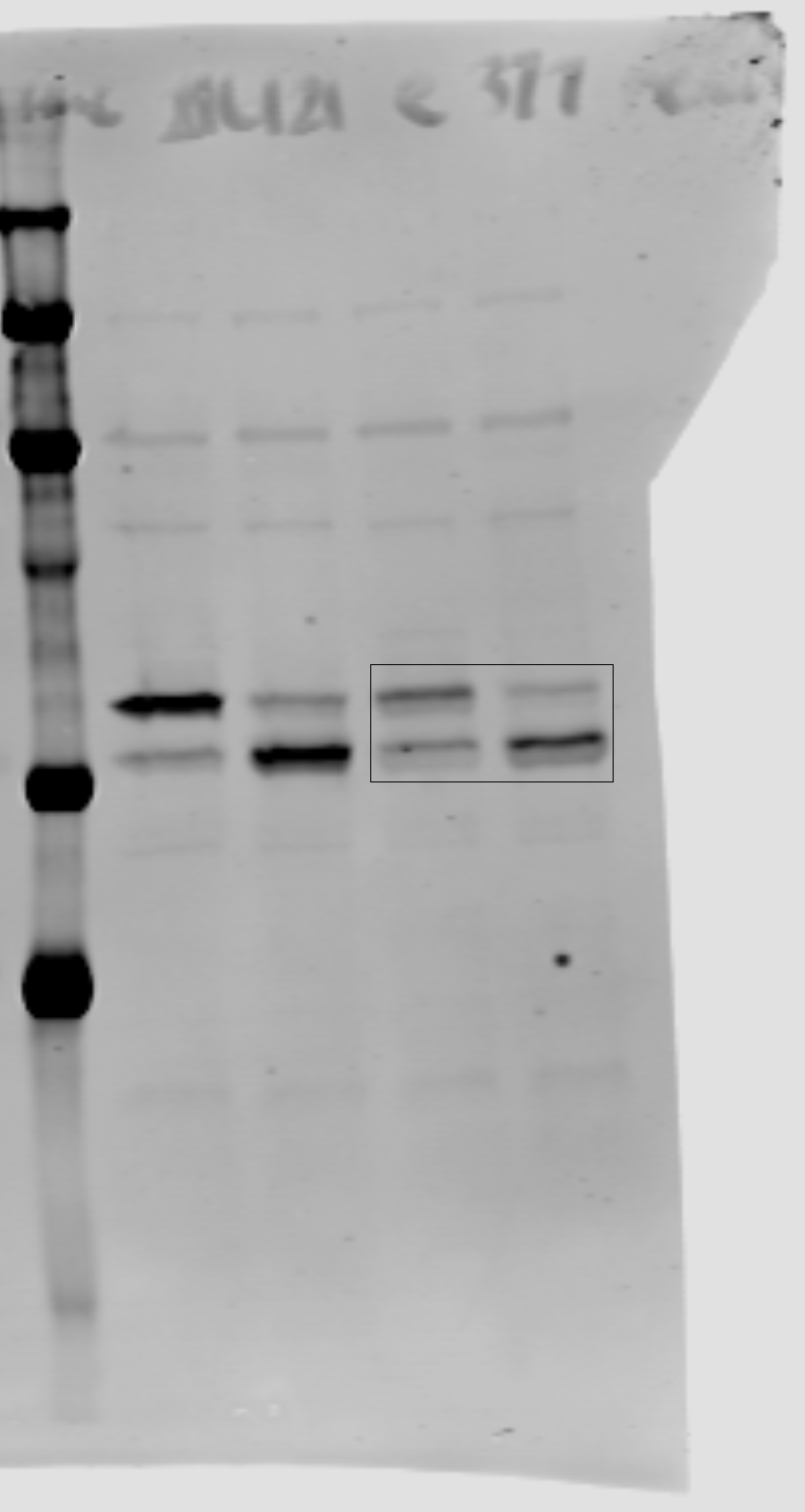

Supplement: Figure 1—figure supplement 2—source data 1. [file elife-82843-fig1-figsupp2-data1.zip › Annotated/Fig. 1-2F-HCT116 SM.tif]

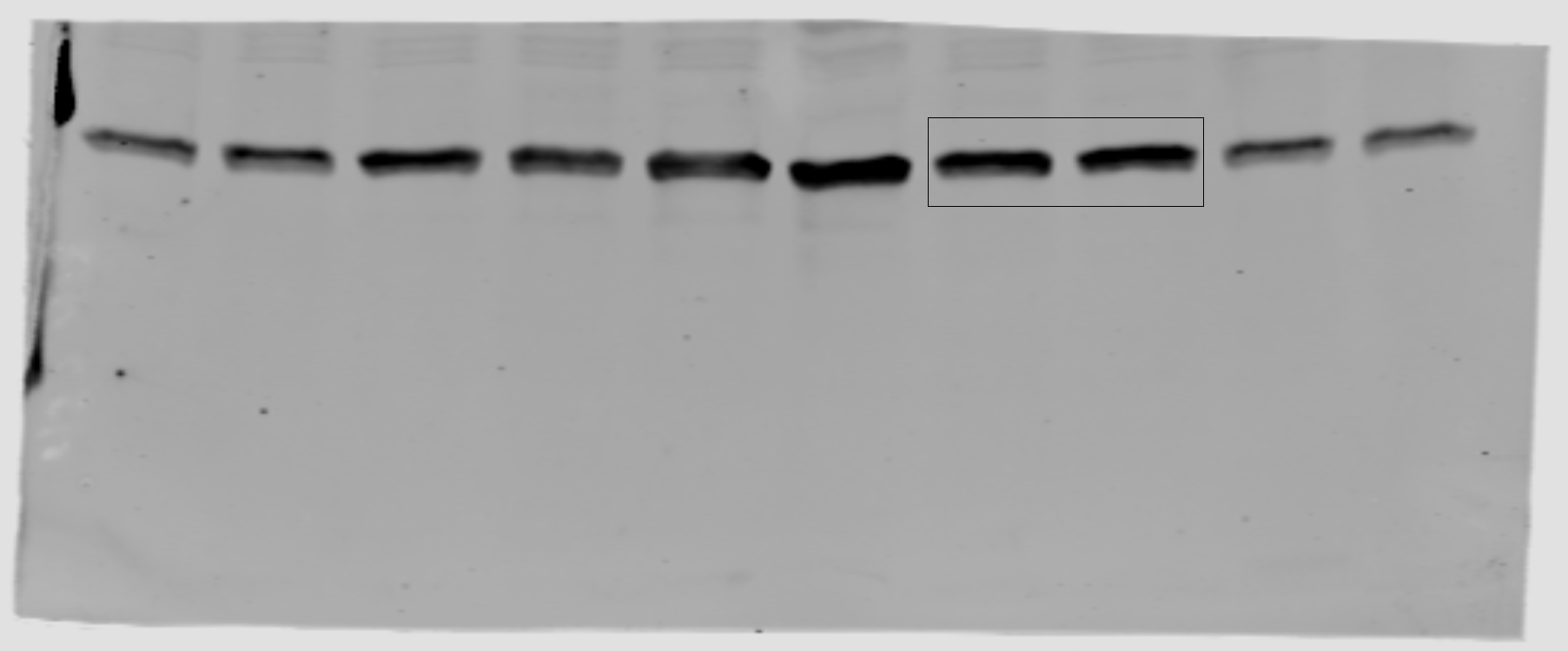

Supplement: Figure 1—figure supplement 2—source data 1. [file elife-82843-fig1-figsupp2-data1.zip › Annotated/Fig. 1-2F-HeLa GAPDH.tif]

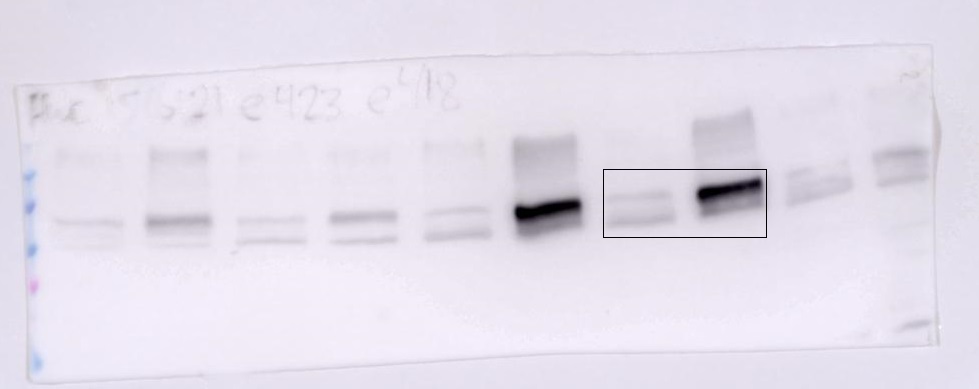

Supplement: Figure 1—figure supplement 2—source data 1. [file elife-82843-fig1-figsupp2-data1.zip › Annotated/Fig. 1-2F-HeLa HIF1a.jpg]

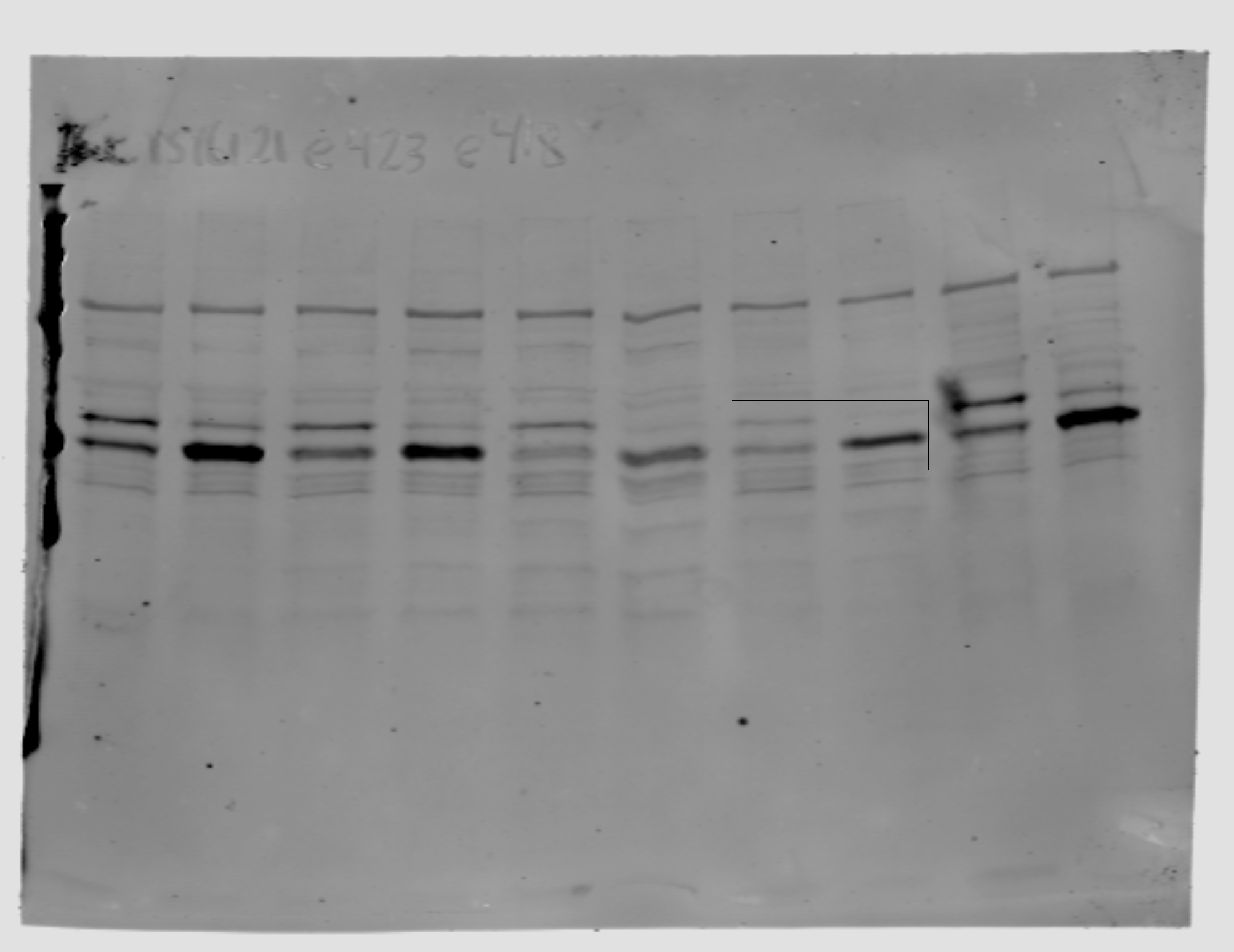

Supplement: Figure 1—figure supplement 2—source data 1. [file elife-82843-fig1-figsupp2-data1.zip › Annotated/Fig. 1-2F-HeLa SM.tif]

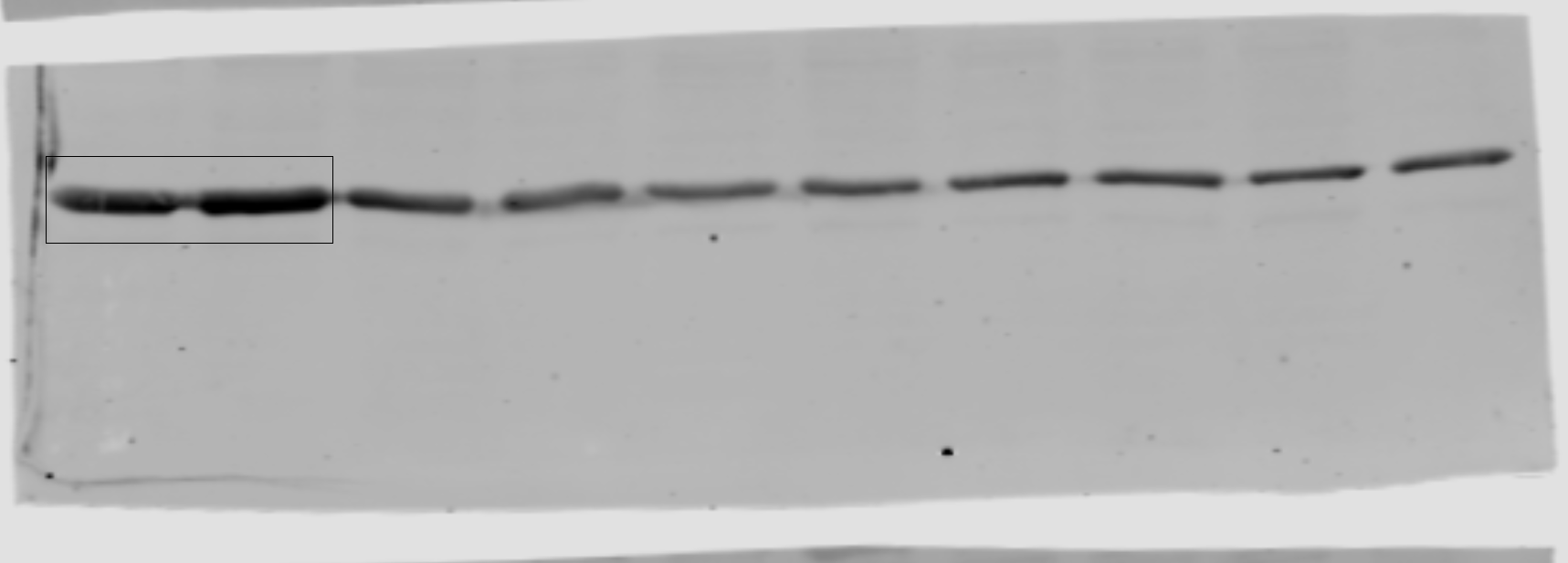

Supplement: Figure 1—figure supplement 2—source data 1. [file elife-82843-fig1-figsupp2-data1.zip › Annotated/Fig. 1-2F-Huh7 GAPDH.tif]

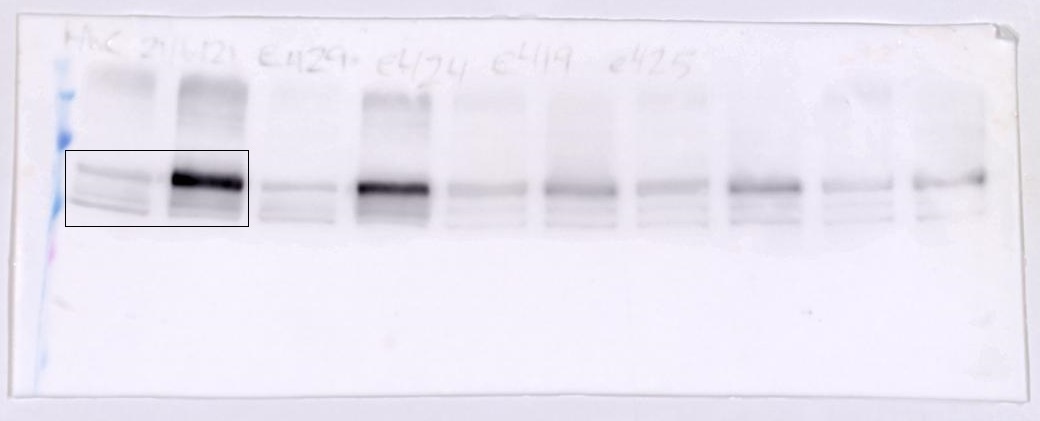

Supplement: Figure 1—figure supplement 2—source data 1. [file elife-82843-fig1-figsupp2-data1.zip › Annotated/Fig. 1-2F-Huh7 HIF1a.jpg]

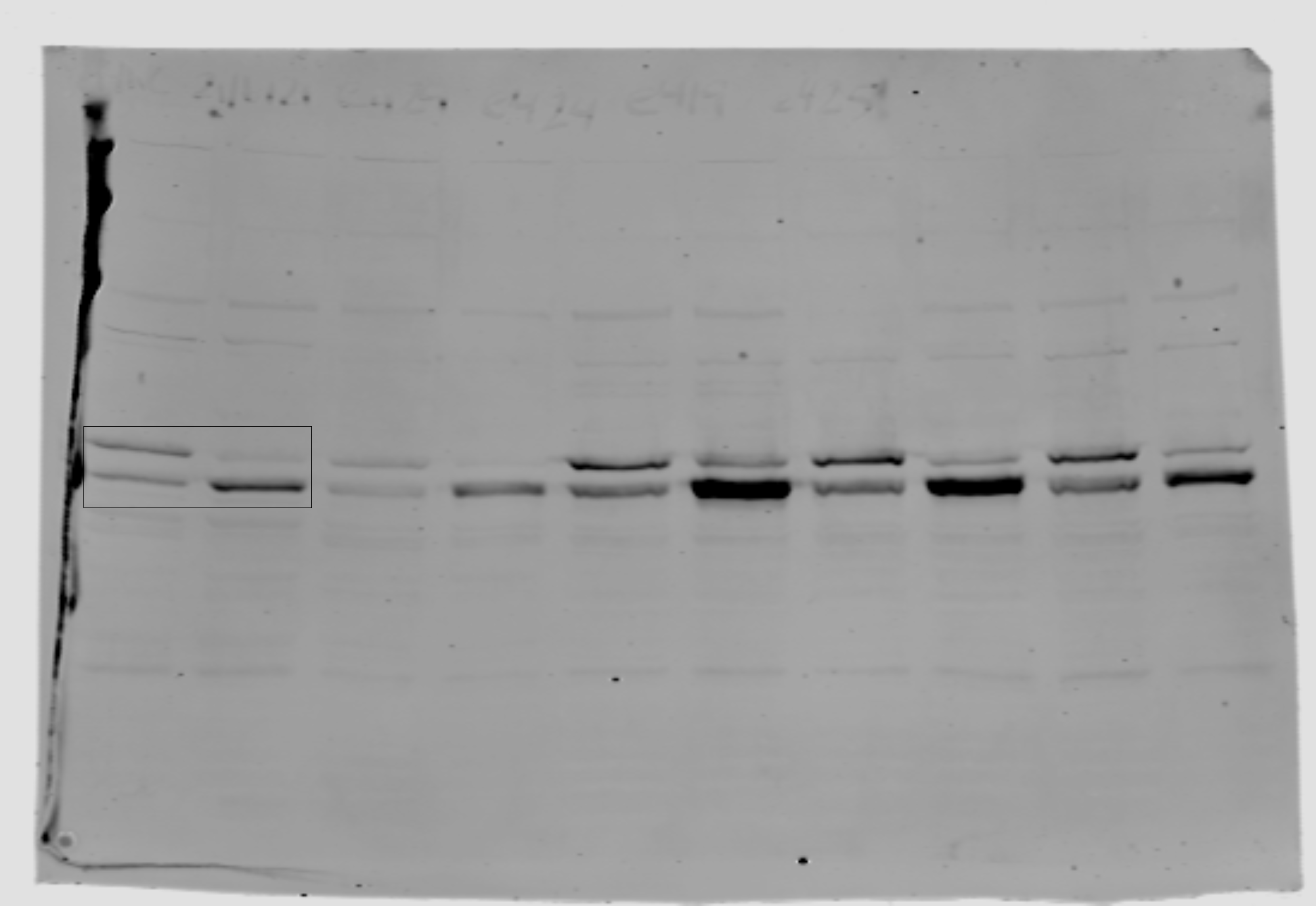

Supplement: Figure 1—figure supplement 2—source data 1. [file elife-82843-fig1-figsupp2-data1.zip › Annotated/Fig. 1-2F-Huh7 SM.tif]

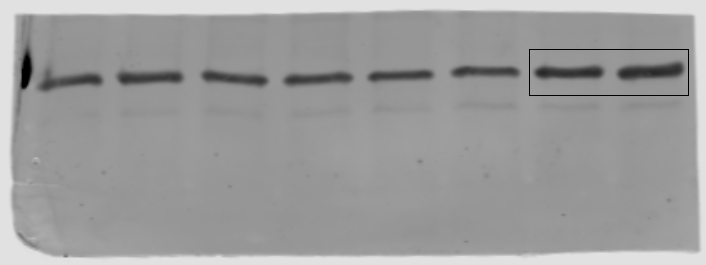

Supplement: Figure 1—figure supplement 2—source data 1. [file elife-82843-fig1-figsupp2-data1.zip › Annotated/Fig. 1-2F-MDA-MB-231 GAPDH.tif]

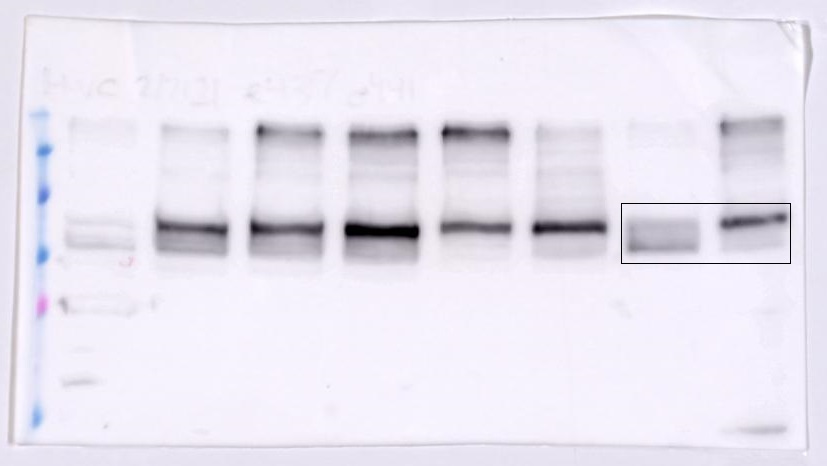

Supplement: Figure 1—figure supplement 2—source data 1. [file elife-82843-fig1-figsupp2-data1.zip › Annotated/Fig. 1-2F-MDA-MB-231 HIF1a.jpg]

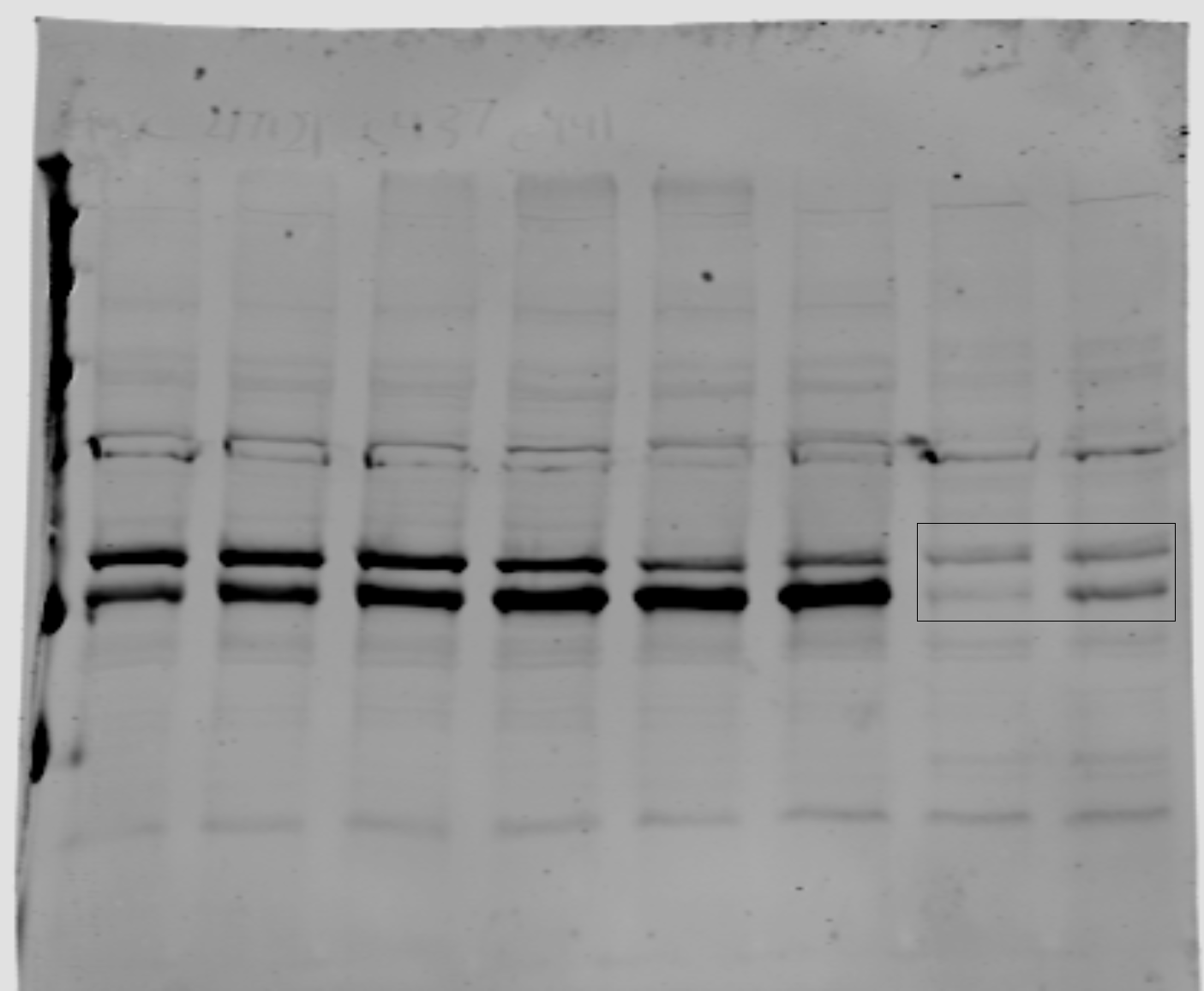

Supplement: Figure 1—figure supplement 2—source data 1. [file elife-82843-fig1-figsupp2-data1.zip › Annotated/Fig. 1-2F-MDA-MB-231 SM.tif]

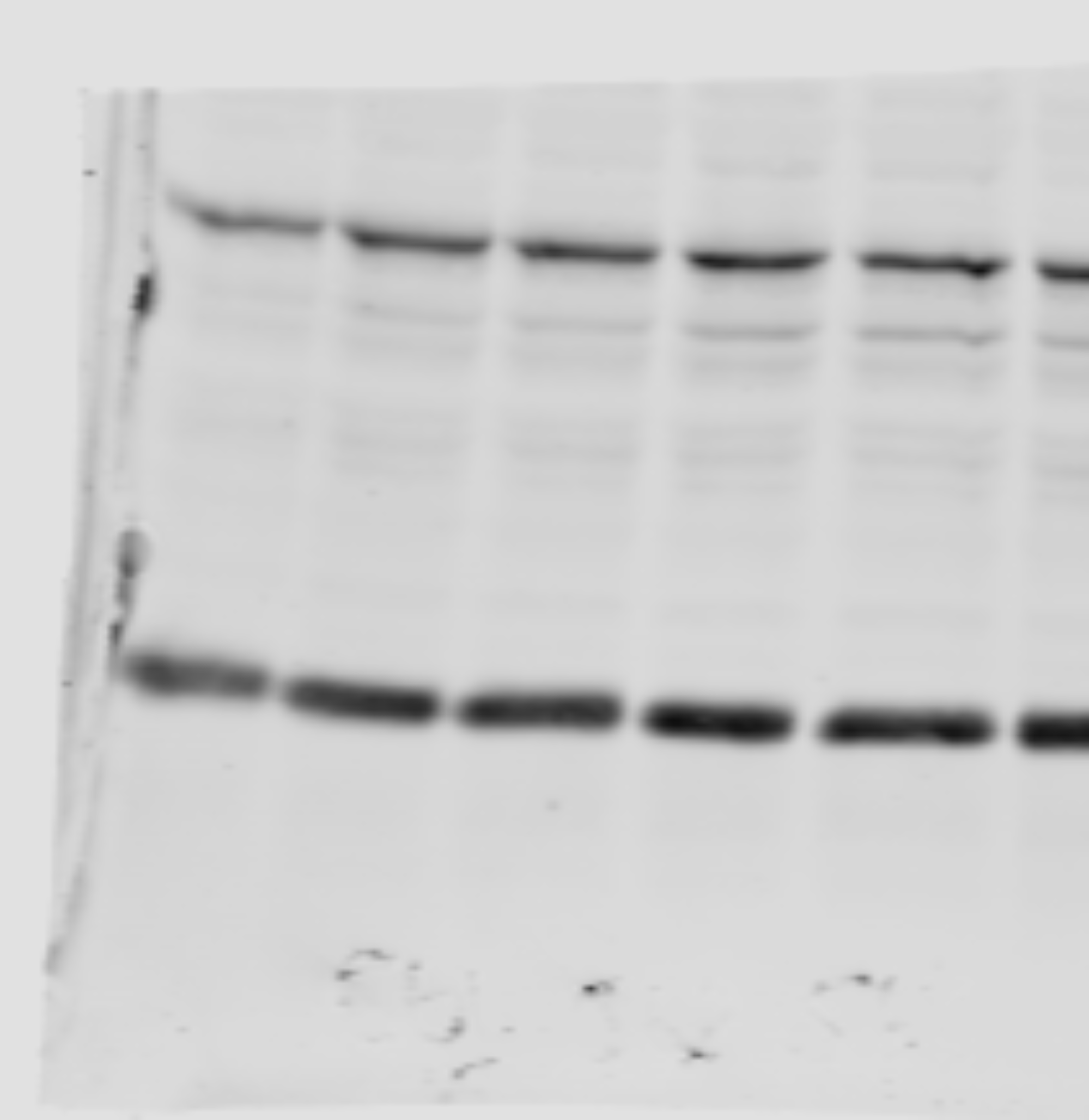

Supplement: Figure 1—figure supplement 2—source data 1. [file elife-82843-fig1-figsupp2-data1.zip › Fig. 1-2E GAPDH.tif]

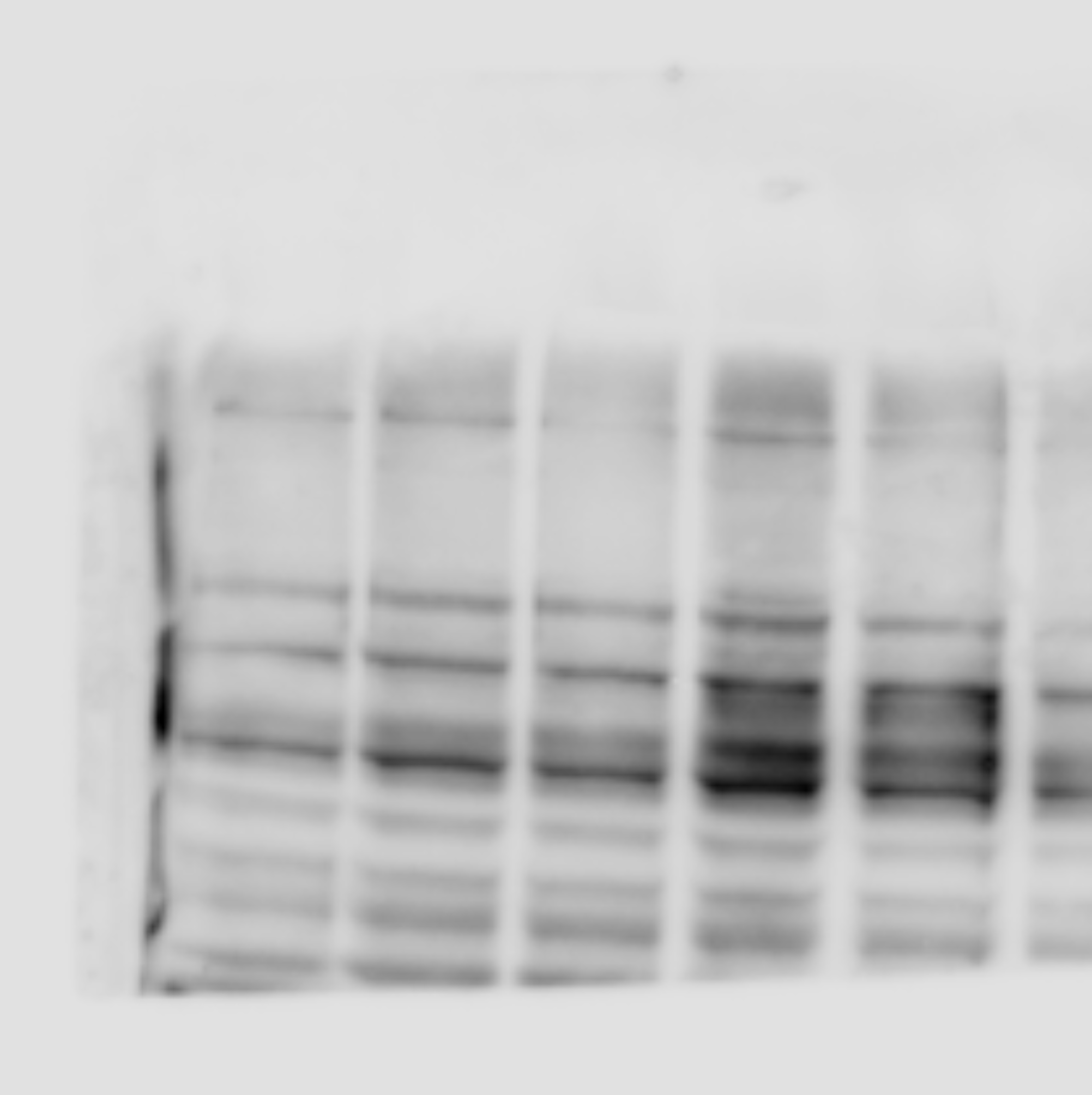

Supplement: Figure 1—figure supplement 2—source data 1. [file elife-82843-fig1-figsupp2-data1.zip › Fig. 1-2E HIF1a.tif]

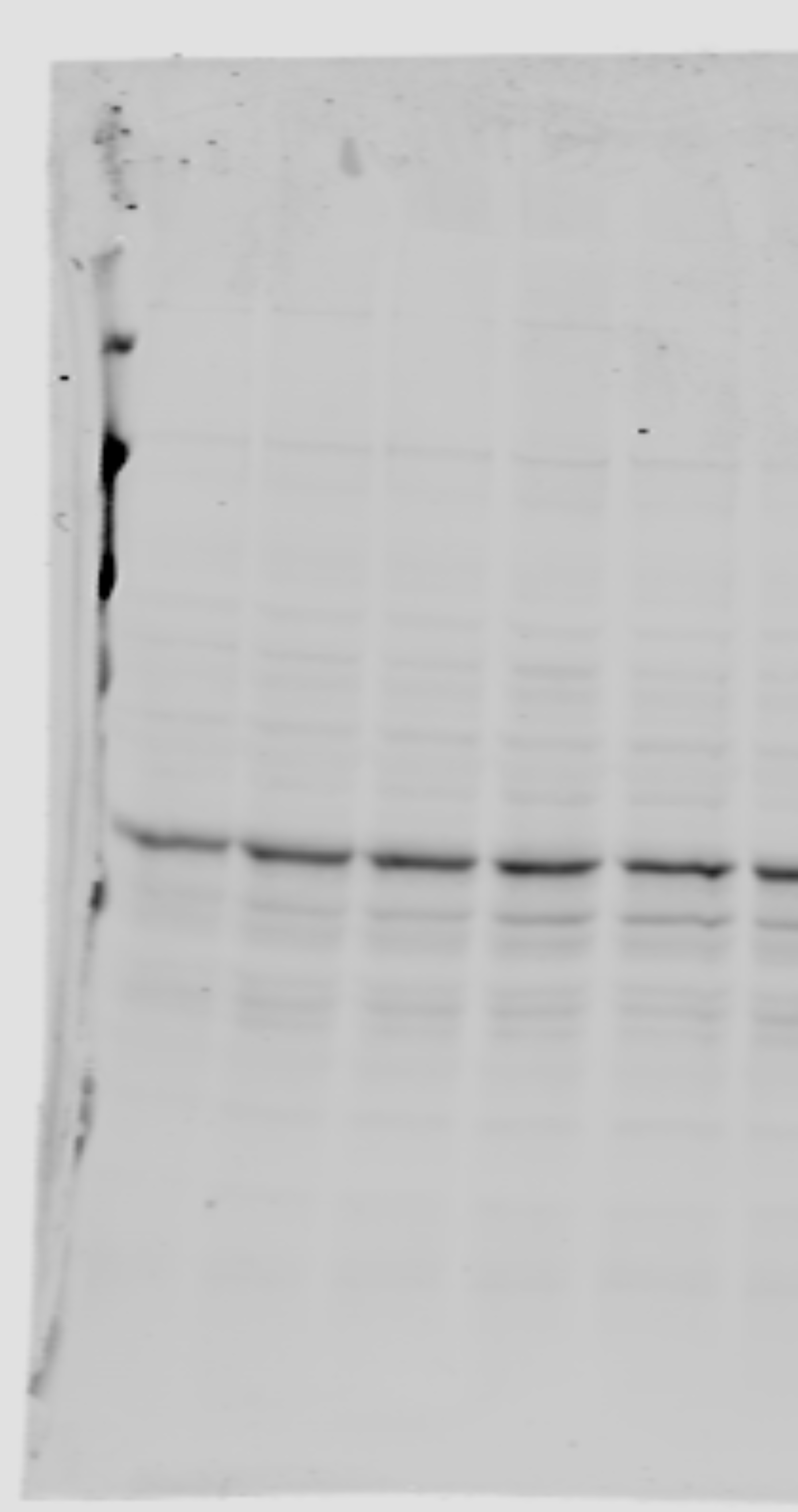

Supplement: Figure 1—figure supplement 2—source data 1. [file elife-82843-fig1-figsupp2-data1.zip › Fig. 1-2E SM.tif]

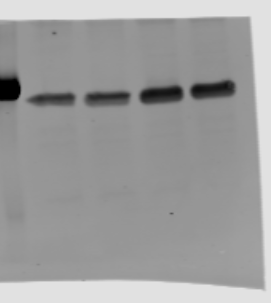

Supplement: Figure 1—figure supplement 2—source data 1. [file elife-82843-fig1-figsupp2-data1.zip › Fig. 1-2F-HCT116 GAPDH.tif]

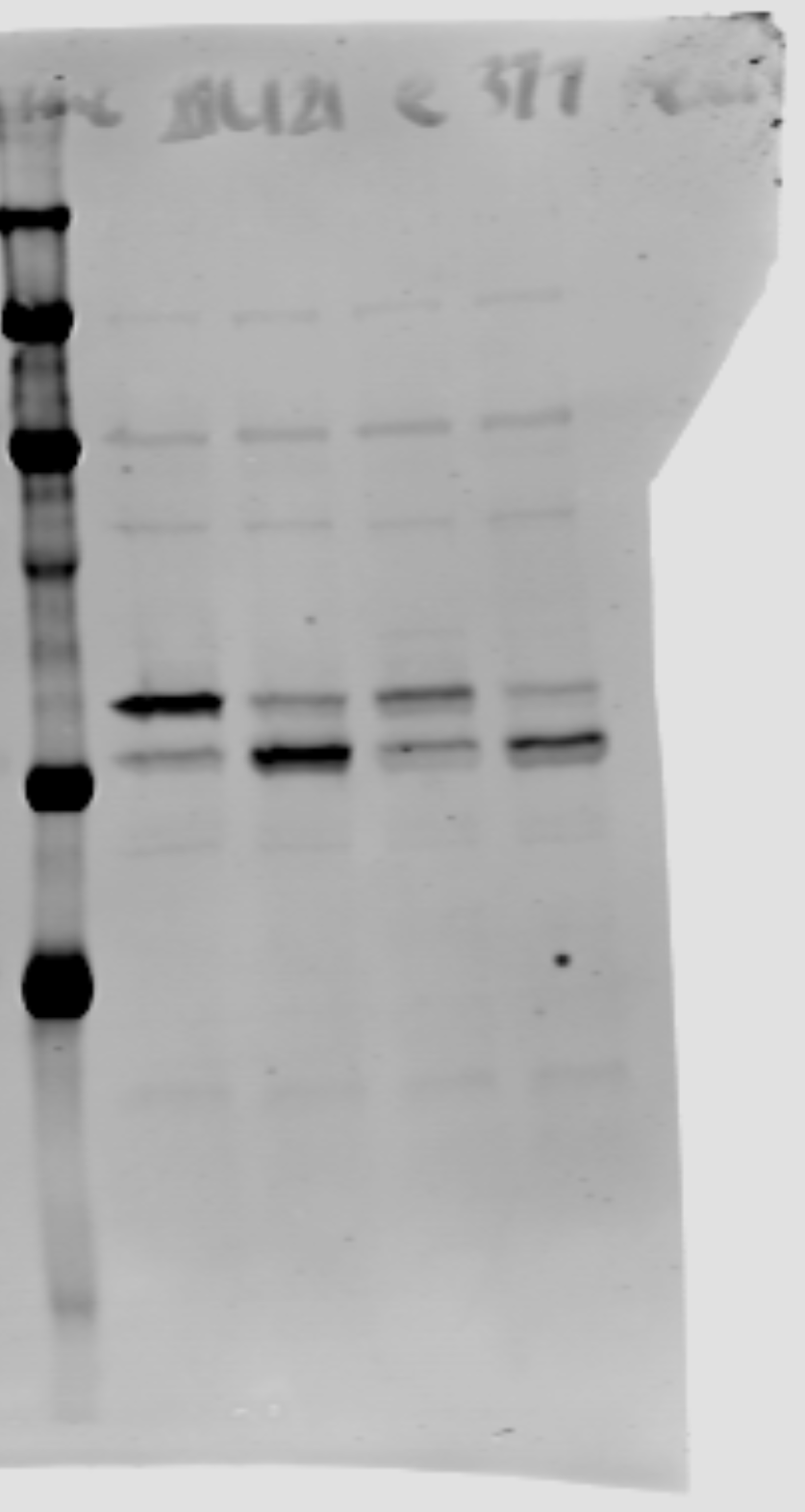

Supplement: Figure 1—figure supplement 2—source data 1. [file elife-82843-fig1-figsupp2-data1.zip › Fig. 1-2F-HCT116 SM.tif]

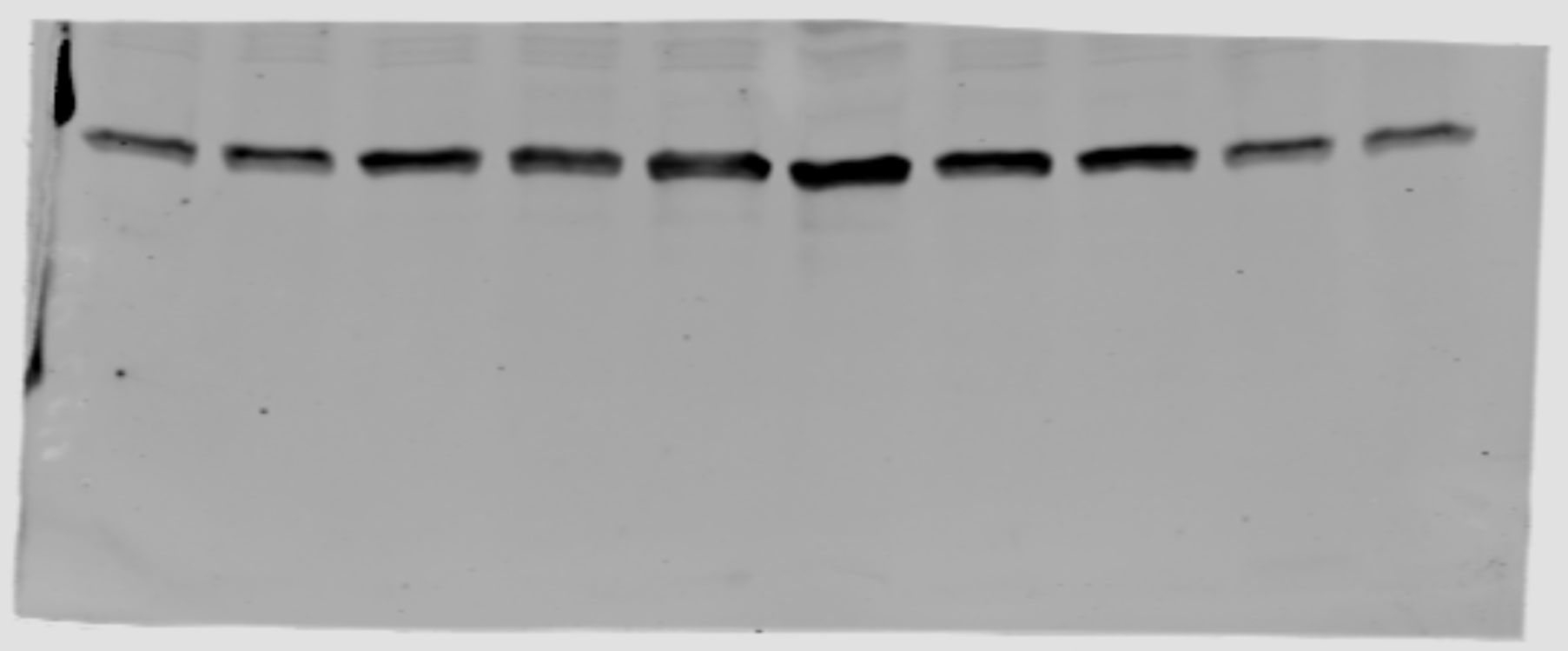

Supplement: Figure 1—figure supplement 2—source data 1. [file elife-82843-fig1-figsupp2-data1.zip › Fig. 1-2F-HeLa GAPDH.tif]

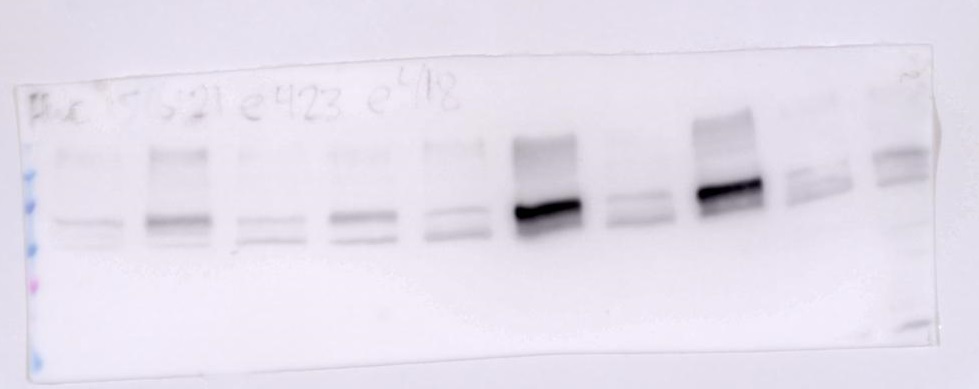

Supplement: Figure 1—figure supplement 2—source data 1. [file elife-82843-fig1-figsupp2-data1.zip › Fig. 1-2F-HeLa HIF1a.jpg]

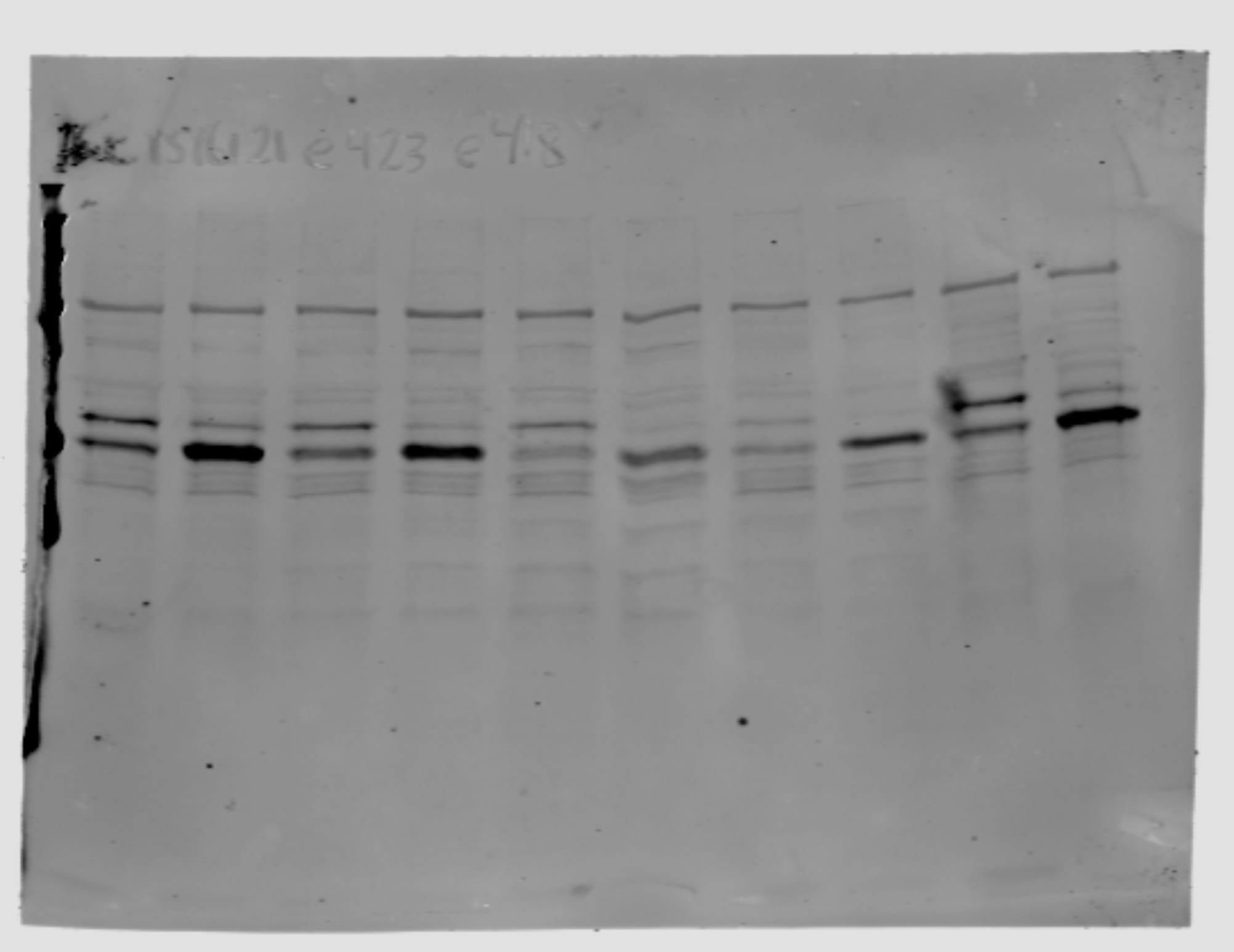

Supplement: Figure 1—figure supplement 2—source data 1. [file elife-82843-fig1-figsupp2-data1.zip › Fig. 1-2F-HeLa SM.tif]

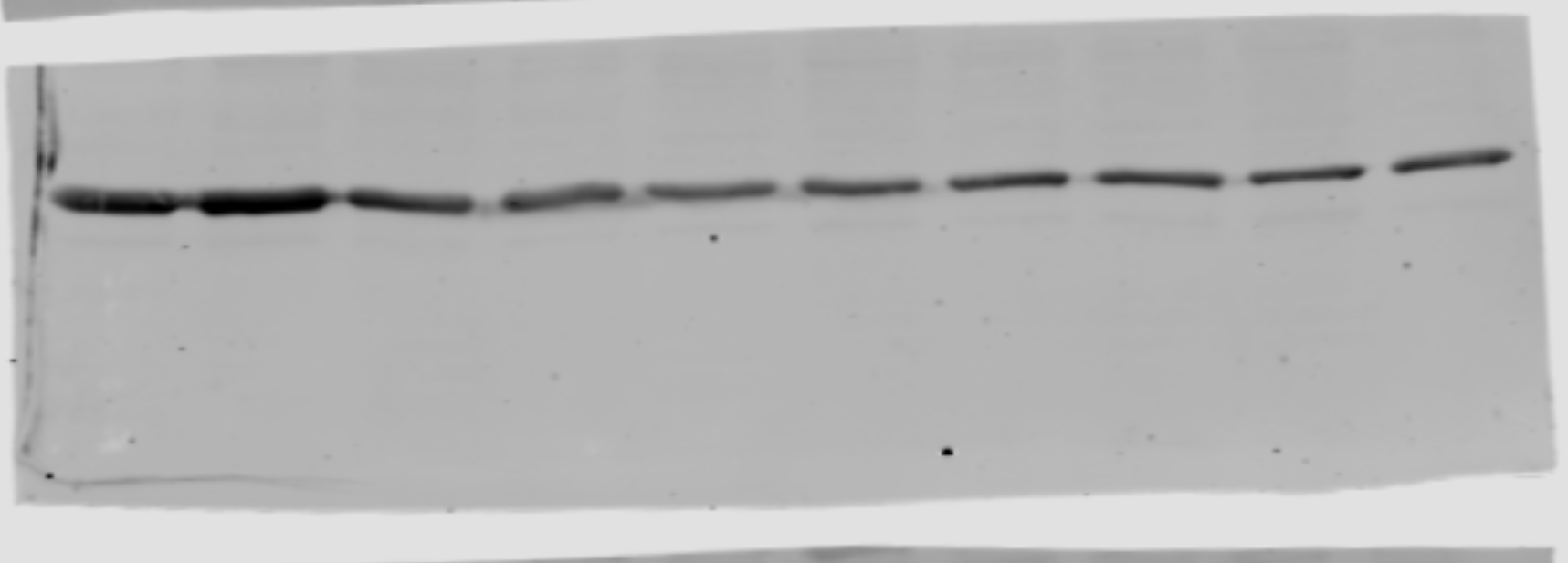

Supplement: Figure 1—figure supplement 2—source data 1. [file elife-82843-fig1-figsupp2-data1.zip › Fig. 1-2F-Huh7 GAPDH.tif]

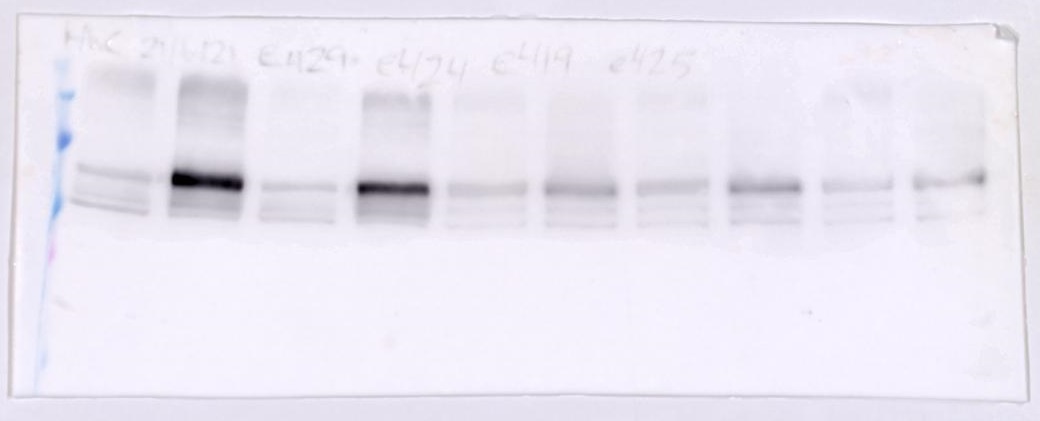

Supplement: Figure 1—figure supplement 2—source data 1. [file elife-82843-fig1-figsupp2-data1.zip › Fig. 1-2F-Huh7 HIF1a.jpg]

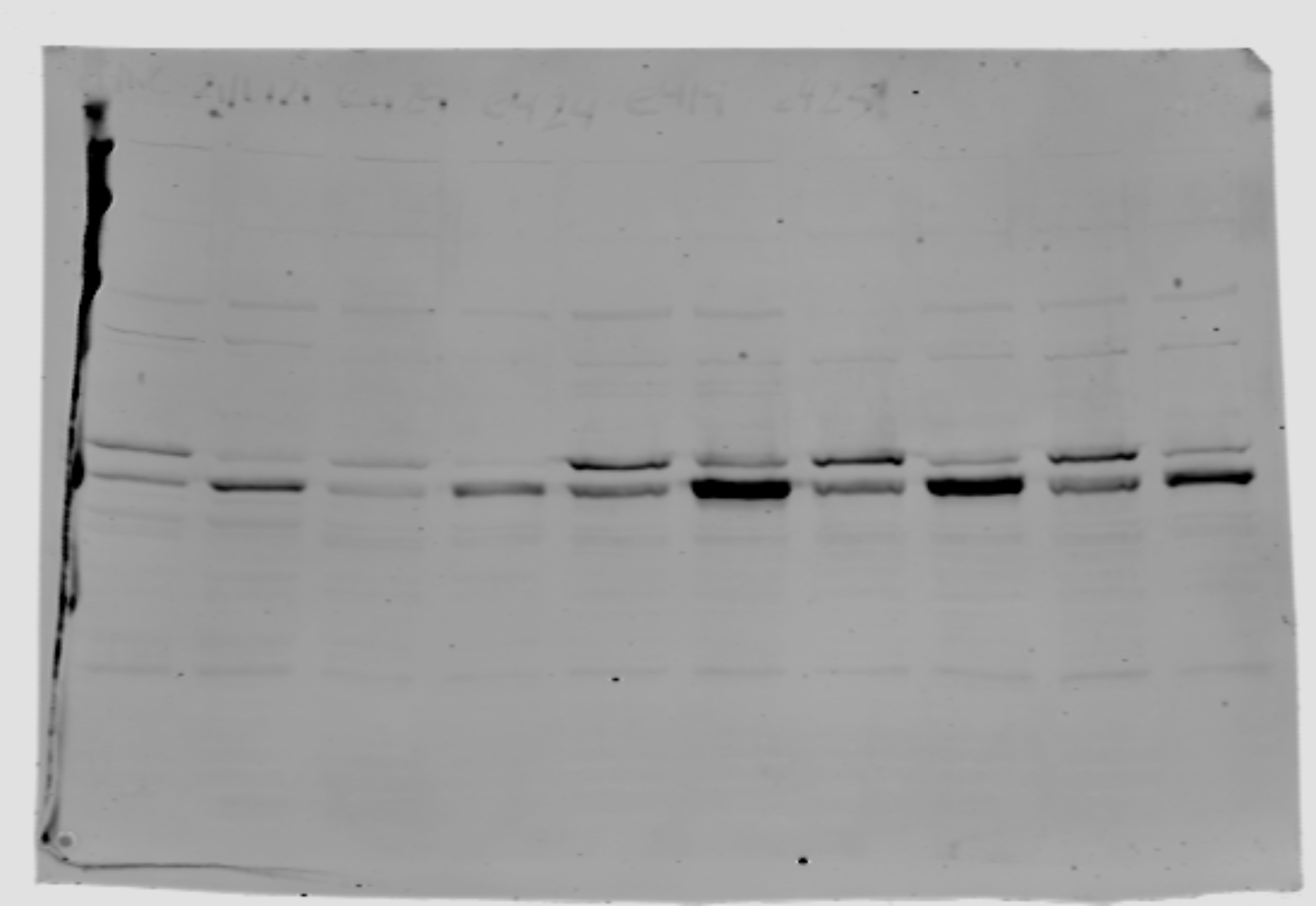

Supplement: Figure 1—figure supplement 2—source data 1. [file elife-82843-fig1-figsupp2-data1.zip › Fig. 1-2F-Huh7 SM.tif]

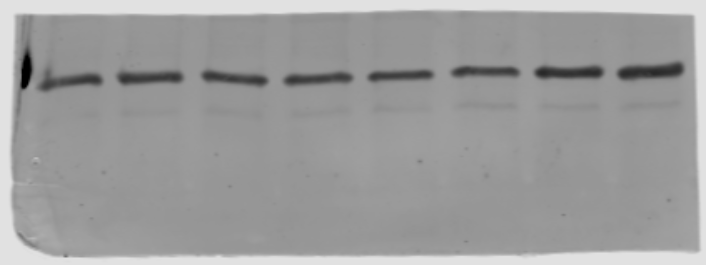

Supplement: Figure 1—figure supplement 2—source data 1. [file elife-82843-fig1-figsupp2-data1.zip › Fig. 1-2F-MDA-MB-231 GAPDH.tif]

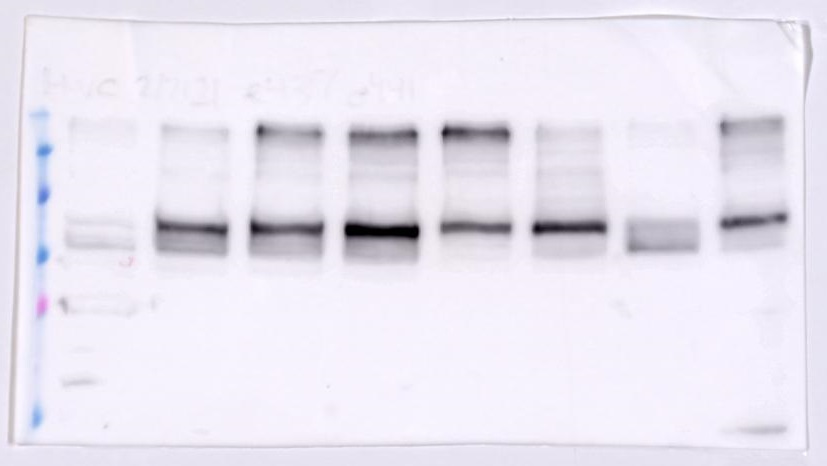

Supplement: Figure 1—figure supplement 2—source data 1. [file elife-82843-fig1-figsupp2-data1.zip › Fig. 1-2F-MDA-MB-231 HIF1a.jpg]

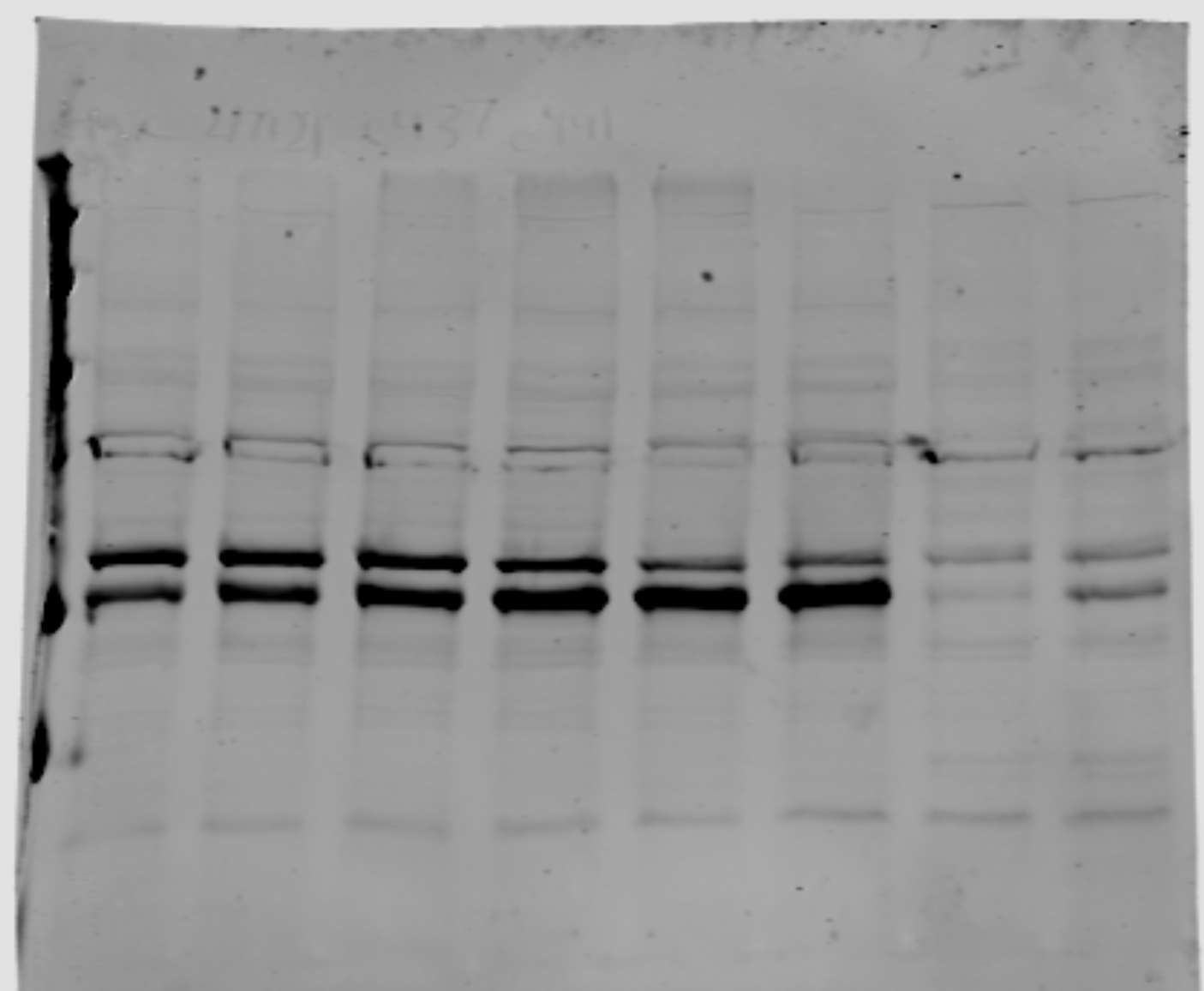

Supplement: Figure 1—figure supplement 2—source data 1. [file elife-82843-fig1-figsupp2-data1.zip › Fig. 1-2F-MDA-MB-231 SM.tif]

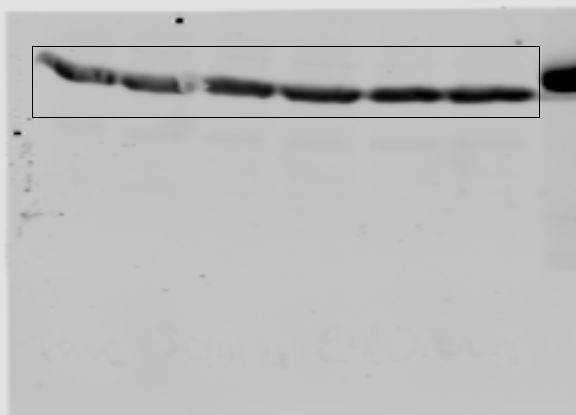

Supplement: Figure 1—figure supplement 3—source data 1. [file elife-82843-fig1-figsupp3-data1.zip › Annotated/Fig. 1-3B GAPDH.tif]

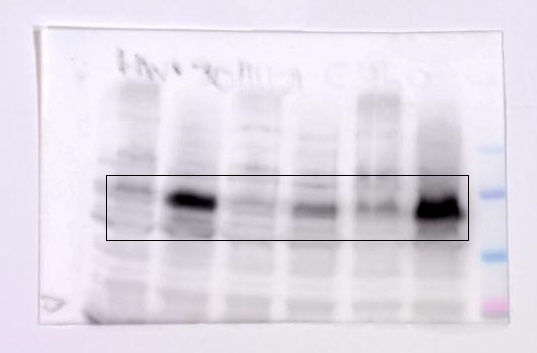

Supplement: Figure 1—figure supplement 3—source data 1. [file elife-82843-fig1-figsupp3-data1.zip › Annotated/Fig. 1-3B HIF1a.jpg]

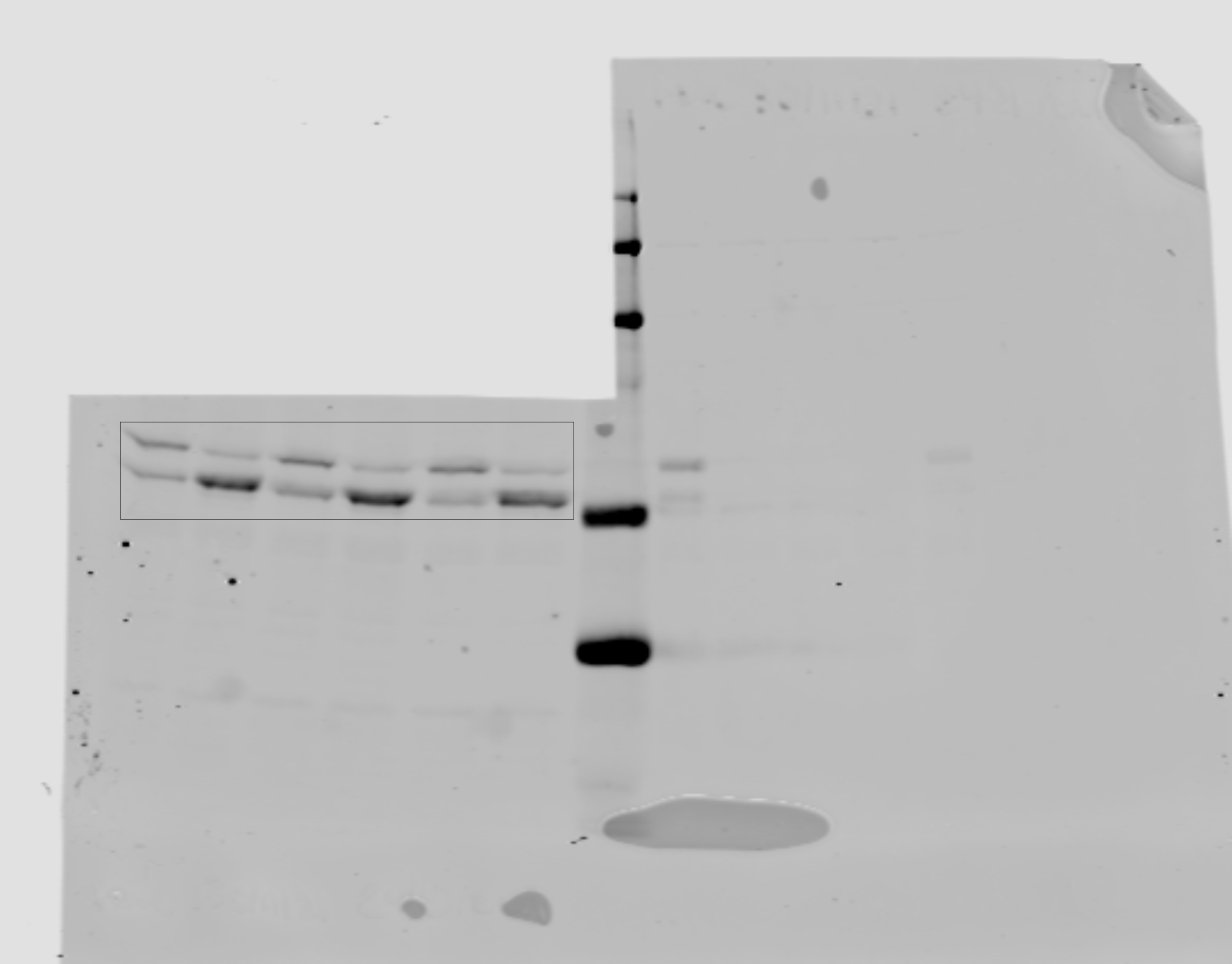

Supplement: Figure 1—figure supplement 3—source data 1. [file elife-82843-fig1-figsupp3-data1.zip › Annotated/Fig. 1-3B SM.tif]

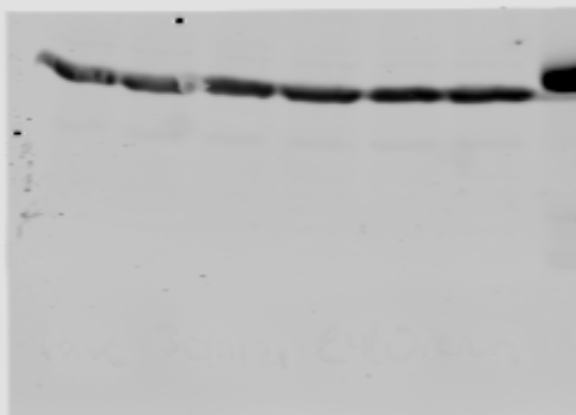

Supplement: Figure 1—figure supplement 3—source data 1. [file elife-82843-fig1-figsupp3-data1.zip › Fig. 1-3B GAPDH.tif]

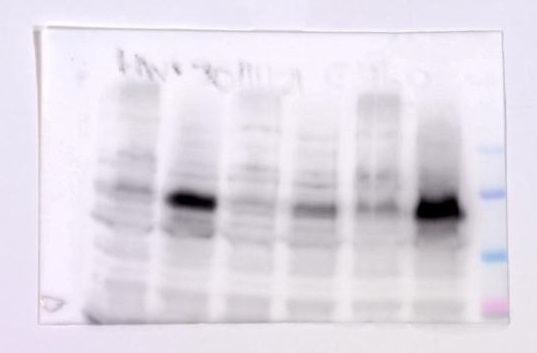

Supplement: Figure 1—figure supplement 3—source data 1. [file elife-82843-fig1-figsupp3-data1.zip › Fig. 1-3B HIF1a.jpg]

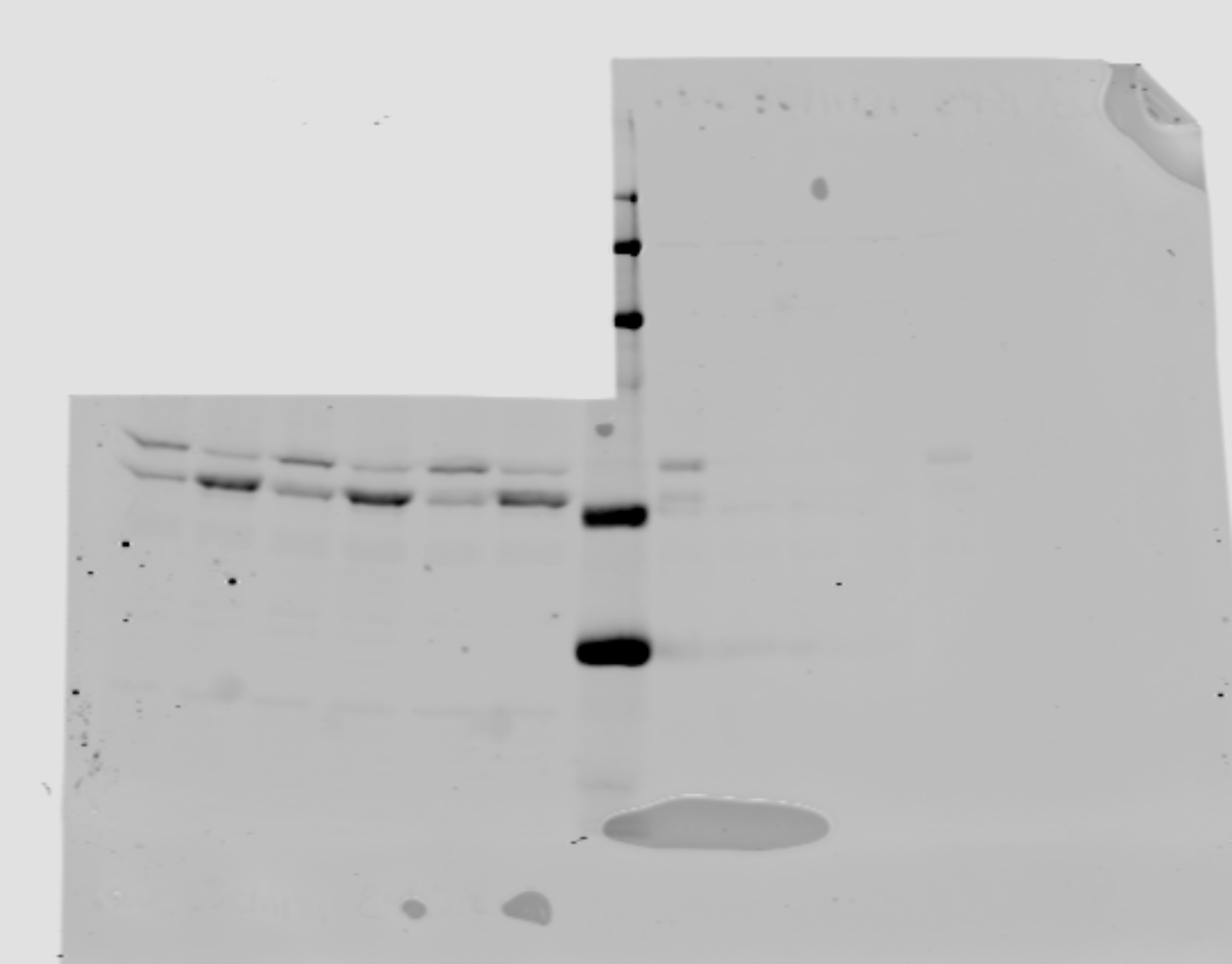

Supplement: Figure 1—figure supplement 3—source data 1. [file elife-82843-fig1-figsupp3-data1.zip › Fig. 1-3B SM.tif]

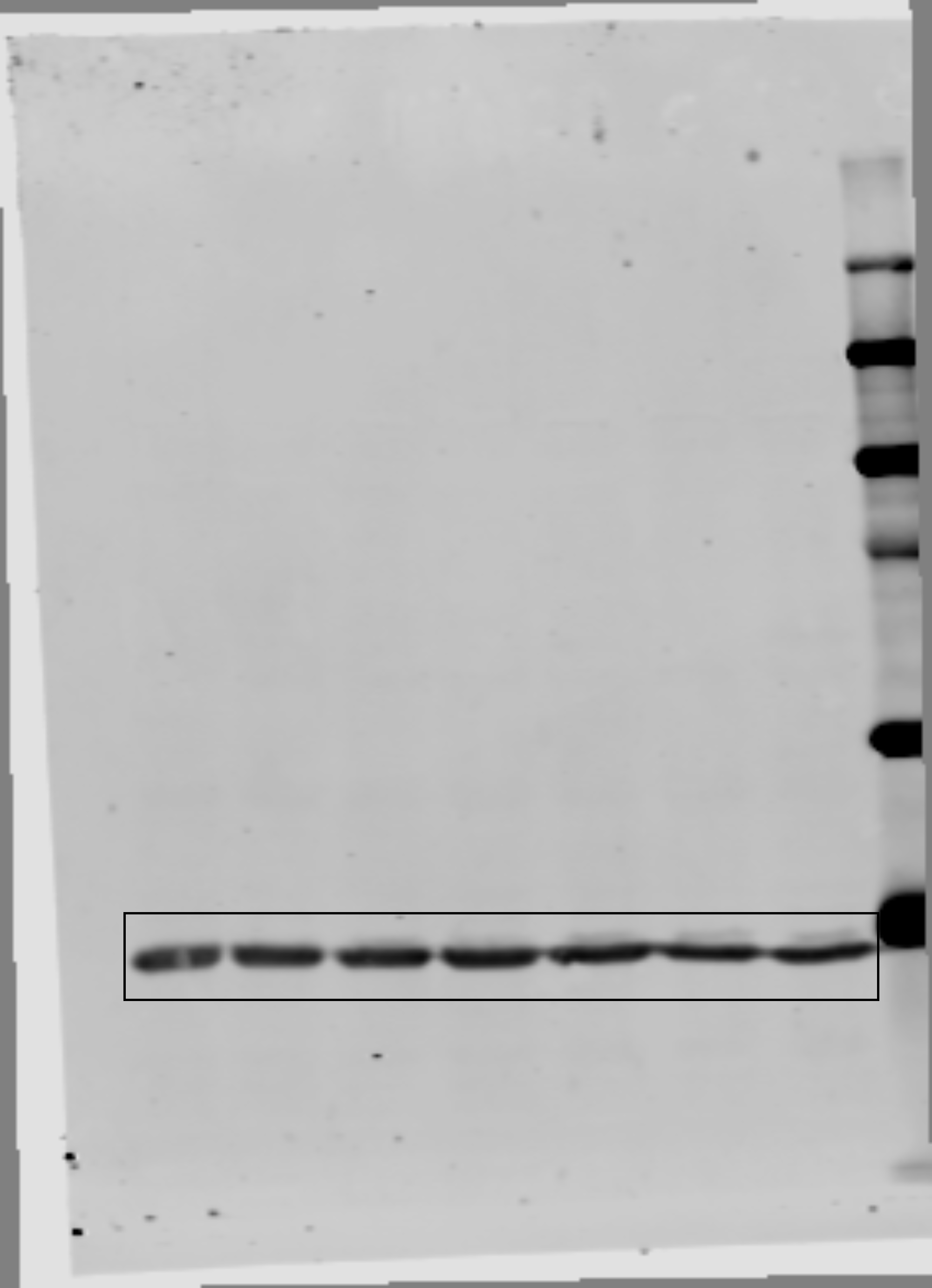

Supplement: Figure 2—source data 1. [file elife-82843-fig2-data1.zip › Annotated/Fig. 2B GAPDH.tif]

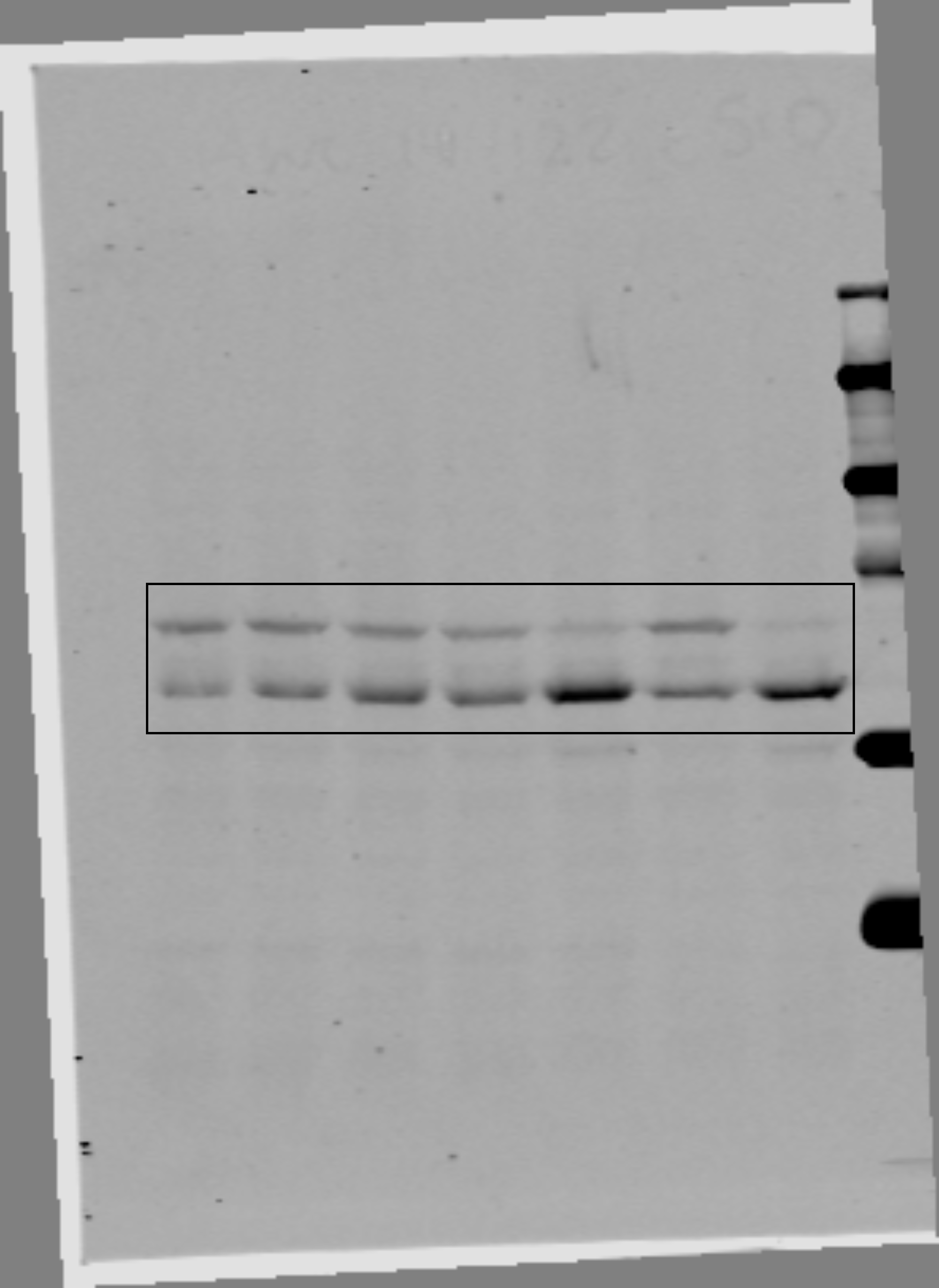

Supplement: Figure 2—source data 1. [file elife-82843-fig2-data1.zip › Annotated/Fig. 2B V5.tif]

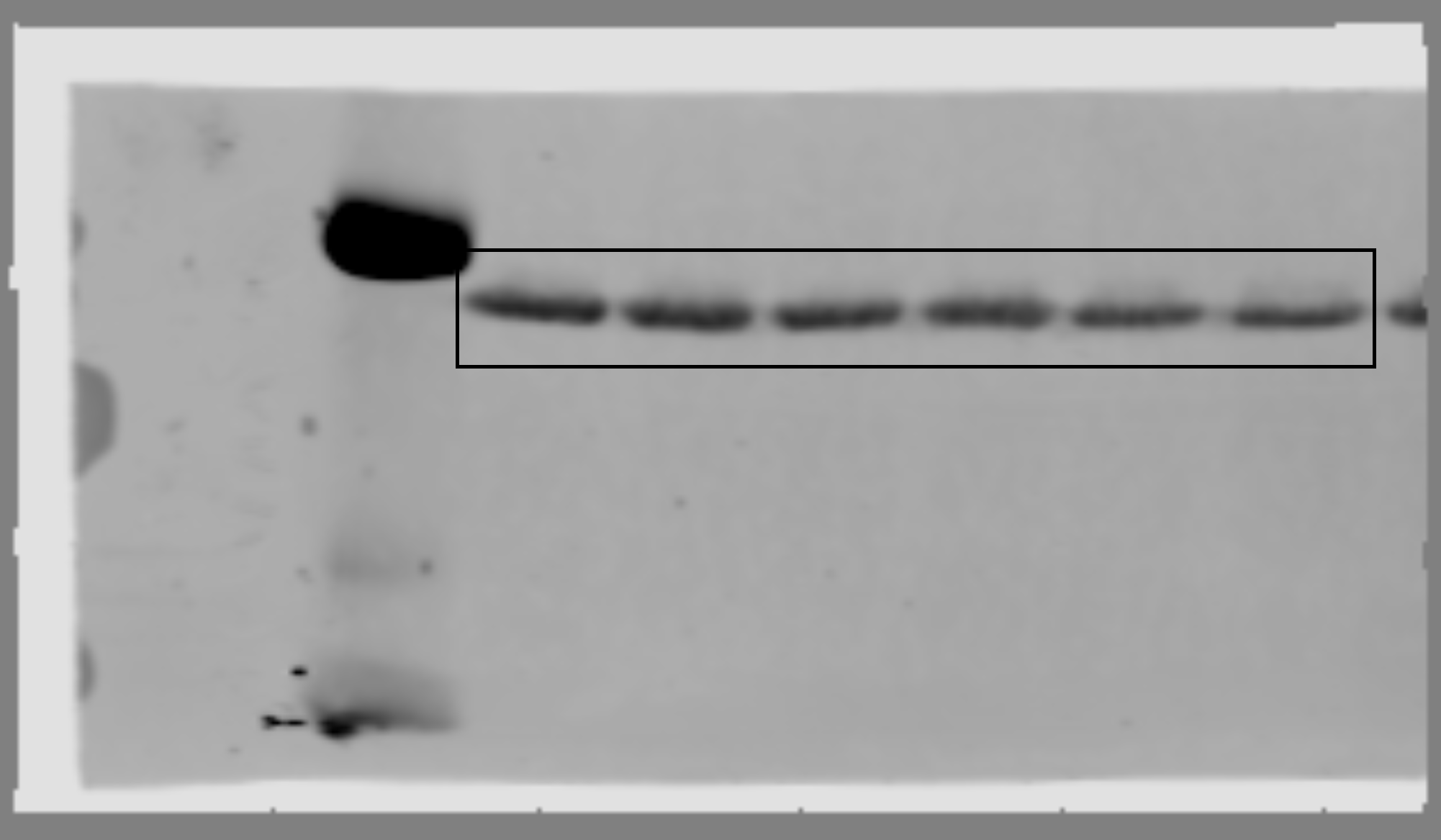

Supplement: Figure 2—source data 1. [file elife-82843-fig2-data1.zip › Annotated/Fig. 2C GAPDH.tif]

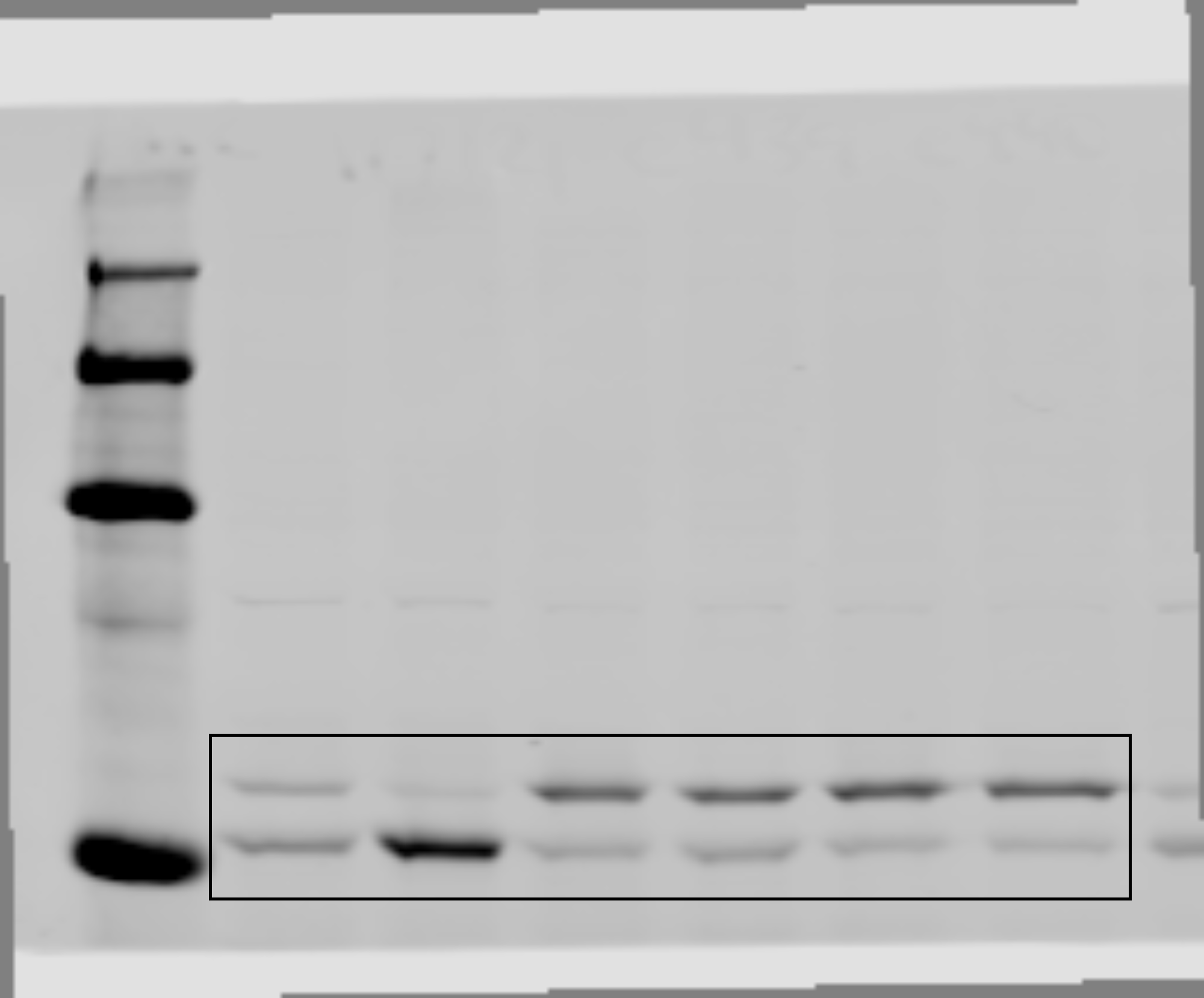

Supplement: Figure 2—source data 1. [file elife-82843-fig2-data1.zip › Annotated/Fig. 2C SM.tif]

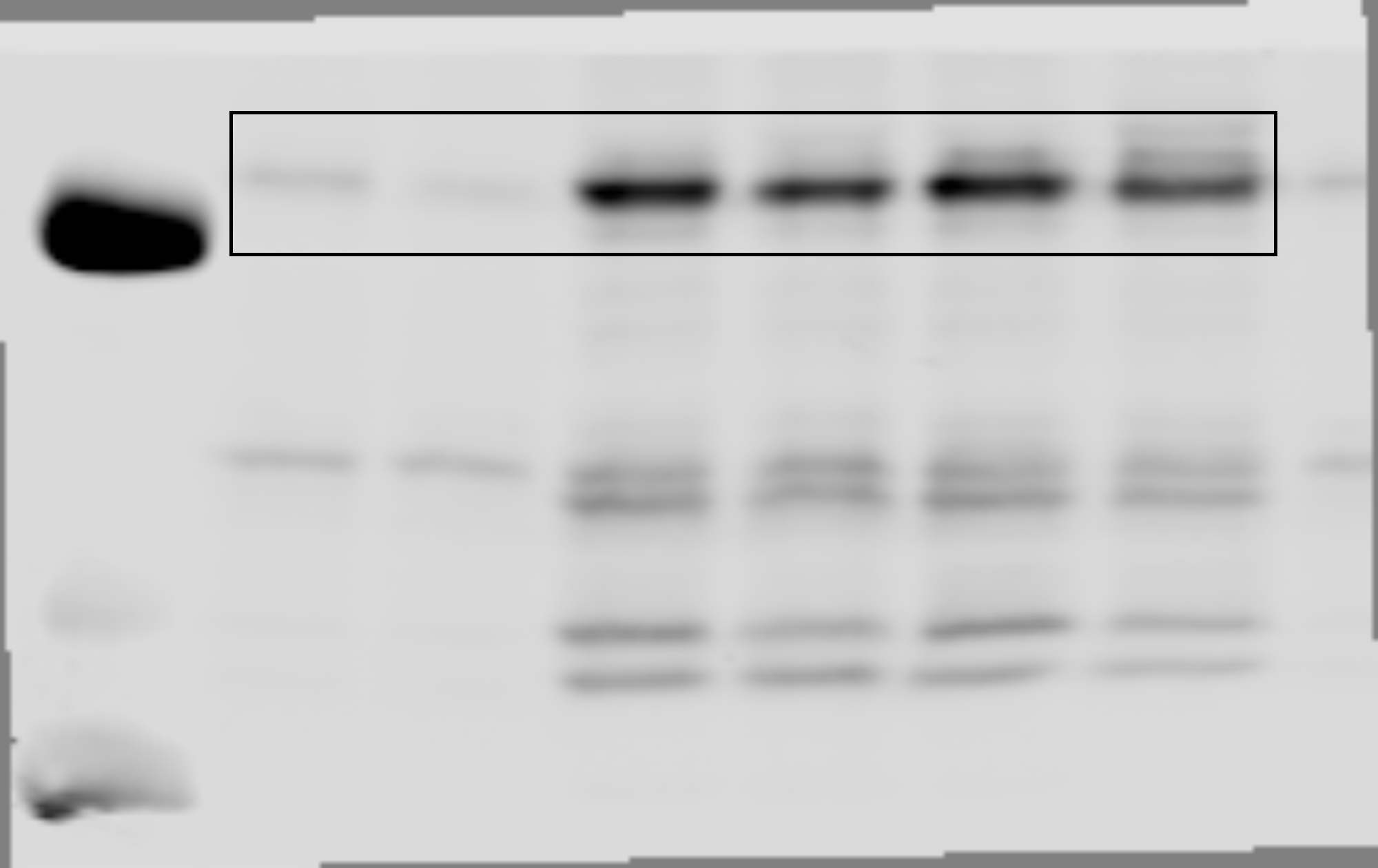

Supplement: Figure 2—source data 1. [file elife-82843-fig2-data1.zip › Annotated/Fig. 2C V5.tif]

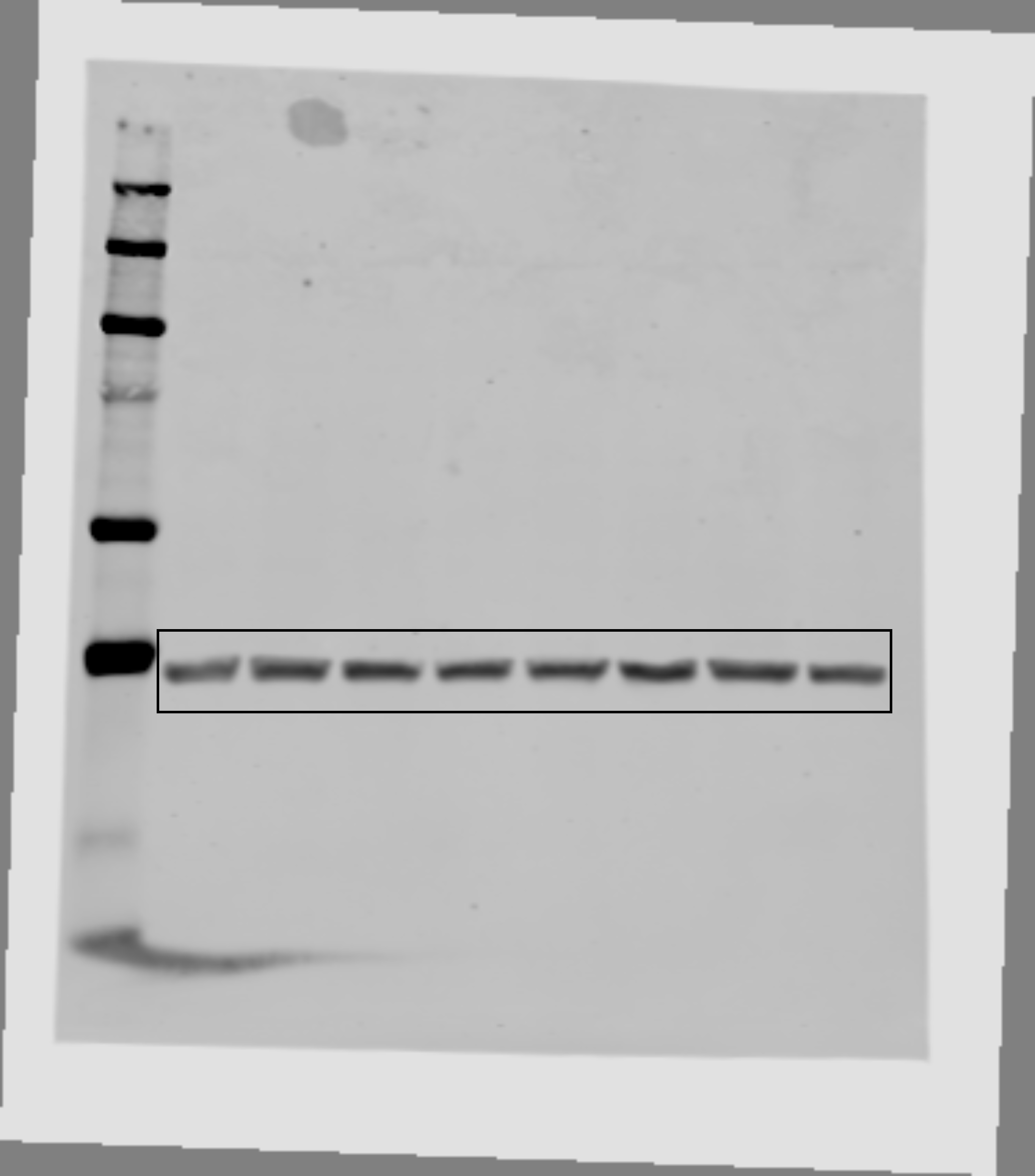

Supplement: Figure 2—source data 1. [file elife-82843-fig2-data1.zip › Annotated/Fig. 2D GAPDH.tif]

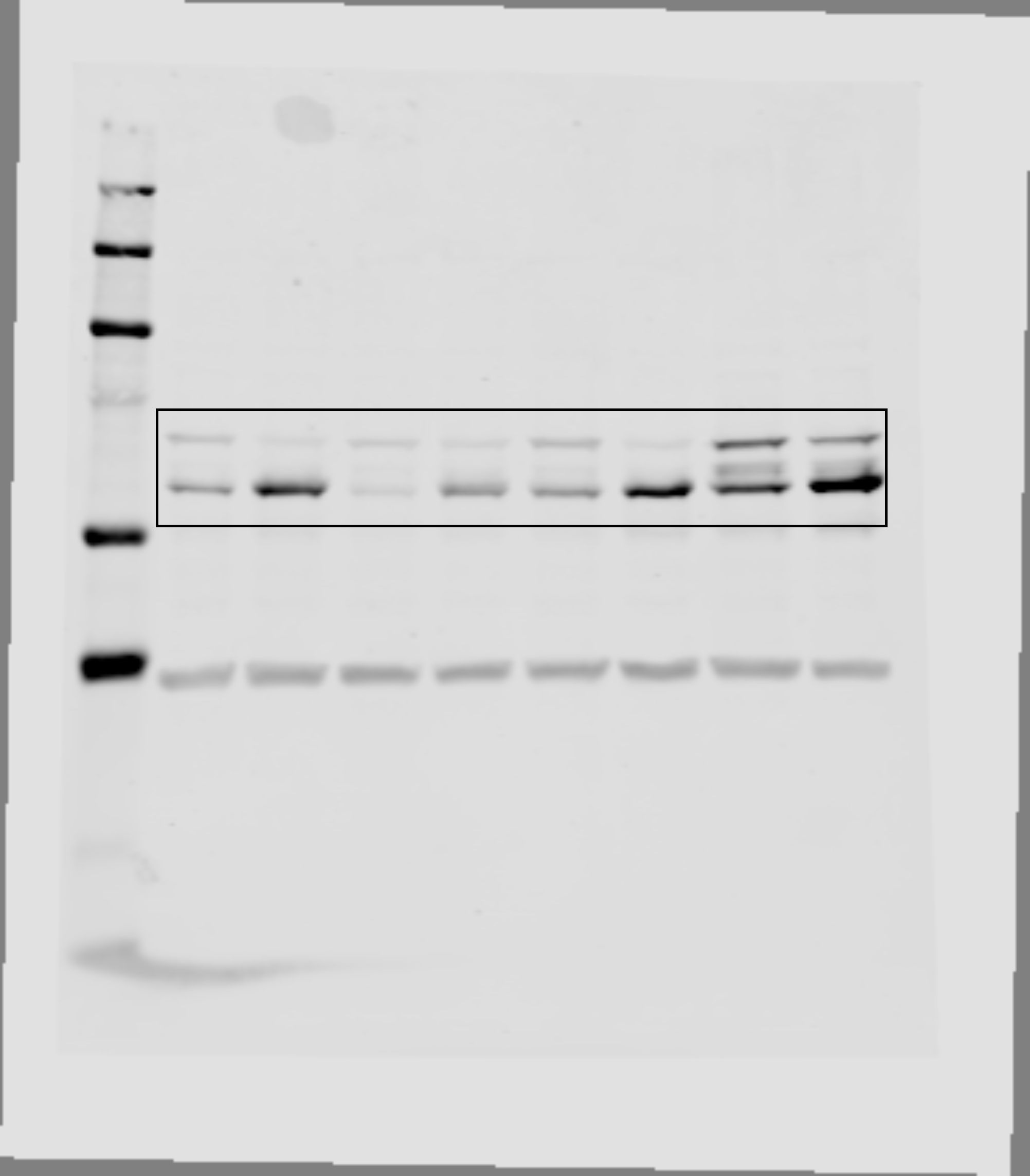

Supplement: Figure 2—source data 1. [file elife-82843-fig2-data1.zip › Annotated/Fig. 2D V5.tif]

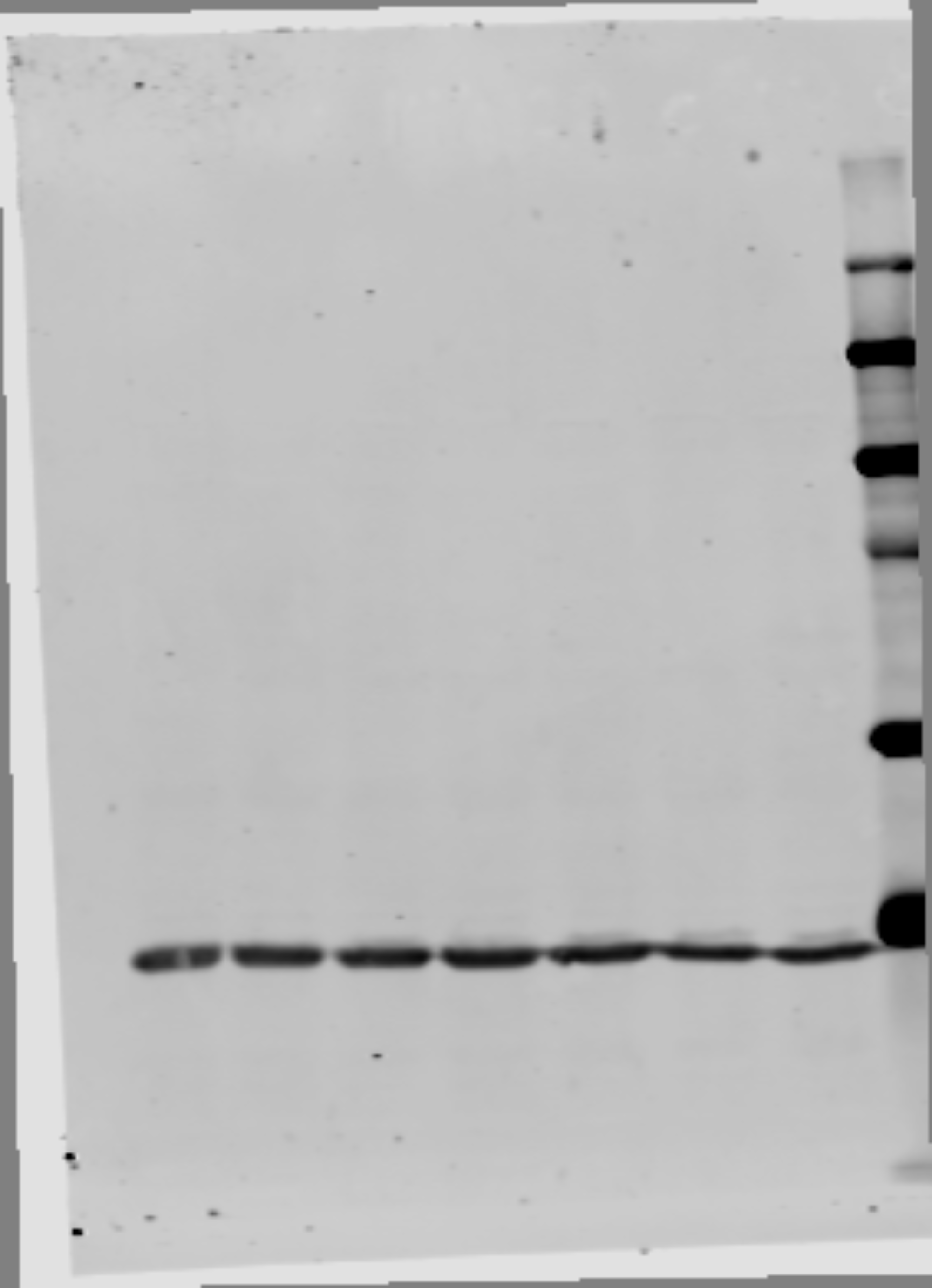

Supplement: Figure 2—source data 1. [file elife-82843-fig2-data1.zip › Fig. 2B GAPDH.tif]

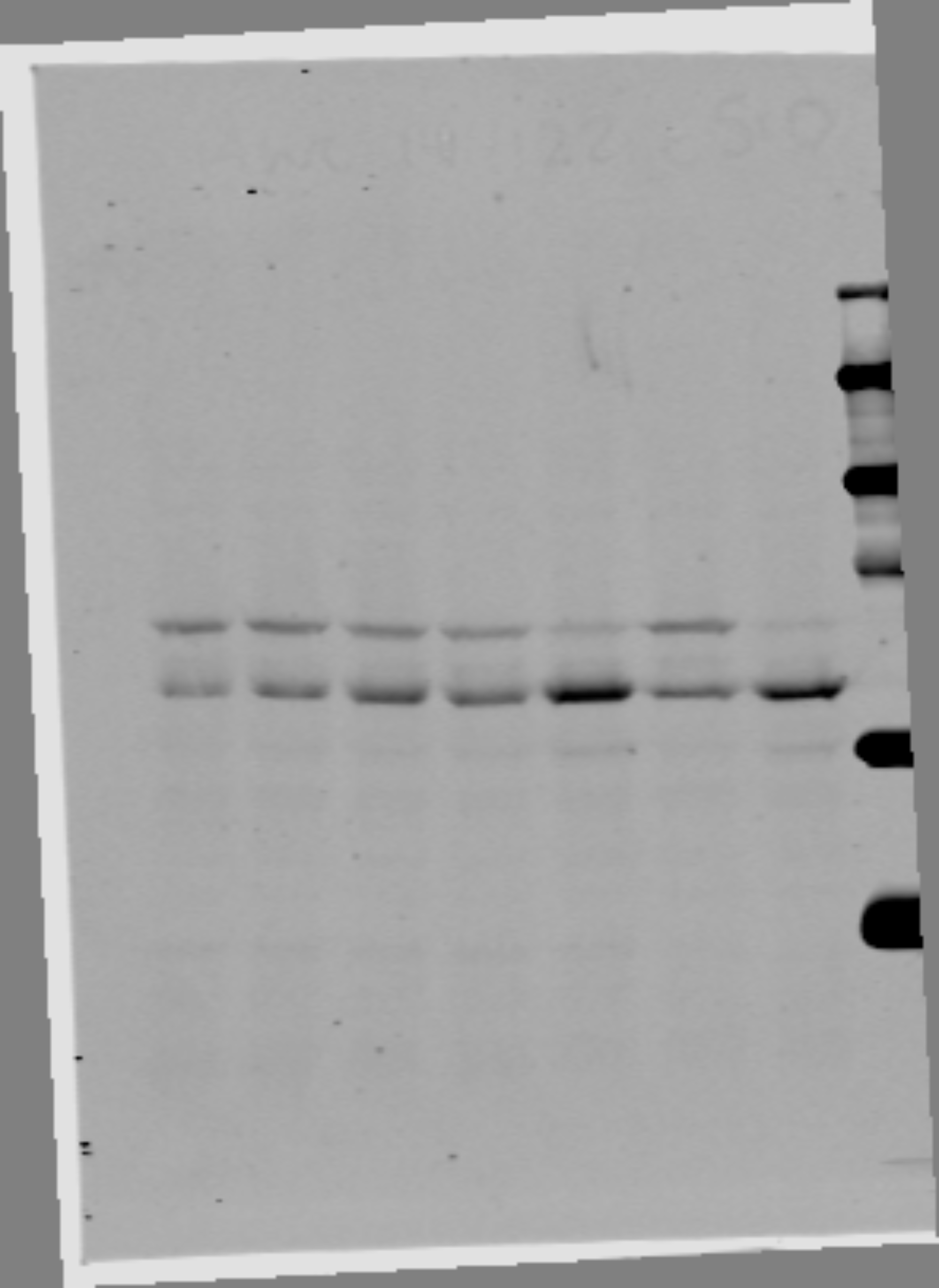

Supplement: Figure 2—source data 1. [file elife-82843-fig2-data1.zip › Fig. 2B V5.tif]

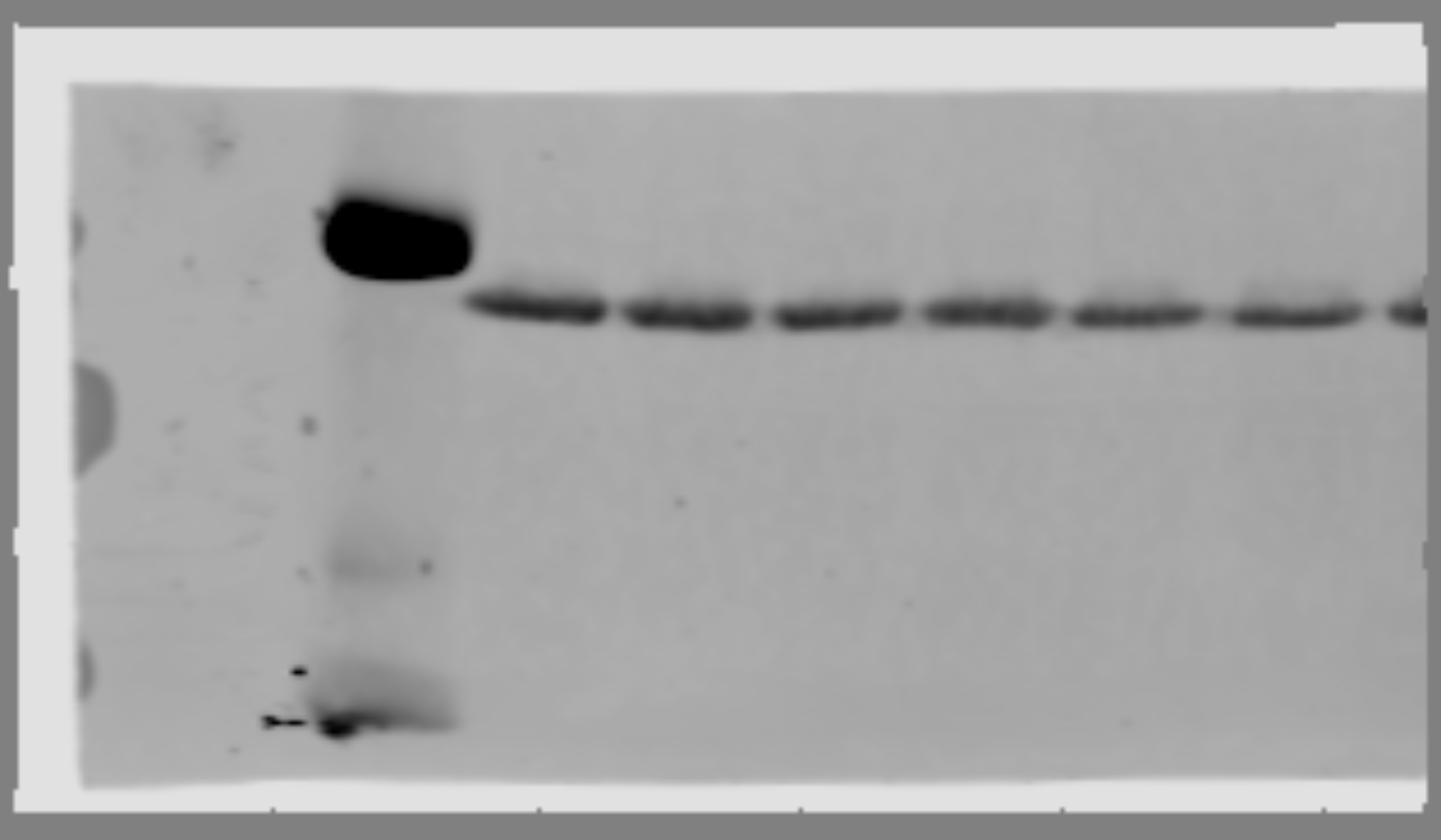

Supplement: Figure 2—source data 1. [file elife-82843-fig2-data1.zip › Fig. 2C GAPDH.tif]

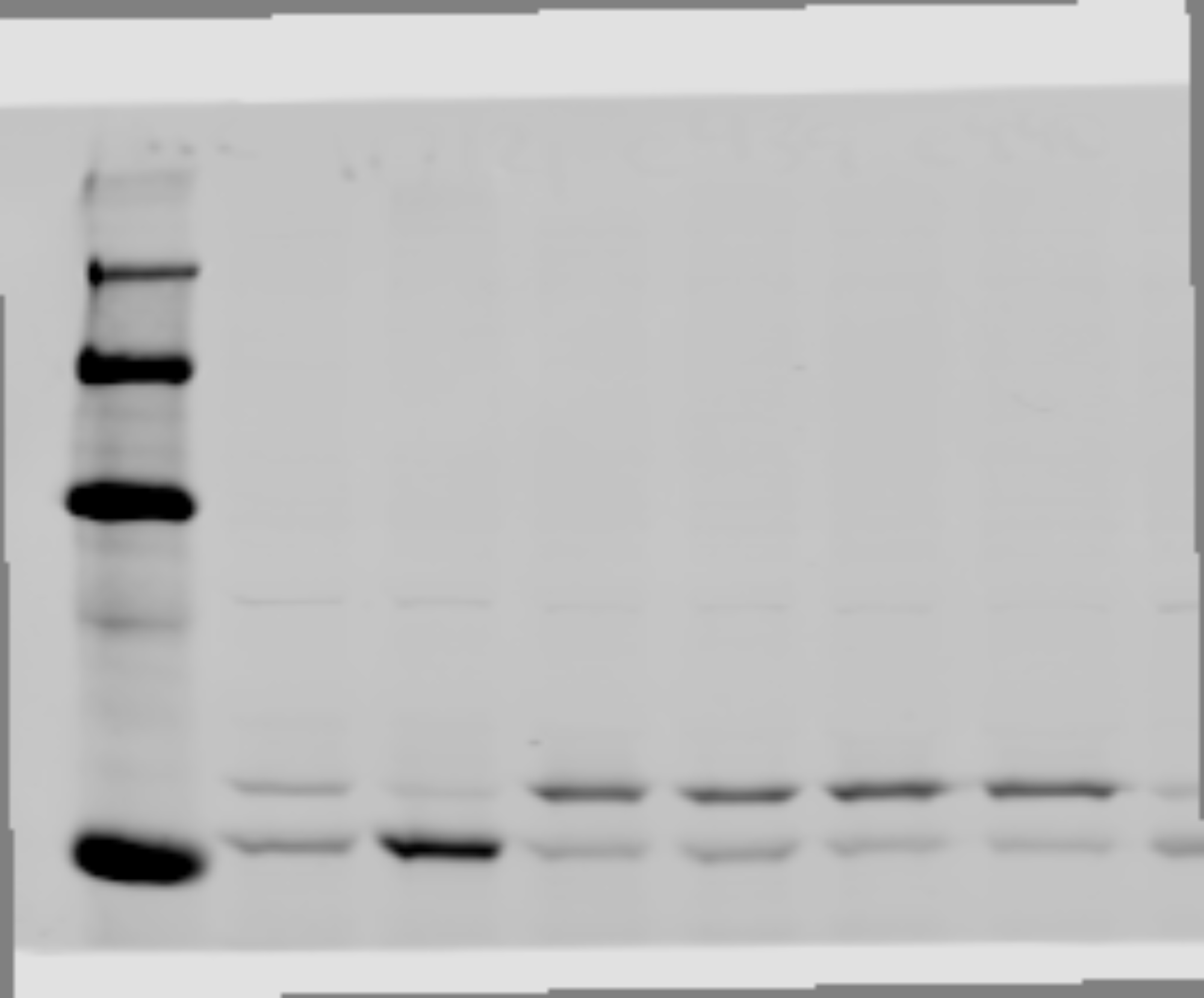

Supplement: Figure 2—source data 1. [file elife-82843-fig2-data1.zip › Fig. 2C SM.tif]
