# Supplementary material for: Hypoxia truncates and constitutively activates the key cholesterol synthesis enzyme squalene monooxygenase
Source: eLife. 2023 Jan 19;12:e82843. doi: 10.7554/eLife.82843 (PMC9851614; doi:10.7554/eLife.82843)

Figure 1—figure supplement 2E – SM

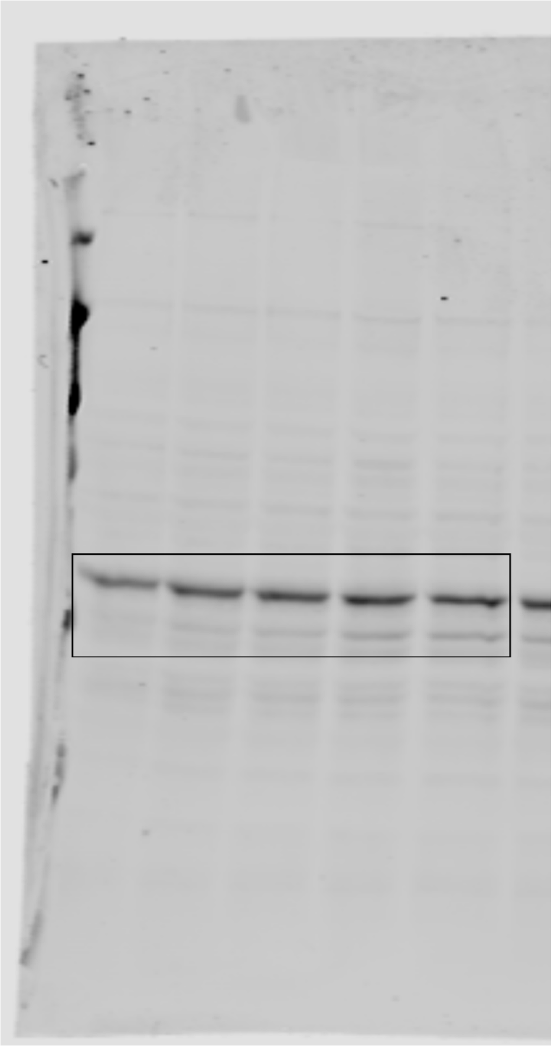

Figure 1—figure supplement 2E – HIF1α

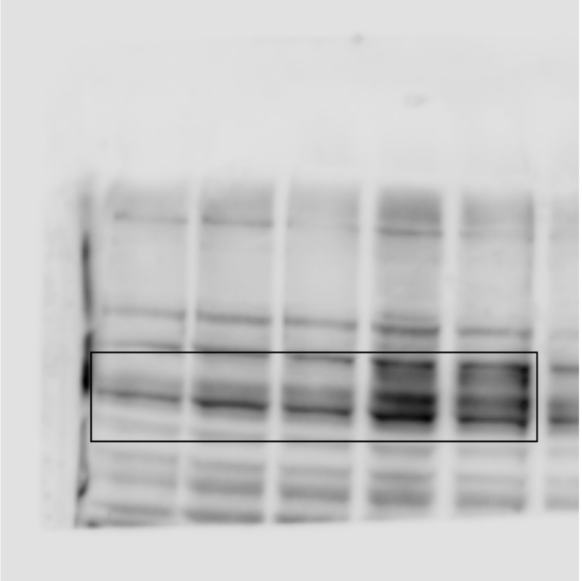

Figure 1—figure supplement 2E – GAPDH

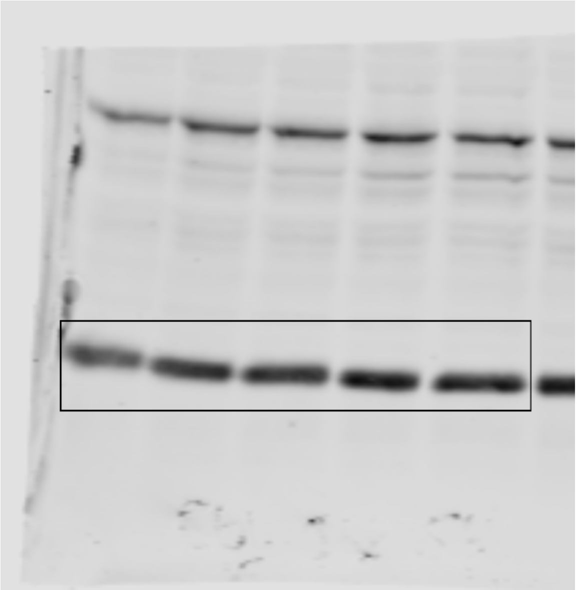

Figure 1—figure supplement 2F – HCT116 – SM

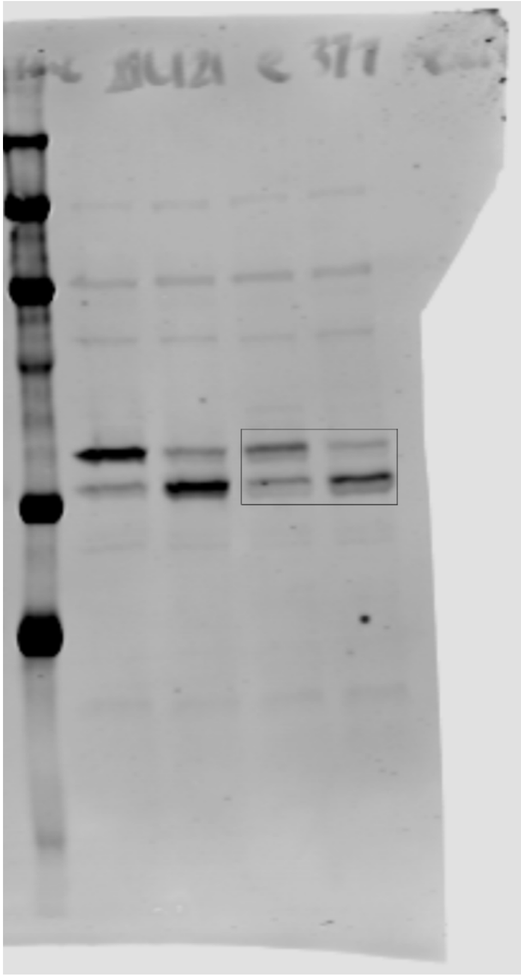

Figure 1—figure supplement 2F – HCT116 – HIF1α

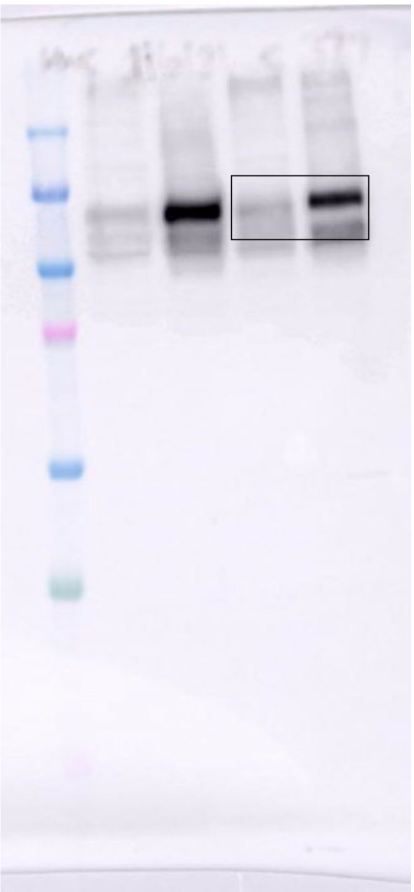

Figure 1—figure supplement 2F – HCT116 – GAPDH

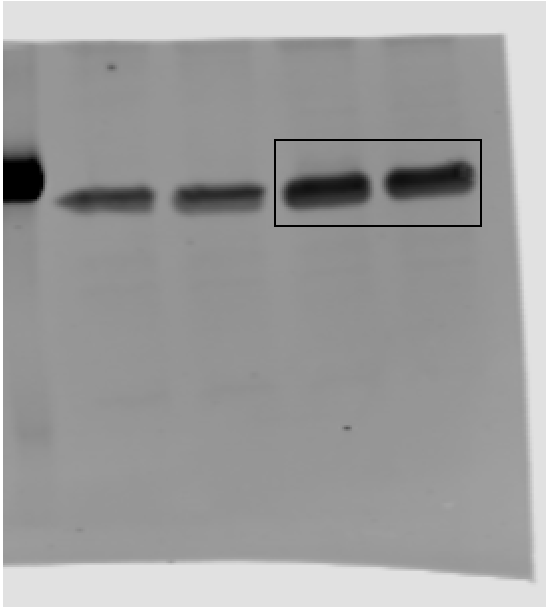

Figure 1—figure supplement 2F – Huh7 – SM

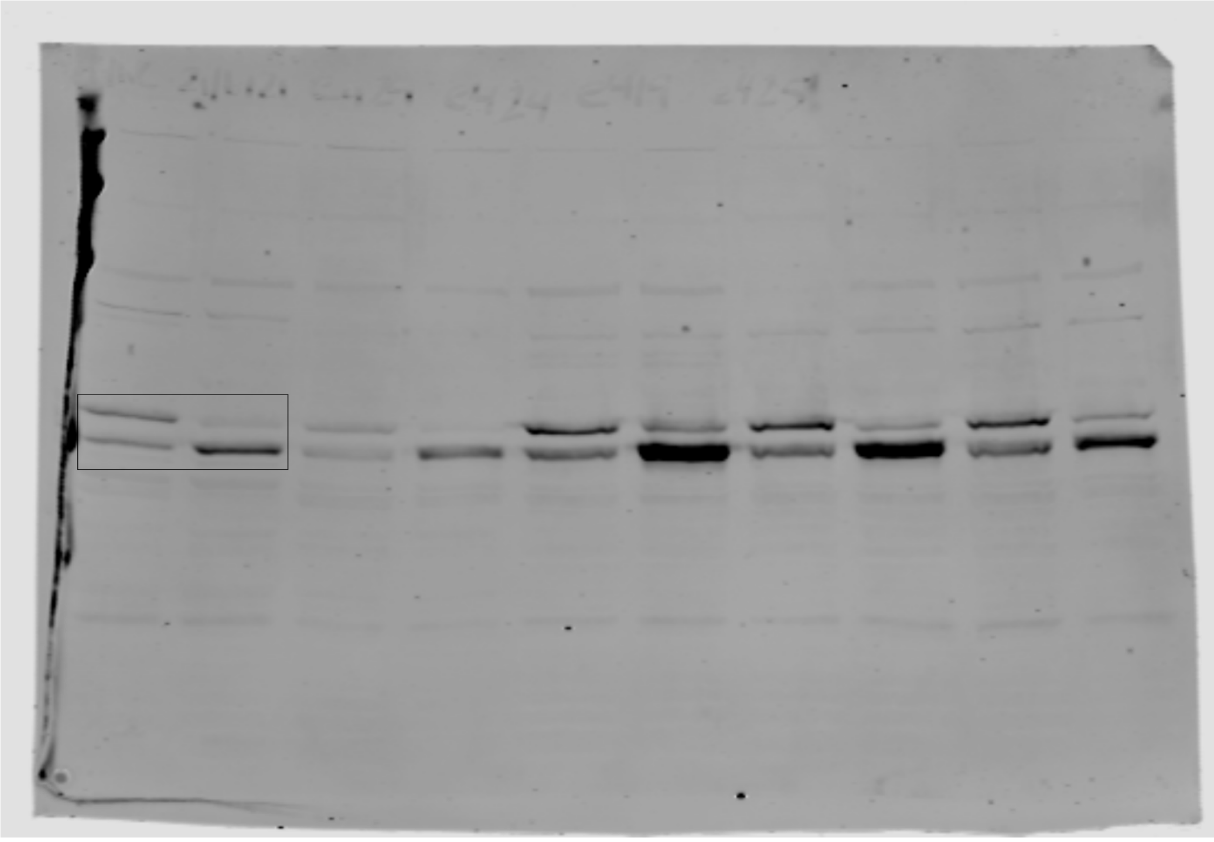

Figure 1—figure supplement 2F – Huh7 – HIF1α

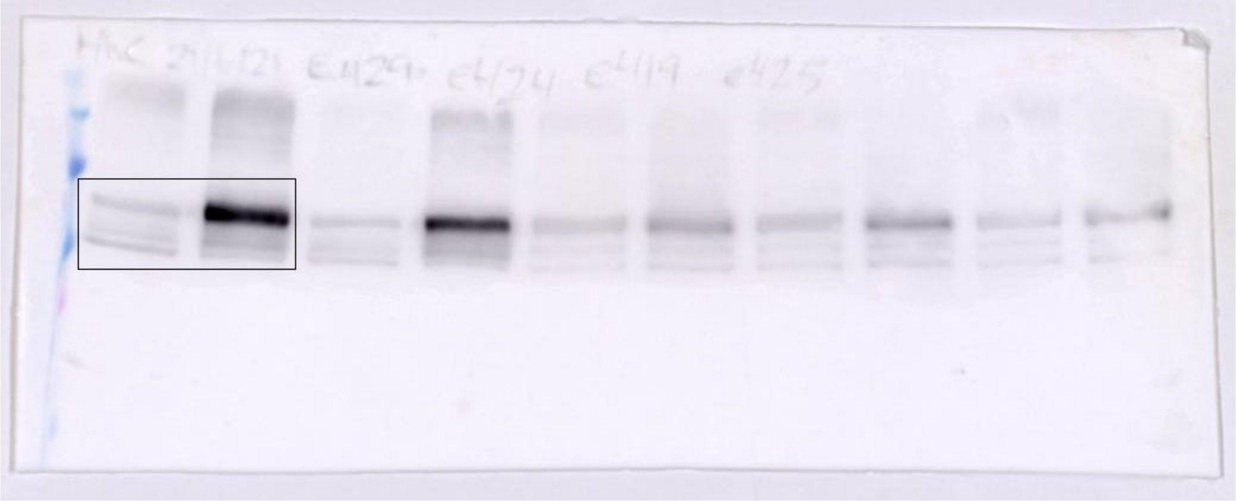

Figure 1—figure supplement 2F – Huh7 – GAPDH

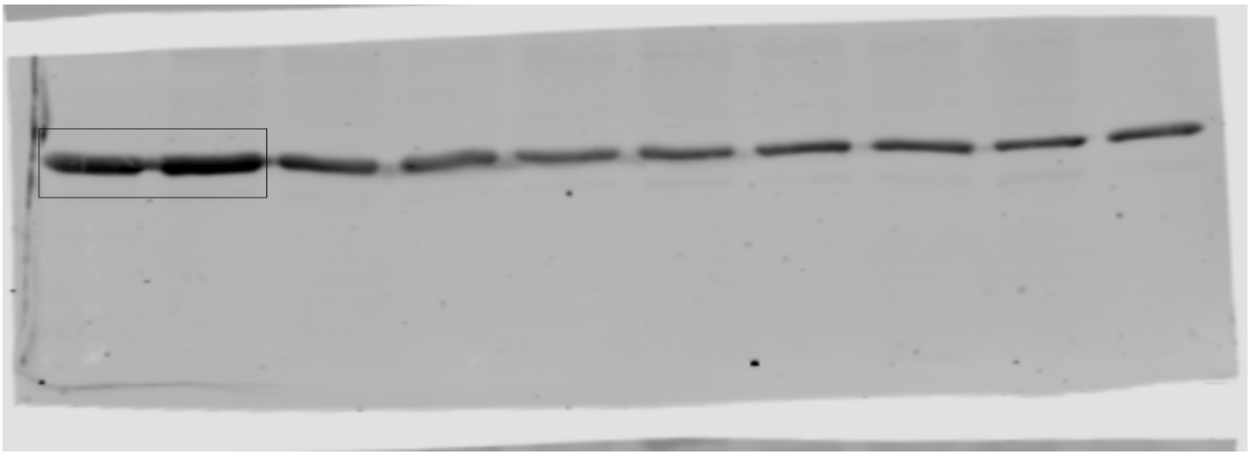

Figure 1—figure supplement 2F – HeLa – SM

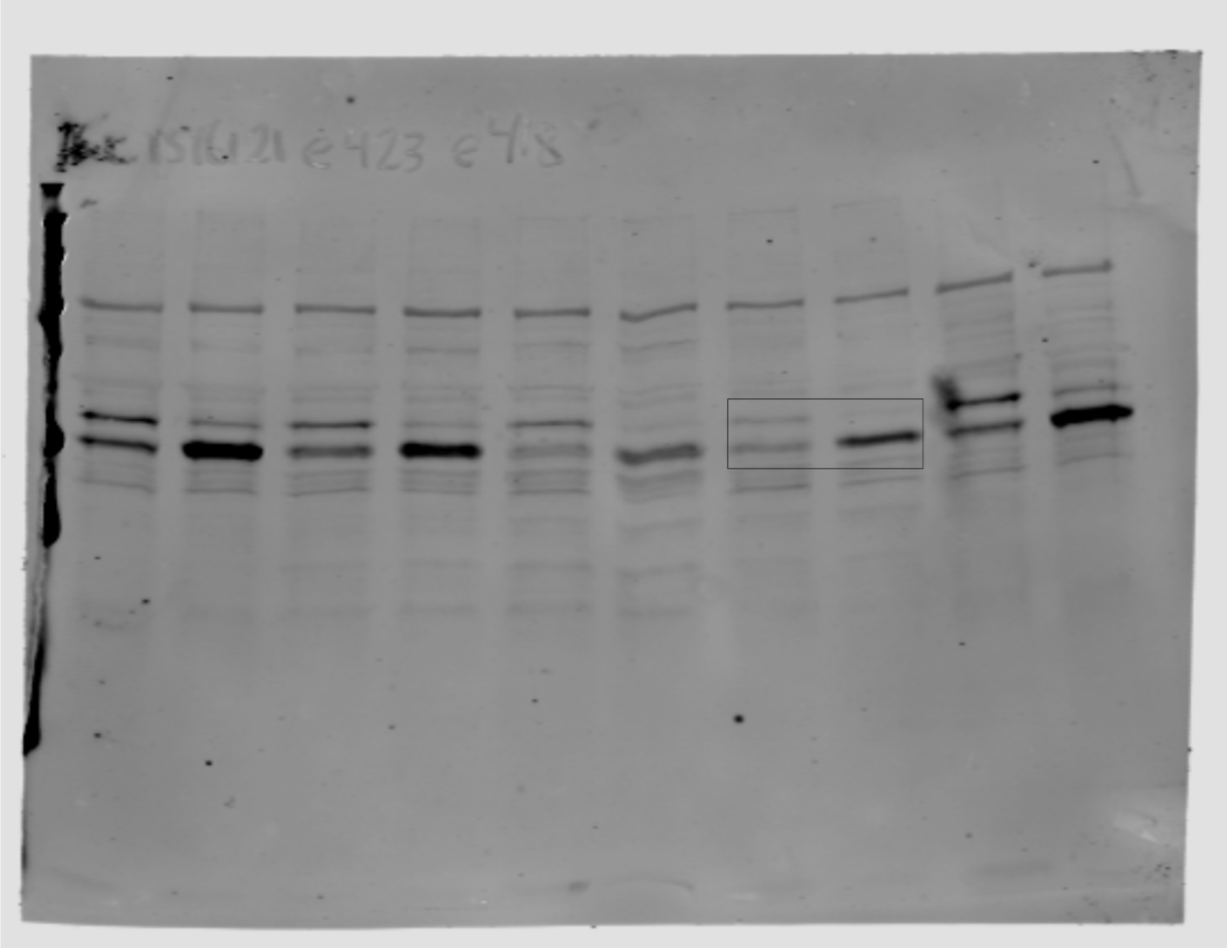

Figure 1—figure supplement 2F – HeLa – HIF1α

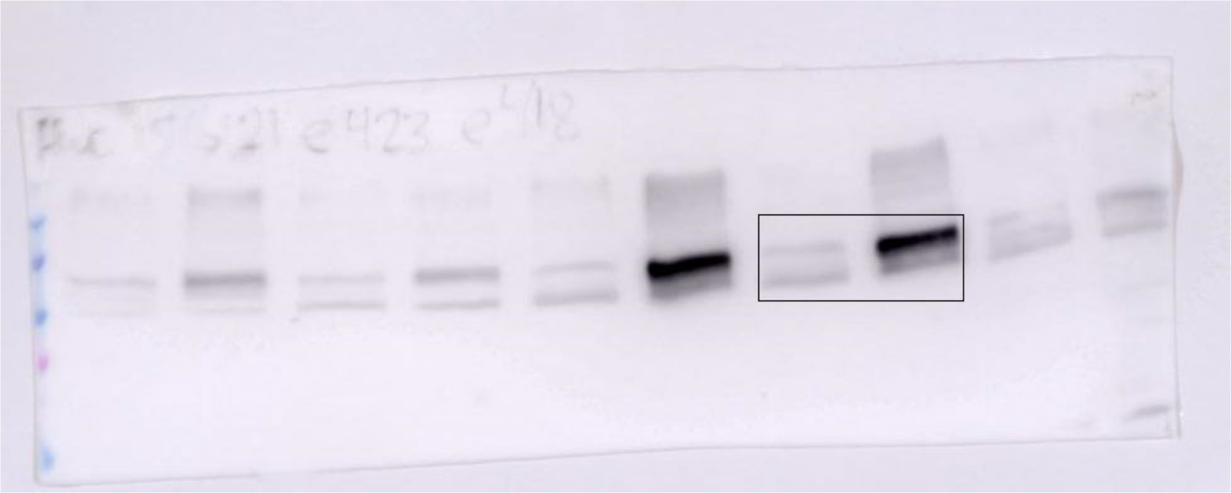

Figure 1—figure supplement 2F – HeLa – GAPDH

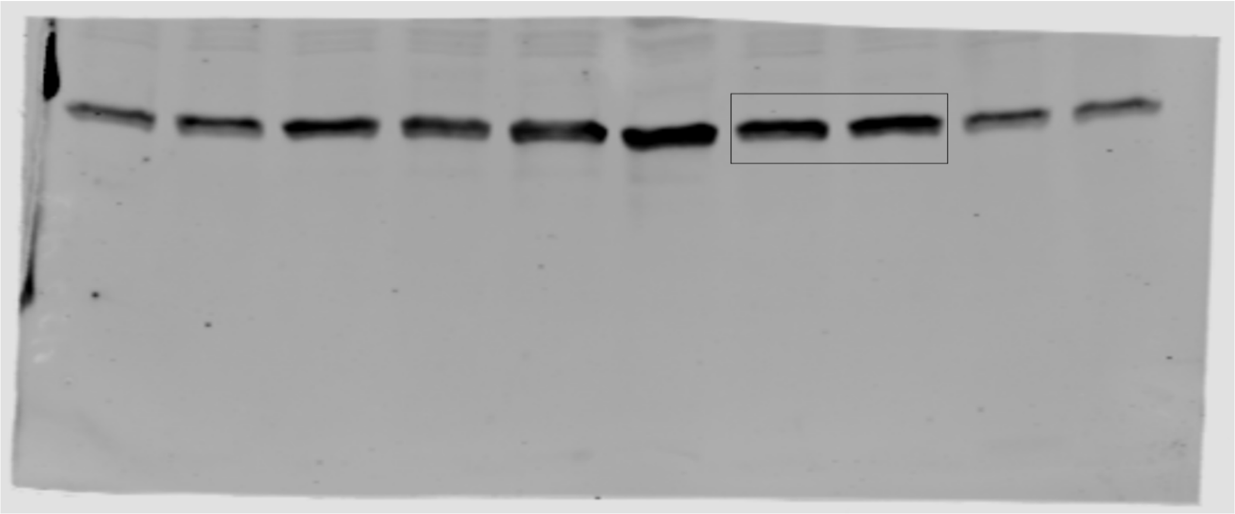

Figure 1—figure supplement 2F – MDA-MB-231 – SM

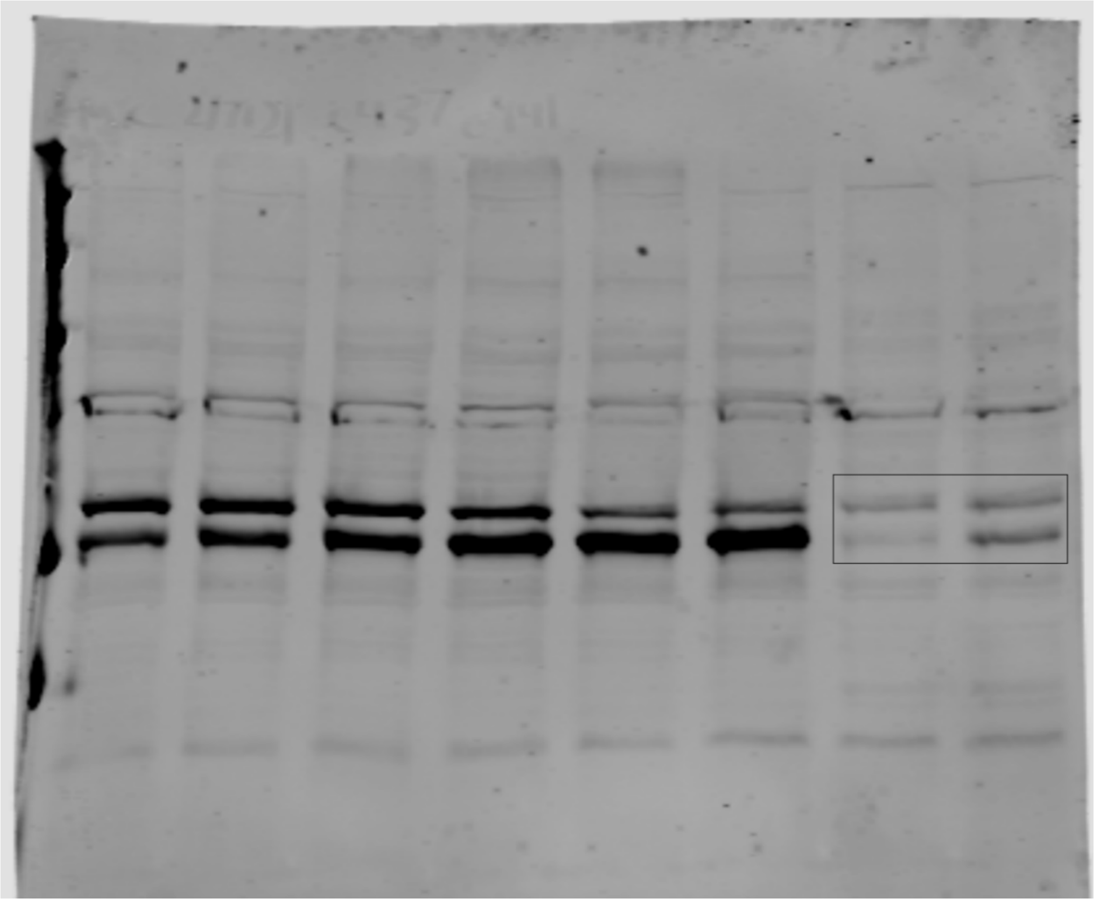

Figure 1—figure supplement 2F – MDA-MB-231 – HIF1α

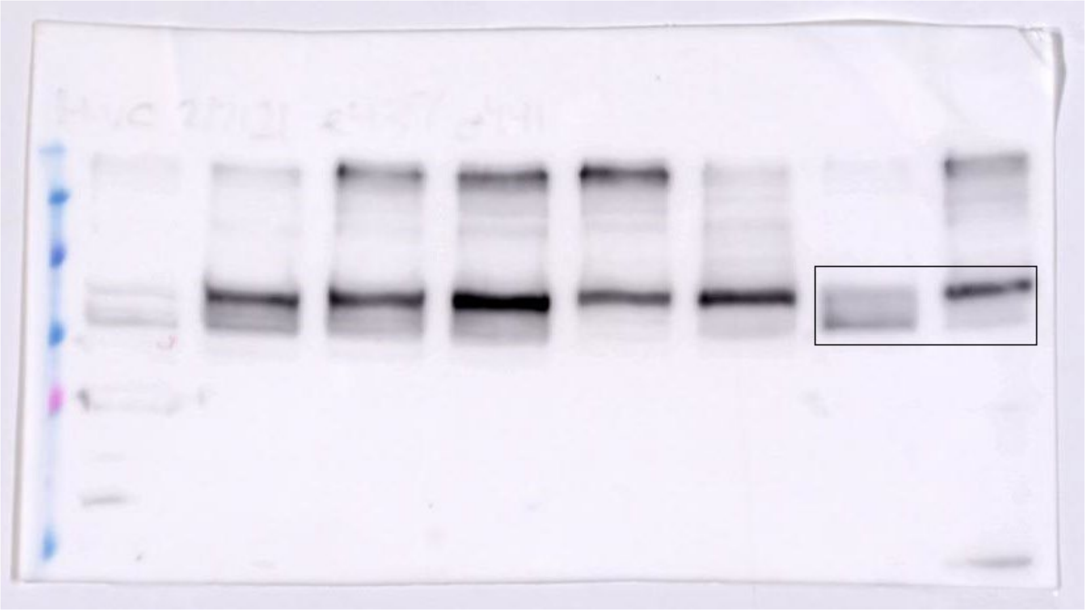

Figure 1—figure supplement 2F – MDA-MB-231 – GAPDH

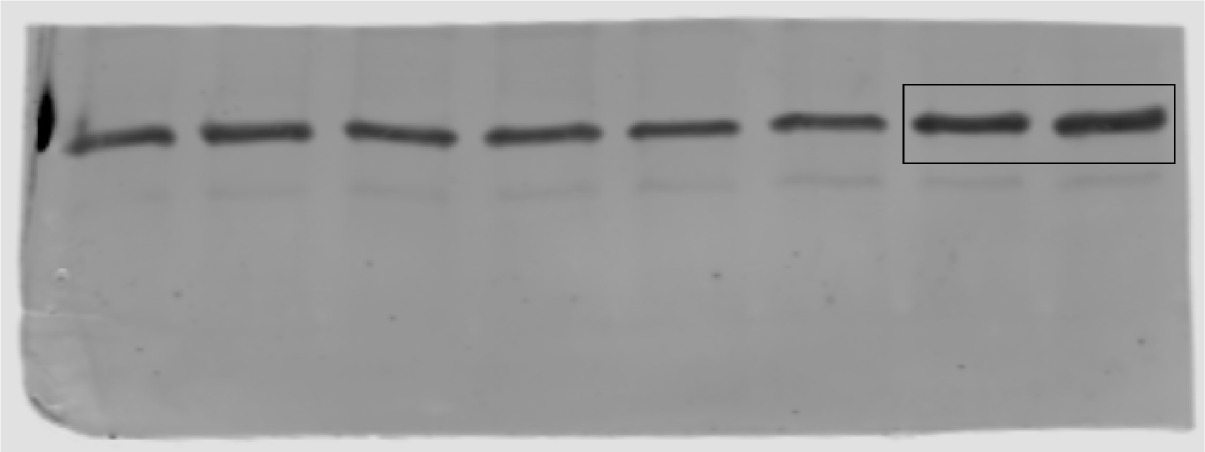

Supplement: Figure 1—figure supplement 2—source data 1. [file elife-82843-fig1-figsupp2-data1.zip › Figure 1-figure supplement 2-annotated source data.pdf]
