# Supplementary material for: Hypoxia truncates and constitutively activates the key cholesterol synthesis enzyme squalene monooxygenase
Source: eLife. 2023 Jan 19;12:e82843. doi: 10.7554/eLife.82843 (PMC9851614; doi:10.7554/eLife.82843)

Figure 1—figure supplement 3B – SM

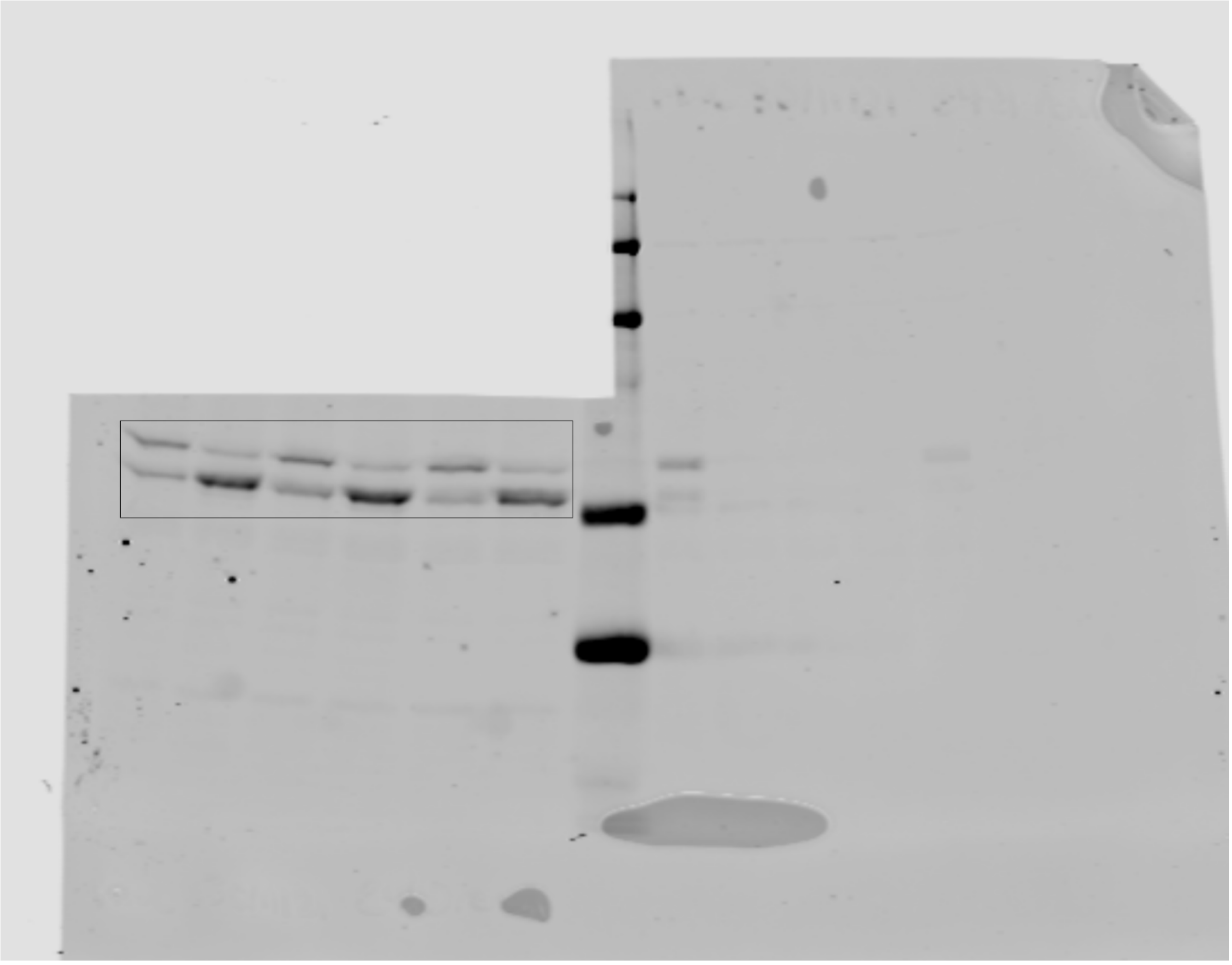

Figure 1—figure supplement 3B – HIF1α

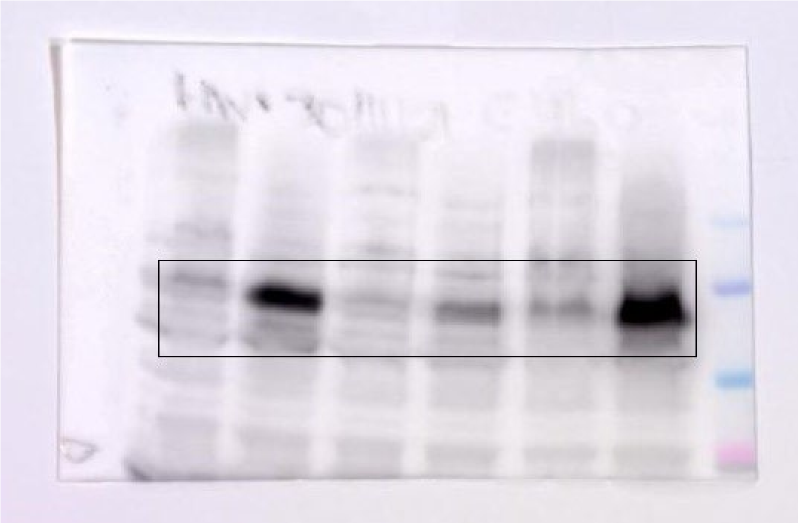

Figure 1—figure supplement 3B – GAPDH

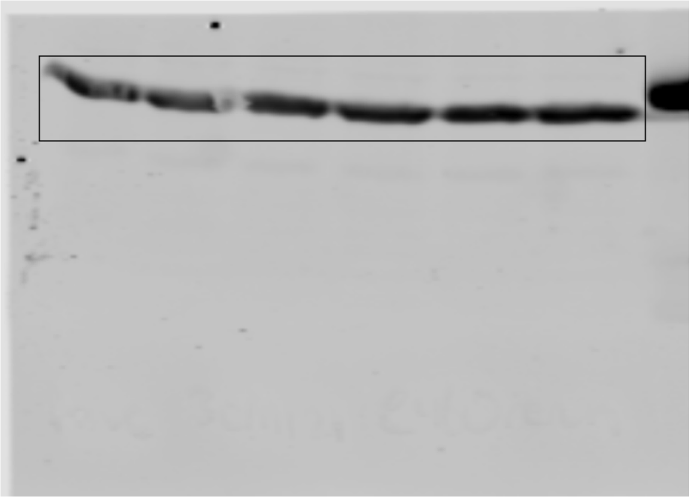

Supplement: Figure 1—figure supplement 3—source data 1. [file elife-82843-fig1-figsupp3-data1.zip › Figure 1-figure supplement 3-annotated source data.pdf]
