# Supplementary figures and images for: Hypoxia truncates and constitutively activates the key cholesterol synthesis enzyme squalene monooxygenase (part 2 of 2)
Source: eLife. 2023 Jan 19;12:e82843. doi: 10.7554/eLife.82843 (PMC9851614; doi:10.7554/eLife.82843)

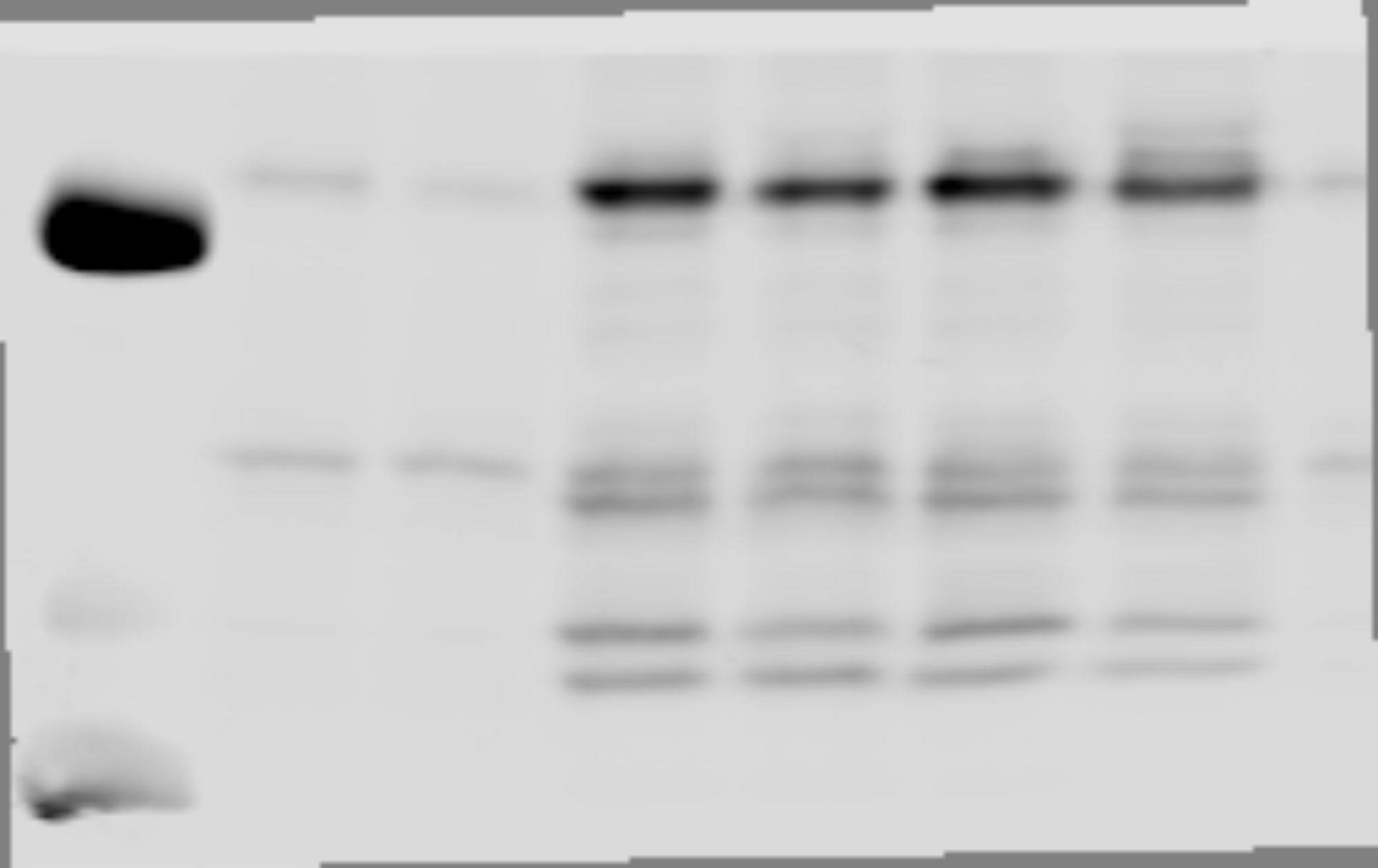

Supplement: Figure 2—source data 1. [file elife-82843-fig2-data1.zip › Fig. 2C V5.tif]

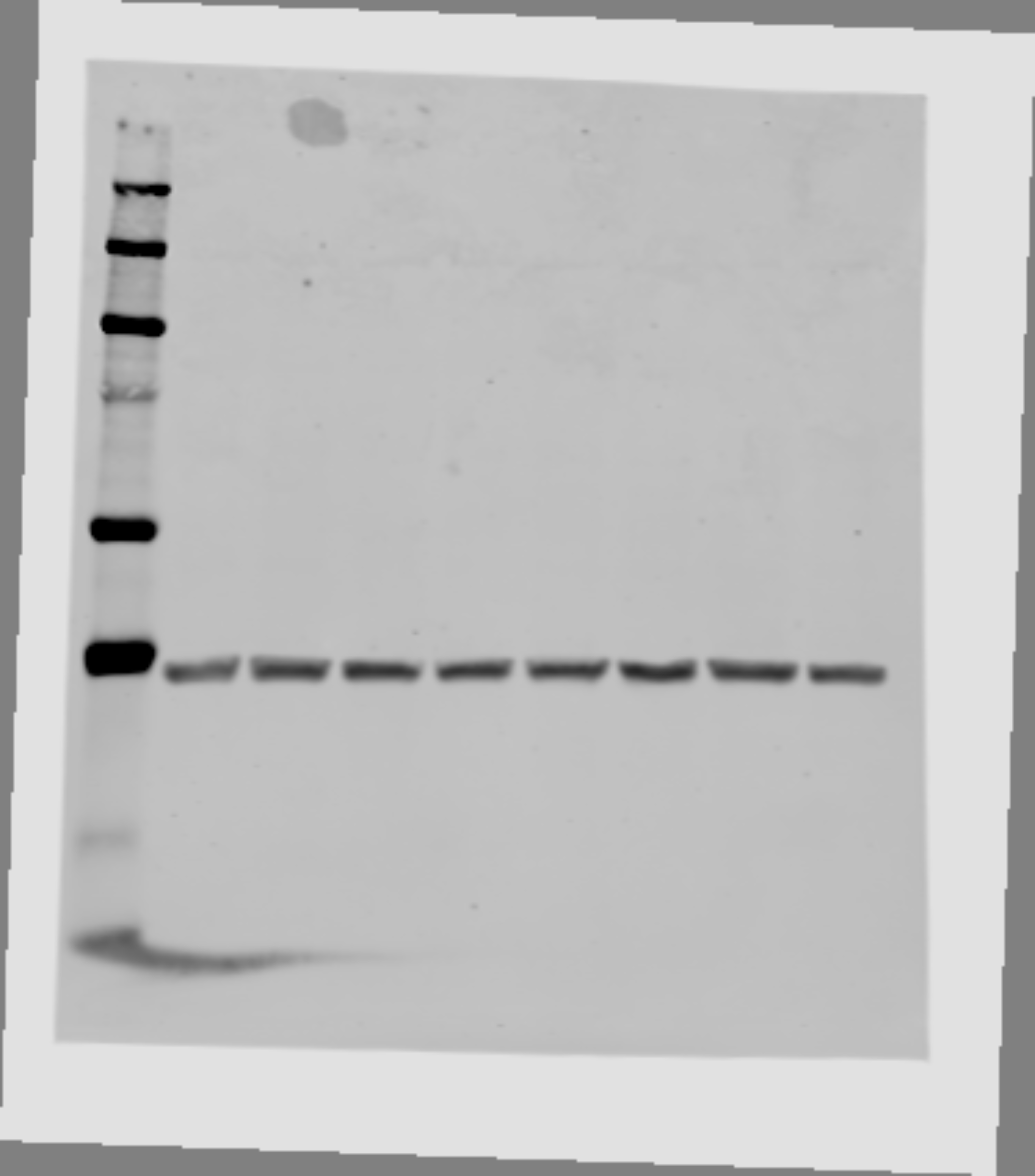

Supplement: Figure 2—source data 1. [file elife-82843-fig2-data1.zip › Fig. 2D GAPDH.tif]

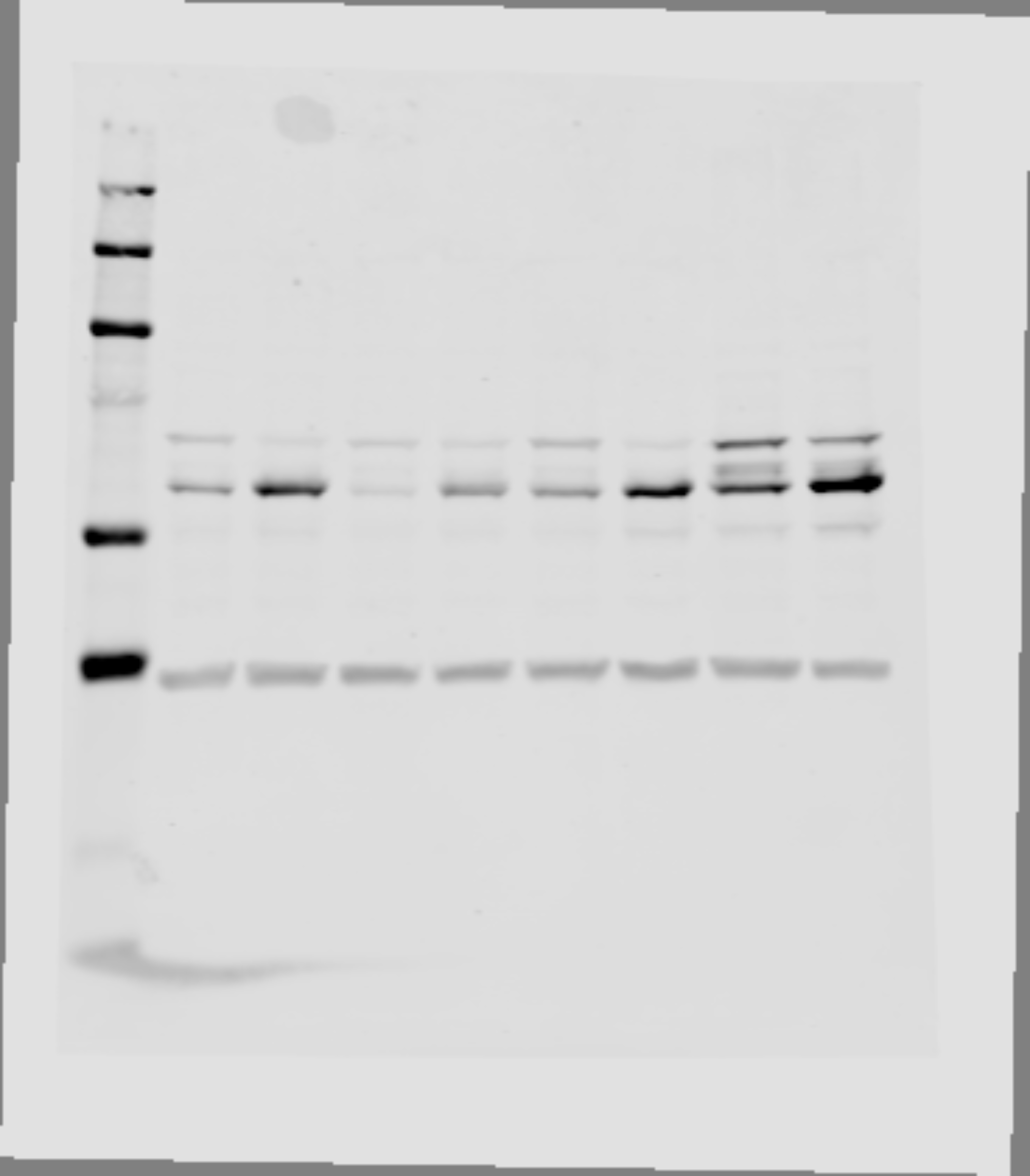

Supplement: Figure 2—source data 1. [file elife-82843-fig2-data1.zip › Fig. 2D V5.tif]

**Figure 2B – V5**

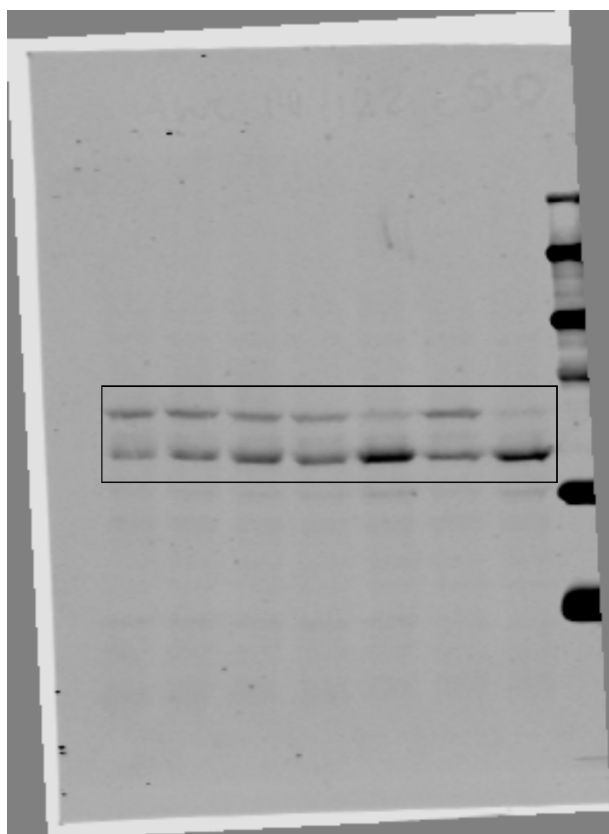

**Figure 2B – GAPDH**

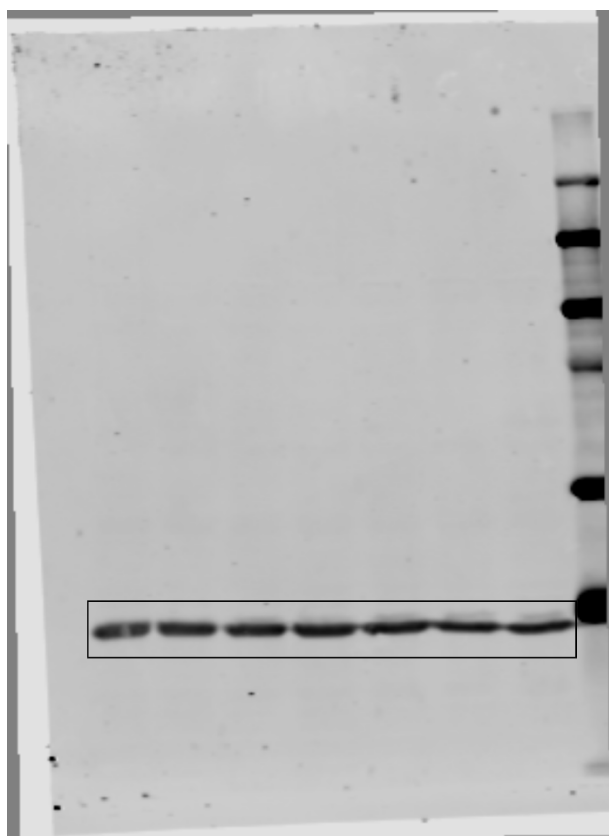

**Figure 2C – SM**

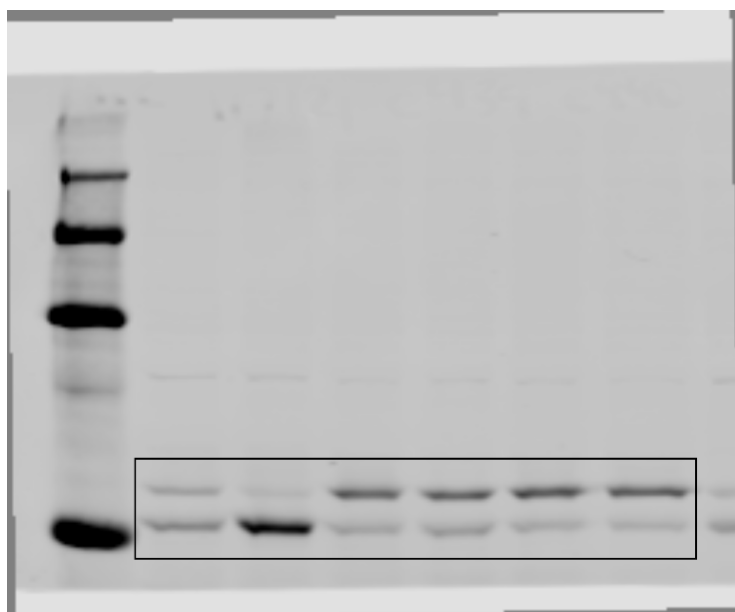

**Figure 2C – V5**

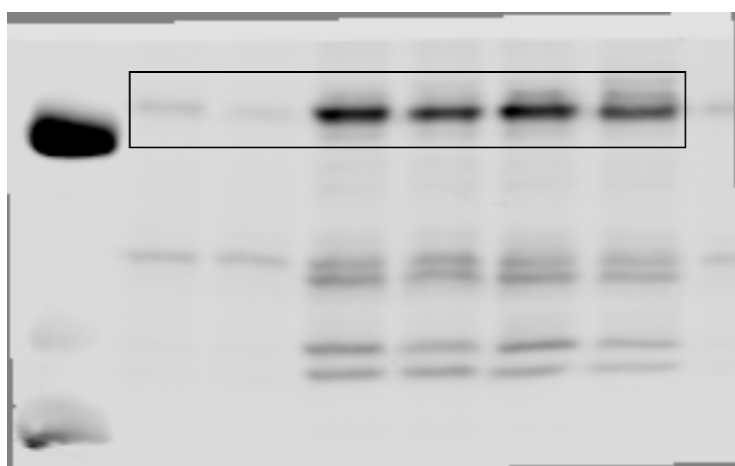

**Figure 2C – GAPDH**

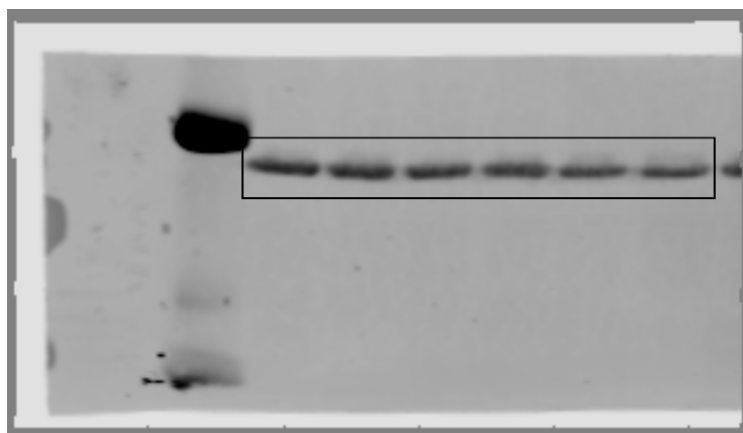

**Figure 2D – V5**

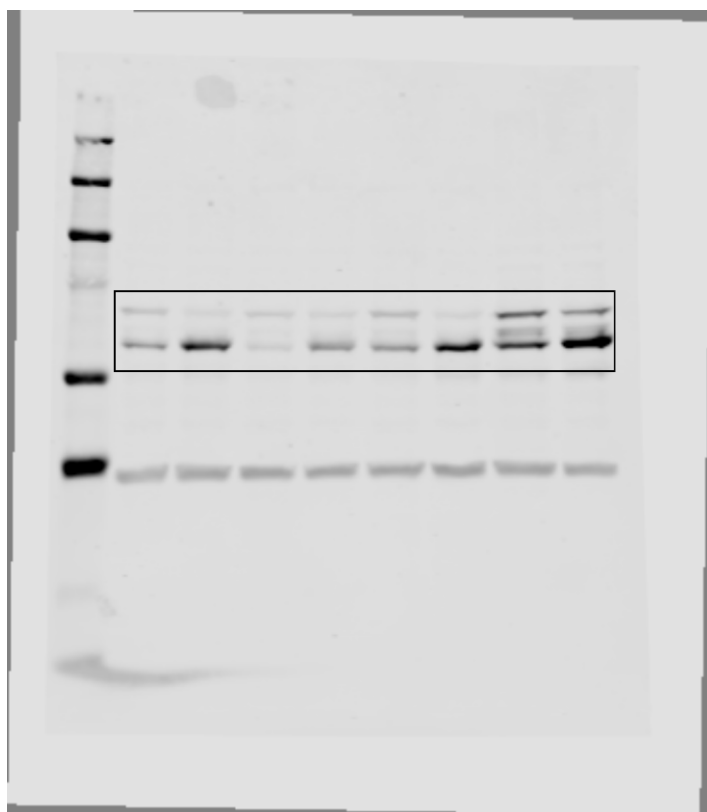

**Figure 2D – GAPDH**

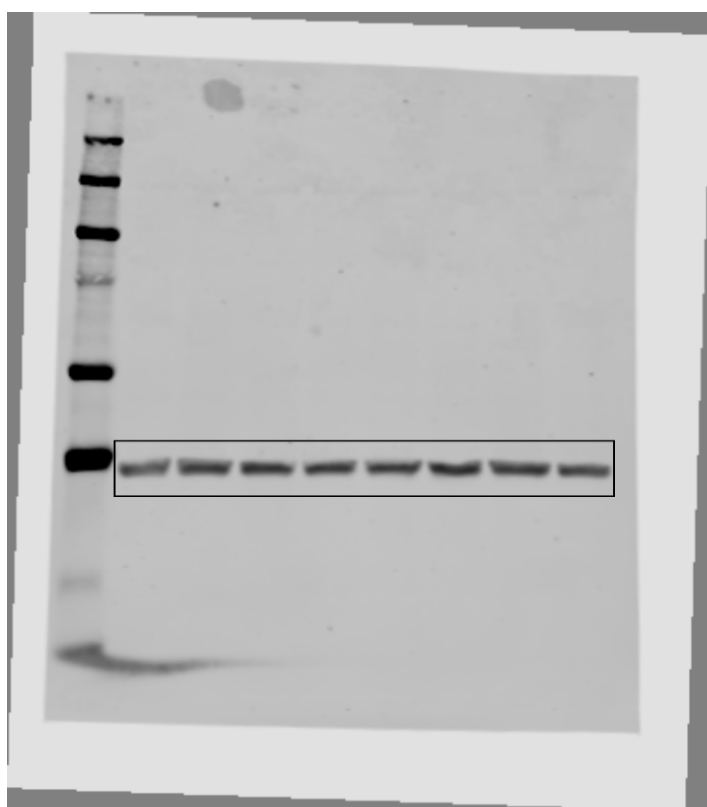

Supplement: Figure 2—source data 1. [file elife-82843-fig2-data1.zip › Figure 2-annotated source data.pdf]

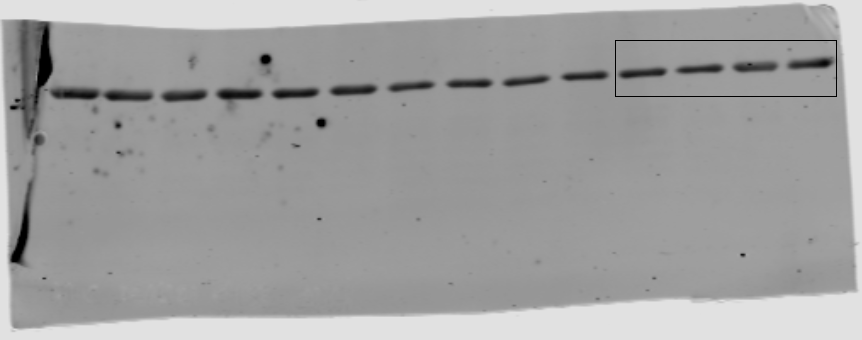

Supplement: Figure 3—source data 1. [file elife-82843-fig3-data1.zip › Annotated/Fig. 3A GAPDH.tif]

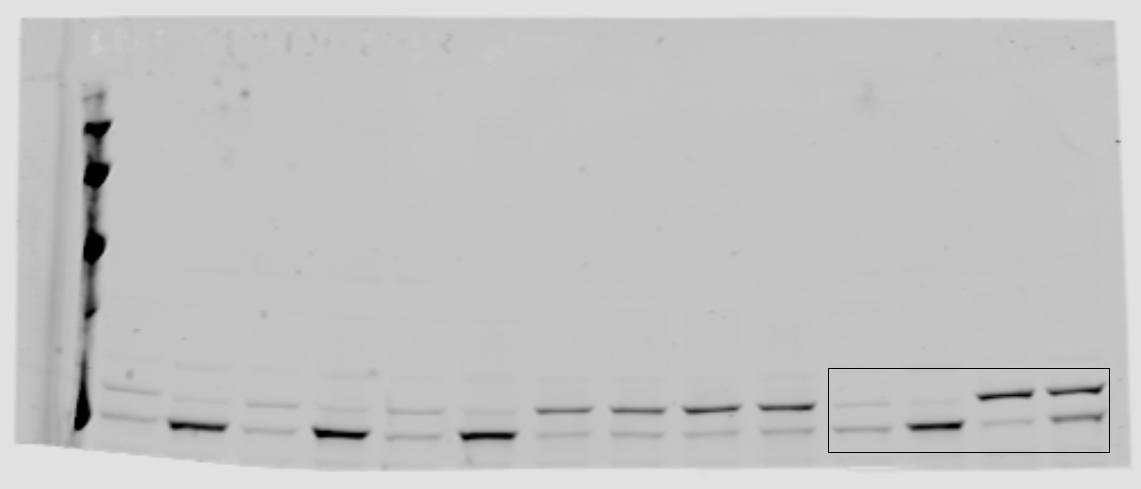

Supplement: Figure 3—source data 1. [file elife-82843-fig3-data1.zip › Annotated/Fig. 3A SM.tif]

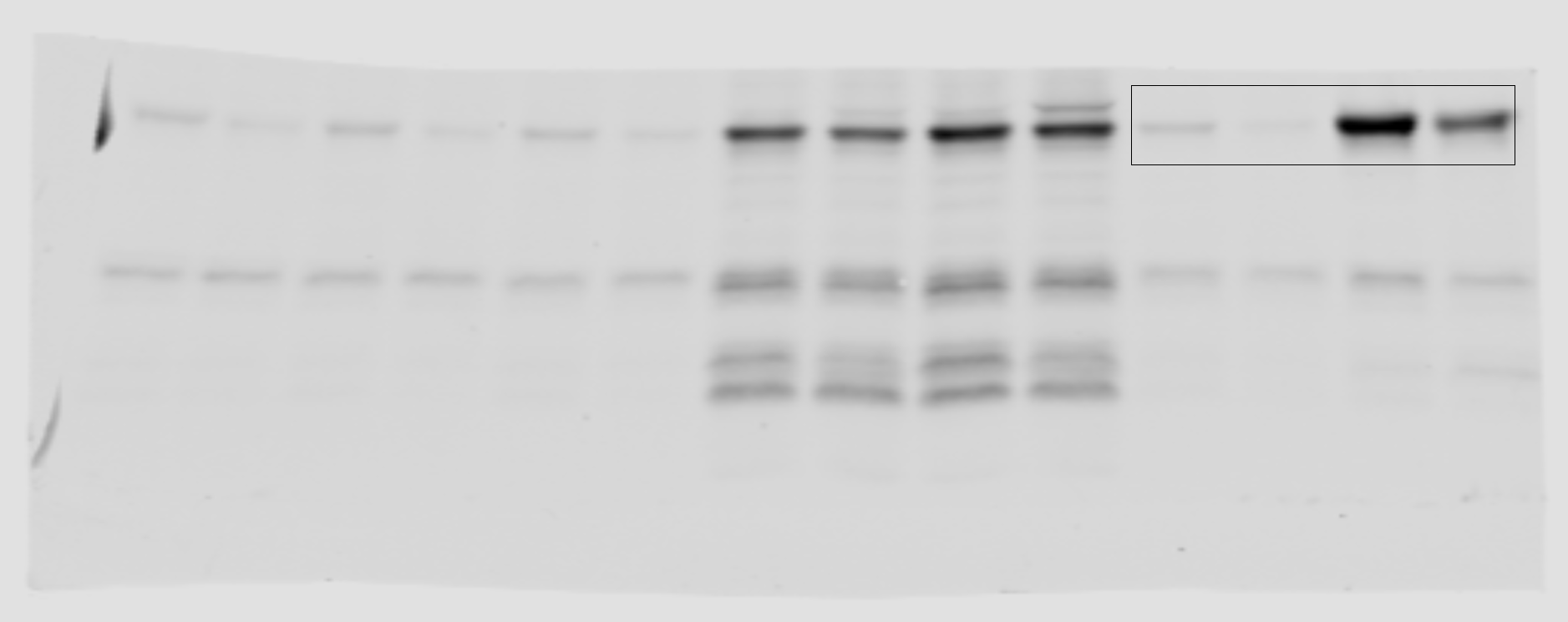

Supplement: Figure 3—source data 1. [file elife-82843-fig3-data1.zip › Annotated/Fig. 3A V5.tif]

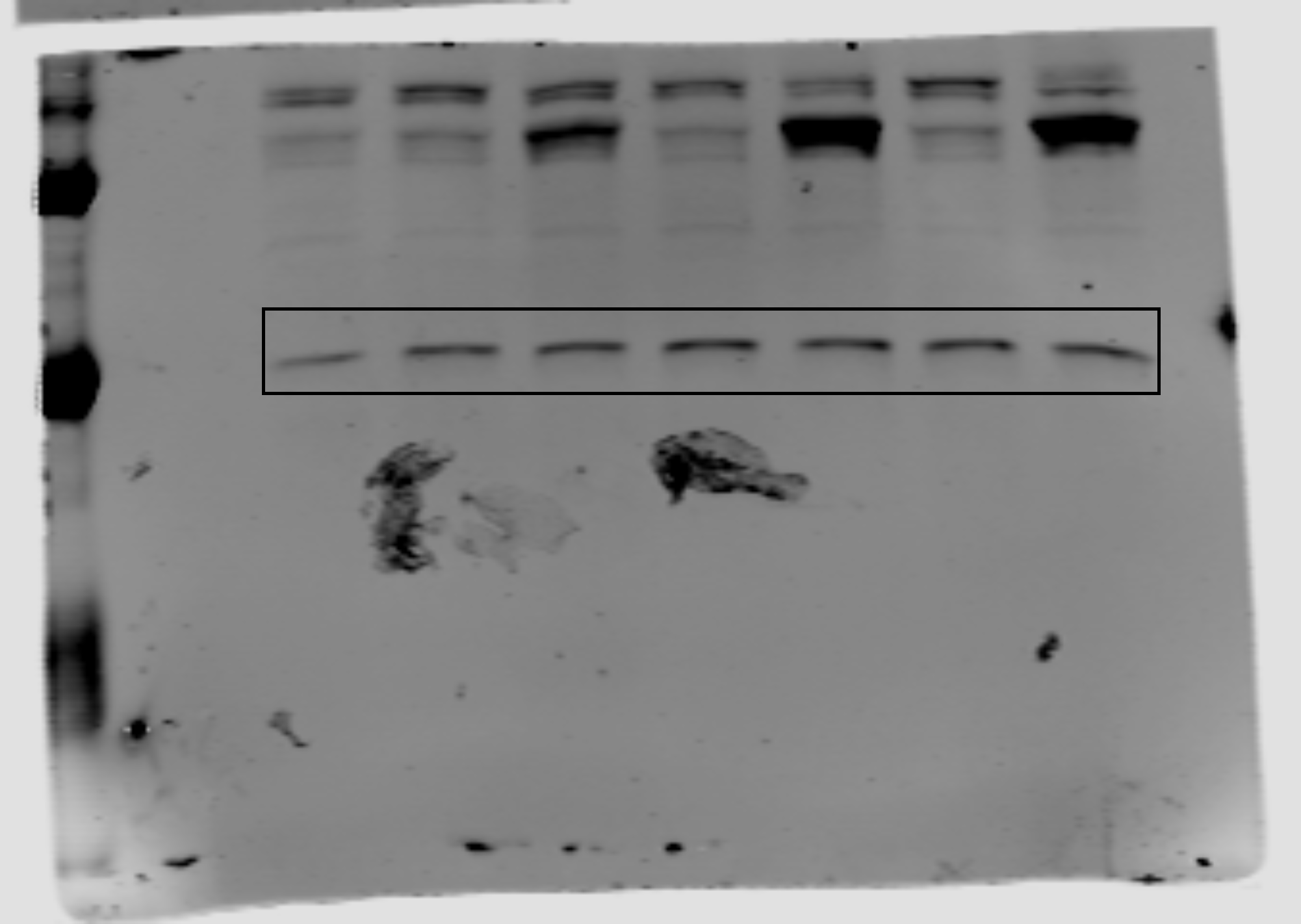

Supplement: Figure 3—source data 1. [file elife-82843-fig3-data1.zip › Annotated/Fig. 3B GAPDH.tif]

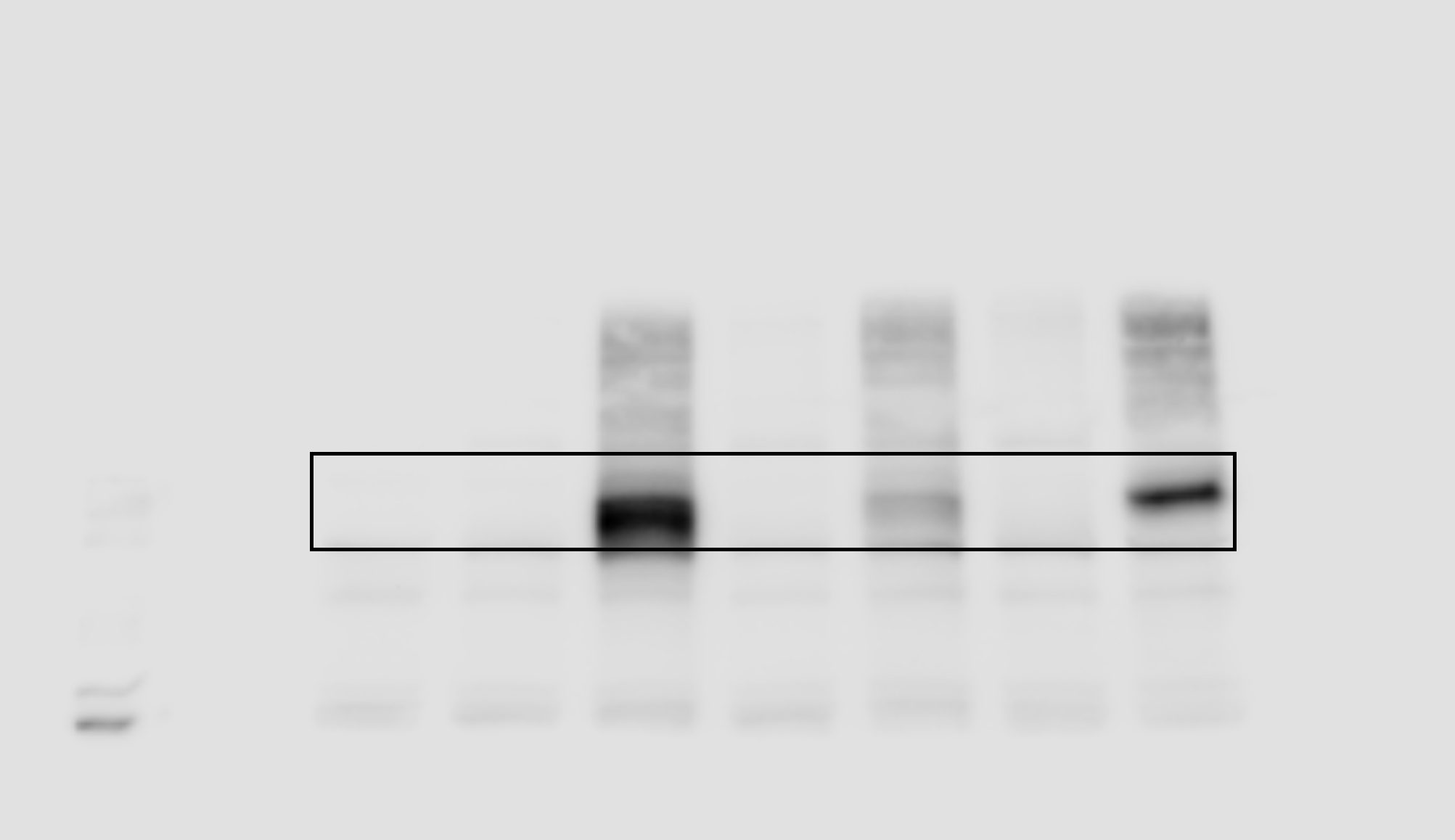

Supplement: Figure 3—source data 1. [file elife-82843-fig3-data1.zip › Annotated/Fig. 3B HIF1a.tif]

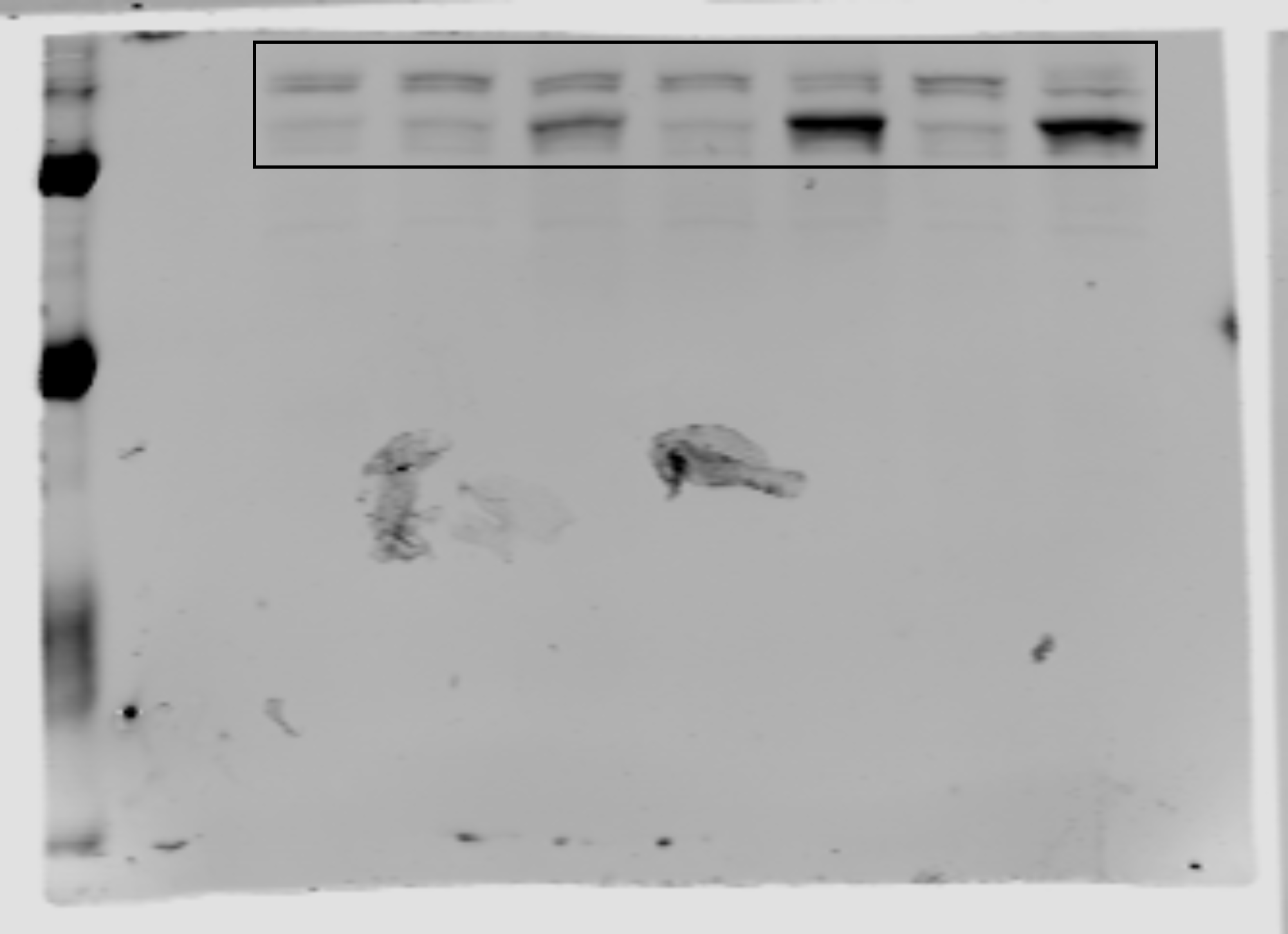

Supplement: Figure 3—source data 1. [file elife-82843-fig3-data1.zip › Annotated/Fig. 3B SM.tif]

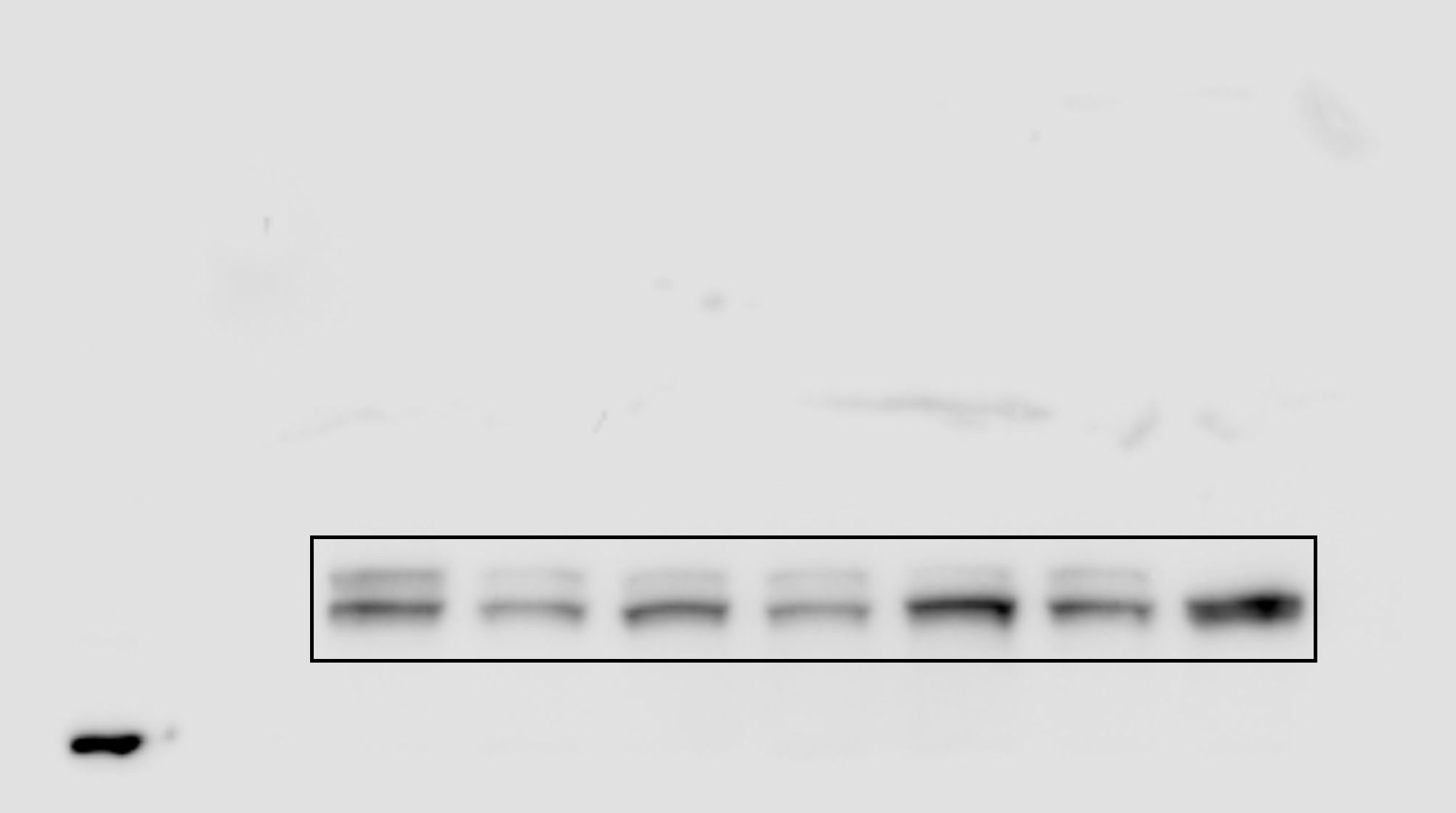

Supplement: Figure 3—source data 1. [file elife-82843-fig3-data1.zip › Annotated/Fig. 3B V5.tif]

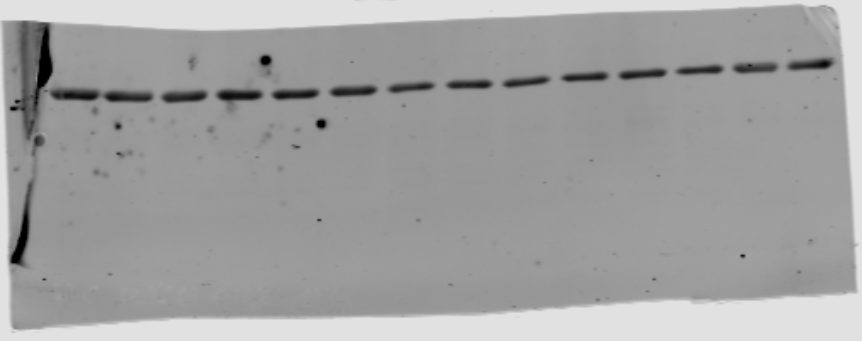

Supplement: Figure 3—source data 1. [file elife-82843-fig3-data1.zip › Fig. 3A GAPDH.tif]

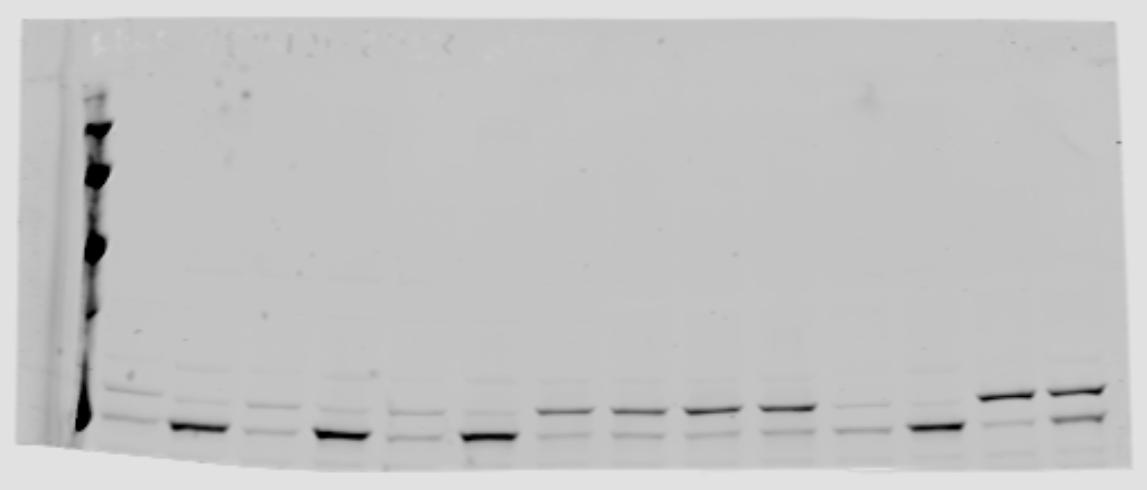

Supplement: Figure 3—source data 1. [file elife-82843-fig3-data1.zip › Fig. 3A SM.tif]

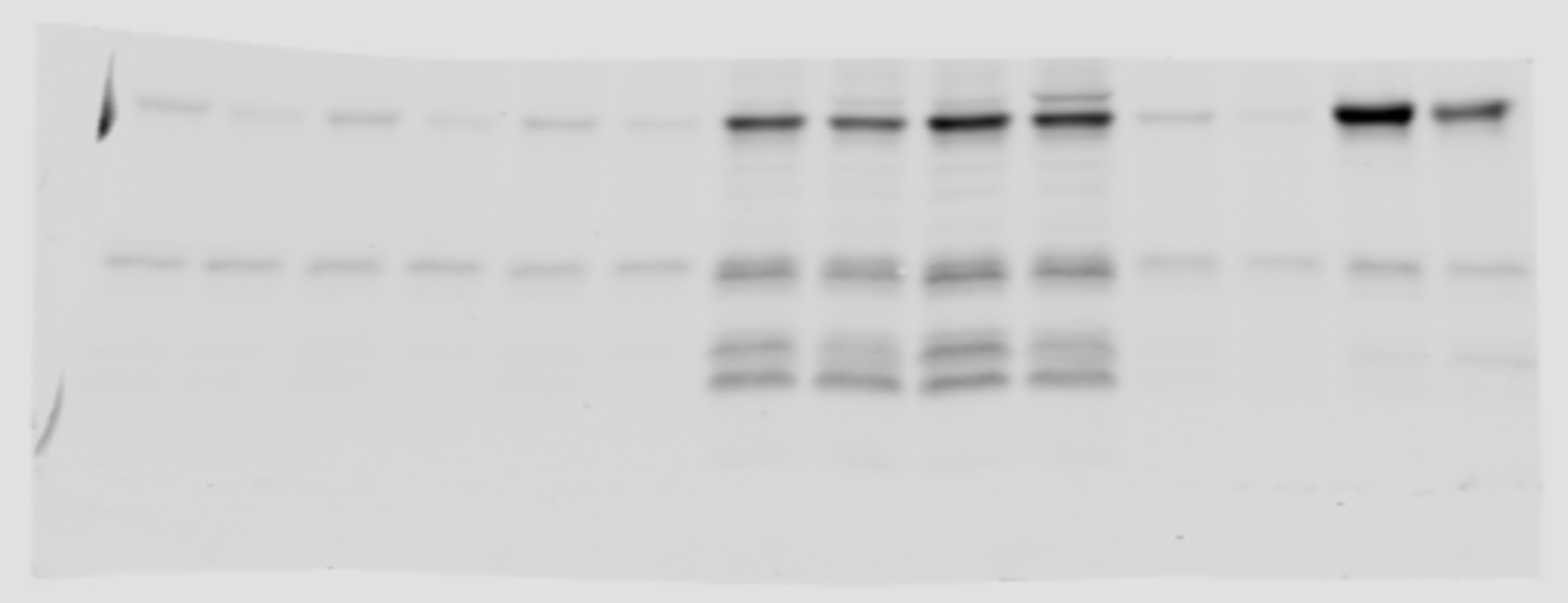

Supplement: Figure 3—source data 1. [file elife-82843-fig3-data1.zip › Fig. 3A V5.tif]

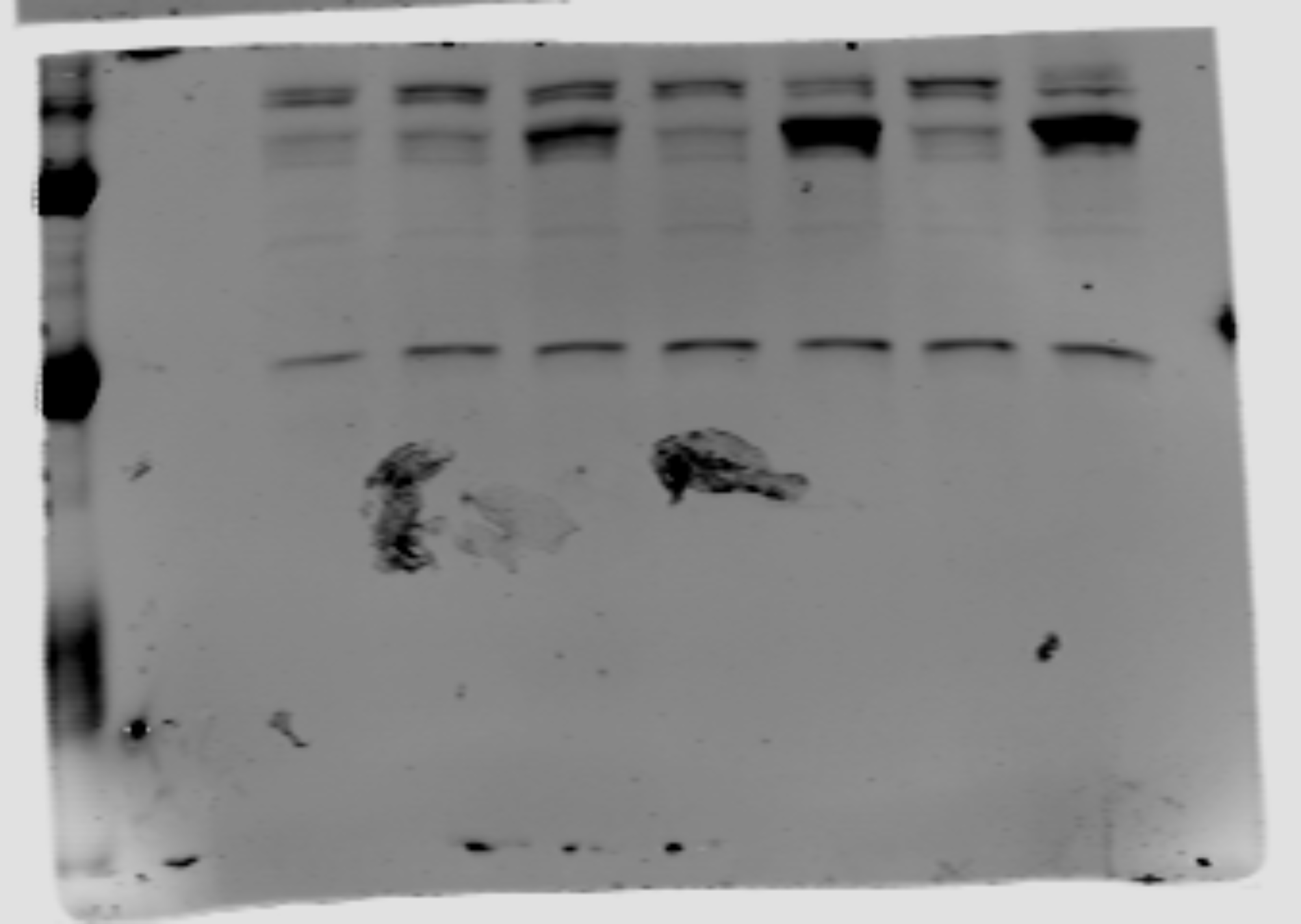

Supplement: Figure 3—source data 1. [file elife-82843-fig3-data1.zip › Fig. 3B GAPDH.tif]

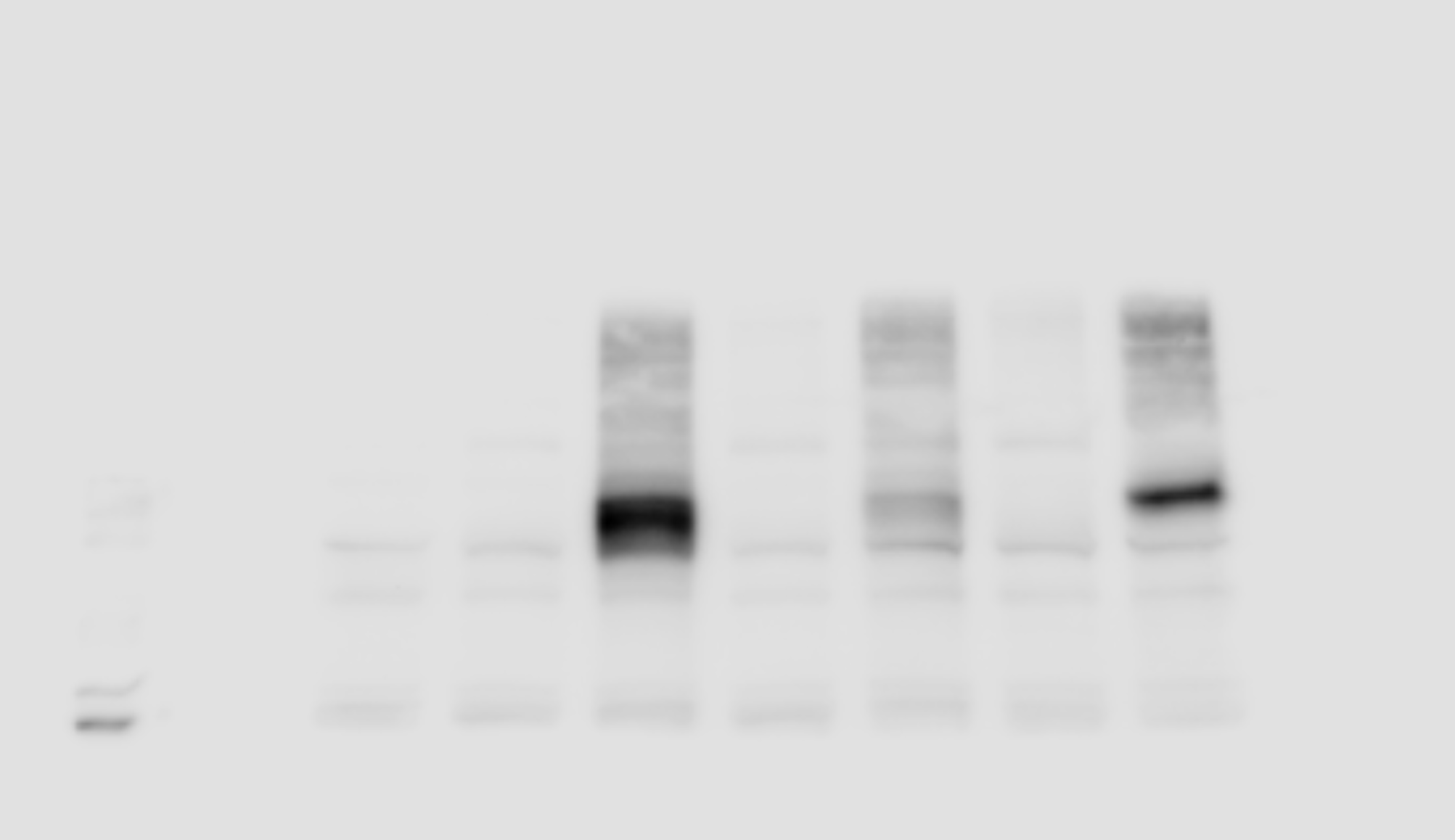

Supplement: Figure 3—source data 1. [file elife-82843-fig3-data1.zip › Fig. 3B HIF1a.tif]

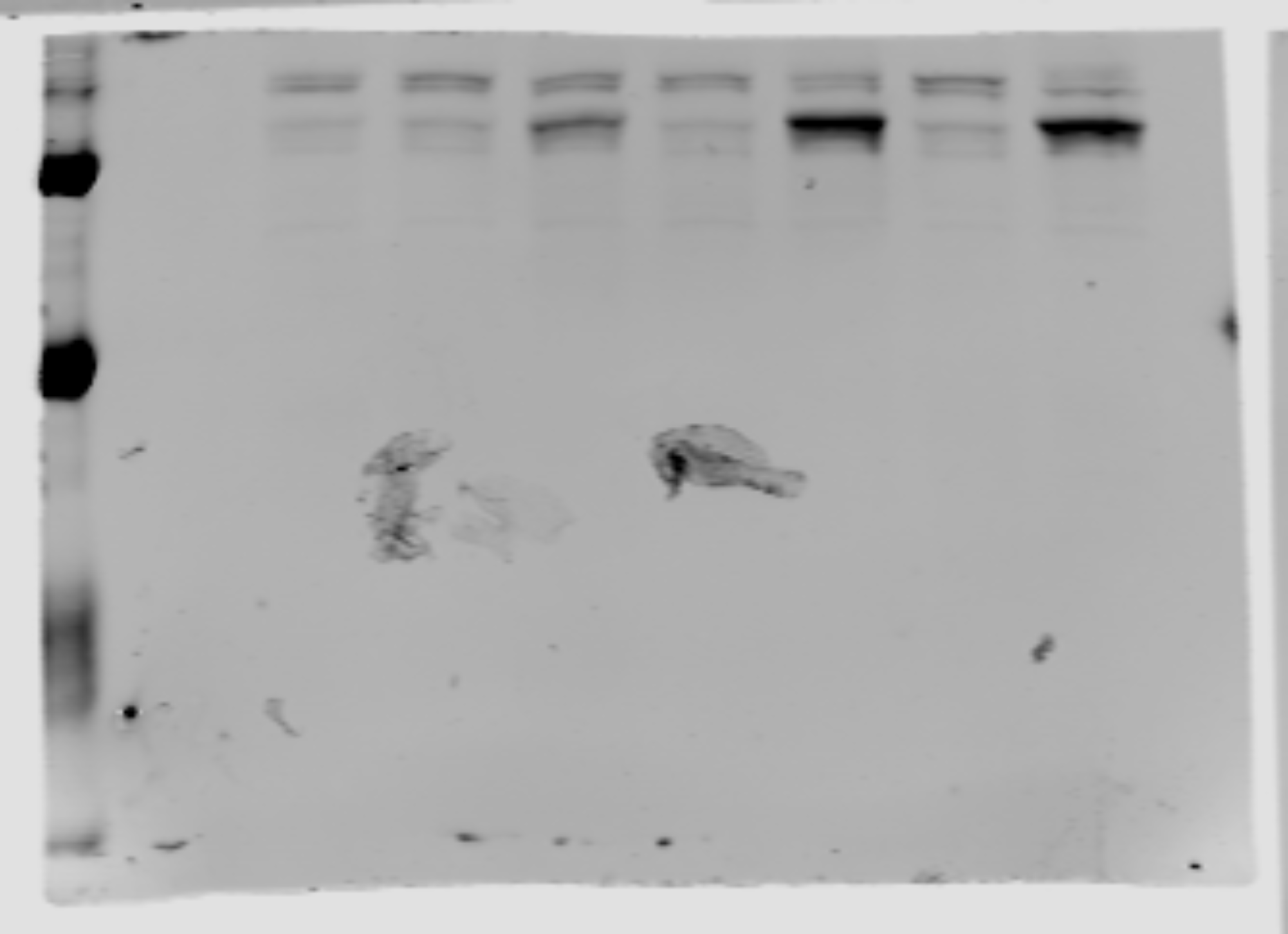

Supplement: Figure 3—source data 1. [file elife-82843-fig3-data1.zip › Fig. 3B SM.tif]

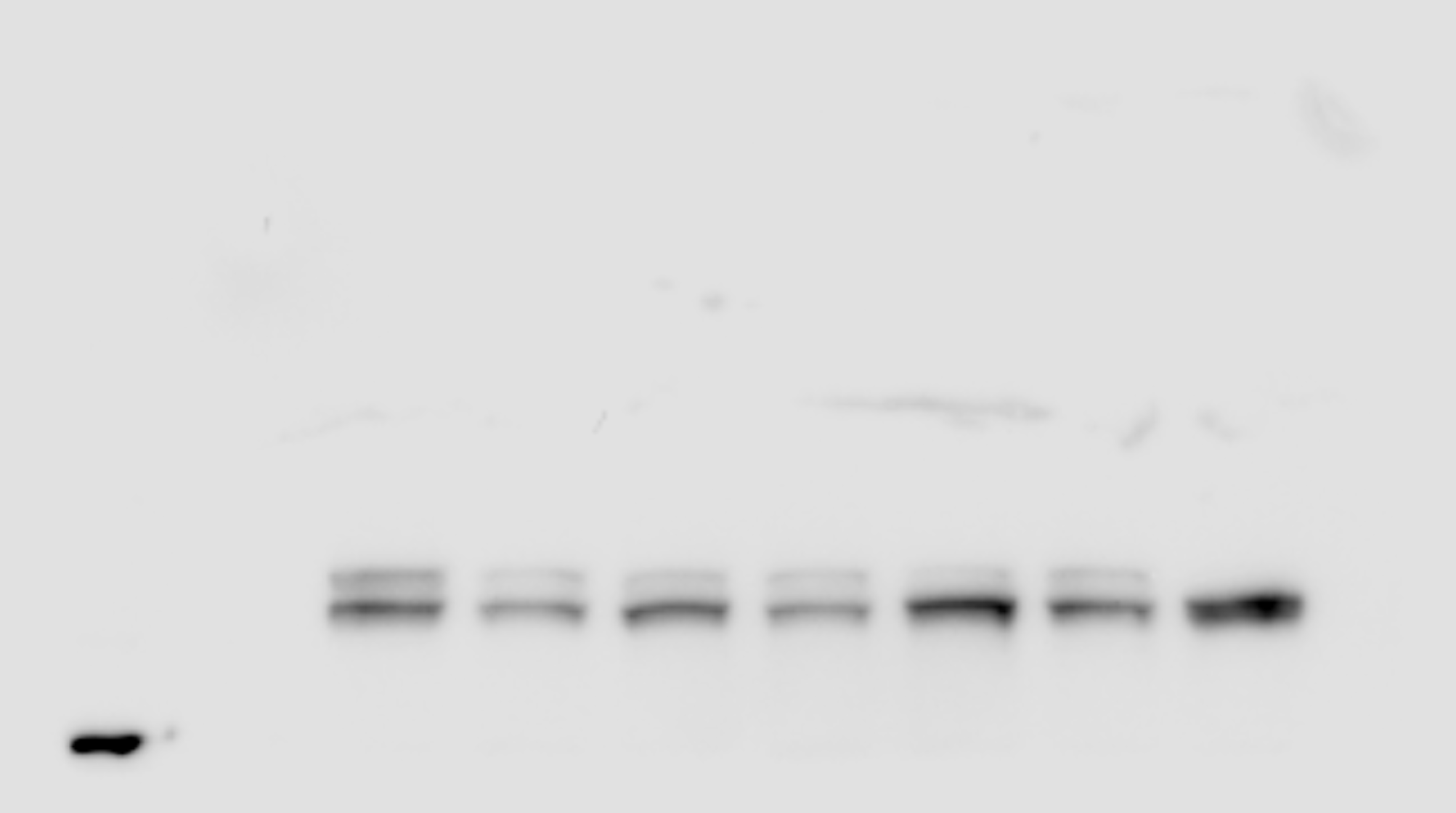

Supplement: Figure 3—source data 1. [file elife-82843-fig3-data1.zip › Fig. 3B V5.tif]

**Figure 3A – SM**

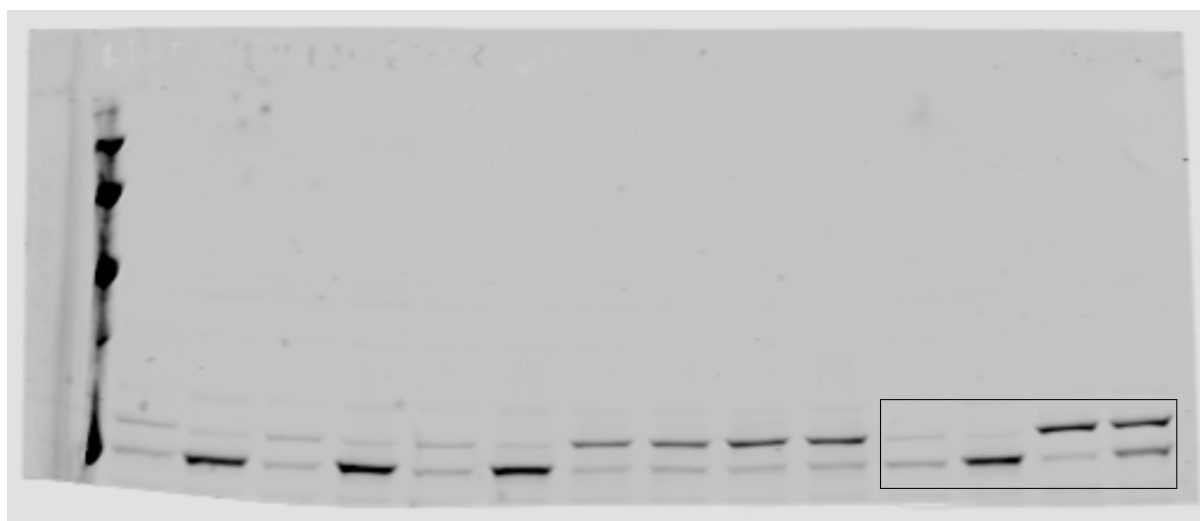

**Figure 3A – V5**

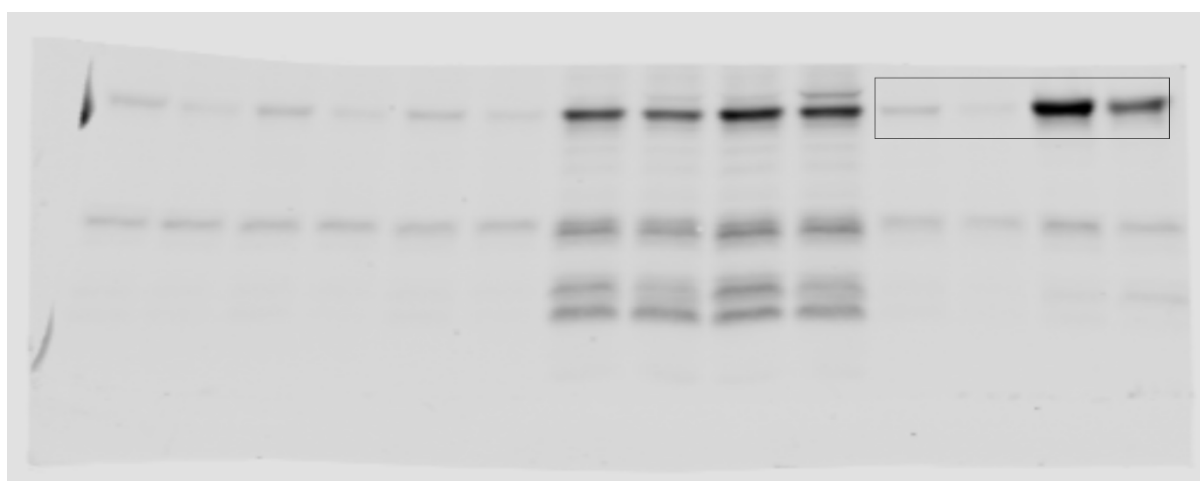

**Figure 3A – GAPDH**

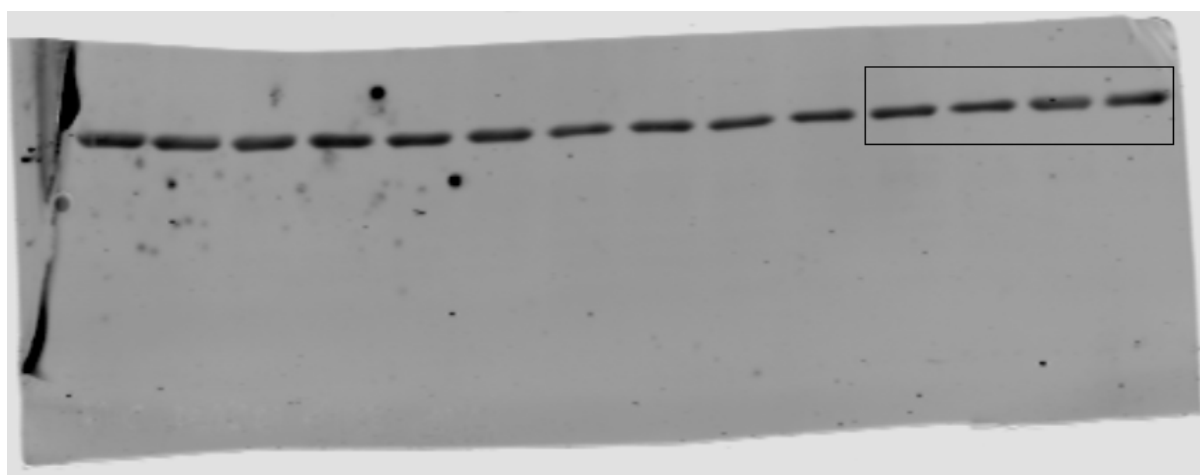

**Figure 3B – V5**

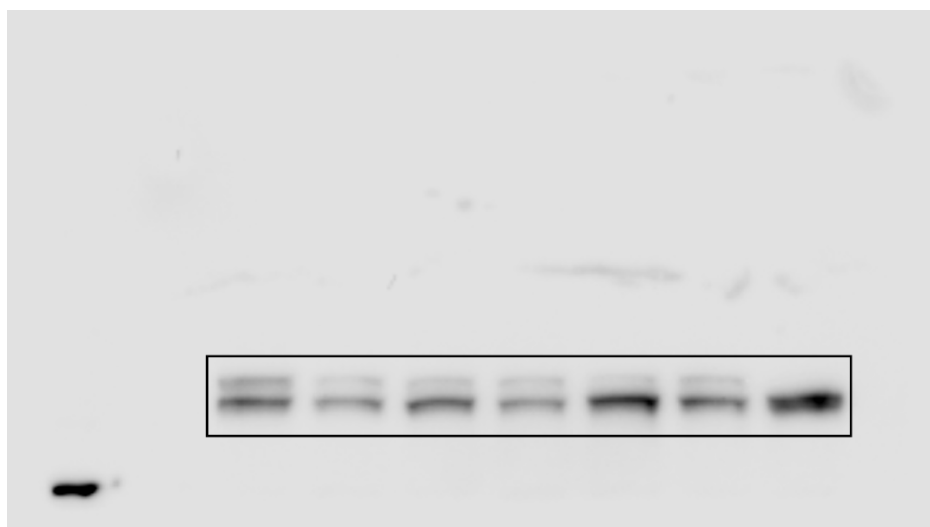

**Figure 3B – SM**

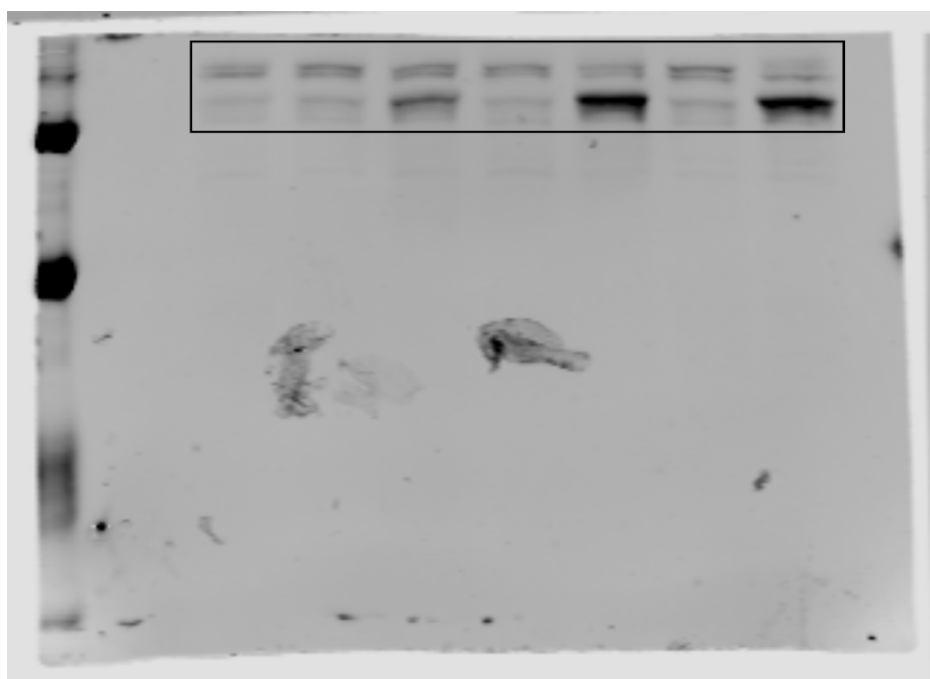

**Figure 3B – HIF1 $\alpha$**

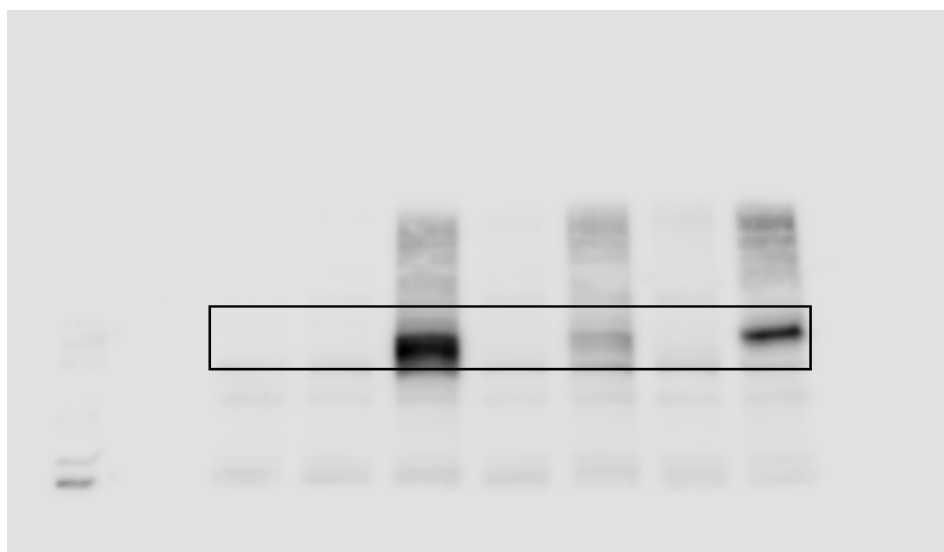

**Figure 3B – GAPDH**

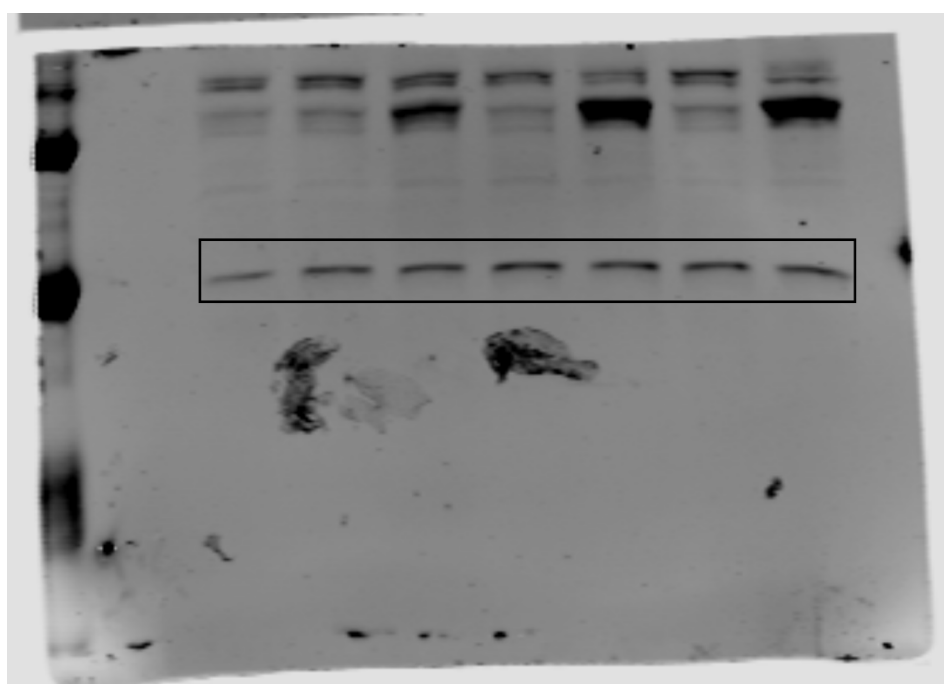

Supplement: Figure 3—source data 1. [file elife-82843-fig3-data1.zip › Figure 3-annotated source data.pdf]

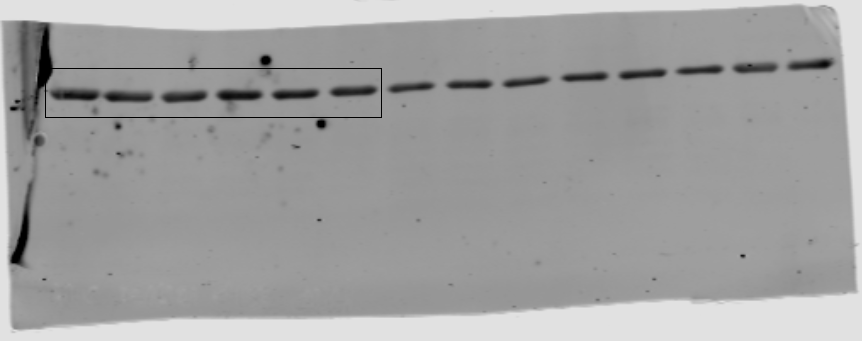

Supplement: Figure 3—figure supplement 1—source data 1. [file elife-82843-fig3-figsupp1-data1.zip › Annotated/Fig. 3-1A GAPDH.tif]

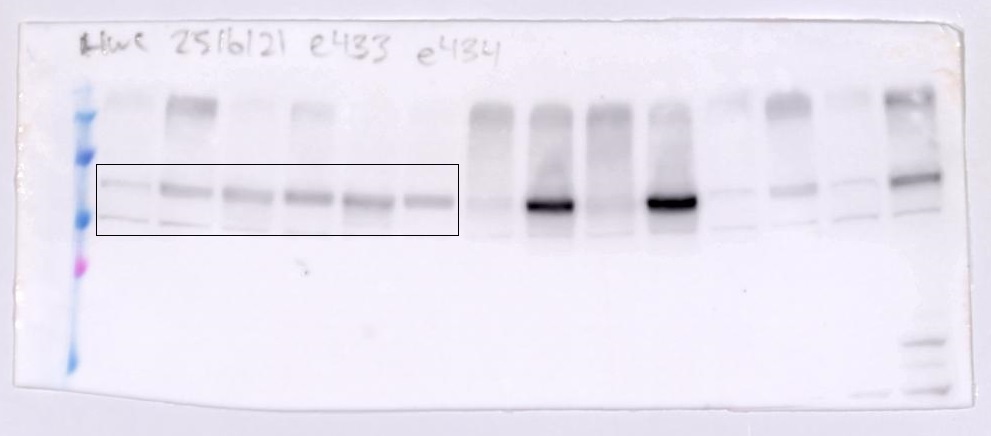

Supplement: Figure 3—figure supplement 1—source data 1. [file elife-82843-fig3-figsupp1-data1.zip › Annotated/Fig. 3-1A HIF1a.jpg]

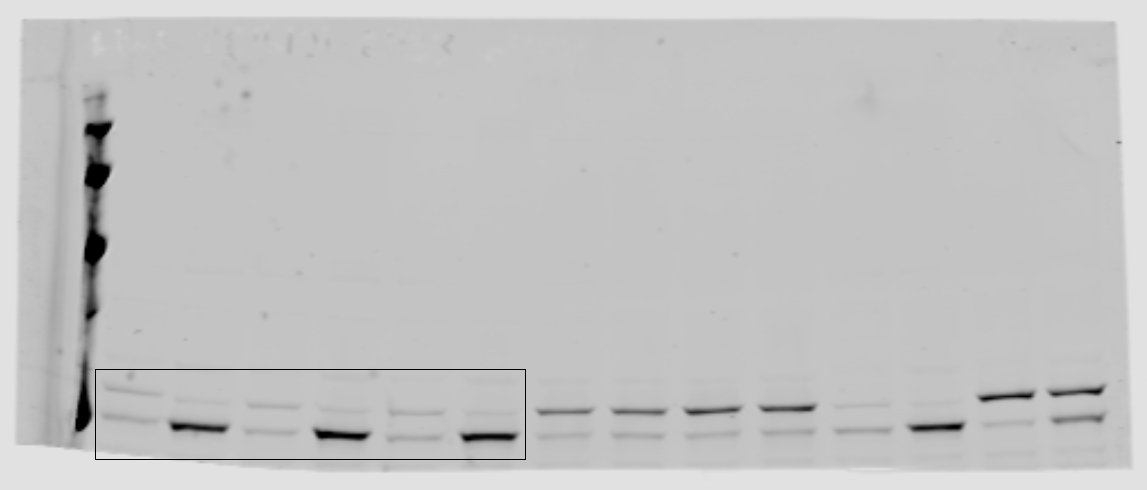

Supplement: Figure 3—figure supplement 1—source data 1. [file elife-82843-fig3-figsupp1-data1.zip › Annotated/Fig. 3-1A SM.tif]

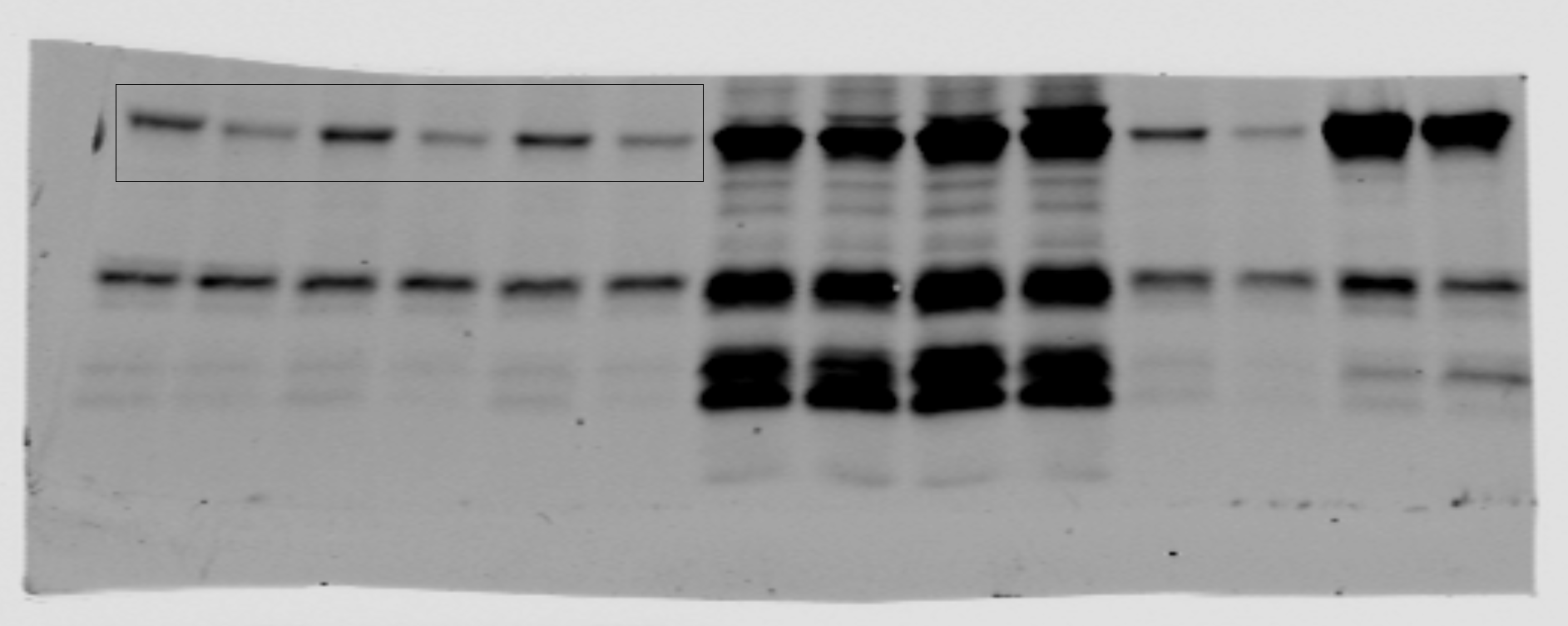

Supplement: Figure 3—figure supplement 1—source data 1. [file elife-82843-fig3-figsupp1-data1.zip › Annotated/Fig. 3-1A V5.tif]

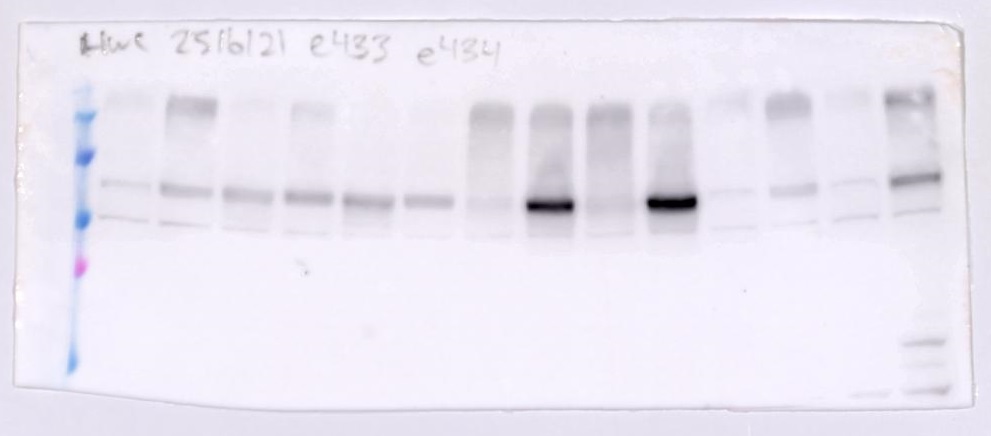

Supplement: Figure 3—figure supplement 1—source data 1. [file elife-82843-fig3-figsupp1-data1.zip › Fig. 3-1A HIF1a.jpg]

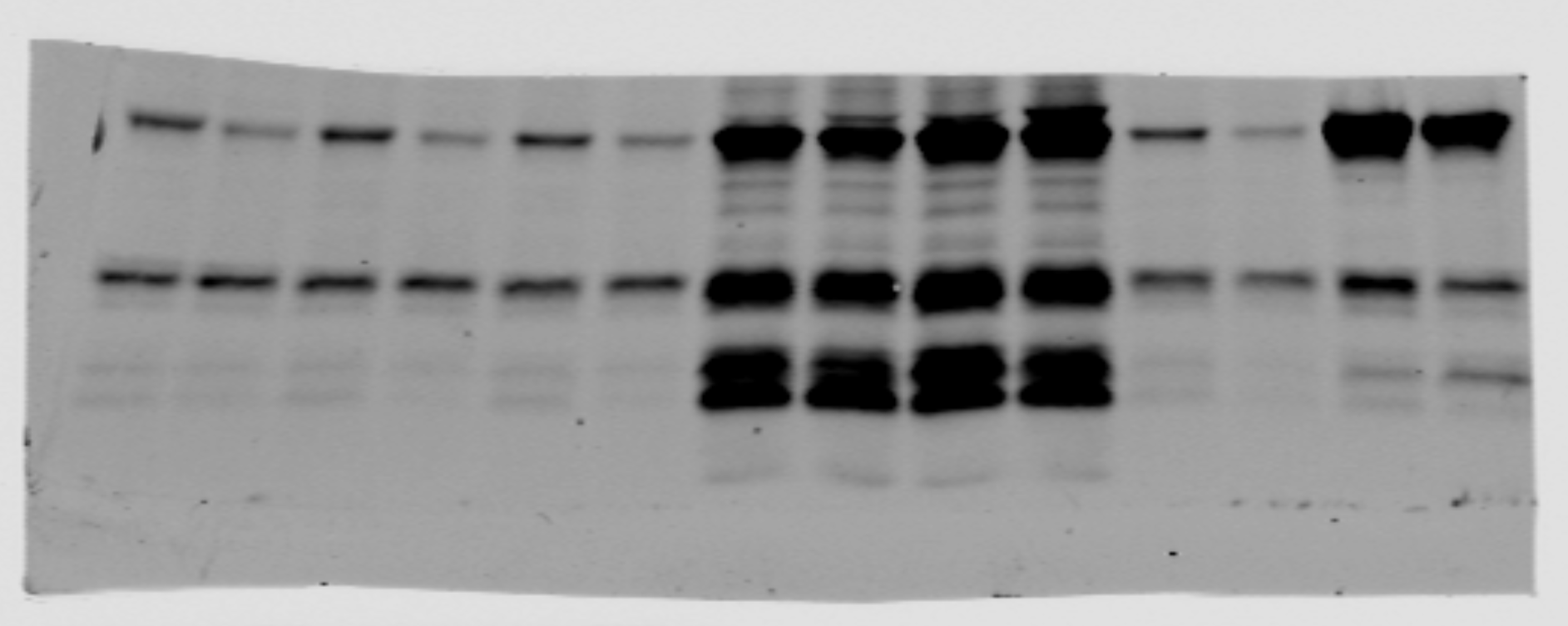

Supplement: Figure 3—figure supplement 1—source data 1. [file elife-82843-fig3-figsupp1-data1.zip › Fig. 3-1A V5.tif]

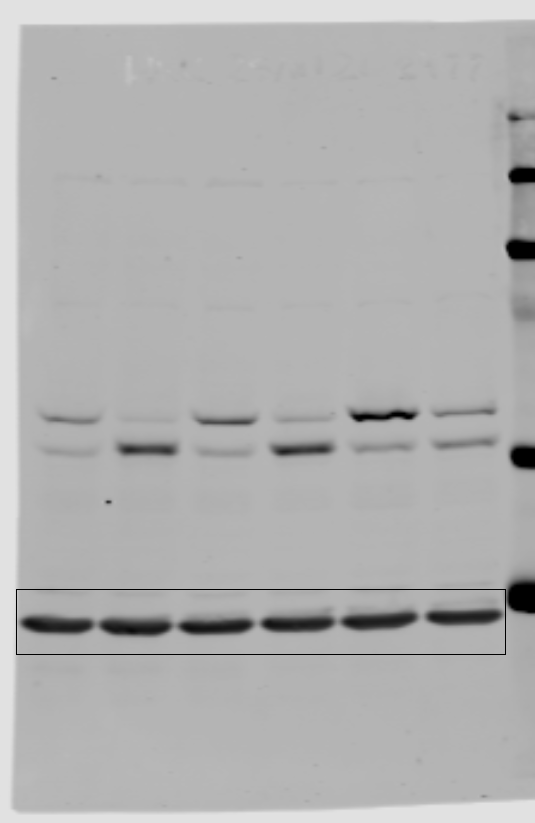

Supplement: Figure 4—source data 1. [file elife-82843-fig4-data1.zip › Annotated/Fig. 4A GAPDH.tif]

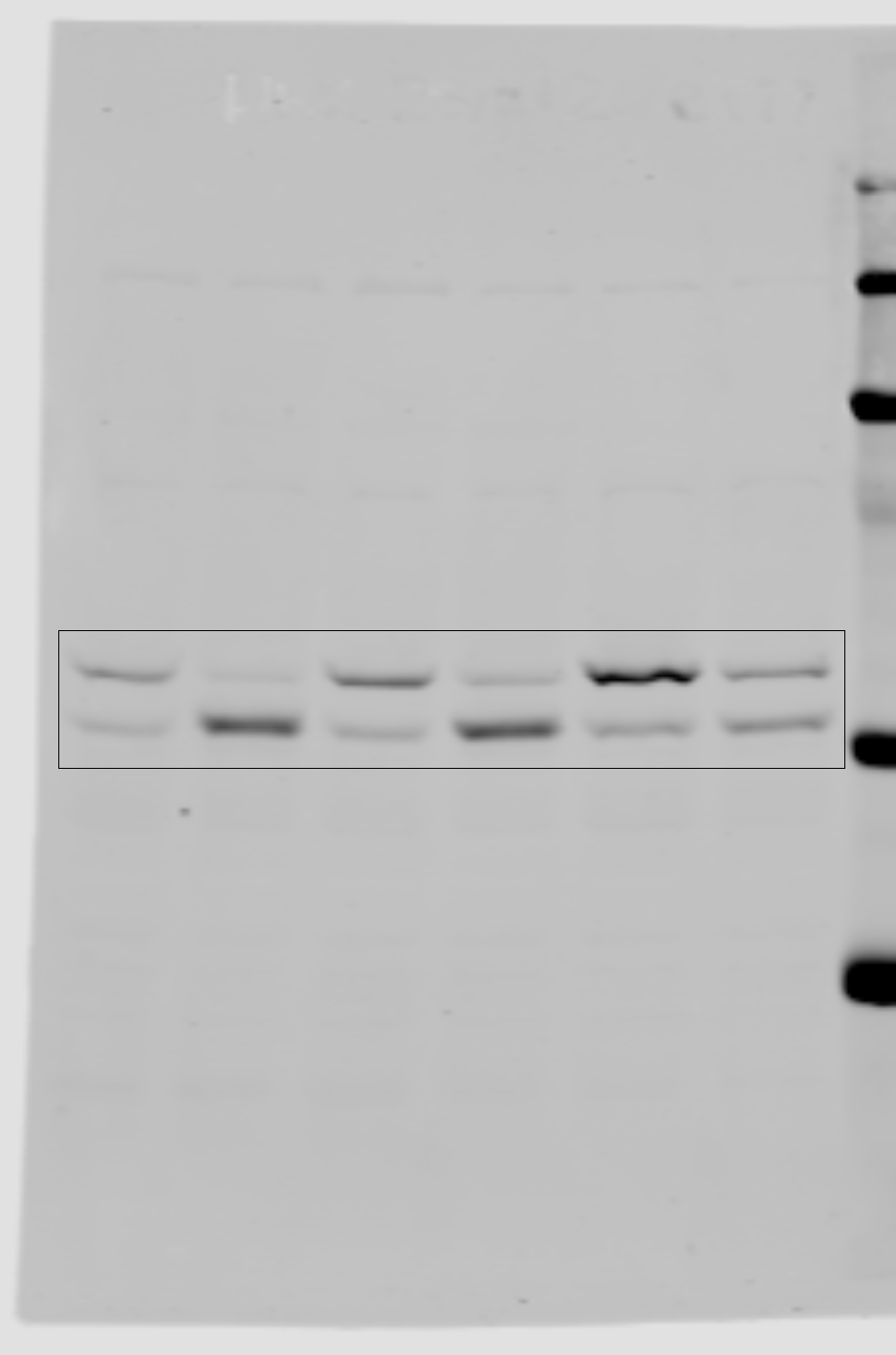

Supplement: Figure 4—source data 1. [file elife-82843-fig4-data1.zip › Annotated/Fig. 4A SM.tif]

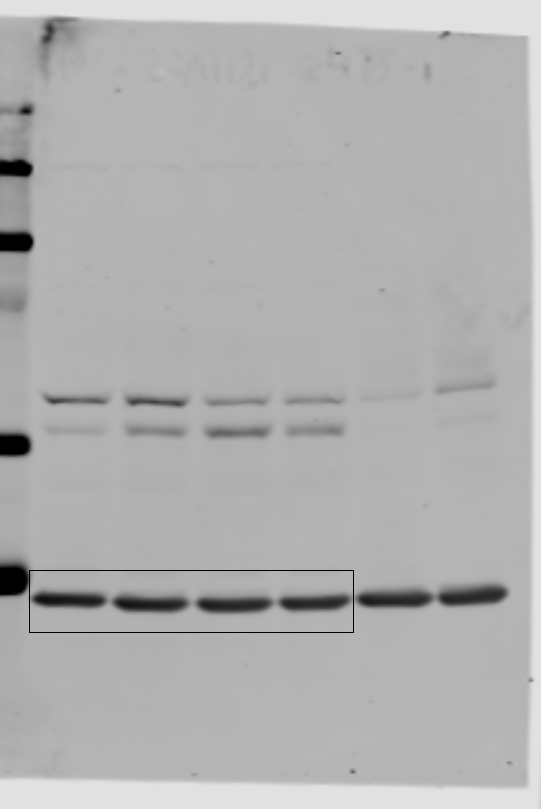

Supplement: Figure 4—source data 1. [file elife-82843-fig4-data1.zip › Annotated/Fig. 4D GAPDH.tif]

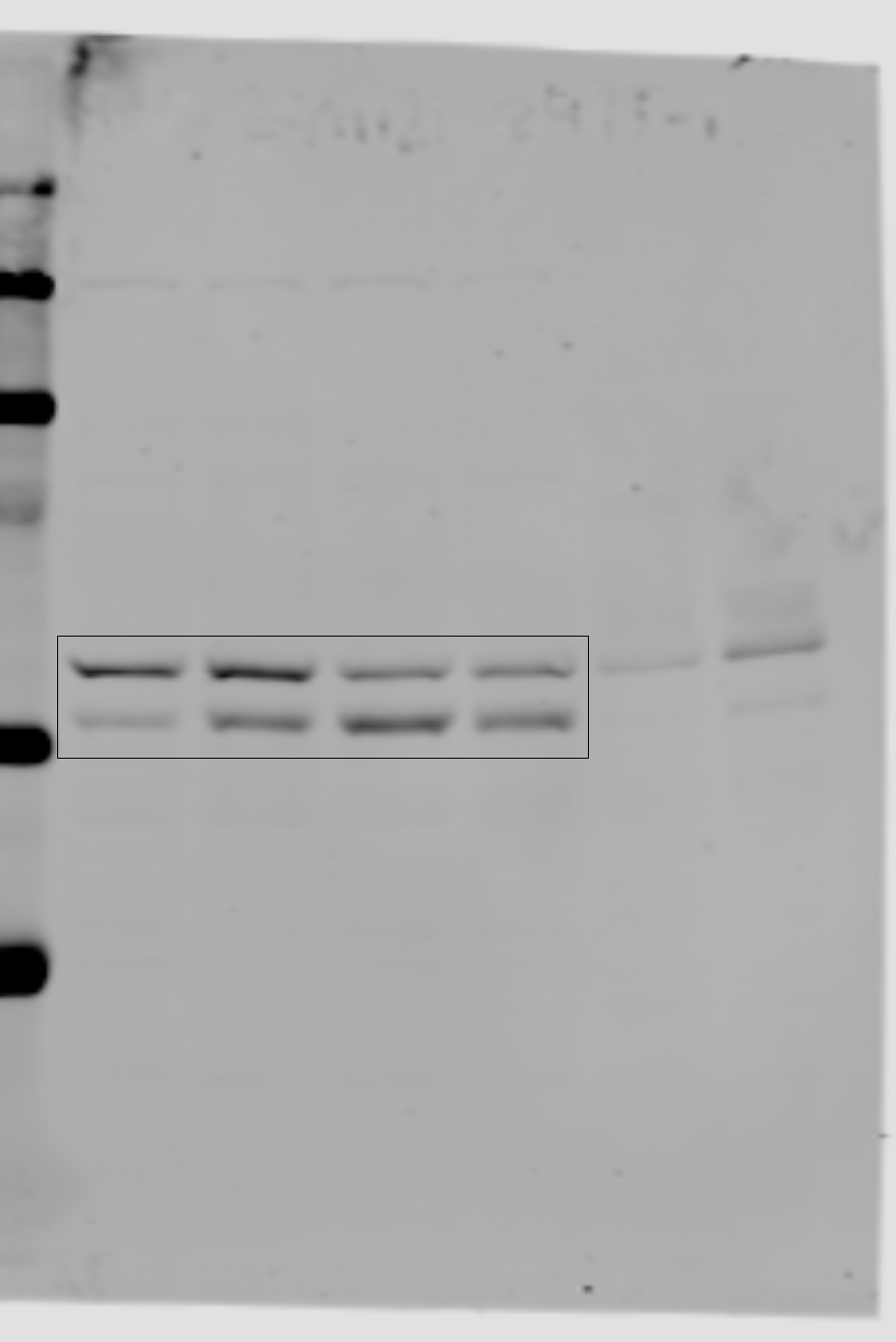

Supplement: Figure 4—source data 1. [file elife-82843-fig4-data1.zip › Annotated/Fig. 4D SM.tif]

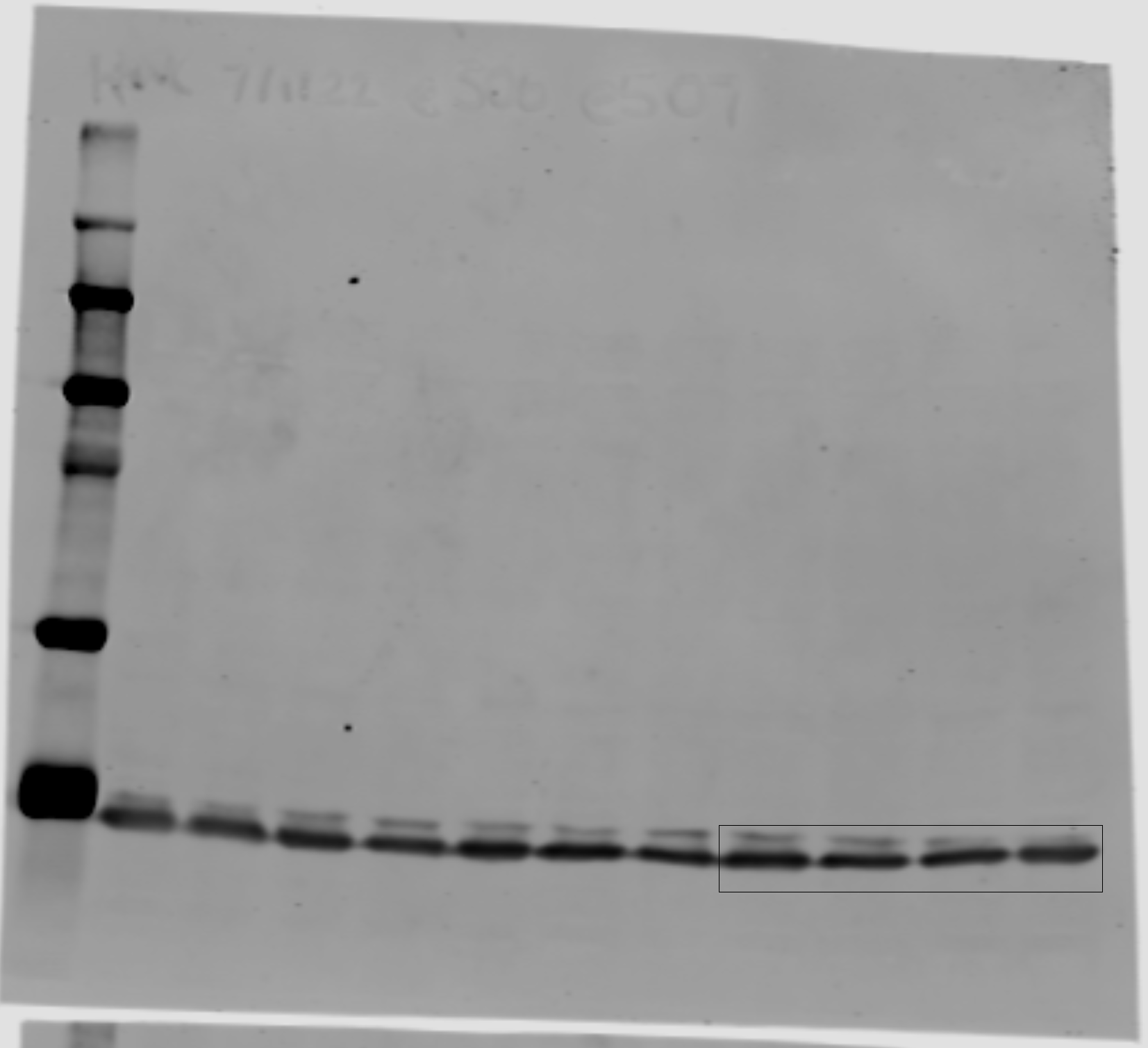

Supplement: Figure 4—source data 1. [file elife-82843-fig4-data1.zip › Annotated/Fig. 4E GAPDH.tif]

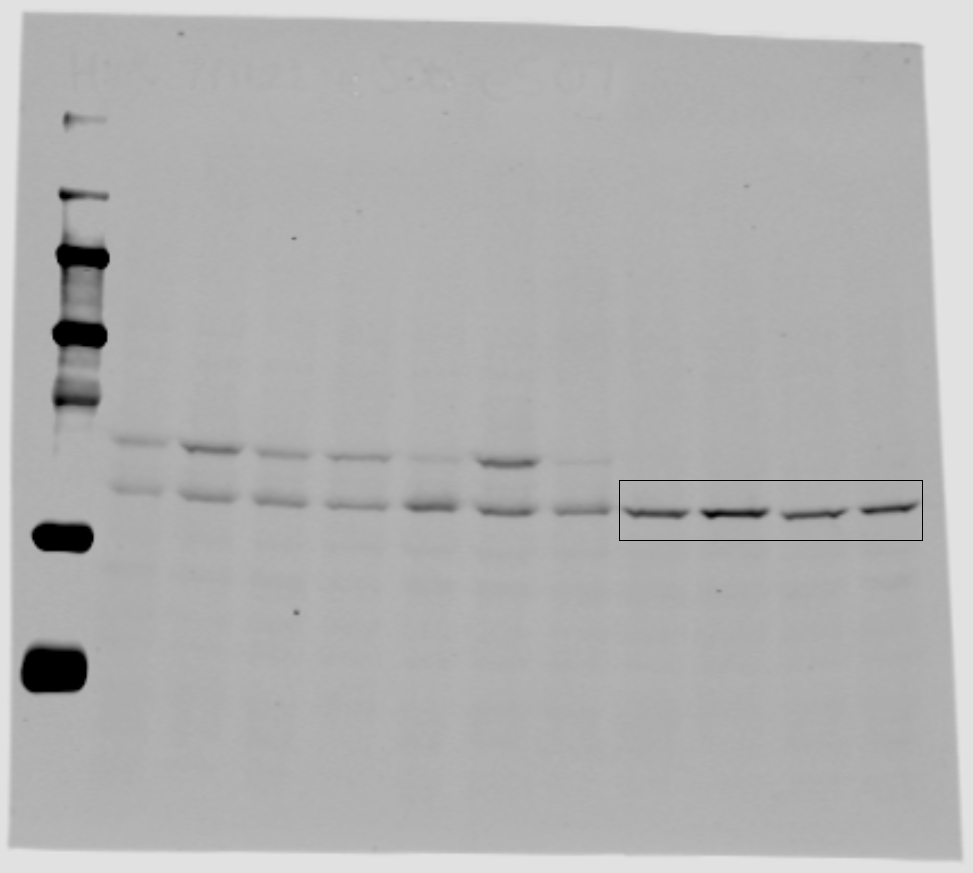

Supplement: Figure 4—source data 1. [file elife-82843-fig4-data1.zip › Annotated/Fig. 4E V5.tif]

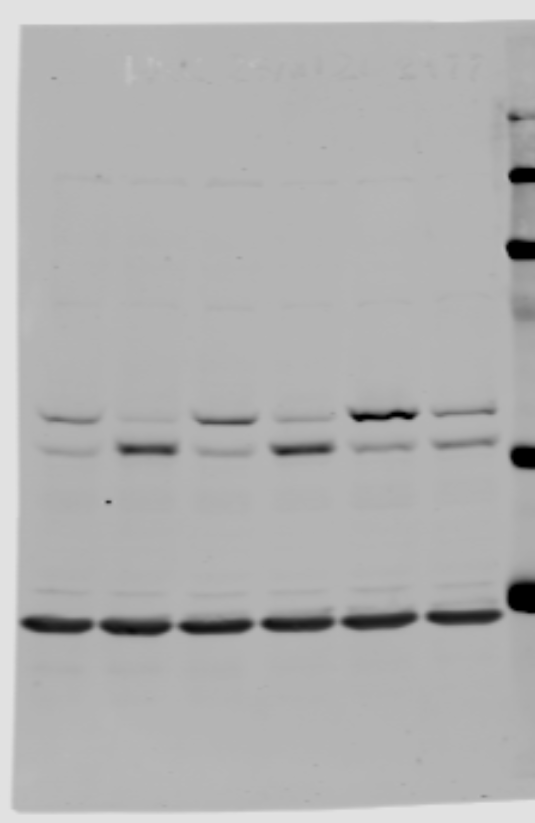

Supplement: Figure 4—source data 1. [file elife-82843-fig4-data1.zip › Fig. 4A GAPDH.tif]

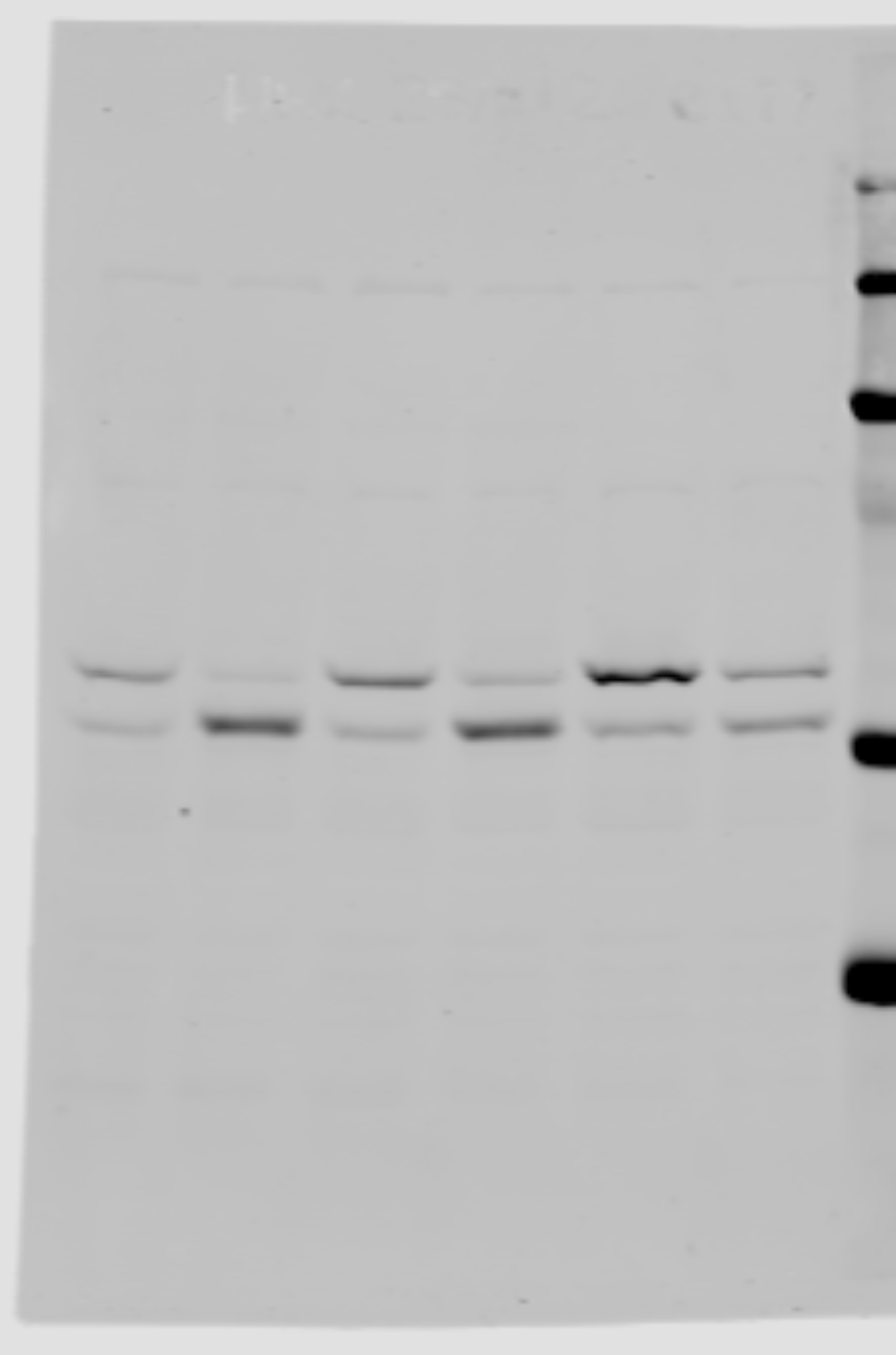

Supplement: Figure 4—source data 1. [file elife-82843-fig4-data1.zip › Fig. 4A SM.tif]

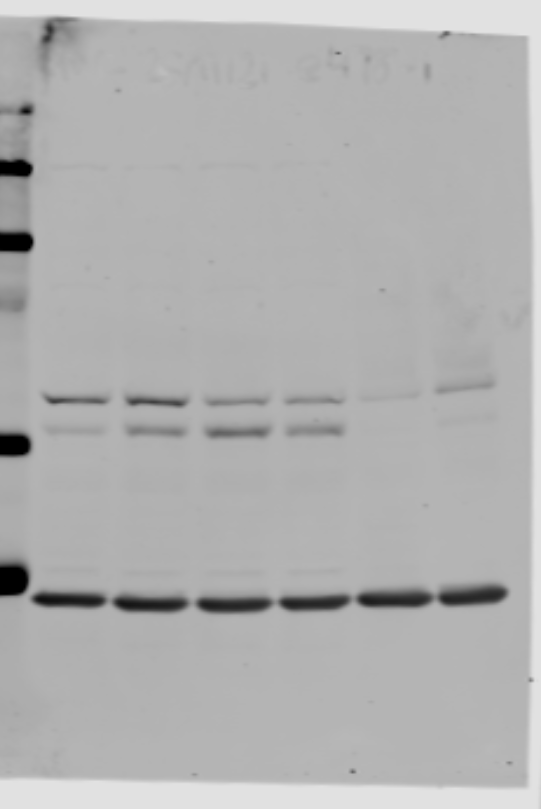

Supplement: Figure 4—source data 1. [file elife-82843-fig4-data1.zip › Fig. 4D GAPDH.tif]

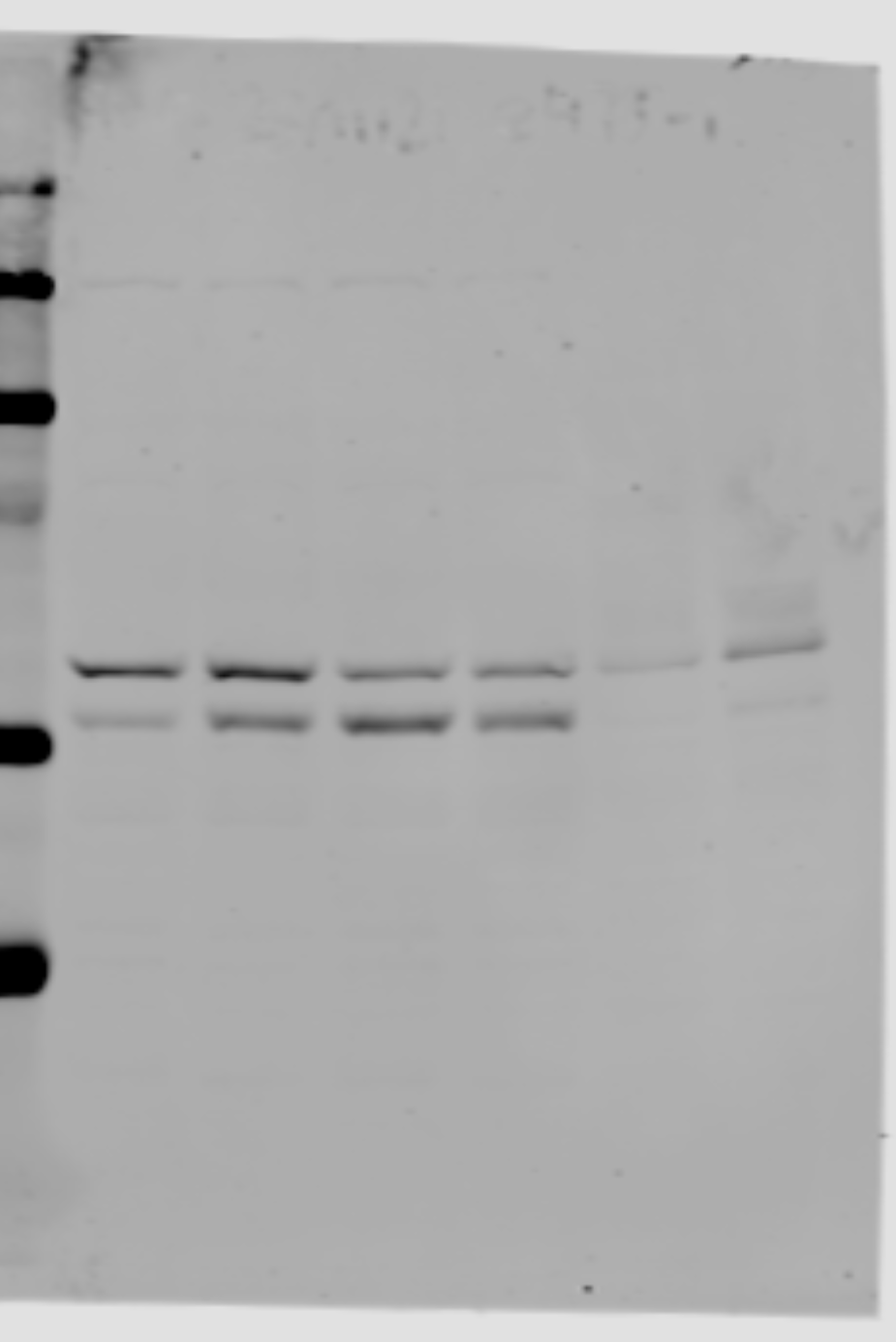

Supplement: Figure 4—source data 1. [file elife-82843-fig4-data1.zip › Fig. 4D SM.tif]

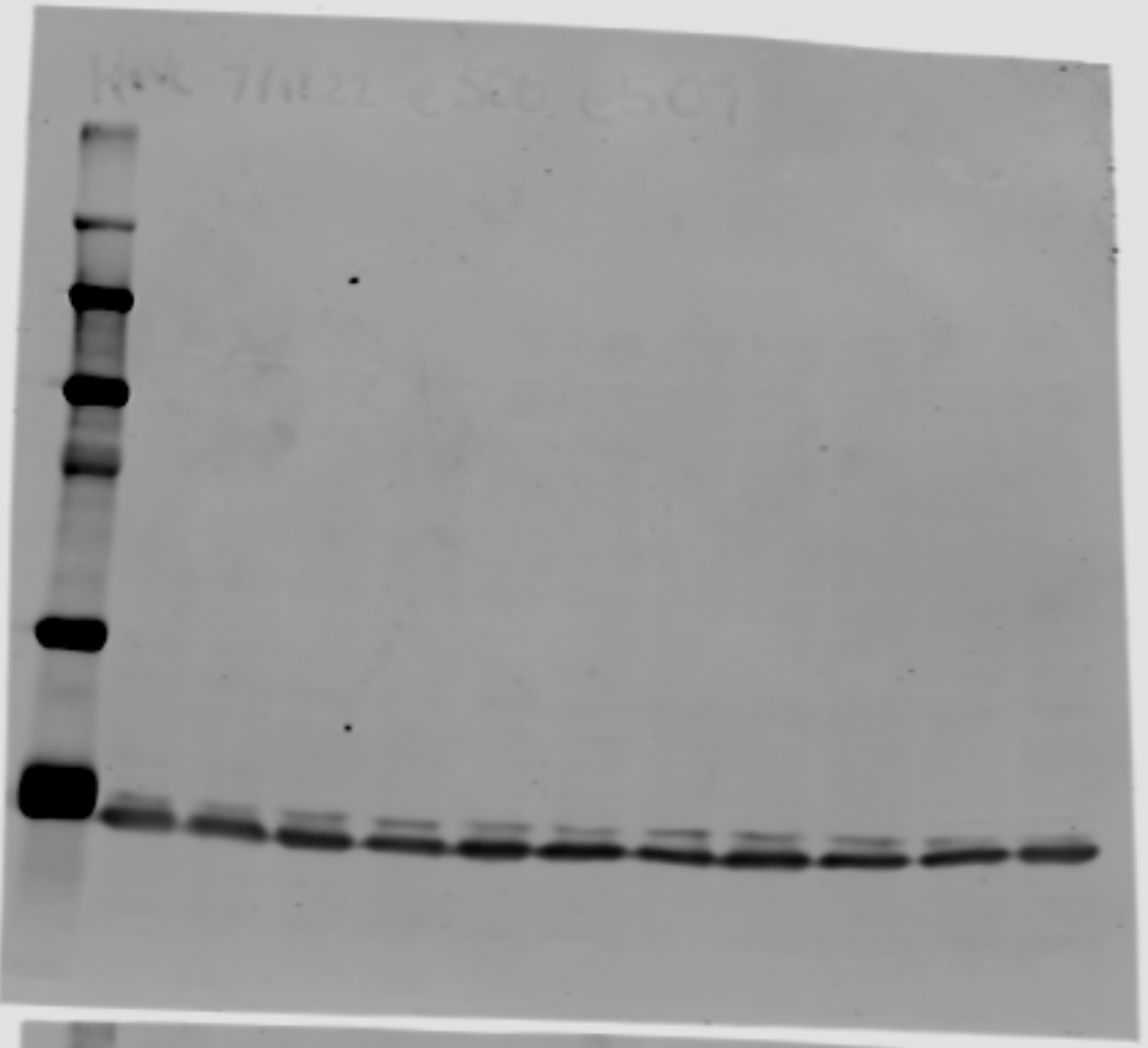

Supplement: Figure 4—source data 1. [file elife-82843-fig4-data1.zip › Fig. 4E GAPDH.tif]

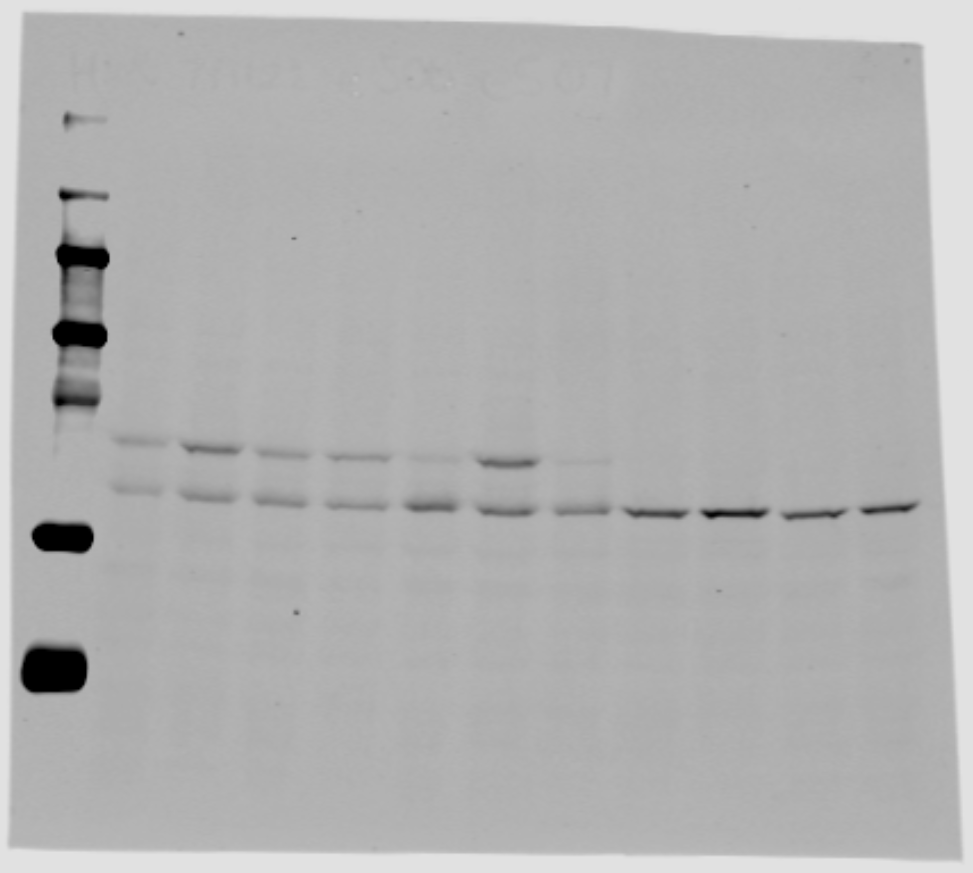

Supplement: Figure 4—source data 1. [file elife-82843-fig4-data1.zip › Fig. 4E V5.tif]

**Figure 4A – SM**

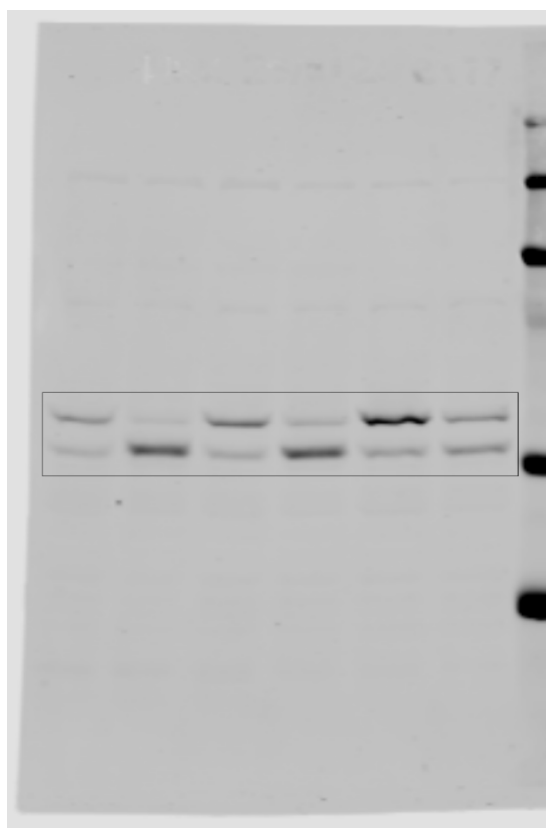

**Figure 4A – GAPDH**

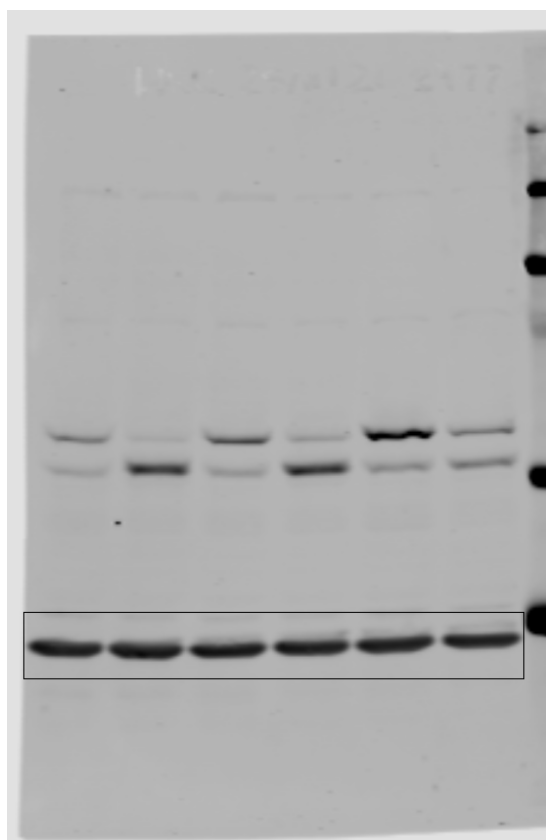

**Figure 4D – SM**

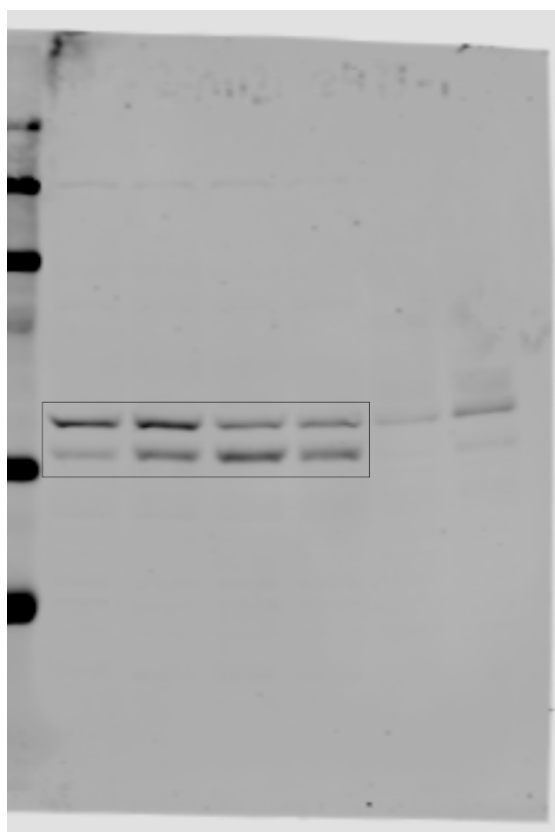

**Figure 4D – GAPDH**

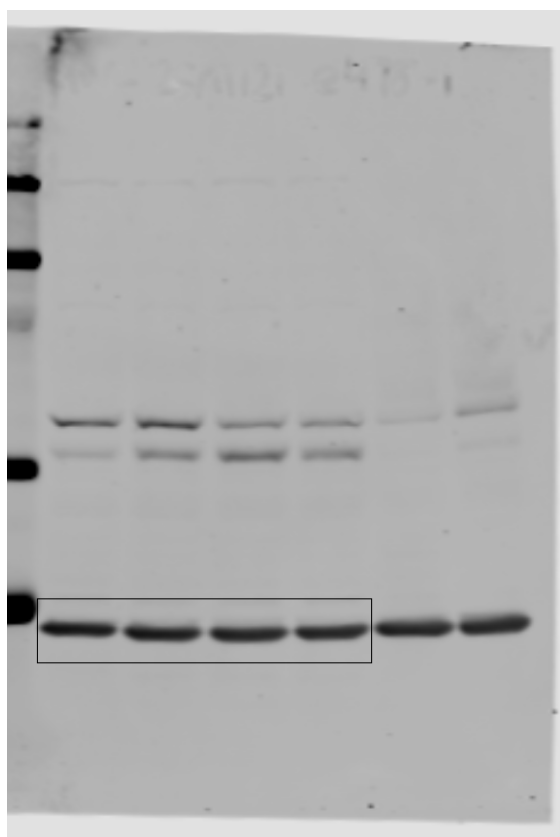

**Figure 4E – V5**

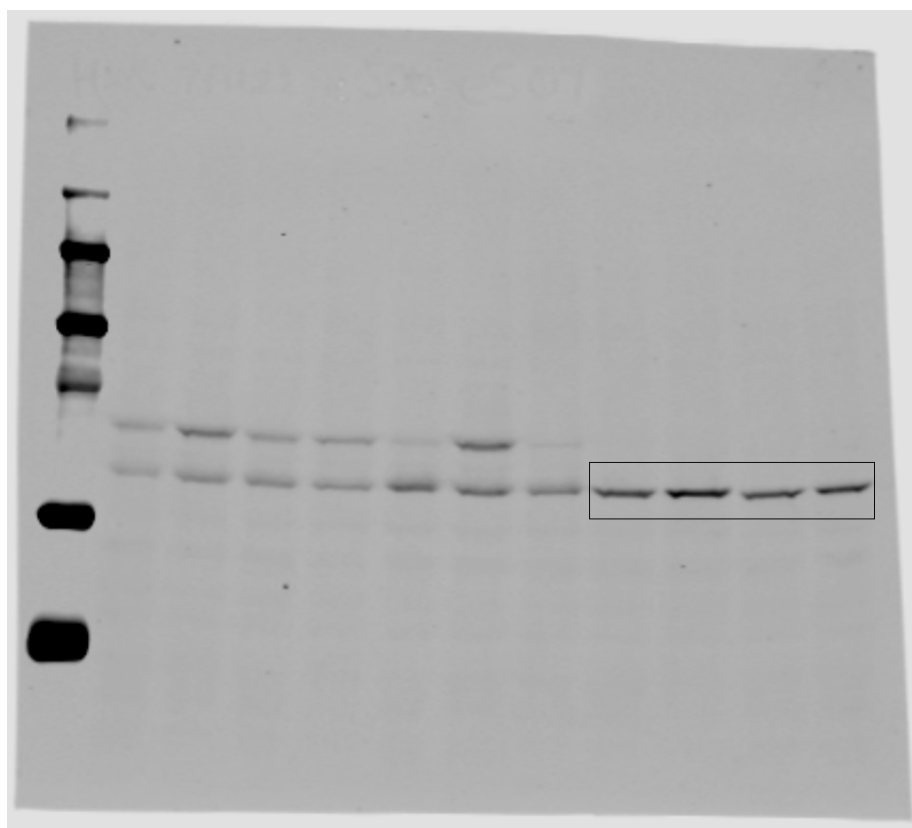

**Figure 4E – GAPDH**

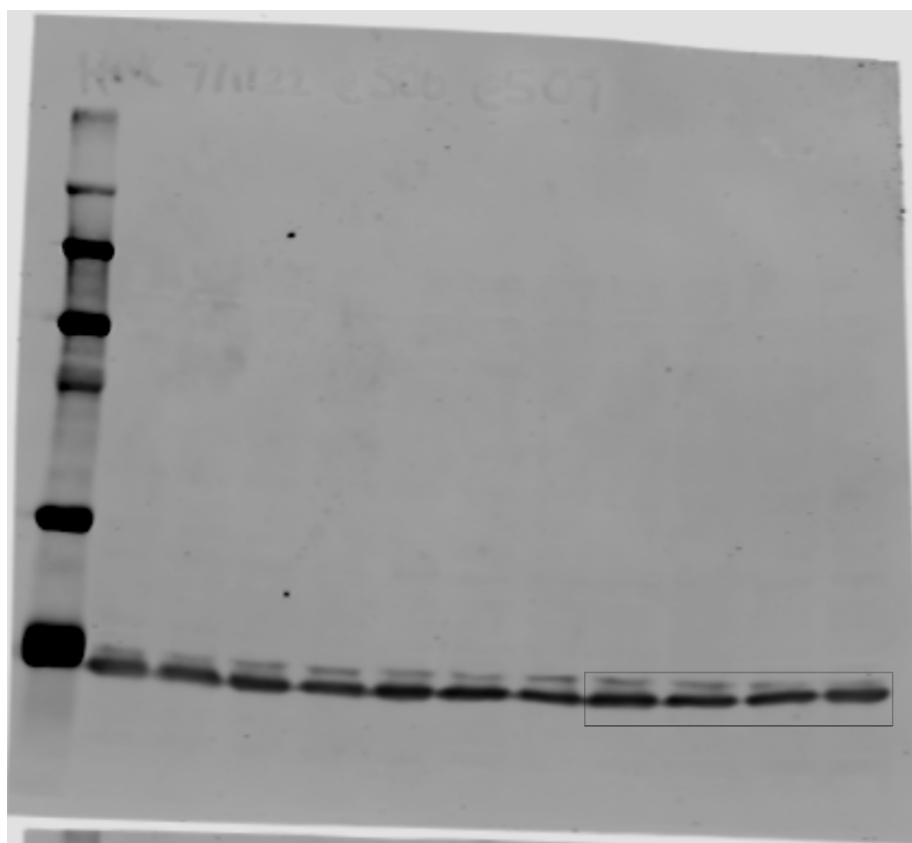

Supplement: Figure 4—source data 1. [file elife-82843-fig4-data1.zip › Figure 4-annotated source data.pdf]

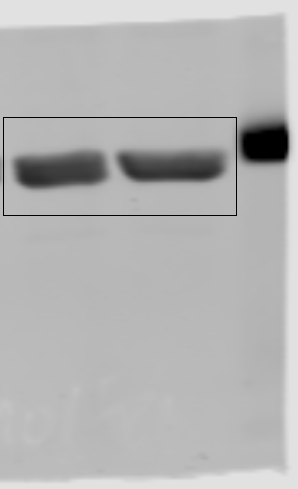

Supplement: Figure 4—figure supplement 2—source data 1. [file elife-82843-fig4-figsupp2-data1.zip › Annotated/Fig. 4-2B GAPDH.tif]

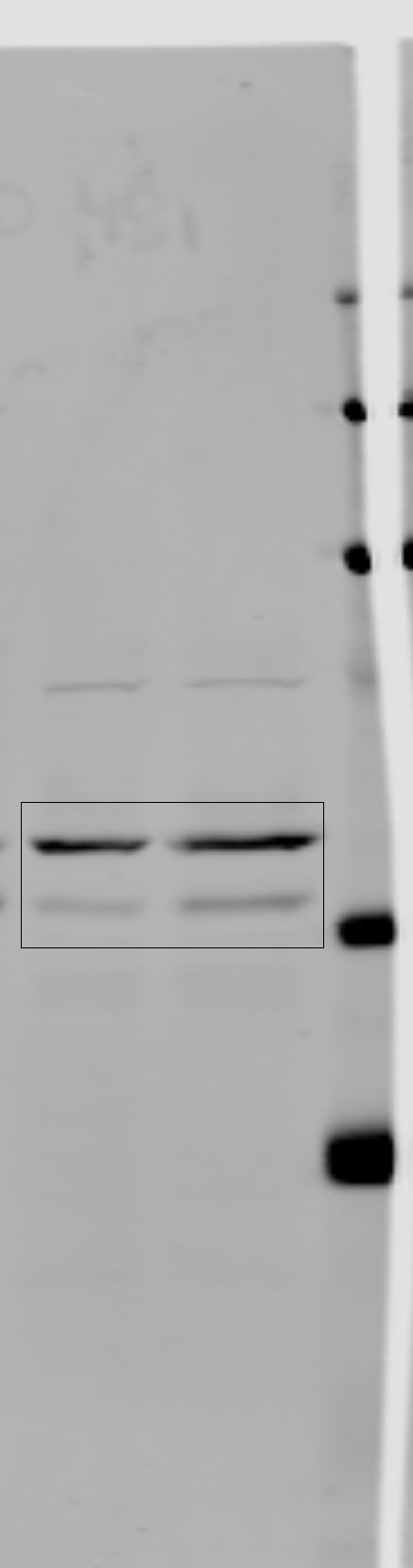

Supplement: Figure 4—figure supplement 2—source data 1. [file elife-82843-fig4-figsupp2-data1.zip › Annotated/Fig. 4-2B SM.tif]

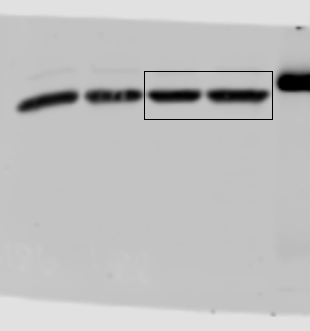

Supplement: Figure 4—figure supplement 2—source data 1. [file elife-82843-fig4-figsupp2-data1.zip › Annotated/Fig. 4-2C GAPDH.tif]

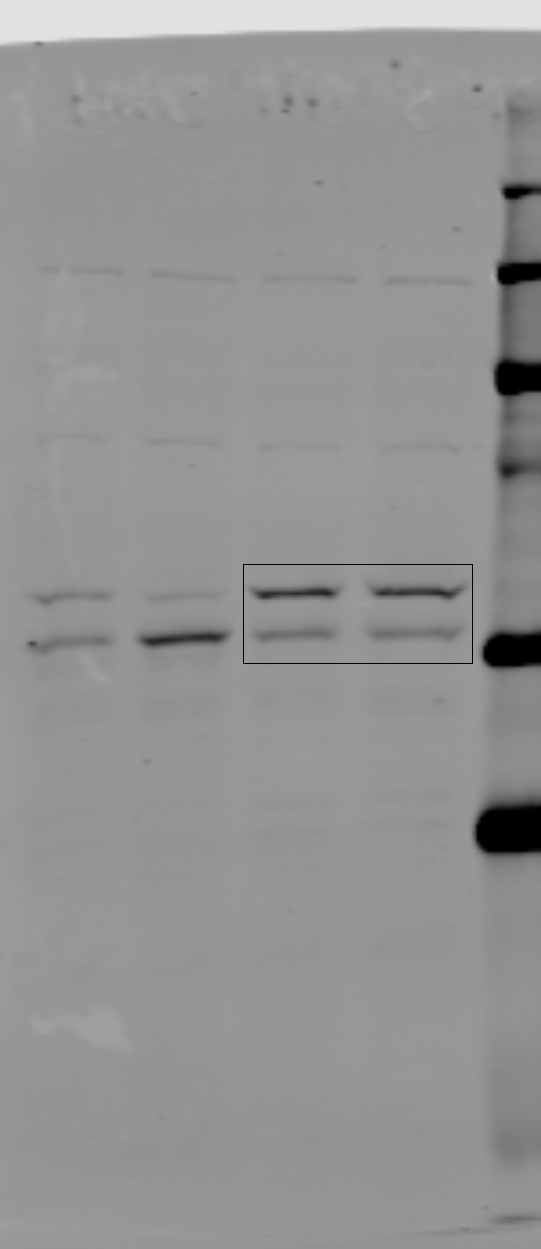

Supplement: Figure 4—figure supplement 2—source data 1. [file elife-82843-fig4-figsupp2-data1.zip › Annotated/Fig. 4-2C SM.tif]

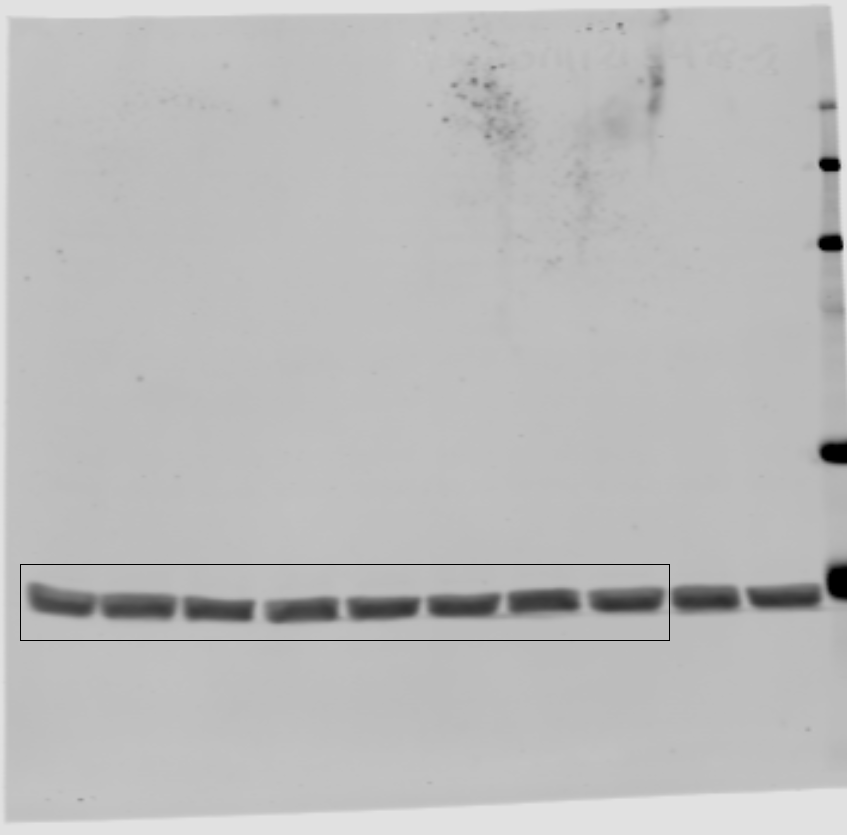

Supplement: Figure 4—figure supplement 2—source data 1. [file elife-82843-fig4-figsupp2-data1.zip › Annotated/Fig. 4-2D GAPDH.tif]

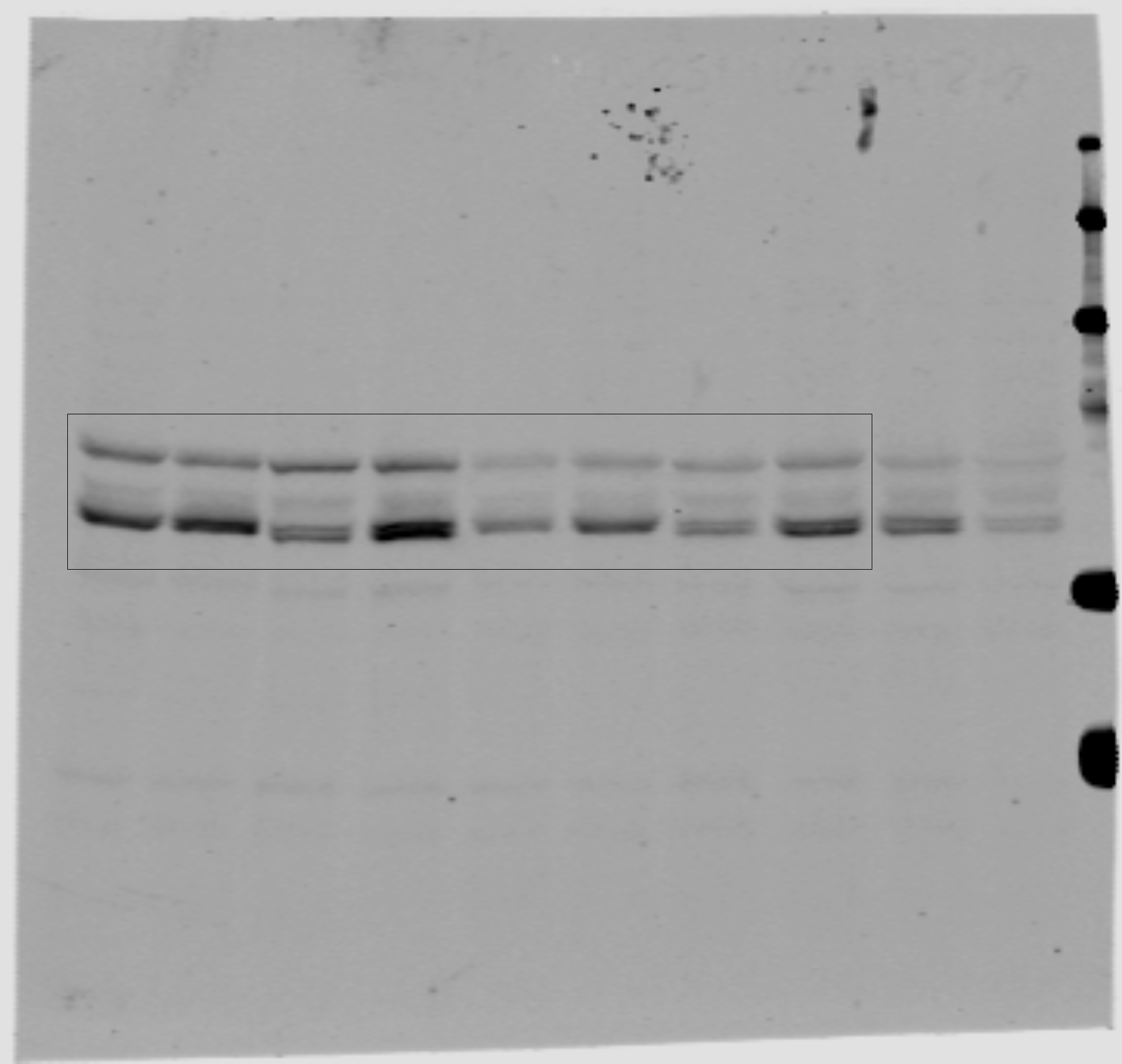

Supplement: Figure 4—figure supplement 2—source data 1. [file elife-82843-fig4-figsupp2-data1.zip › Annotated/Fig. 4-2D V5.tif]

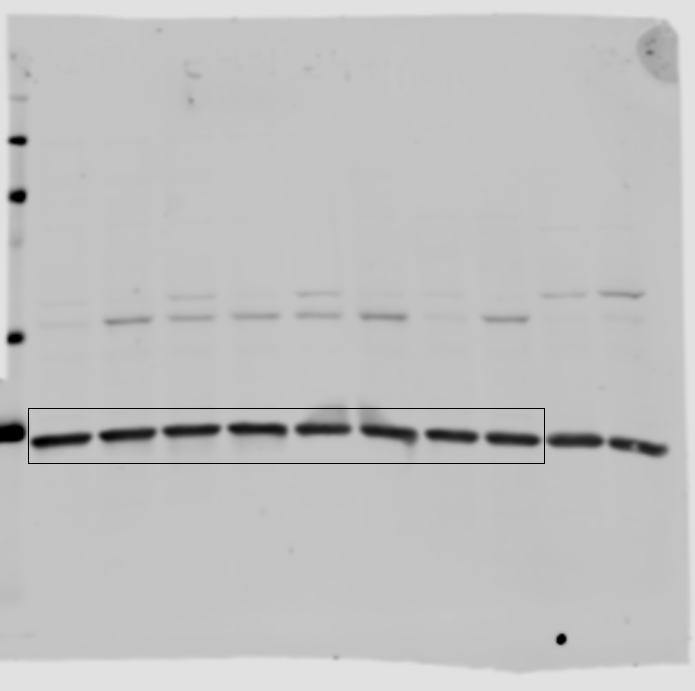

Supplement: Figure 4—figure supplement 2—source data 1. [file elife-82843-fig4-figsupp2-data1.zip › Annotated/Fig. 4-2E GAPDH.tif]

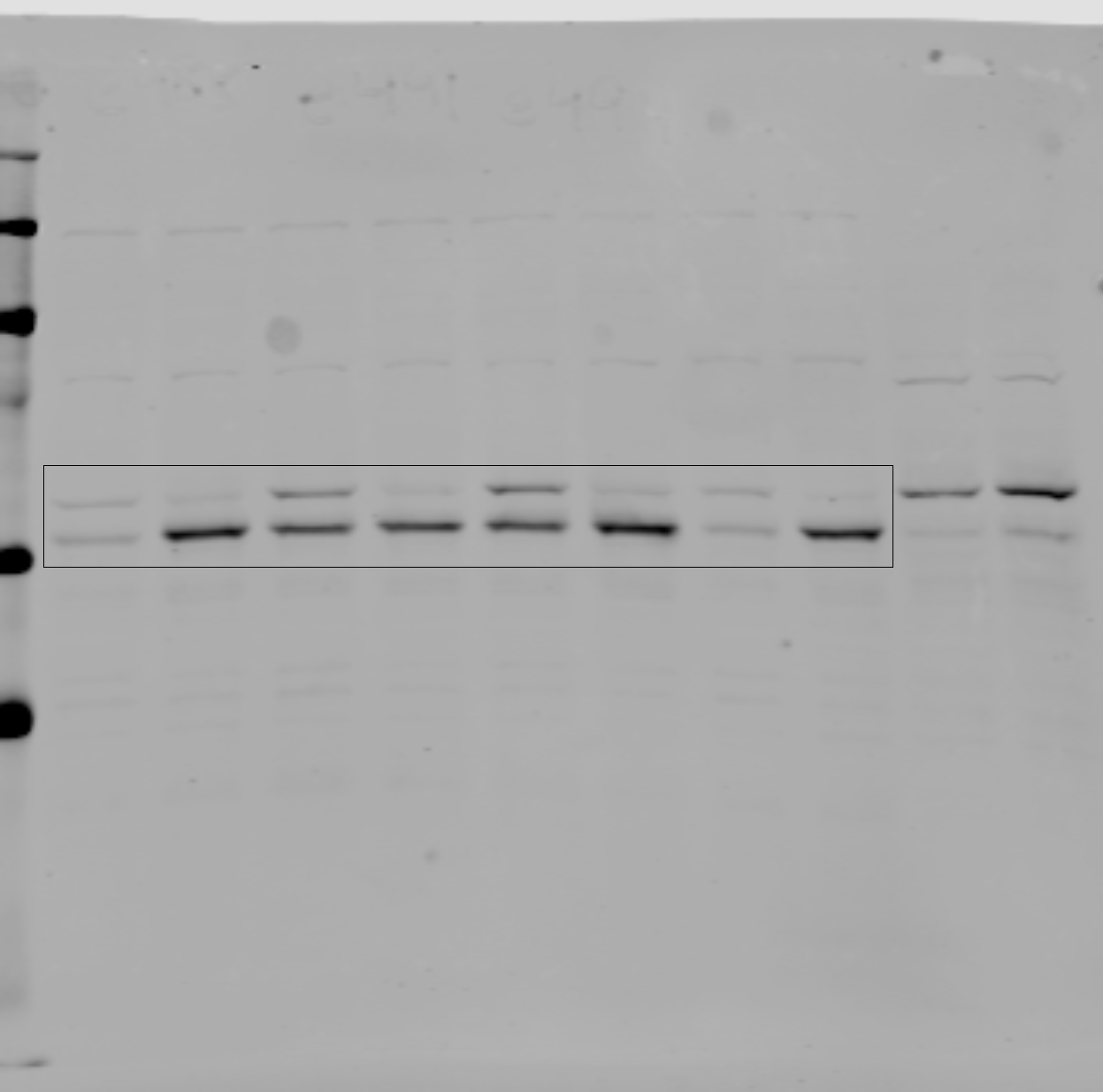

Supplement: Figure 4—figure supplement 2—source data 1. [file elife-82843-fig4-figsupp2-data1.zip › Annotated/Fig. 4-2E SM.tif]

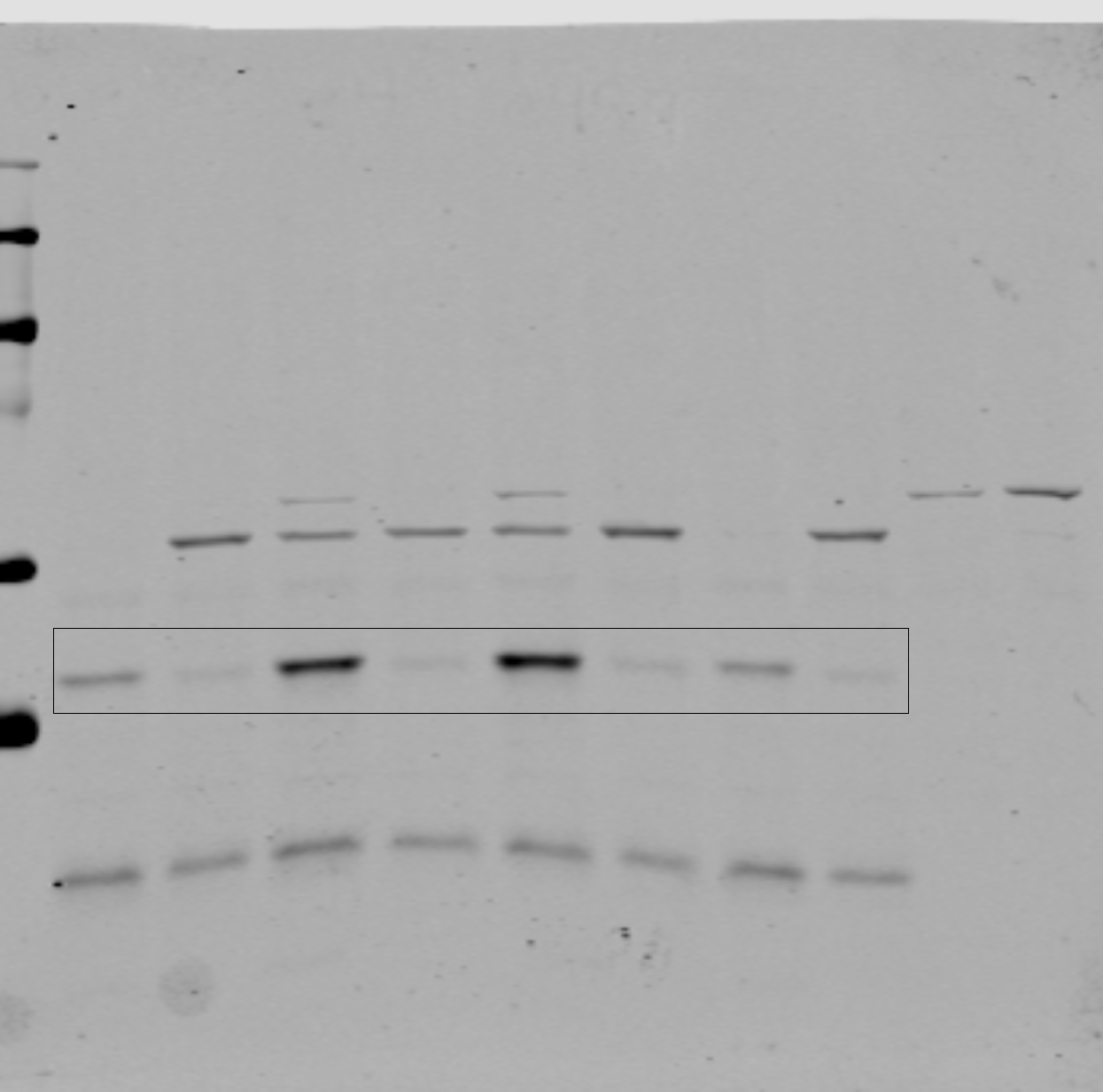

Supplement: Figure 4—figure supplement 2—source data 1. [file elife-82843-fig4-figsupp2-data1.zip › Annotated/Fig. 4-2E V5.tif]

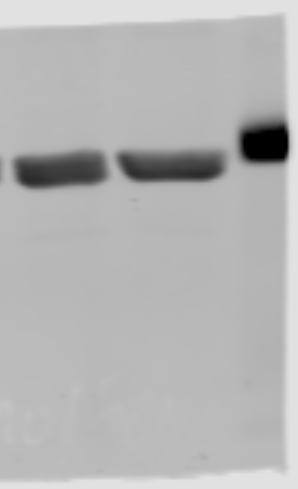

Supplement: Figure 4—figure supplement 2—source data 1. [file elife-82843-fig4-figsupp2-data1.zip › Fig. 4-2B GAPDH.tif]

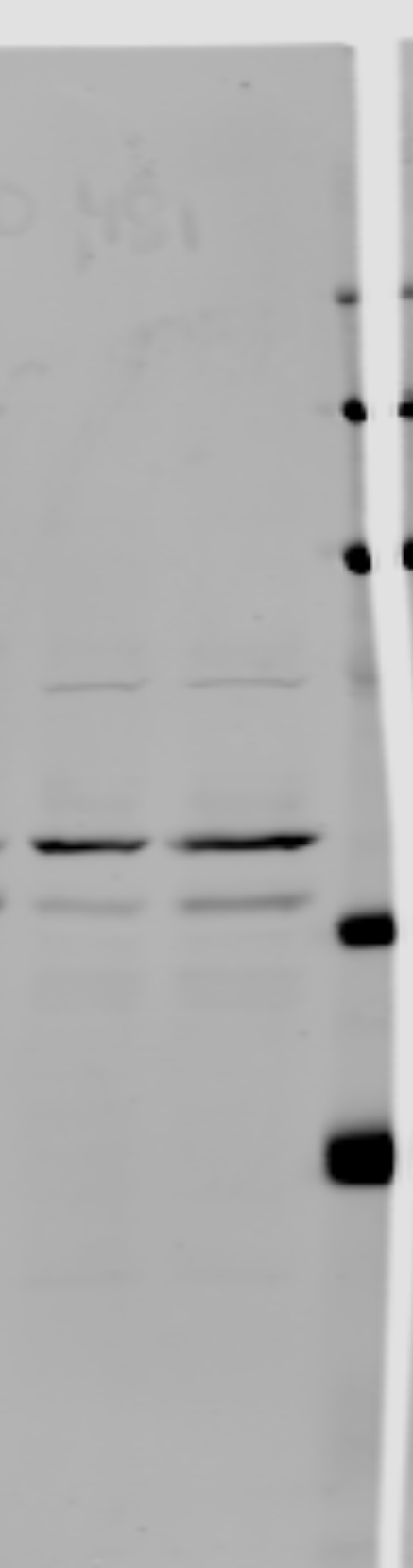

Supplement: Figure 4—figure supplement 2—source data 1. [file elife-82843-fig4-figsupp2-data1.zip › Fig. 4-2B SM.tif]

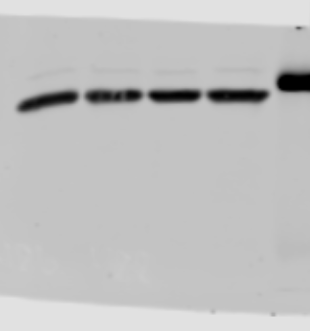

Supplement: Figure 4—figure supplement 2—source data 1. [file elife-82843-fig4-figsupp2-data1.zip › Fig. 4-2C GAPDH.tif]

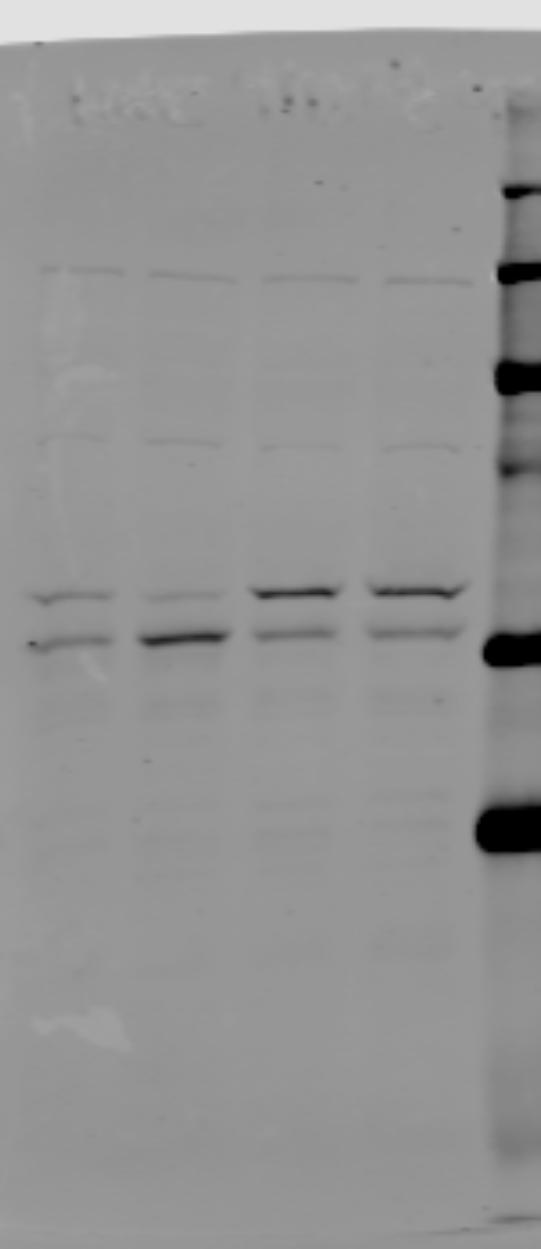

Supplement: Figure 4—figure supplement 2—source data 1. [file elife-82843-fig4-figsupp2-data1.zip › Fig. 4-2C SM.tif]

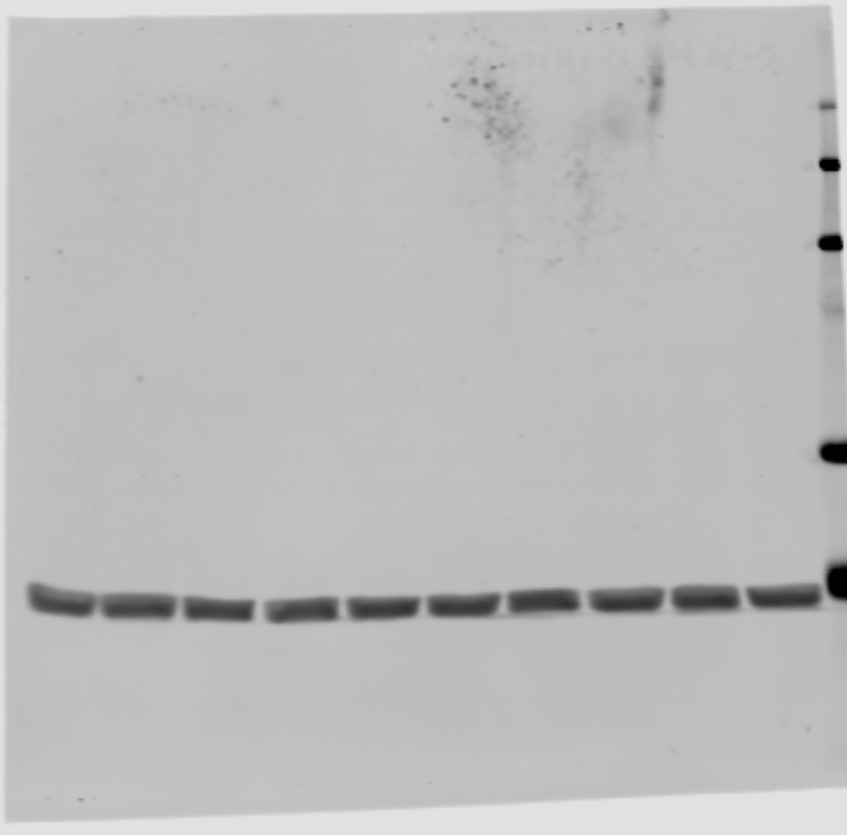

Supplement: Figure 4—figure supplement 2—source data 1. [file elife-82843-fig4-figsupp2-data1.zip › Fig. 4-2D GAPDH.tif]

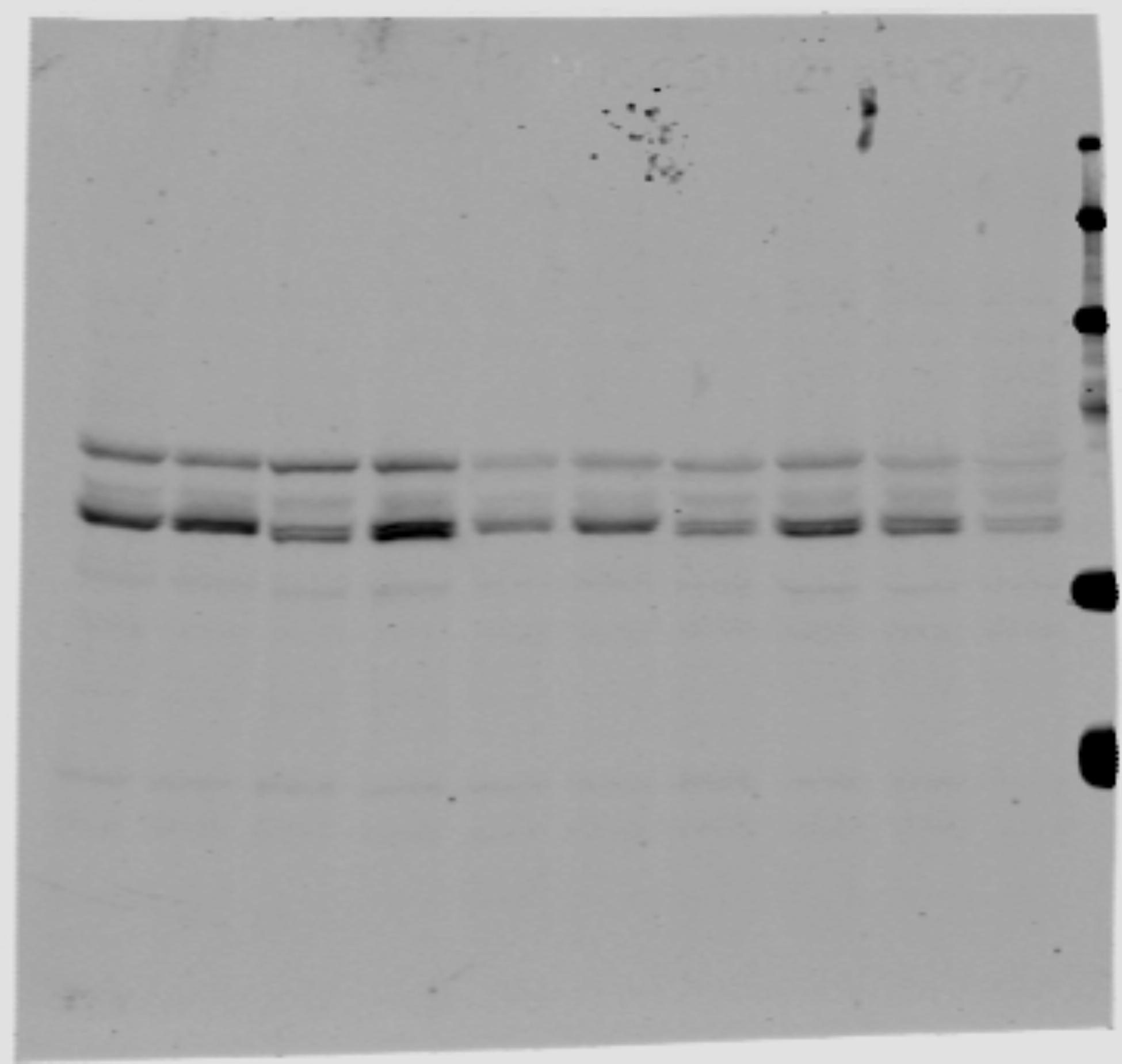

Supplement: Figure 4—figure supplement 2—source data 1. [file elife-82843-fig4-figsupp2-data1.zip › Fig. 4-2D V5.tif]

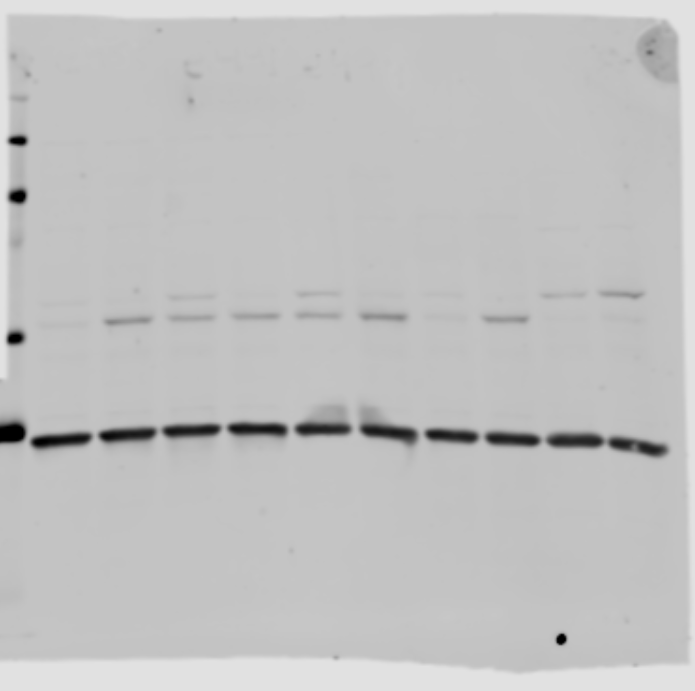

Supplement: Figure 4—figure supplement 2—source data 1. [file elife-82843-fig4-figsupp2-data1.zip › Fig. 4-2E GAPDH.tif]

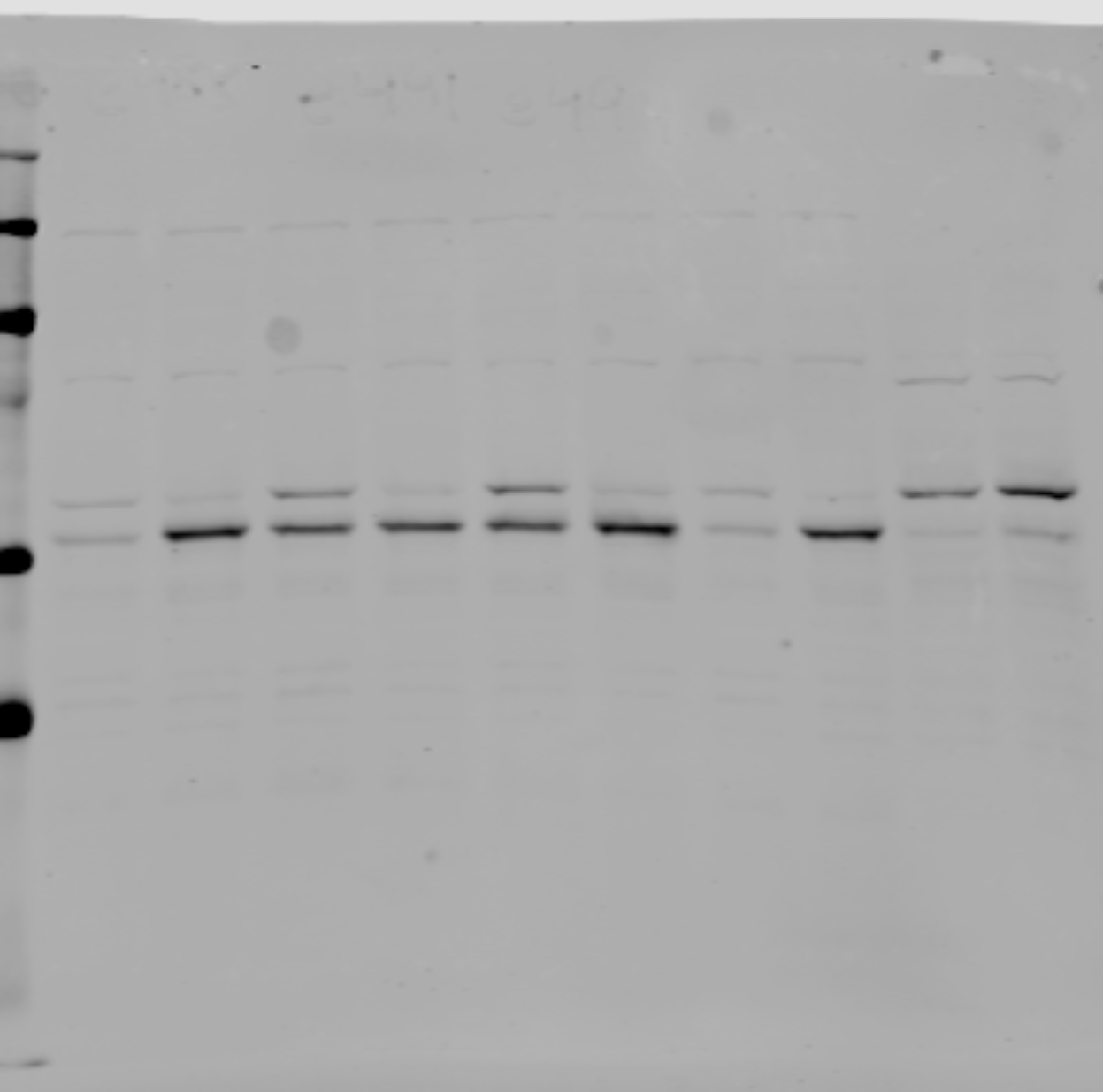

Supplement: Figure 4—figure supplement 2—source data 1. [file elife-82843-fig4-figsupp2-data1.zip › Fig. 4-2E SM.tif]

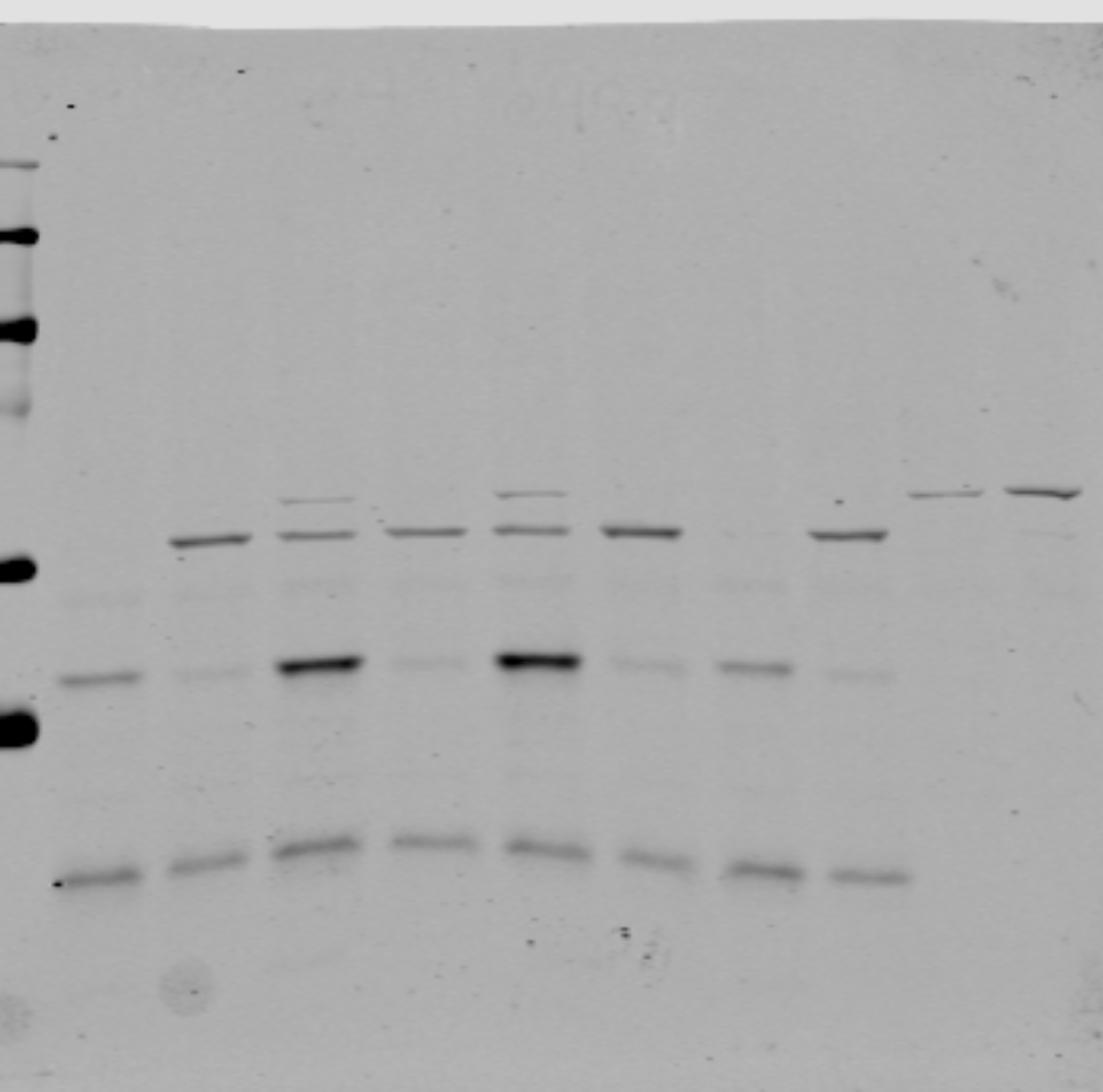

Supplement: Figure 4—figure supplement 2—source data 1. [file elife-82843-fig4-figsupp2-data1.zip › Fig. 4-2E V5.tif]

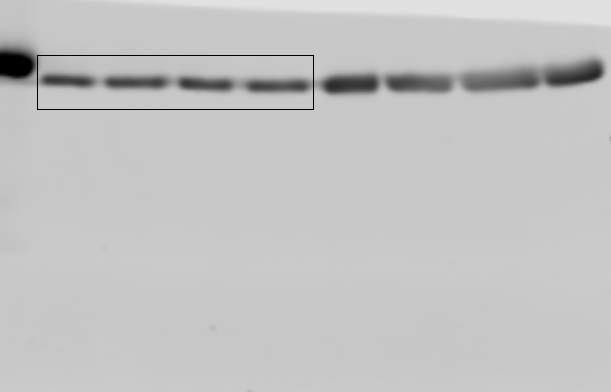

Supplement: Figure 4—figure supplement 3—source data 1. [file elife-82843-fig4-figsupp3-data1.zip › Annotated/Fig. 4-3C GAPDH.tif]

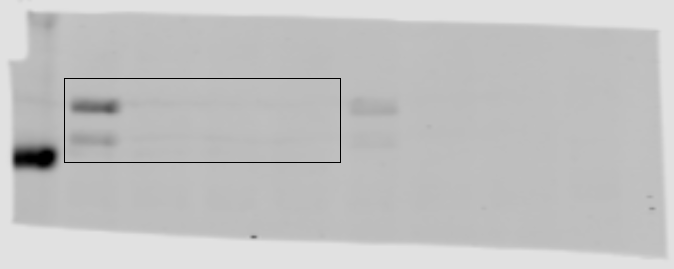

Supplement: Figure 4—figure supplement 3—source data 1. [file elife-82843-fig4-figsupp3-data1.zip › Annotated/Fig. 4-3C SM.tif]

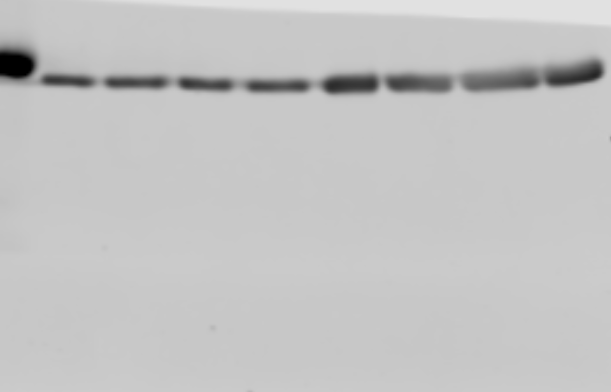

Supplement: Figure 4—figure supplement 3—source data 1. [file elife-82843-fig4-figsupp3-data1.zip › Fig. 4-3C GAPDH.tif]

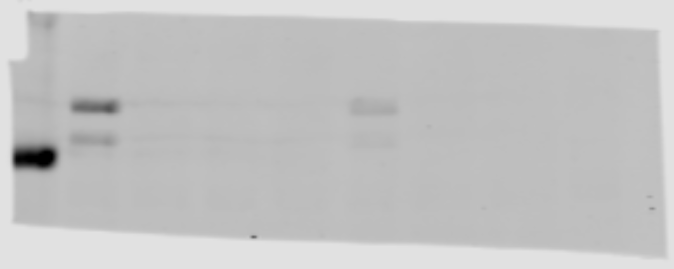

Supplement: Figure 4—figure supplement 3—source data 1. [file elife-82843-fig4-figsupp3-data1.zip › Fig. 4-3C SM.tif]

**Figure 4—figure supplement 3C – SM**

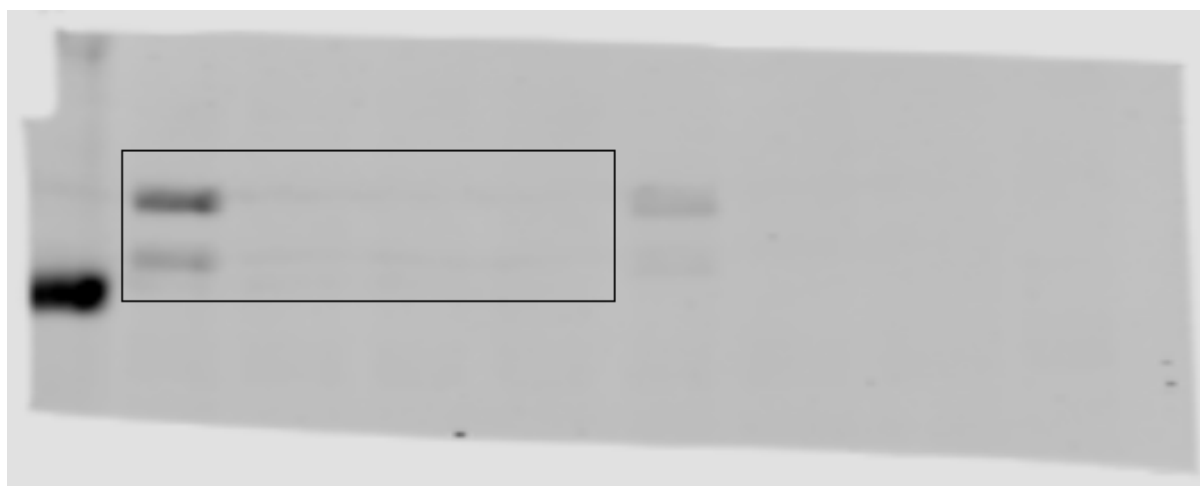

**Figure 4—figure supplement 3C – GAPDH**

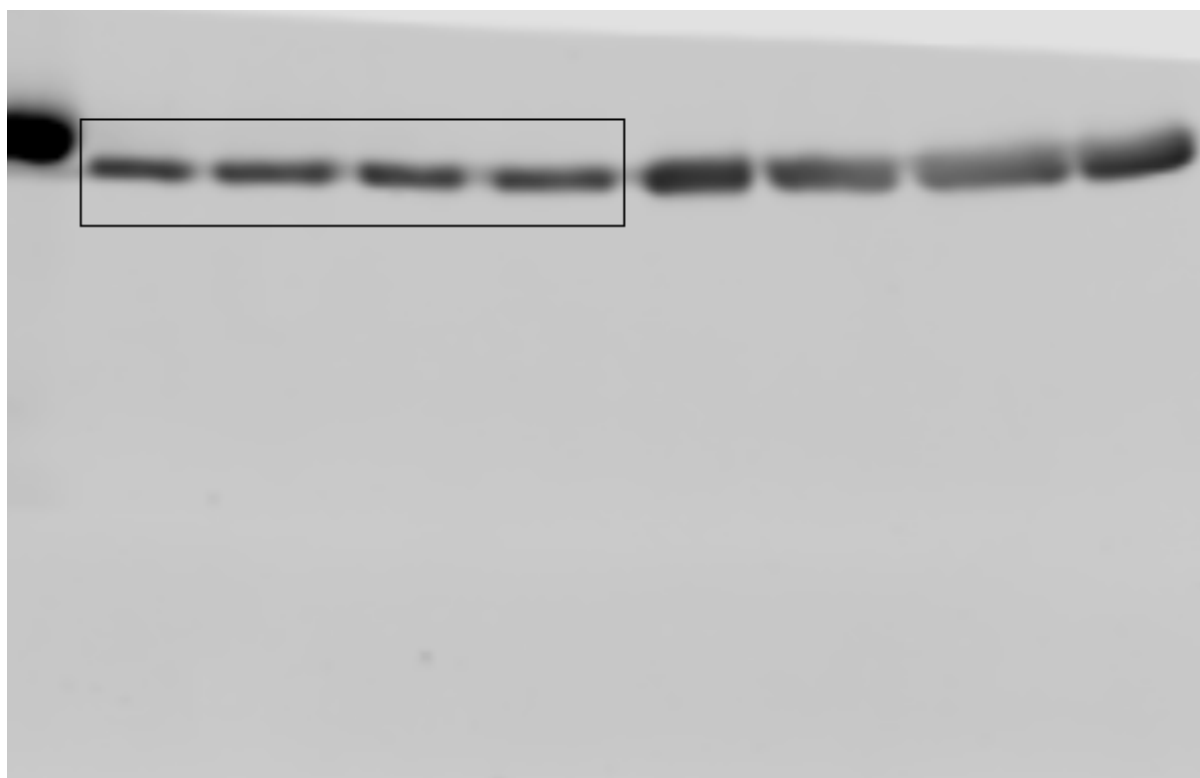

Supplement: Figure 4—figure supplement 3—source data 1. [file elife-82843-fig4-figsupp3-data1.zip › Figure 4-figure supplement 3-annotated source data.pdf]

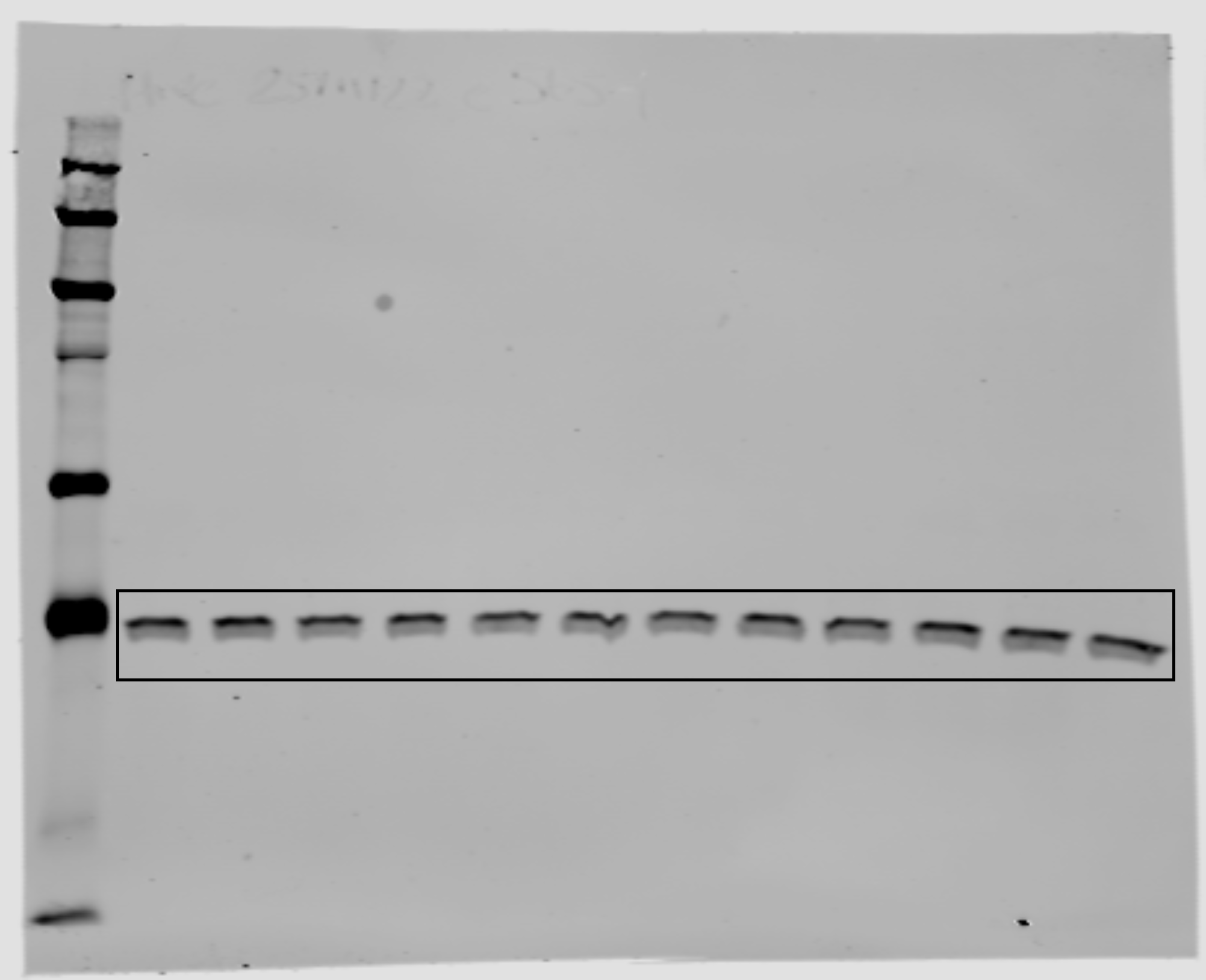

Supplement: Figure 4—figure supplement 4—source data 1. [file elife-82843-fig4-figsupp4-data1.zip › Annotated/Fig. 4-4 GAPDH.tif]

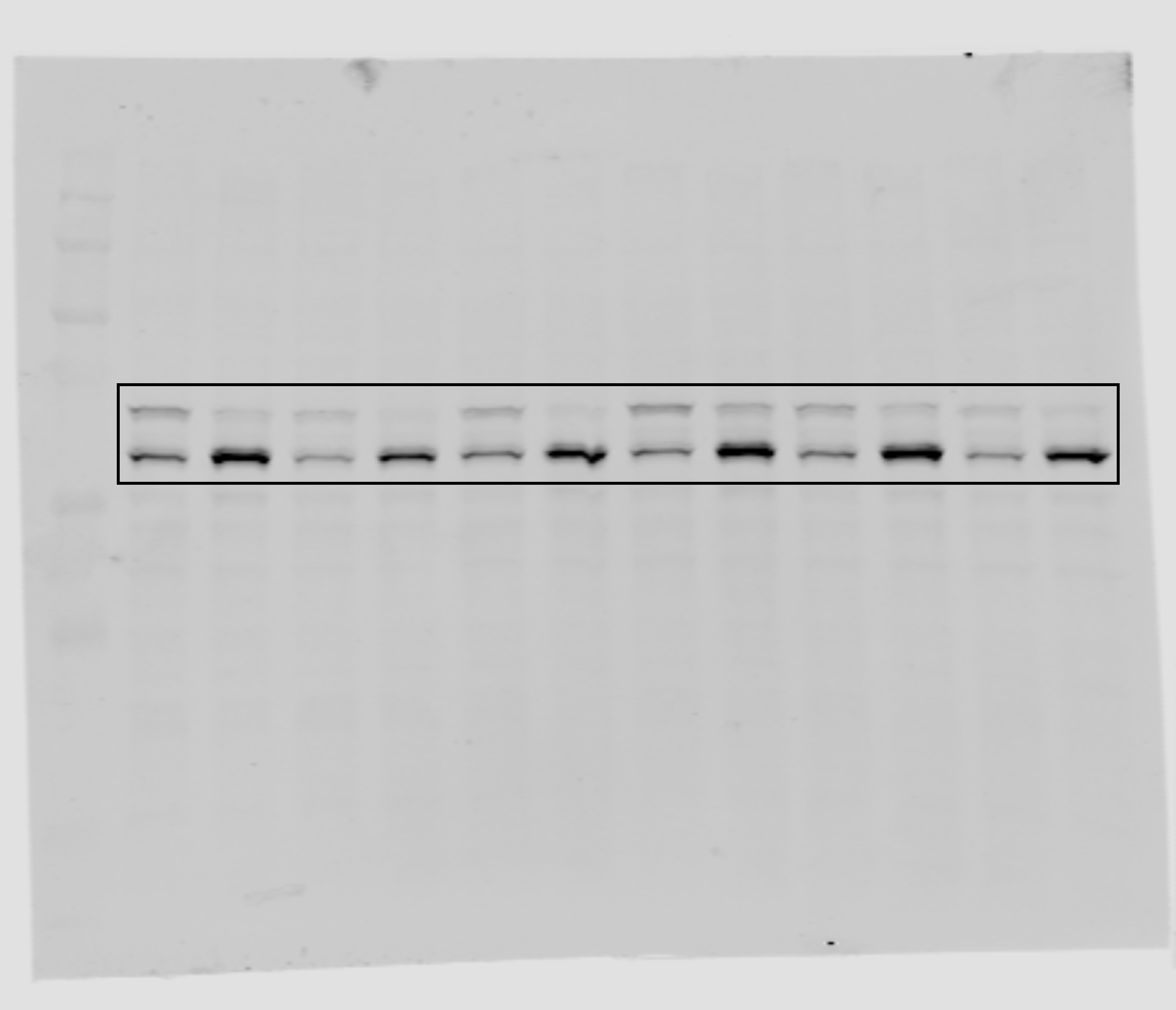

Supplement: Figure 4—figure supplement 4—source data 1. [file elife-82843-fig4-figsupp4-data1.zip › Annotated/Fig. 4-4 V5.tif]

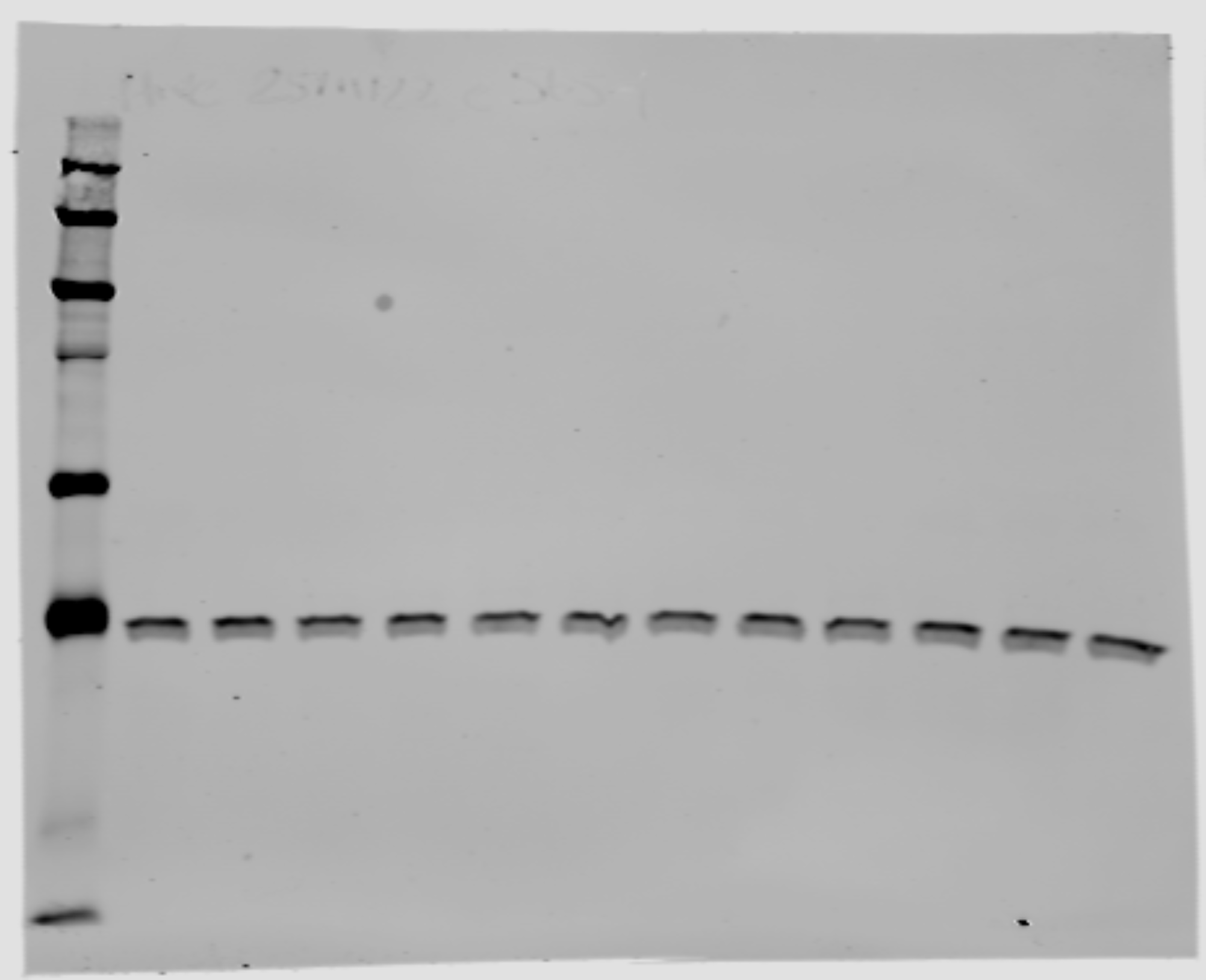

Supplement: Figure 4—figure supplement 4—source data 1. [file elife-82843-fig4-figsupp4-data1.zip › Fig. 4-4 GAPDH.tif]

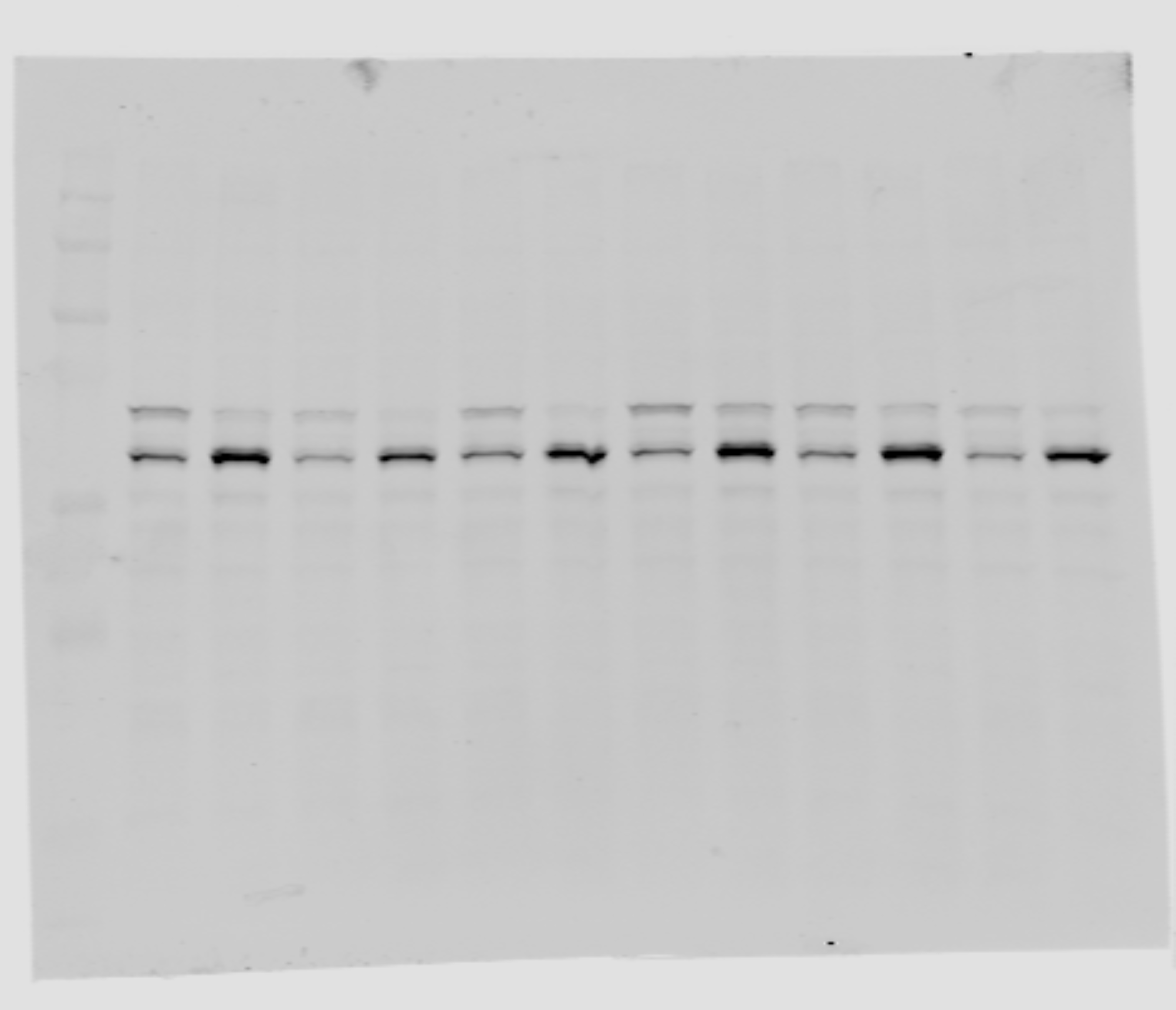

Supplement: Figure 4—figure supplement 4—source data 1. [file elife-82843-fig4-figsupp4-data1.zip › Fig. 4-4 V5.tif]

Figure 4—figure supplement 4 – V5

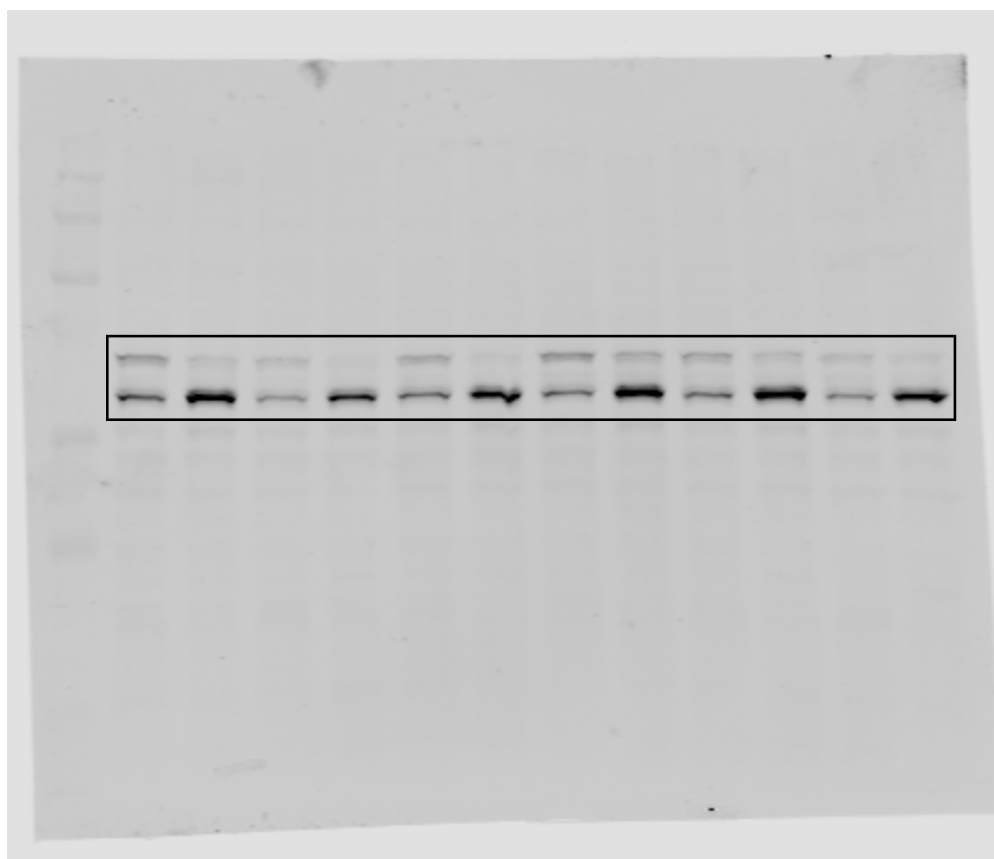

Figure 4—figure supplement 4 – GAPDH

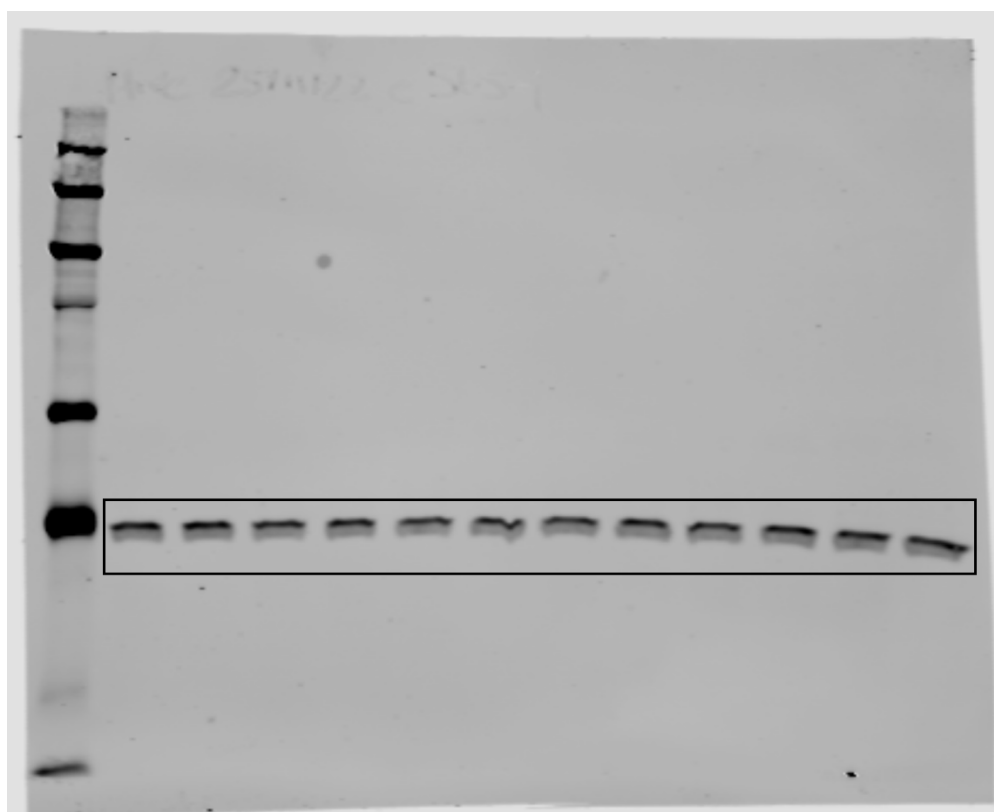

Supplement: Figure 4—figure supplement 4—source data 1. [file elife-82843-fig4-figsupp4-data1.zip › Figure 4-figure supplement 4-annotated source data.pdf]

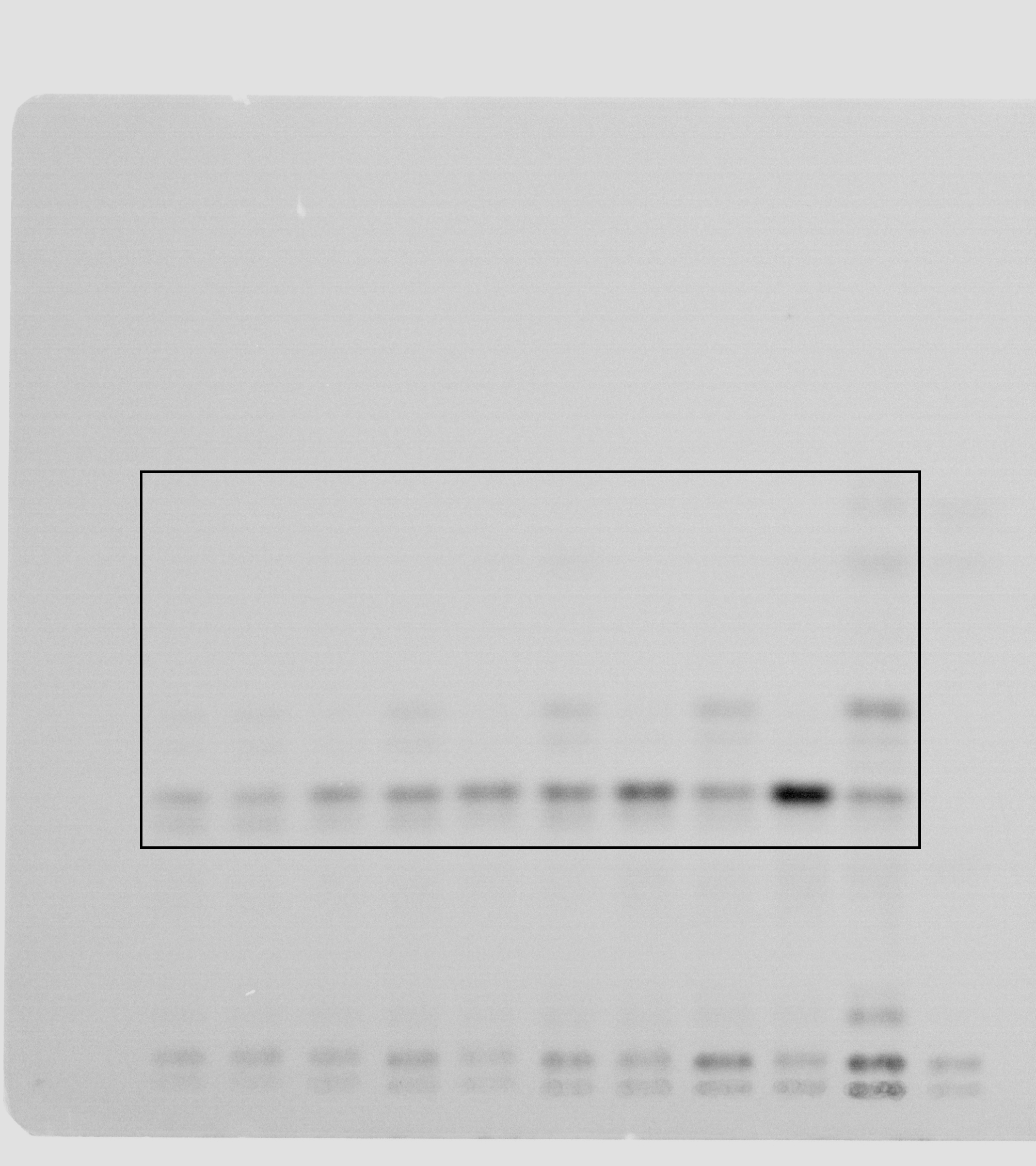

Supplement: Figure 5—source data 1. [file elife-82843-fig5-data1.zip › Annotated/Fig. 5.tif]

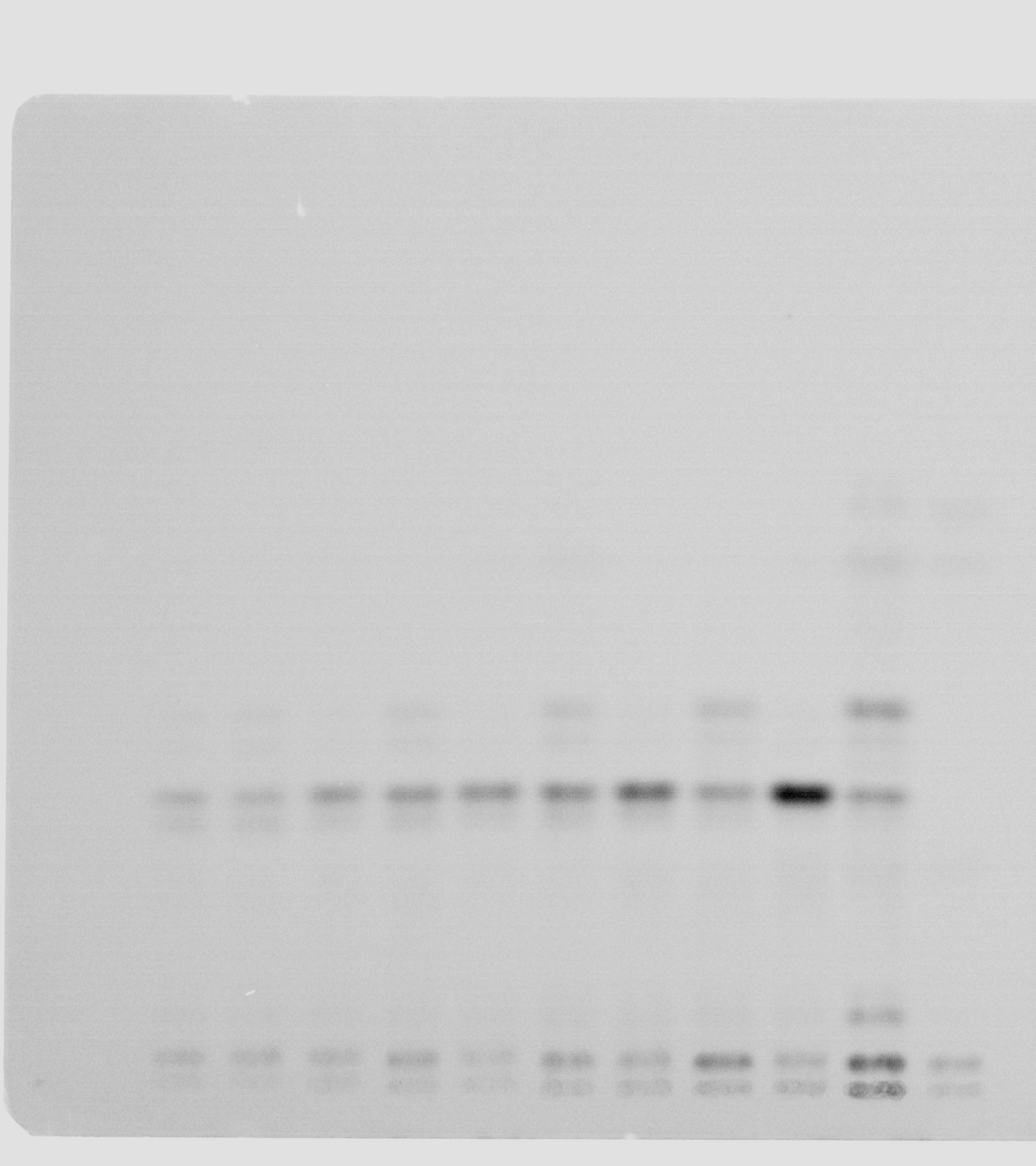

Supplement: Figure 5—source data 1. [file elife-82843-fig5-data1.zip › Fig. 5.tif]

**Figure 5**

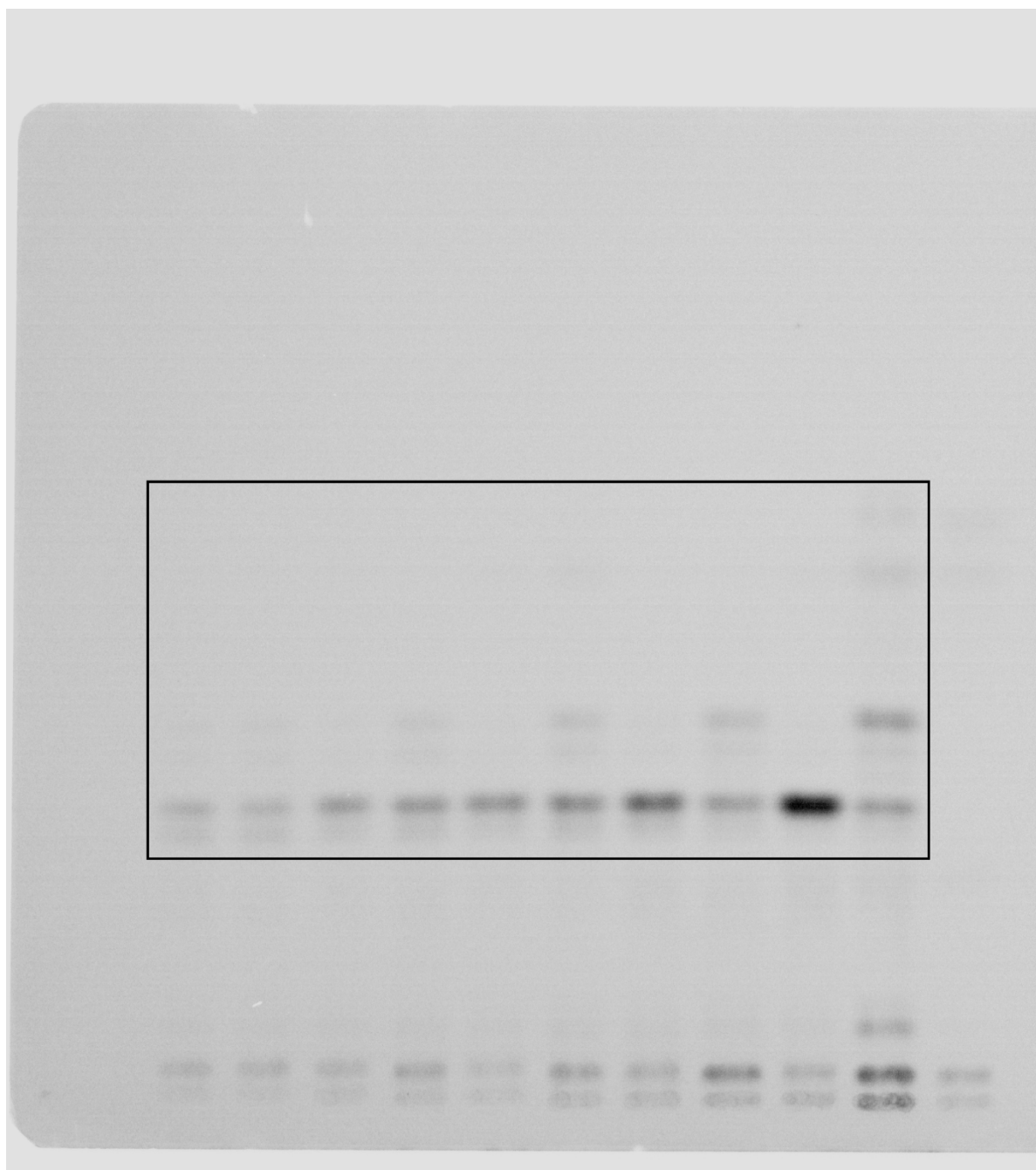

Supplement: Figure 5—source data 1. [file elife-82843-fig5-data1.zip › Figure 5-annotated source data.pdf]
