# Supplementary material for: Hypoxia truncates and constitutively activates the key cholesterol synthesis enzyme squalene monooxygenase
Source: eLife. 2023 Jan 19;12:e82843. doi: 10.7554/eLife.82843 (PMC9851614; doi:10.7554/eLife.82843)

Figure 3—figure supplement 1A – SM

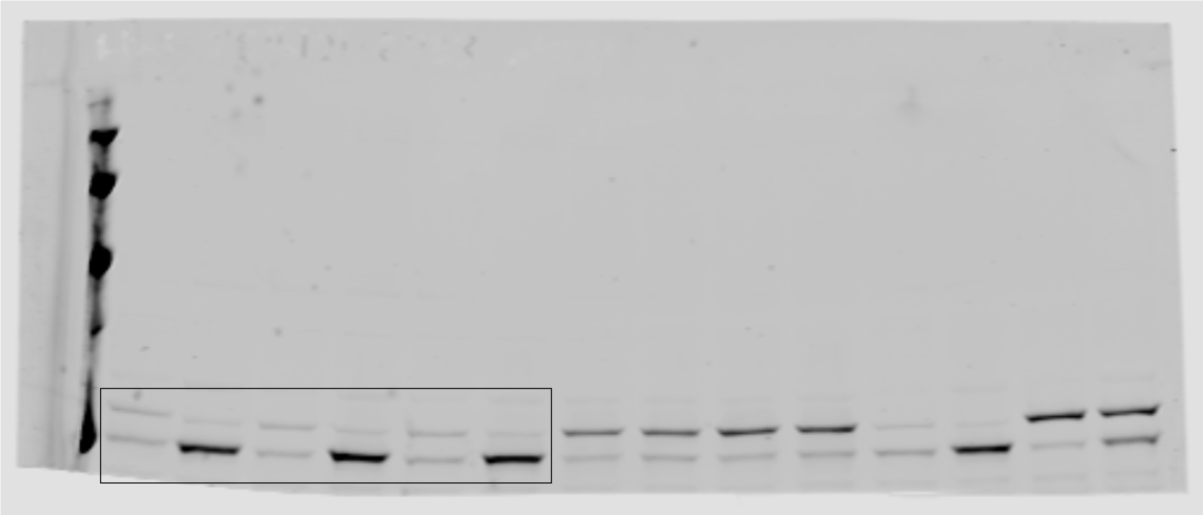

Figure 3—figure supplement 1A – V5

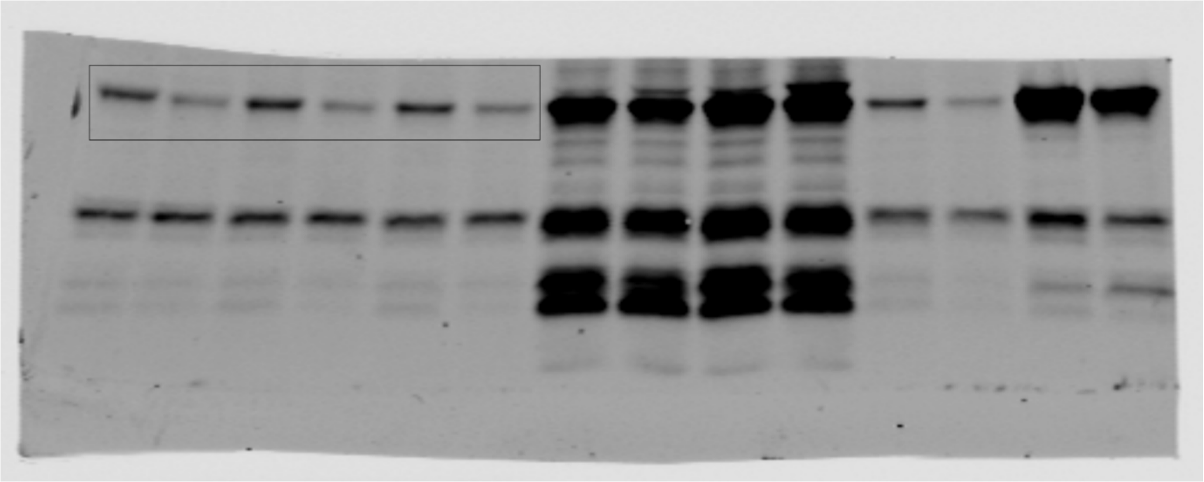

Figure 3—figure supplement 1A – HIF1α

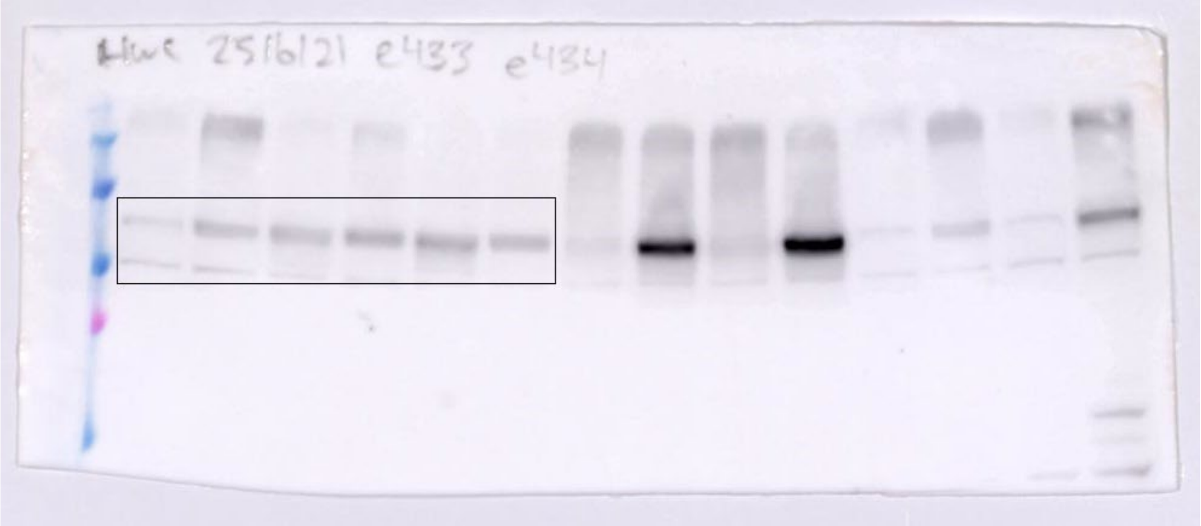

Figure 3—figure supplement 1A – GAPDH

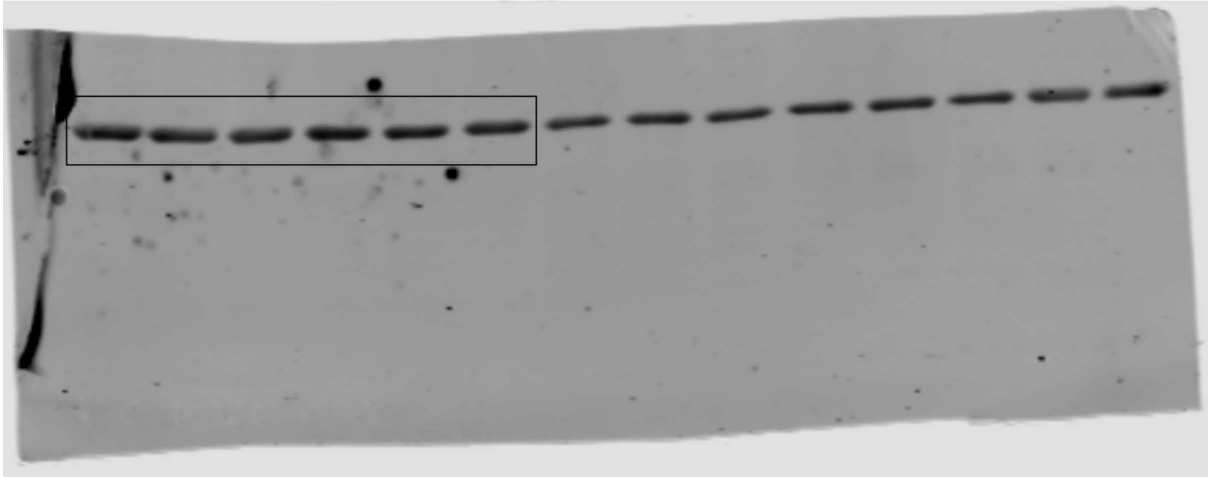

Supplement: Figure 3—figure supplement 1—source data 1. [file elife-82843-fig3-figsupp1-data1.zip › Figure 3-figure supplement 1-annotated source data.pdf]
