# Supplementary material for: Hypoxia truncates and constitutively activates the key cholesterol synthesis enzyme squalene monooxygenase
Source: eLife. 2023 Jan 19;12:e82843. doi: 10.7554/eLife.82843 (PMC9851614; doi:10.7554/eLife.82843)

Figure 4—figure supplement 2B – SM

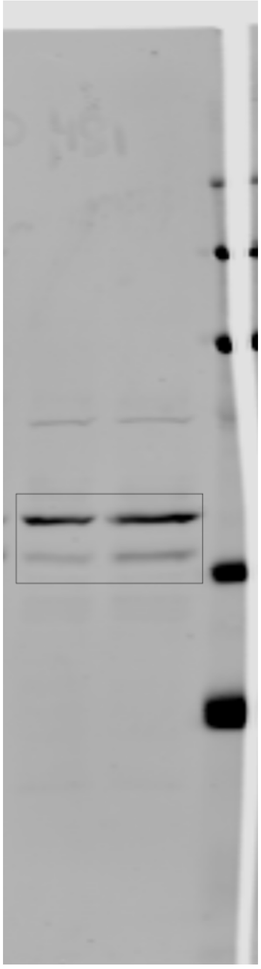

Figure 4—figure supplement 2B – GAPDH

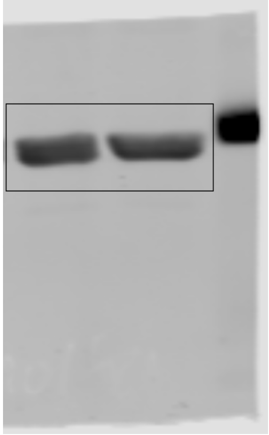

Figure 4—figure supplement 2C – SM

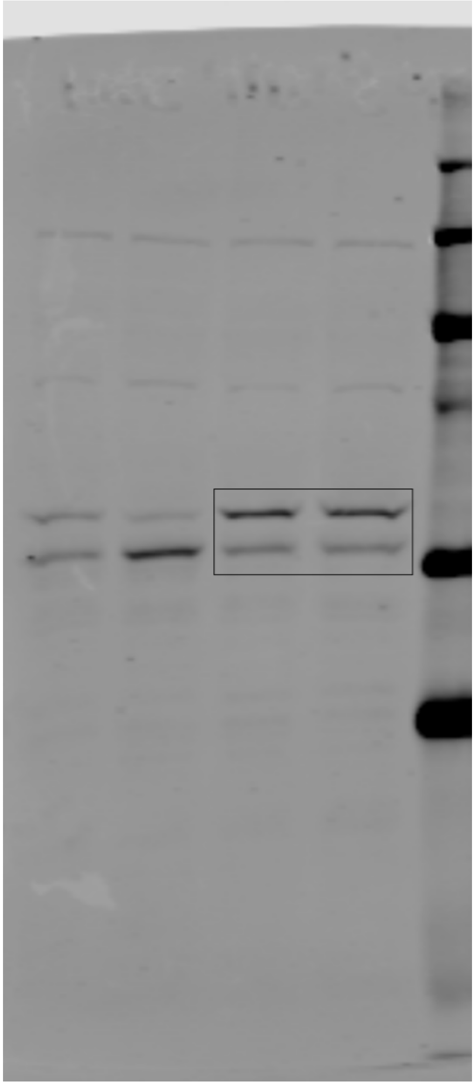

Figure 4—figure supplement 2C – GAPDH

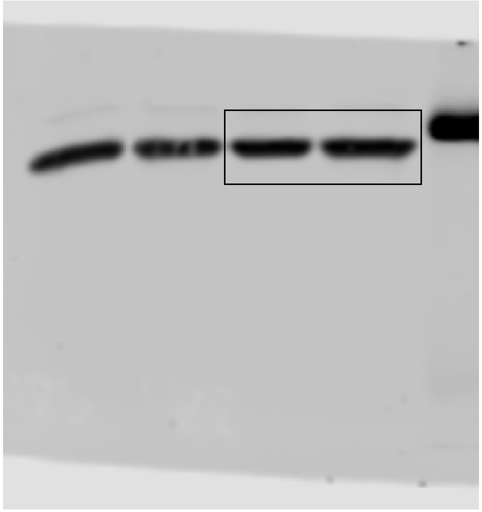

Figure 4—figure supplement 2D – V5

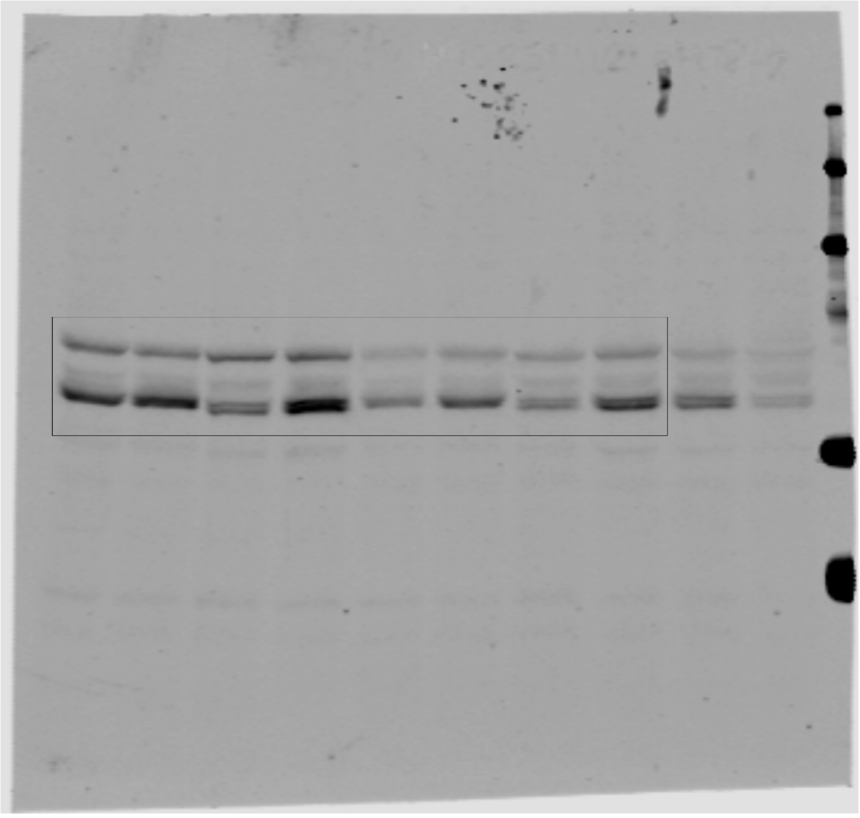

Figure 4—figure supplement 2D – GAPDH

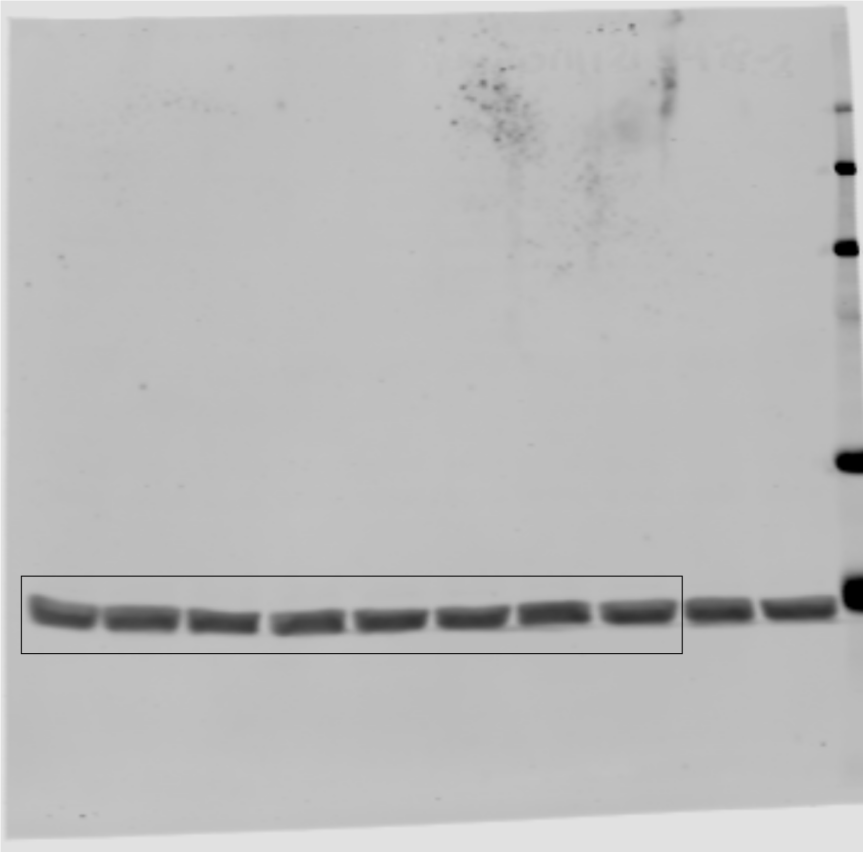

Figure 4—figure supplement 2E – SM

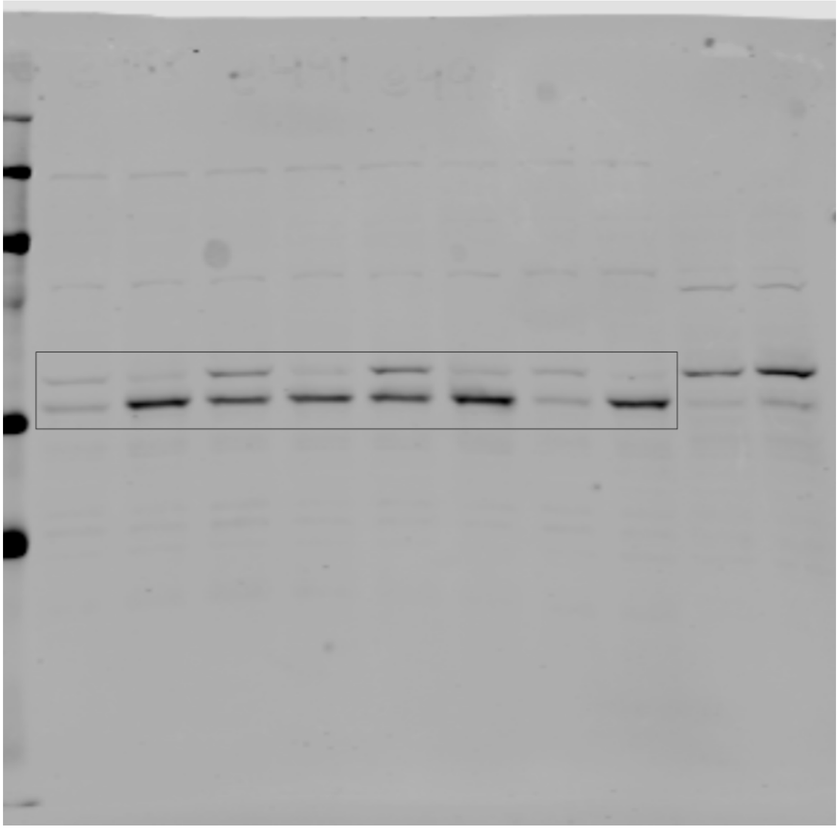

Figure 4—figure supplement 2E – V5

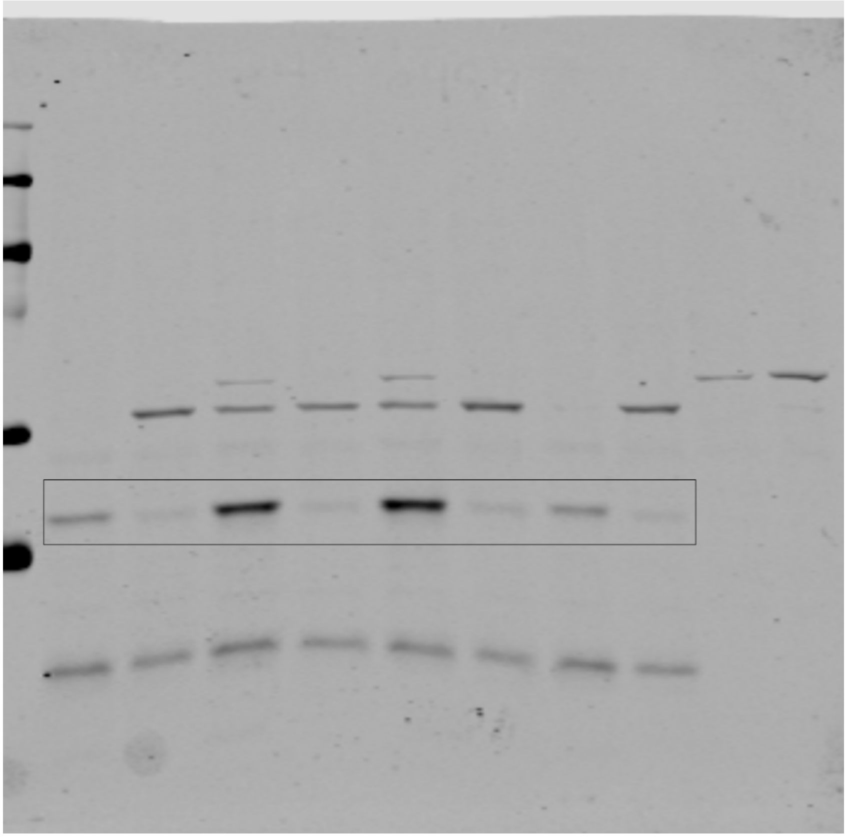

Figure 4—figure supplement 2E – GAPDH

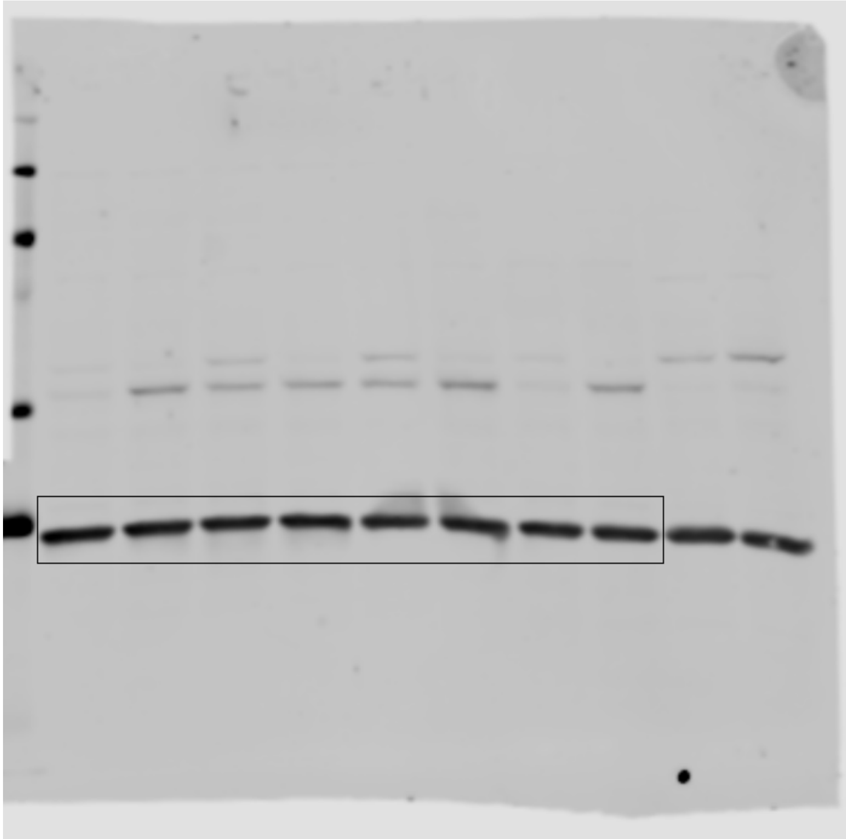

Supplement: Figure 4—figure supplement 2—source data 1. [file elife-82843-fig4-figsupp2-data1.zip › Figure 4-figure supplement 2-annotated source data.pdf]
